# Glucose-stimulated insulin secretion depends on FFA1 and Gq in neonatal mouse islets

**DOI:** 10.1007/s00125-023-05932-5

**Published:** 2023-05-23

**Authors:** Estela Lorza-Gil, Gabriele Kaiser, Christopher Carlein, Markus D. A. Hoffmann, Gabriele M. König, Sieglinde Haug, Leticia Prates Roma, Elisabeth Rexen Ulven, Trond Ulven, Evi Kostenis, Andreas L. Birkenfeld, Hans-Ulrich Häring, Susanne Ullrich, Felicia Gerst

**Affiliations:** 1 German Center for Diabetes Research (DZD e.V.), Tübingen, Germany; 2 Institute for Diabetes Research and Metabolic Diseases of the Helmholtz Zentrum München at the University of Tübingen (IDM), Tübingen, Germany; 3 Department of Internal Medicine, Endocrinology, Diabetology and Nephrology, University Hospital Tübingen, Tübingen, Germany; 4 Department of Biophysics Faculty of Medicine, Saarland University, Homburg, Germany; 5 Institute of Pharmaceutical Biology, Bonn University, Bonn, Germany; 6 Department of Drug Design and Pharmacology, University of Copenhagen, Copenhagen, Denmark

**Keywords:** *Ffar1*^-/-^ mice, Gq, Insulin secretion, Offspring islets, Parental high-fat diet

## Abstract

**Aims/hypothesis:**

After birth, the neonatal islets gradually acquire glucose-responsive insulin secretion, a process that is subjected to maternal imprinting. Although NEFA are major components of breastmilk and insulin secretagogues, their role for functional maturation of neonatal beta cells is still unclear. NEFA are the endogenous ligands of fatty acid receptor 1 (FFA1, encoded by *Ffar1* in mice), a Gq-coupled receptor with stimulatory effect on insulin secretion. This study investigates the role of FFA1 in neonatal beta cell function and in the adaptation of offspring beta cells to parental high-fat feeding.

**Methods:**

Wild-type (WT) and *Ffar1*^*−/−*^ mice were fed high-fat (HFD) or chow diet (CD) for 8 weeks before mating, and during gestation and lactation. Blood variables, pancreas weight and insulin content were assessed in 1-, 6-, 11- and 26-day old (P1–P26) offspring. Beta cell mass and proliferation were determined in P1–P26 pancreatic tissue sections. FFA1/Gq dependence of insulin secretion was evaluated in isolated islets and INS-1E cells using pharmacological inhibitors and siRNA strategy. Transcriptome analysis was conducted in isolated islets.

**Results:**

Blood glucose levels were higher in CD-fed *Ffar1*^*−/−*^ P6-offspring compared with CD-fed WT P6-offspring. Accordingly, glucose-stimulated insulin secretion (GSIS) and its potentiation by palmitate were impaired in CD *Ffar1*^*−/−*^ P6-islets. In CD WT P6-islets, insulin secretion was stimulated four- to fivefold by glucose and five- and sixfold over GSIS by palmitate and exendin-4, respectively. Although parental HFD increased blood glucose in WT P6-offspring, it did not change insulin secretion from WT P6-islets. In contrast, parental HFD abolished glucose responsiveness (i.e. GSIS) in *Ffar1*^*−/−*^ P6-islets. Inhibition of Gq by FR900359 or YM-254890 in WT P6-islets mimicked the effect of *Ffar1* deletion, i.e. suppression of GSIS and of palmitate-augmented GSIS. The blockage of Gi/o by pertussis toxin (PTX) enhanced (100-fold) GSIS in WT P6-islets and rendered *Ffar1*^*−/−*^ P6-islets glucose responsive, suggesting constitutive activation of Gi/o. In WT P6-islets, FR900359 cancelled 90% of PTX-mediated stimulation, while in *Ffar1*^*−/−*^ P6-islets it completely abolished PTX-elevated GSIS. The secretory defect of *Ffar1*^*−/−*^ P6-islets did not originate from insufficient beta cells, since beta cell mass increased with the offspring’s age irrespective of genotype and diet. In spite of that, in the breastfed offspring (i.e. P1–P11) beta cell proliferation and pancreatic insulin content had a genotype- and diet-driven dynamic. Under CD, the highest proliferation rate was reached by the *Ffar1*^*−/−*^ P6 offspring (3.95% vs 1.88% in WT P6), whose islets also showed increased mRNA levels of genes (e.g. *Fos*, *Egr1*, *Jun*) typically high in immature beta cells. Although parental HFD increased beta cell proliferation in both WT (4.48%) and *Ffar1*^*−/−*^ (5.19%) P11 offspring, only the WT offspring significantly increased their pancreatic insulin content upon parental HFD (5.18 µg under CD to 16.93 µg under HFD).

**Conclusions/interpretation:**

FFA1 promotes glucose-responsive insulin secretion and functional maturation of newborn islets and is required for adaptive offspring insulin secretion in the face of metabolic challenge, such as parental HFD.

**Graphical Abstract:**

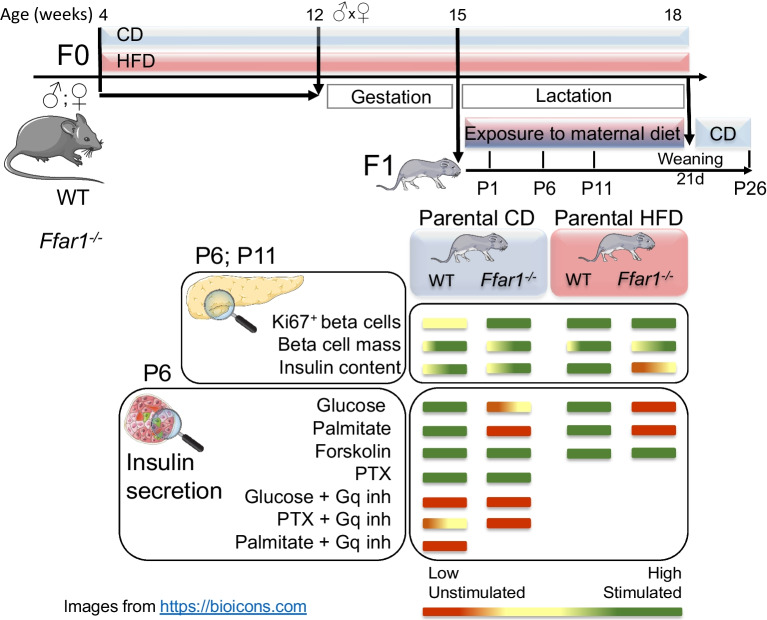

**Supplementary Information:**

The online version contains peer-reviewed but unedited supplementary material available at 10.1007/s00125-023-05932-5.


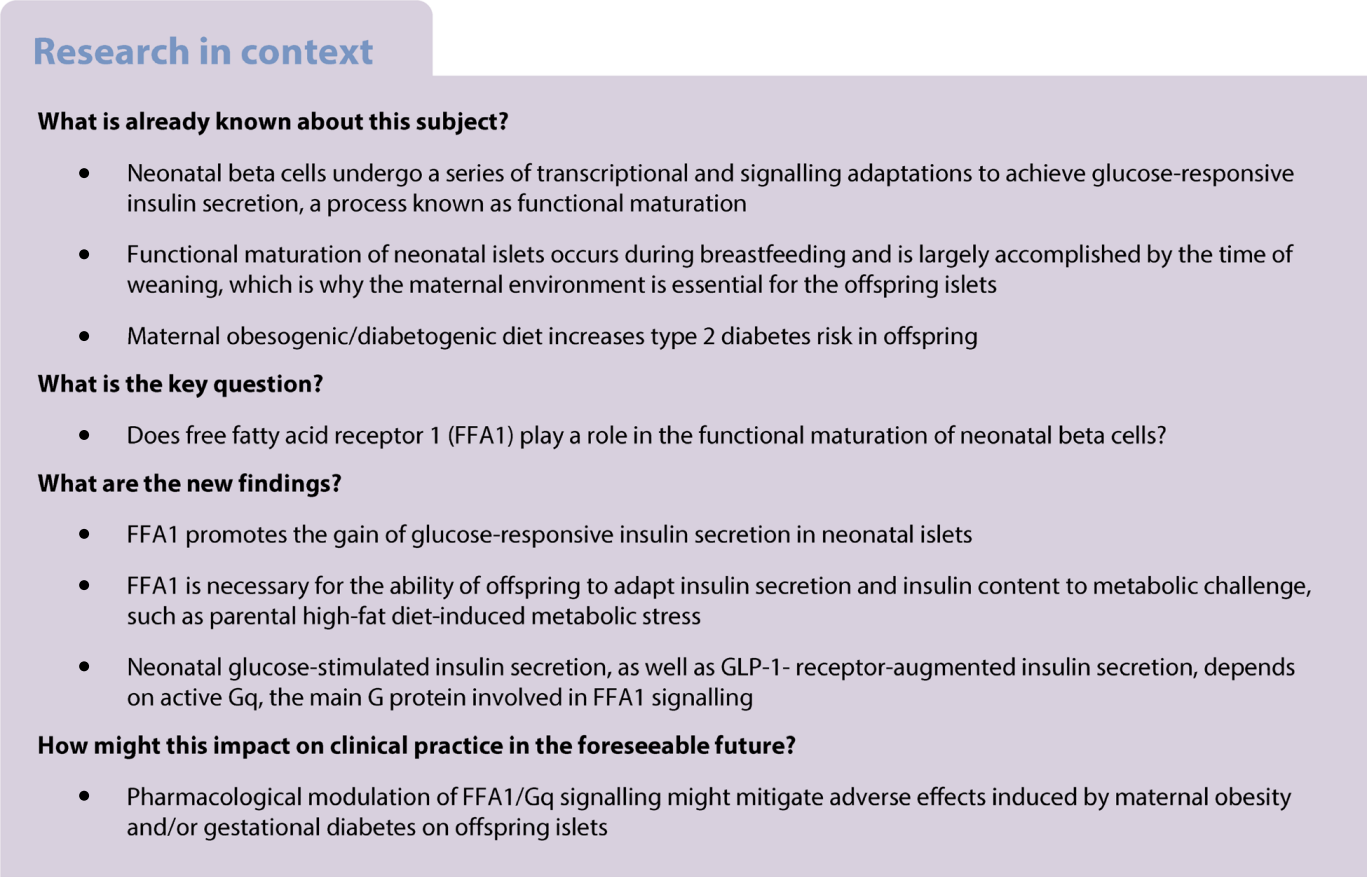


## Introduction

Obesity, a major risk factor for type 2 diabetes, requires enhanced insulin production and adaptive insulin hypersecretion. The failure of pancreatic beta cells to cope with this high insulin demand leads to a relative insulin deficiency, which is ultimately the cause of chronic hyperglycaemia and overt type 2 diabetes in humans [1–3].

Glucose-stimulated insulin secretion (GSIS), the most important functional trait of healthy beta cells, is not fully developed in newborn islets. The neonatal islets are poorly glucose responsive and their insulin secretion is largely driven by amino acids [4–6]. Glucose responsiveness develops gradually during early postnatal life and is imperative for adequate beta cell function in adulthood. Therefore, the ability of adult beta cells to meet metabolic demands depends on both the intrauterine and neonatal environment, i.e. maternal metabolism and breastmilk feeding [7–9]. The consequences of an obesogenic diet in the mother for the metabolic plasticity of beta cells in the offspring and the progeny’s risk for developing diabetes are only partly known [10–12]. In rodents, maternal feeding of a high-fat diet (HFD) during pregnancy and lactation resulted in an increase in body weight and beta cell mass, fasting hyperinsulinaemia, hyperglycaemia and glucose intolerance in the offspring [13–15]. Consistent with these findings in animal models, maternal obesity in humans is associated with an increased incidence of type 2 diabetes in the offspring [16].

Although NEFA are major components of breast milk [17] and insulin secretagogues, their role in the functional maturation of neonatal beta cells remains elusive. NEFA are endogenous agonists of free fatty acid receptor 1 (FFA1), a Gq/11 G-protein-coupled receptor (GPCR) that stimulates insulin secretion and is selectively expressed in beta cells [18–20]. Both Gq/11- and Gs-coupled receptors stimulate insulin secretion, whereas pertussis toxin (PTX)-sensitive Gi/o-coupled receptors inhibit it. Gs-coupled glucagon-like peptide-1 receptor (GLP1R) and FFA1 potentiate GSIS in a glucose-dependent manner, which is a therapeutic requirement for avoiding hypoglycaemia [21]. While GLP1R emerged as an effective tool for type 2 diabetes treatment, FFA1 has not yet progressed beyond clinical trials [22]. Different GPCRs may share one type of G protein, but there is experimental evidence that one receptor can recruit different G proteins. In adult beta cells, Gq is the major G protein recruited by FFA1, but FFA1 shows some degree of promiscuity for G proteins, e.g. Gs or G12/13, depending on the receptor ligand and cell system [23, 24]. Similarly, the Gs-coupled GLP1R can also couple to Gq and in this way maintain insulin secretion under hyperglycaemic conditions. In beta cells exposed to metabolic stress, Gq-mediated signalling is thus a potent stimulator of GSIS [25]. GPCRs also modulate beta cell survival and proliferation. While Gi/oPCRs reduce beta cell proliferation, GLP1R and FFA1 promote beta cell survival and thereby the maintenance of beta cell mass [26–28]. Distinct to adult beta cells, with their extremely low proliferation rate, neonatal beta cells are responsive to proliferative cues, and neonatal proliferation impacts on adult beta cell mass. Although a positive effect of NEFA on neonatal beta cell proliferation has been described, a role of FFA1/Gq in this is unclear [29].

The aim of the present study is to understand the role of FFA1 and Gq for proliferation and functional maturation, and specifically, the gain of glucose responsiveness of neonatal beta cells in the context of an obesogenic parental diet.

## Methods

All animal experiments were approved by the local authorities (Approval No. M10-18G and M20/21M of the Regional Council Tübingen, Germany). Animal care and handling was conducted in compliance with the German law for the protection of animals used for scientific purposes and in accordance with Animal Research: Reporting of In Vivo Experiments (ARRIVE) guidelines.

### Mouse model and feeding

**Fig. 1:**
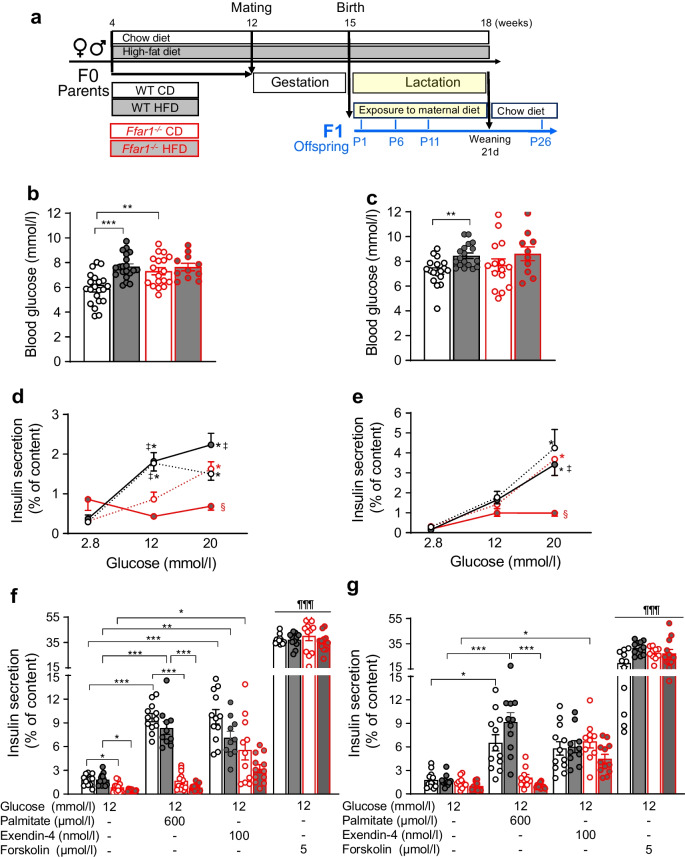
Effects of *Ffar1* deletion and parental HFD feeding on blood variables and insulin secretion in P6 and P11 offspring. (**a**) Experimental design. (**b**, **c**) Concentrations of blood glucose in (**b**) P6- and (**c**) P11-offspring. Results are expressed as mean ± SEM for *n*=9–21 animals. (**d**–**g**) Insulin secretion from islets of (**d**, **f**) P6- and (**e**, **g**) P11-offspring isolated and incubated with test substances as described under Methods. Insulin secretion is expressed as mean ± SEM for the indicated number of replicates from *n*=3 mice per genotype and diet (independent experiments); (**d**, **e**) 11–12 replicates from *n*=3 independent experiments. Black lines and dots represent WT, red lines and dots *Ffar1*^*−/−*^ mice; white- and grey-filled bars/dots represent parental CD and HFD, respectively. *p<0.05, **p<0.005, ***p<0.001 between groups as indicated. (**d**, **e**) *p<0.05 vs secretion at respective 2.8 mmol/l glucose (glucose effect); ^‡^*p*<0.05 vs *Ffar1*^*−/−*^ genotype on the same diet and the same glucose concentration (genotype effect); ^§^*p*<0.05 vs respective genotype on parental CD at the same glucose concentration (diet effect); (**f**, **g**) ^¶¶¶^*p*<0.001 vs respective 12 mmol/l glucose. d, days

C57BL/6-RipCre-ROSA^mT/mG^ (B6.Cg-Tg(Ins2-cre)25Mgn/J-B6.129(Cg)-Gt(ROSA)26Sortm4(ACTB-tdTomato,-EGFP)Luo/J) WT and *Ffar1*^*−/−*^ animals were generated as described in the electronic supplementary material (ESM) Methods. The islets of these mice display an insulin secretion similar to that of WT and *Ffar1*^*−/−*^ C57BL/6 mice, respectively (ESM Fig. 1c–e). WT and *Ffar1*^*−/−*^ animals (4 weeks old) randomly assigned to chow (CD) or high-fat diet (HFD) were fed accordingly 8 weeks before mating (Fig. 1a). WT and *Ffar1*^*−/−*^ breeding pairs were kept on the respective diet throughout gestation and lactation. Offspring animals (21 days old) were weaned on CD. Male and female offspring were examined at postnatal days 1, 6, 11 and 26 (P1–P26).

### Measurement of blood variables and Oil Red O staining

Blood variables and liver Oil Red O staining were assessed as described in the ESM Methods.

### Offspring beta cell mass, beta cell proliferation and pancreatic insulin content

P1–P26 offspring pancreases (male and female animals) were processed as described in the ESM Methods. Pancreatic area (red fluorescence) and insulin-positive area (green fluorescence) were determined using Fiji (version 1.48d). Beta cell mass (mg) was calculated by multiplying relative beta cell area (GFP-positive/Tomato-positive) by pancreas weight (mg). Beta cell proliferation was expressed as per cent of Ki67^+^/GFP^+^ of GFP^+^ cells. For insulin content, P1–P26 offspring pancreases were weighed and minced in ice-cold acid–ethanol (1.5% HCl in 75% ethanol; [vol./vol.]) to extract total insulin. Insulin was measured by ELISA.

### Islet isolation and insulin secretion

Islets were isolated from P6/P11 offspring and adult mice (males and females) and cultured as described in the ESM Methods. INS-1E cells (mycoplasma-free, kindly provided by C.B. Wollheim, University of Geneva) and isolated islets were preincubated for 1 h in KRB containing 2.8 mmol/l glucose, then incubated for 1 h in KRB containing 2.8 or 12 mmol/l glucose, supplemented with test substances as indicated in the ESM Methods. Secreted insulin and insulin content after extraction in acid–ethanol were measured by ELISA.

### INS-1E cell culture, siRNA treatment, western blotting and immunostaining

INS-1E cells were cultured and treated as described in detail in the ESM Methods.

### RNA isolation, quantitative RT-PCR and bulk RNAseq

Isolated islets, INS-1E cells, islet cell suspensions and FACS-sorted islet cells (see ESM Methods) were lysed and cellular RNA was isolated according to the instructions provided by the Nucleospin RNA isolation kit (Macherey-Nagel, Düren, Germany). Cellular RNA (0.1 µg) was transcribed into cDNA using the Transcriptor first strand cDNA synthesis kit (#04897030001, Roche Diagnostics, Indianapolis, USA). Semi-quantitative RT-PCR was performed with the LightCycler 480 system (Roche Diagnostics) using mouse/rat specific primers (Invitrogen; Thermo Fisher Scientific, Waltham, USA) as listed in ESM Table 1. *Rps13* was used as housekeeping gene. Aliquots of RNA isolated from CD WT and *Ffar1* knockout (KO) P6-offspring islets were subjected to RNAseq analysis as previously described [30].

### Cytosolic [Ca^2+^] measurement

Adult WT islets were kept in culture medium during 1 h of loading with FURA-2 AM (2 µmol/l), then transferred to KRB–Henseleit solution (KHB; ESM Methods) supplemented with 0.5% DMSO or FR900359 (1 µmol/l). The islets were then placed in KHB with 2.8 mmol/l glucose for 5 min baseline recording; thereafter, the glucose concentration was raised to 12 mmol/l and the measurement continued for 30 min. KCl (30 mmol/l) was used as positive control. When pre-treatment with Gq inhibitor was omitted, FR900359 was added during recording at high glucose.

#### Statistical analysis

Data are expressed as mean ± SEM with the number (*n*) of replicates and independent experiments as indicated. RNAseq dataset was processed with the R package DESeq2 v1.22.1. All other results were subjected to statistical ANOVA analysis using GraphPad Prism (version 9.1.2). Differences were considered statistically significant at *p*<0.05 and reported as *p<0.05, **p<0.005 and ***p<0.001. Mice and isolated islets were randomly distributed for analysis. Quantification of histological staining was performed by a trained scientist, who was masked to the experimental condition.

## Results

### Parental HFD feeding worsens glucose responsiveness of insulin secretion in *Ffar1*^*−/−*^ offspring

HFD caused significant weight gain in F0 males (Fig. 1a and ESM Fig. 2a,b). Neither HFD nor *Ffar1* deletion altered fed blood variables in F0 mice (ESM Fig. 2c–j). However, HFD induced liver steatosis in WT, and to a lesser extent in *Ffar1*^*−/−*^ animals, as previously described (ESM Fig. 2k, [19]).

The role of FFA1 in offspring glucose homeostasis was investigated by comparing WT and *Ffar1*^*−/−*^ offspring (F1) at P1–P26 (Fig. 1a and ESM Fig. 3). Parental HFD increased blood glucose in WT but not in *Ffar1*^*−/−*^ P6- and P11-offspring, respectively (Fig. 1b,c). Of note, plasma glucose of *Ffar1*^*−/−*^ P6-offspring was higher than in WT P6-offspring already under parental CD. This difference was transient, as blood glucose levels of P1-, P11- and P26-offspring were similar regardless of genotype (Fig. 1c and ESM Fig. 3a,b). HFD increased leptin, but had not effect on serum adiponectin in *Ffar1*^*−/−*^ P6 mice (ESM Fig. 3c,d). Neither parental diet nor *Ffar1* deletion altered offspring body and pancreatic weights (ESM Fig. 3e,f). These results suggest that parental HFD and FFA1 deficiency, the latter independent of parental metabolic pressure, transiently affect neonatal in vivo glucose homeostasis.

Elevated blood glucose level in the offspring could be due to impaired beta cell function. Therefore, we examined insulin secretion in P6- and P11-offspring islets (Fig. 1d–g). At P6, glucose (12 and 20 mmol/l) increased (four- to fivefold) insulin secretion of WT islets irrespective of parental diet (Fig. 1d). On the contrary, insulin secretion of *Ffar1*^*−/−*^ islets was affected by parental feeding. Thus, when exposed to parental HFD, *Ffar1*^*−/−*^ islets were glucose unresponsive, while under parental CD their insulin secretion was significantly stimulated by 20 mmol/l glucose only (Fig. 1d). Of note, at 12 mmol/l glucose, *Ffar1*^*−/−*^ islets secreted significantly less insulin than WT islets, irrespective of parental diet (Fig. 1d). *Ffar1*^*−/−*^ P6 islets displayed lower insulin content per islet than WT islets, irrespective of parental diet (ESM Fig. 3g). At P11, the negative effect of parental HFD on GSIS of *Ffar1*^*−/−*^ islets persisted at 20 mmol/l glucose. However, GSIS of *Ffar1*^*−/−*^ islets under parental CD and of WT islets under parental CD and HFD, as well as their insulin content, were similar (Fig. 1e and ESM Fig. 3h).

Endorsing the role of FFA1 in neonatal insulin secretion, palmitate increased GSIS in CD/HFD WT P6- (5.5- and 4.5-fold, respectively) and P11-islets (3.7- and 5.6-fold, respectively), but failed to augment GSIS of *Ffar1*^*−/−*^ offspring islets irrespective of parental diet (Fig. 1f,g). Unlike palmitate, exendin-4 stimulated GSIS to a similar extent in WT and *Ffar1*^*−/−*^ islets under parental CD at both P6 (five- and sixfold over GSIS, respectively) and P11 (three- and fourfold over GSIS, respectively). Parental HFD did not significantly change the exendin-4-induced effects (Fig. 1f,g). Forskolin, which bypasses GLP1R, augmented GSIS massively, independent of parental diet and genotype (Fig. 1f,g). These results indicate that FFA1 signalling promotes neonatal GSIS and the offspring’s ability to adapt insulin secretion to metabolic challenge, such as high concentration of glucose and NEFA.

### *Ffar1* KO offspring islets have increased proliferation rate

**Fig. 2:**
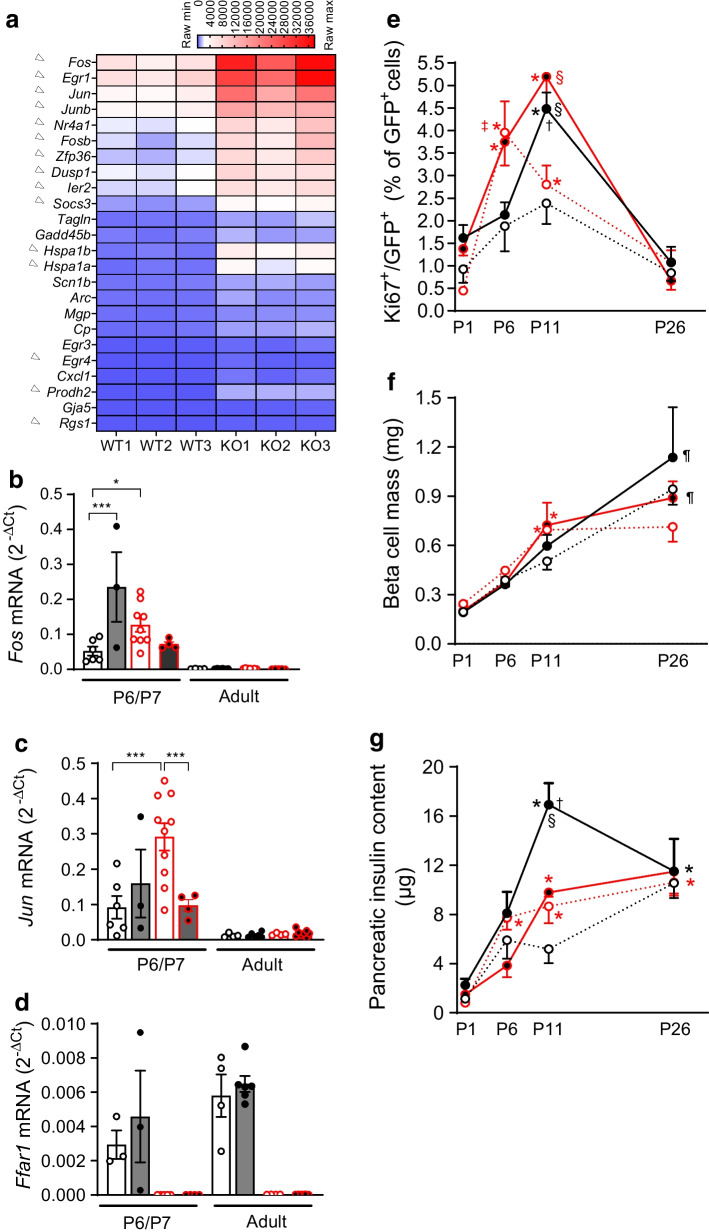
*Ffar1* deletion and parental HFD increased expression of proliferation markers in P6-islets. (**a**) Heat map showing differentially expressed genes in CD *Ffar1*^*−/−*^ P6-islets (Log_2_FC>1 over WT; adjusted *p* value <0.05); arrowhead denotes genes typically high in immature beta cells (**b**–**d**) Semi-quantitative analysis of cellular mRNA levels of P6/P7- and adult offspring islets of WT (black outlines and dots) and *Ffar1*^*−/−*^ (red outlines and dots) progenitors fed CD (white-filled bars) and HFD (grey-filled bars). Results are given as mean ± SEM for *n*=3–10 independent islet preparations. *p<0.05, ***p<0.001 between groups as indicated. (**e**) Percentage of proliferative, Ki67-positive beta cells (GFP^+^) and (**f**) total beta cell mass (mg) assessed as described under Methods. Pancreatic sections (*n*=4–8 for each animal, of *n*=3–5 animals/group were examined). (**g**) Total pancreatic insulin content (µg/pancreas) during postnatal development. Results are expressed as mean ± SEM for *n*=3–7 pancreases (independent experiments) for each group. (**e**–**g**) WT (black line and dots) and *Ffar1*^*−/−*^ (red line and dots) offspring animals from CD- (white-filled dots) and HFD- (black-filled dots) fed progenitors; *p<0.05 vs respective P1, † vs respective P6 and ¶ vs respective P11 of the same genotype and diet (time effect); ^§^*p*<0.05 vs respective genotype on parental CD at P11 (diet effect); ^‡^*p*<0.05 vs WT genotype on the same diet at P6 (genotype effect)

Since lower GSIS in *Ffar1*^*−/−*^ P6-islets might result from disturbed islet maturation, we performed comparative transcriptome analysis (RNAseq) of CD WT and *Ffar1* KO P6-islets. We found 24 up- and eight downregulated genes (+1<Log_2_ fold change [FC]<−1) in *Ffar1* KO islets (Fig. 2a, ESM Table 2). With the exception of lower *Sst* mRNA level, beta cell-specific genes and G protein mRNAs were largely unaltered in *Ffar1* KO P6-islets (ESM Table 3 and ESM Fig. 4a).

Top upregulated and highly expressed in *Ffar1*^*−/−*^ P6-islets were the early-response genes *Fos, Egr1* and *Jun*, known to promote proliferation (Fig. 2a). Furthermore, the *Ffar1*^*−/−*^ islets had increased levels of *Hes1*, *Myc* and *Sox9* mRNA, genes typically high in immature beta cells ([31], ESM Fig. 4b). Parental HFD increased the level of *Fos* and *Jun* mRNA in WT P6-islets but had an opposite effect on these genes in *Ffar1*^*−/−*^ P6-islets (Fig. 2b,c). In addition, *Hspa1a, Hspa1b* (unfolded protein response) as well as *Socs3* and *Dusp1* (cytokine signalling) were upregulated in *Ffar1*^*−/−*^ P6-islets (ESM Fig. 4c–f). Parental HFD increased these mRNAs in WT P6-islets to levels comparable to those of *Ffar1*^*−/−*^ P6-islets. In adult islets, the mRNA levels of the abovementioned genes were low and, similarly to *Ffar1*, not altered by diet or genotype (Fig. 2b–d and ESM Fig 4c–f). Gene ontology (GO) overrepresentation analysis identified enrichment for terms related to extracellular matrix (ECM) organisation, cytoskeleton, focal adhesion and endoplasmic reticulum (ER) lumen in *Ffar1*^*−/−*^ P6-islets (ESM Fig. 4h).

The top ranking upregulated gene in *Ffar1*^*−/−*^ P6-islets was *Prodh2,* encoding the low abundant hydroxyproline dehydrogenase, a mitochondrial enzyme that catabolises 4-hydroxy-proline. *Prodh2* upregulation was diet- and age-independent (ESM Fig. 4a,g and Fig. 2a). In accordance with the increased expression of *Prodh2*, and unlike in WT P6-islets, 4-hydroxy-proline, but also l-proline, improved GSIS of CD *Ffar1*^*−/−*^ P6-islets (ESM Fig. 4i,j). Thus, in *Ffar1*^*−/−*^ P6-islets PRODH2 supports amino acid-responsive insulin secretion, a functional trait of immature beta cells.

Considering the upregulation of proliferative genes in *Ffar1*^*−/−*^ P6-islets, we quantified beta cell proliferation in P1–P26 offspring pancreases (Fig. 2e and ESM Fig. 5). The percentage of Ki67-positive beta cells increased during the breastmilk feeding period, (P1–P11) and declined in weaned P26-offspring, regardless of parental diet and genotype. Notably, under CD conditions, the highest proliferation rate was achieved by *Ffar1*^*−/−*^ P6-offspring (3.95±0.69%), which had a significantly higher percentage of Ki67-positive beta cells than WT P6-offspring (1.88±0.56%) (i.e. genotype effect). Under parental HFD conditions, the peak of proliferation shifted toward P11 in both WT and *Ffar1*^*−/−*^ offspring, and their proliferation rates were significantly higher (4.48±0.35% and 5.19±0.69% in WT and *Ffar1*^*−/−*^, respectively) than those of corresponding P11 under CD (2.38±0.46% and 2.80±0.42% in WT and *Ffar1*^*−/−*^, respectively; i.e. diet effect) (Fig. 2e). In parallel, beta cell mass increased gradually from P1 to P26 (Fig. 2f). The overall increase was not altered by diet, yet its quality was slightly different between genotypes. Thus, in WT offspring beta cell mass reached the highest level at P26, whereas in *Ffar1*^*−/−*^ offspring it stopped increasing further after P11 (Fig. 2f). In parallel to the gain in beta cell mass, pancreatic insulin content steadily increased from P1 to P26. However, as long as the offspring were breastfed, solely the WT progeny were able to adjust their insulin content to parental HFD, as the insulin content of the WT P11-offspring was significantly higher under HFD (16.93±1.75 µg) than under CD (5.18±1.14 µg). Furthermore, WT P11-offspring had a higher insulin content under parental HFD than *Ffar1*^*−/−*^ P11-offspring (9.78±0.35 µg) (Fig. 2g).

These results suggest that FFA1 deficiency promotes an immature phenotype in neonatal beta cells along with increased beta cell proliferation, and it impairs the offspring’s ability to adapt insulin content to maternal HFD during breastfeeding.

### GSIS is regulated by G proteins in mouse islets

**Fig. 3:**
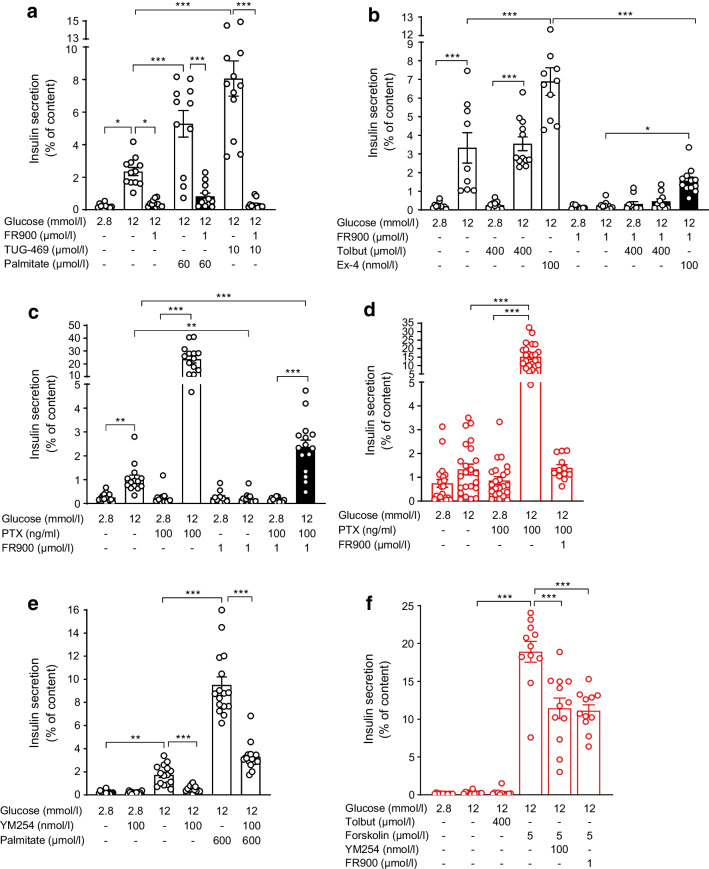
Effects of Gq inhibitors and of Gi/o inactivation on GSIS in P6-islets. (**a**–**c**, **e**) WT and (**d**, **f**) *Ffar1*^*−/−*^ P6-islets were isolated and incubated with test substances as described under Methods. Insulin secretion is expressed as mean ± SEM for the given number of replicates (3–4/experiment) from *n*=3–4 independent experiments. *p<0.05, **p<0.005, ***p<0.001 between groups as indicated. Ex-4, exendin-4; FR900, FR900359; PTX, pertussis toxin; Tolbut, tolbutamide; YM254, YM-254890

Since we found no altered expression of genes promoting glucose responsiveness and insulin secretion in *Ffar1*^*−/−*^ P6-islets, we asked whether impaired neonatal GSIS might emerge as a consequence of disturbed signalling downstream of FFA1. The major FFA1 signalling involved in stimulation of GSIS relies on Gq-coupled phospholipase C and generation of inositol 1,4,5-trisphosphate (IP_3_) and DAG (diacylglycerol), which then increase cytosolic [Ca^2+^] [32]. To assess whether Gq promotes GSIS in WT P6-islets, we used FR900359, a specific Gq inhibitor purified from *Ardisia crenata* leaves as described earlier [33]. In WT P6-islets, FR900359 inhibited palmitate- and TUG-469-amplified insulin secretion (Fig. 3a). Of note, FR900359 also abolished GSIS and its inhibitory effect persisted in the presence of tolbutamide- and exendin-4 (Fig. 3a,b).

As inhibition of GSIS upon *Ffar1* deletion or Gq inhibition may result from derepressed Gi/o-mediated inhibitory pathways, we used PTX to inactivate the Gi/o proteins [34]. PTX increased 100-fold GSIS of WT P6-islets without affecting basal secretion (Fig. 3c). FR900359 reduced the PTX effect by 90%, suggesting that GSIS largely depends on Gq when Gi/o signalling is off. Of note, the simultaneous inhibition of Gq and Gi/o (FR900359 + PTX) unmasked a 14-fold stimulation of insulin secretion by glucose in WT P6-islets (Fig. 3c). PTX-mediated inactivation of Gi/o rendered *Ffar1*^*−/−*^ P6-islets glucose responsive (tenfold stimulation), an effect cancelled by FR900359 (Fig. 3d). To confirm the role of Gq for neonatal GSIS, a second Gq inhibitor (YM-254890) was tested. YM-254890 recapitulated the effects of FR900359 on glucose and palmitate-stimulated insulin secretion in WT P6-islets (Fig. 3e). Interestingly, both FR900359 and YM-254890 reduced forskolin-stimulated insulin secretion of *Ffar1*^*−/−*^ P6-islets by 40% (Fig. 3f). These findings attest to a key role of Gq for glucose responsiveness and insulin secretion of neonatal islets.

**Fig. 4:**
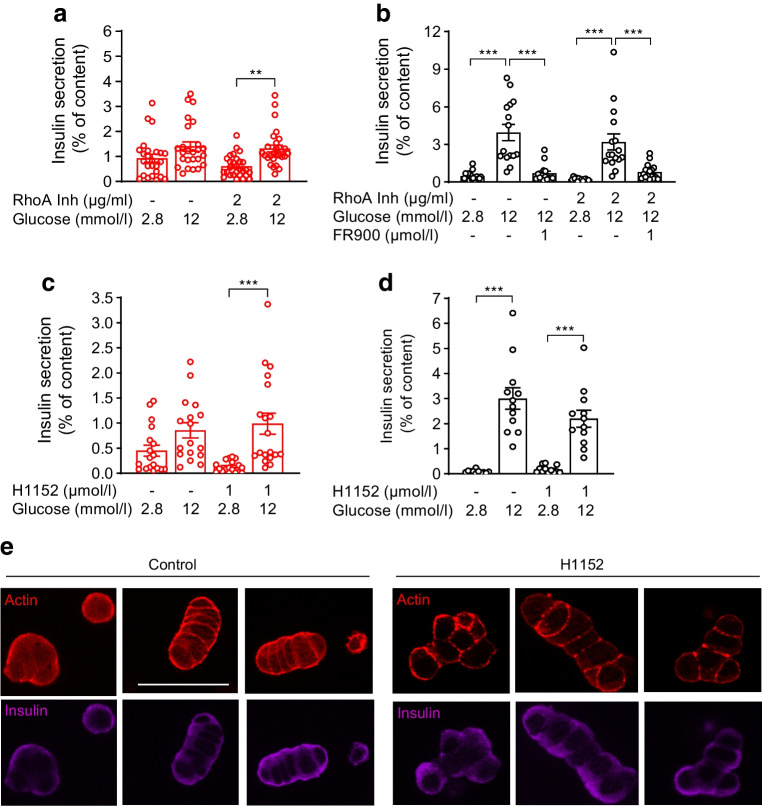
Effects of RhoA and ROCK inhibition on GSIS in WT and *Ffar1*^*−/−*^ P6-islets. (**a**, **c**) *Ffar1*^*−/−*^ and (**b**, **d**) WT P6-islets were isolated and incubated with test substances as indicated and described under Methods. Results are expressed as mean ± SEM for the given number of replicates (3–4/experiment) from *n*=3–7 independent experiments. **p<0.005, ***p<0.001 between groups as indicated. (**e**) WT P6-islet cells were isolated and cultured as described under Methods. H1152 (1 µmol/l) was added for 1 h in cell culture. The cells were stained for actin (phalloidin, red) and insulin (anti-insulin Ab, magenta). Scale bar, 40 µm. Inh, inhibition

FFA1 can also recruit G12/13, a G protein able to activate RhoA/Rho-associated protein kinase (ROCK) and trigger actin network remodelling. Since ROCK inhibition sustains functional maturation of iPS-derived beta cells [35], we examined the effect of RhoA/ROCK inhibitors in P6-islets. RhoA inhibition with exoenzyme C3 slightly improved glucose responsiveness in *Ffar1* KO P6-islets (Fig. 4a). In WT P6-islets, RhoA inhibitor neither improved GSIS nor rescued this after FR900359 treatment (Fig. 4b). Similarly, the ROCK inhibitor H1152 improved glucose responsiveness in *Ffar1*^*−/−*^ P6-islets (Fig. 4c) but had no effect in WT P6-islets (Fig. 4d), although the cortical actin web of WT beta cells became punctured upon H1152 treatment (Fig. 4e).

Together, these results indicate that GSIS in neonatal mouse islets largely depends on the activity of stimulatory Gq and inhibitory Gi/o.

### Downregulation of Gq expression in INS-1E cells mimics the effect of Gq inhibitors

**Fig. 5:**
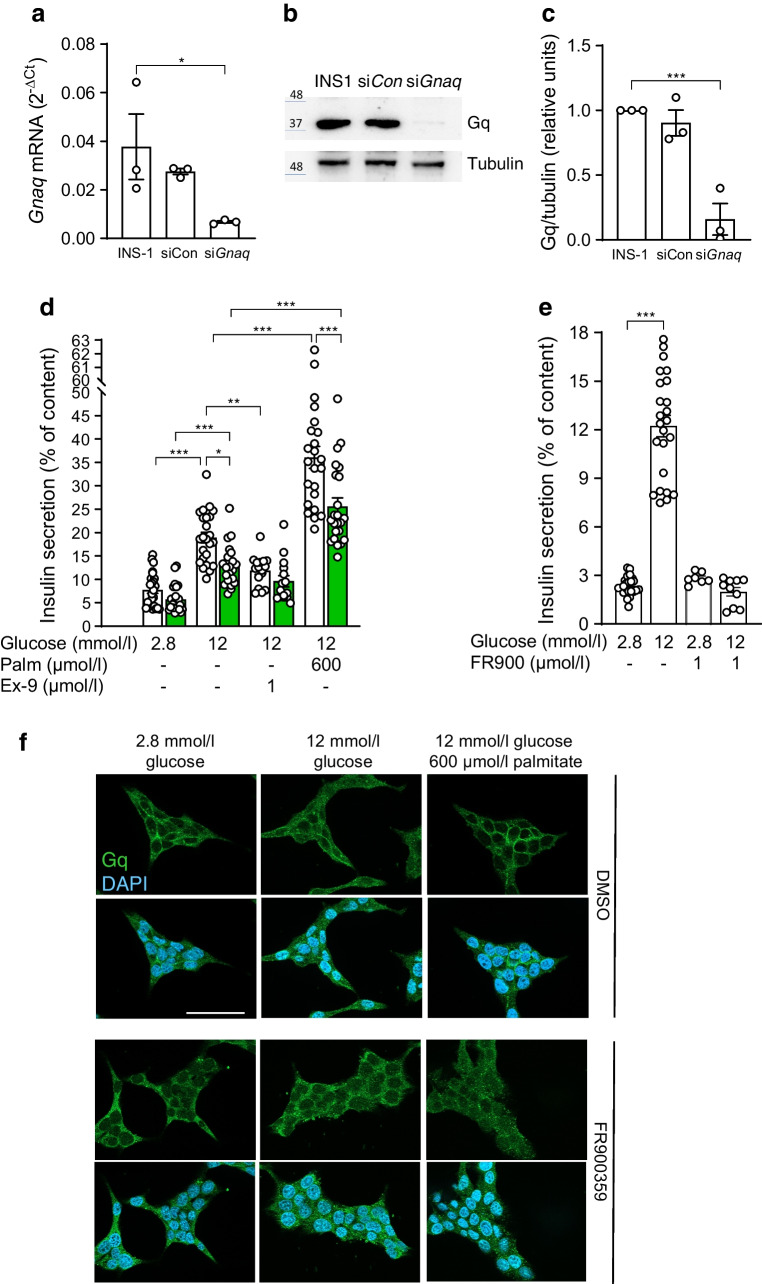
GSIS of INS-1E cells depends on Gq. (**a**–**c**) INS-1E cells were incubated, transfected and cellular RNA and proteins extracted as described under Methods. (**a**) Relative mRNA levels of *Gnaq* assessed by RT-PCR and (**b**, **c**) protein level of Gq in untransfected cells (INS-1) and upon treatment with non-targeting (siCon) and anti-*Gnaq* siRNA. Results are expressed as mean ± SEM of *n*=3 independent experiments. (**d**, **e**) Insulin secretion of (**d**) INS-1E cells upon treatment with non-targeting (white bars) and *Gnaq* (green bars) siRNA and (**e**) INS-1E cells treated as indicated. Results are expressed as mean ± SEM for the given number of replicates (3–4/experiment) from *n*=3–6 independent experiments; *p<0.05, **p<0.005, ***p<0.001 between groups as indicated. (**f**) Representative confocal images of INS-1E cells treated as described under Methods and immunostained for Gq (green). Nuclei were stained with DAPI (blue). Scale bar, 50 µm. Ex-9, exendin-(9-39); FR900, FR900359; Palm, palmitate

To further confirm the need for Gq for GSIS, we downregulated Gq expression in INS-1E cells. Following *Gnaq* siRNA treatment, mRNA and protein levels of Gq were reduced by 80% (Fig. 5a–c), and glucose- and palmitate-stimulated insulin secretion were significantly lower than in INS-1E cells transfected with control siRNA (Fig. 5d). Moreover, the GLP1R antagonist exendin-(9-39) lowered GSIS in control cells but not in INS-1E cells with downregulated Gq (Fig. 5d). Thus, GSIS of INS-1E cells depends partly on active Gq.

GSIS of INS-1E cells was also inhibited by FR900359 (Fig. 5e). Since FR900359 acts as a guanine nucleotide dissociation inhibitor for Gq [33] we next assessed via immunostaining whether FR900359 interferes with the subcellular distribution of Gq. In INS-1E cells exposed to glucose (2.8 or 12 mmol/l) ± palmitate, the Gq-associated fluorescence displayed a plasma membrane-like distribution (Fig. 5f, upper panels). In cells treated with FR900359 we detected mainly cytosolic residing Gq-associated fluorescence (Fig. 5f, lower panels). Hence, FR900359 interferes with the subcellular distribution of Gq and in this way it may also hinder GPCRs from optimal recruitment of Gq.

### GSIS and cytosolic Ca^2+^ of adult islets are sensitive to Gq inhibition

**Fig. 6:**
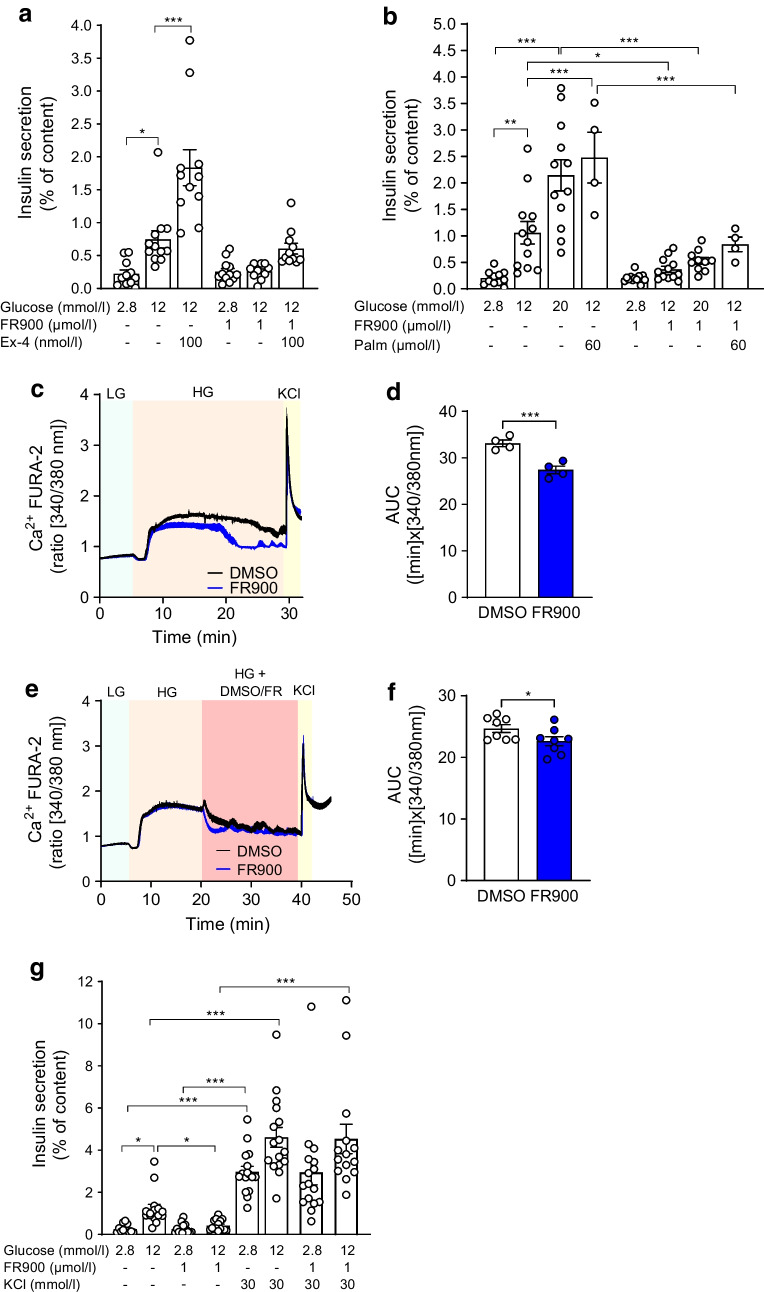
Effects of Gq inhibitor FR900359 on GSIS and cytosolic [Ca^2+^] in adult islets. (**a**, **b**, **g**) Adult WT islets were isolated and incubated as described under Methods. Insulin secretion is expressed as mean ± SEM for the given number of replicates (3–4/experiment) from *n*=3–5 independent experiments. (**c**–**f**) Cytosolic Ca^2+^ was assessed with FURA-2 in adult mouse islets cultured and treated as described in the Methods. The islets were (**c**, **d**) preincubated for 1 h with FR900359 before the recording was started or (**e**, **f**) acutely treated with FR900359 during FURA-2 recording. (**c**, **e**) Representative traces of cytosolic [Ca^2+^] measurements. (**d**, **f**) AUC of cytosolic [Ca^2+^] expressed as mean ± SEM for *n*=4–8 independent experiments. (**g**) Insulin secretion from adult WT islets isolated and incubated with test substances as described under Methods. Insulin secretion is expressed as mean ± SEM for the indicated number of replicates (3–4/experiment) from *n*=4 independent experiments. Ex-4, exendin-4; FR/FR900, FR900359; HG, high glucose (12 mmol/l); LG, low glucose (2.8 mmol/l); Palm, palmitate

To assess whether dependency of GSIS on Gq is restricted to neonatal islets, we performed GSIS in FR900359-treated adult WT islets. Similar to the neonates, FR900359 inhibited GSIS, exendin-4- and palmitate-amplified insulin secretion in adult islets (Fig. 6a,b). In view of the pronounced effect of Gq inhibitors on GSIS, we tested whether FR900359 interferes with cytosolic [Ca^2+^] in adult WT islets. Although FR900359 reduced the amplitude of [Ca^2+^] in response to 12 mmol/l glucose (17.3% of AUC glucose), it did not interfere with the initial changes to [Ca^2+^] induced by glucose (Fig. 6c,d). Acute addition of FR900359 triggered fast reduction of [Ca^2+^] without affecting the KCl-induced rise in [Ca^2+^] (8.3% of AUC glucose; Fig. 6e,f). These rather small effects of FR900359 on cytosolic [Ca^2+^] may not explain its profound effect on GSIS. In accordance with its lack of effect on KCl-induced [Ca^2+^] increase, FR900359 did not inhibit KCl-stimulated insulin secretion in WT adult islets (Fig. 6g). These observations suggest that Gq action might involve inhibition of a hyperpolarising channel.

## Discussion

In the present study, we investigated the role of FFA1 on offspring islet function during neonatal life using a well characterised *Ffar1*^*−/−*^ mouse model [19]. We show here that deletion of FFA1, as well as inhibition of its signalling partner Gq, impairs GSIS of P6-islets. Moreover, the ability of the offspring islets to sustain insulin secretion when experiencing metabolic challenge (i.e. parental high-fat feeding) depends on FFA1. As such, FFA1 deletion rendered P6- and P11-islets under parental HFD glucose unresponsive, whereas in WT P6- and P11-islets neither GSIS nor exendin-4 or palmitate-stimulated insulin secretion were affected by HFD. These observations suggest that FFA1 and Gq promote neonatal insulin secretion, especially during early life when functional maturation of beta cells is subjected to parental imprinting.

In functionally mature islets, glucose effectiveness on insulin secretion is modulated by GPCRs, that is, stimulatory Gs-coupled (GLP1R, gastric inhibitory polypeptide receptor [GIPR], glucagon receptor [GCGR]) and Gq/11-coupled (FFA1, cholinergic receptor muscarinic 3 [CHRM3]) receptors, and inhibitory Gi/o-coupled receptors (adrenoceptor alpha 2A [ADRA2A], somatostatin receptor [SSTR]) [36]. Distinct from the Gs-coupled receptors therapeutically targeted to treat insulin deficiency in type 2 diabetes, and in spite of the considerable evidence for the positive role of FFA1 for insulin secretion, the therapeutic potential of FFA1 remains controversial [19, 21, 22, 26, 37].

We provide new evidence that FFA1 and its downstream signalling partner Gq improve glucose responsiveness, and describe a Gq–Gi/o interplay. Thus, neonatal GSIS was restricted by Gi/o, as PTX-induced inactivation of Gi/o markedly increased neonatal GSIS. On the other hand, PTX-mediated increase of GSIS largely depended on Gq, as FR900359 curtailed 90% of PTX-stimulated GSIS. Nevertheless, FR900359 had no impact on KCl-augmented insulin secretion, suggesting that Gq likely acts via inhibition of a hyperpolarising K^+^-channel. Such inhibition of Gi/o-activated K^+^-channels (GIRK1/4) by Gq-coupled muscarinic receptor has been already described [38]. Previously, Sassmann et al described diminished GSIS of mouse islets with beta cell-specific deletion of Gq/11, as a consequence of impaired closure of K_ATP_-channels [39]. In agreement, we show that Gq inhibition (FR900359 and YM254890) abrogate GSIS. Although FR900359 lowered the plateau phase of glucose-induced cytosolic [Ca^2+^] increase, it did not affect the initial reduction and the subsequent rise of [Ca^2+^] triggered by glucose, implying: (1) no toxic effect on islet cell metabolism; and (2) a possible activation of hyperpolarising K^+^-channels distinct from K_ATP_-channels. In line with this, FR900359 also inhibited tolbutamide-stimulated insulin secretion.

Beside a putative Gq-mediated inhibition of hyperpolarising K^+^-channels, a role of Gq in Ca^2+^ mobilisation and Ca^2+^-dependent exocytosis [40] could explain the loss of GSIS upon Gq inhibition. Indeed, DAG generated by Gq-activated phospholipase C can interact with the SNAP receptor (SNARE) protein Munc13-1 and prime the secretory vesicles for exocytosis in chromaffin cells [41]. The sensitivity of exendin-4- and forskolin-augmented GSIS toward Gq inhibition is in disagreement with some previous observations [39, 42] but in accordance with a recent work indicating that GLP-1R/Gq signalling rescues insulin secretion of islets exposed to metabolic stress [25]. Whether such a Gs-to-Gq shift explains this discrepancy needs further experimental work.

FR900359 abrogated GSIS and siRNA-mediated downregulation of *Gnaq *impaired GSIS in INS-1E cells, suggesting a direct action of Gq in beta cells. However, we cannot exclude an action of Gq inhibitor in alpha cells, as Gq supports secretion of glucagon, which augments insulin secretion via paracrine activation of GLP1R [43].

Neonatal beta cells are proliferative, a capacity gradually lost with the acquisition of functional competence [44]. Our RNAseq results, in accordance with previous observations [31, 45], revealed elevated expression of several markers of beta cell immaturity and dedifferentiation (*Sox9*, *Hes1*, *Myc*) in *Ffar1*^*−/−*^ P6-offspring islets, a further indication that FFA1 promotes functional maturation. In fact, 15 of the 24 top-upregulated (Log_2_FC>1) genes in *Ffar1*^*−/−*^ P6-islets are highly expressed in juvenile islets and downregulated in adult islets, as previously shown (GEO: GSE87375 in [31, 46]). Several lines of evidence indicate that maternal overnutrition impairs offspring beta cell function and increases offspring risk for type 2 diabetes [10, 11]. Similarly, a loss-of-function mutation of FFA1, which is more common in severely obese individuals, is associated with impaired cytosolic [Ca^2+^] and reduced insulin secretion, indicating the importance of FFA1 for adaptive insulin secretion under conditions of lipid overload [37]. Both *Ffar1* deletion and parental HFD modulated offspring beta cell proliferation, but with different consequences. Thus, *Ffar1* deletion, in spite of promoting proliferation of P6-islets, increased neither functional beta cell mass nor pancreatic insulin content, very likely because proliferation was driven by the immature phenotype (i.e. Myc-dependent) [44]. Among the P6-offspring insulin content was the lowest in the HFD KO group, i.e. the one with the glucose-unresponsive islets. Although HFD extended the proliferative time window toward P11 in a genotype-independent manner, only WT P11-offspring increased their pancreatic insulin content, confirming the necessity of FFA1 for offspring adaptation to parental metabolic stress. These FFA1- and parental diet-related effects were limited to the pre-weaning phase, (i.e. exposure to maternal nutrition), as all P26 offspring displayed similar proliferation rates, beta cell mass and insulin content. The lipid-rich neonatal environment may therefore justify the functional relevance of FFA1 during early life, since FFA1 is the main mediator of long-chain fatty acid effects on GSIS [20, 47]. Whether Gq solely transmits the effects of FFA1 in neonatal islets is, to our knowledge, not yet known. Nevertheless, Gi and Gs were reported to regulate neonatal beta cell proliferation [28, 48].

Based on our results, we postulate that FFA1 and Gq promote GSIS in neonatal islets, and that FFA1 is required for offspring adaptation to parental fat overload. In conclusion, therapeutic modulation of FFA1/Gq signalling might protect from the adverse effects of maternal obesity on offspring glucose homeostasis.

## Supplementary Information

Below is the link to the electronic supplementary material.

Supplementary file1 (PDF 3.49 MB)

## Data Availability

The complete gene list of RNAseq data is available on request from the authors.

## Abbreviations

CD: Chow diet
FFA1: Free fatty acid receptor 1
FC: Fold change
GLP1R: Glucagon-like peptide-1 receptor
GPCR: G-protein-coupled receptor
GSIS: Glucose-stimulated insulin secretion
HFD: High-fat diet
KO: Knockout
P1–P26: Postnatal days 1, 6, 11 and 26
PTX: Pertussis toxin
ROCK: Rho-associated protein kinase
WT: Wild-type
