# Supplementary material for: Glucose-stimulated insulin secretion depends on FFA1 and Gq in neonatal mouse islets
Source: Diabetologia. 2023 May 23;66(8):1501–15. doi: 10.1007/s00125-023-05932-5 (PMC10317898; doi:10.1007/s00125-023-05932-5)
Supplement: Supplementary file 1 — Supplementary file1 (PDF 3.49 MB) [file 125_2023_5932_MOESM1_ESM.pdf]

ESM Methods

Antibodies and drugs

Drugs were purchased as following: pertussis toxin (P7208; Merck, Darmstadt, Germany); palmitic acid (#P0500; Sigma-Aldrich, St. Louis, USA), TUG-469; exendin-4 (#E7144; Merck); exendin-(9-39) (#E7269; Merck), forskolin (#F3917; Merck); FR900359 (Gq inhibitor); YM-254890 (Gq inhibitor, #10-1590; Focus Biomolecules), H1152 (#SYN1221; AdipoGen Life Scriences, Fuellinsdorf, Switzerland) CT3 toxin (#CT04; Cytoskeleton, Denver, USA); FURA-2 AM (#F1221; Invitrogen, Thermofisher Scrientific, Waltham, USA). TUG-469 was synthesized and characterised by ERU and TU and FR900359 was isolated and characterised by GMK and EK.

The antibodies used are as following: anti-Ki67 antibody (#ab15580, abcam; Cambridge, England), anti-tubulin (#2148, Cell Signalling, Danvers, USA), anti-rabbit IgG crossed adsorbed secondary antibody Alexa-Fluor 633 (#A-21070; Invitrogen), anti-rabbit IgG cross-adsorbed secondary antibody Alexa Fluor 488 (#A-11008; Invitrogen); anti-Gq antibody (#ab210004, abcam); anti-rabbit IgG HRP coupled antibody (#31460; Invitrogen).

Mouse model and feeding

mT/mG mice (STOCK Gt(ROSA)26Sor$^{tm4(ACTB\text{-}tdTomato,\text{-}EGFP)Luo/J}$, strain # 007576; https://www.jax.org/strain/007576; The Jackson Laboratory) were bred with RIP-cre mice (B6.Cg-Tg(Ins2-cre)25Mgn/J; strain #003573; https://www.jax.org/strain/003573; The Jackson Laboratory) to generate C57BL/6-RIP-Cre Tomato mice (B6.Cg-Tg(Ins2-cre)25Mgn/J-B6.129(Cg)-Gt(ROSA)26Sortm4(ACTB-tdTomato,-EGFP)Luo/J). The mT/mG mice possess loxP sites on each side of a membrane-targeted tdTomato (mT) cassette and have red fluorescent tissues. In the Cre-expressing cells of C57BL/6-RIP-Cre Tomato mice, plasma membrane-bound EGFP (mG) replaces the red fluorescent mT (ESM Fig. 1a-b). C57BL/6-RIP-CreTomato mice were bred with *Ffar1*$^{-/-}$ mice (Ffar1<tm1Heed/NPA>; https://www.informatics.jax.org/allele/MGI:3774293) to create C57BL/6-RipCre-ROSA$^{mT/mG}$ WT and *Ffar1*$^{-/-}$ animals. Mice were kept on a 12 h day-night cycle with free access to water and regular chow diet. The chow diet (CD, # D12450B; Research Diets Inc, New Brunswick, USA) contained 41.84 kJ% fat, while high fat diet (HFD; #D12451, Research Diets Inc) contained 188.28 kJ% fat.

Measurement of blood variables and Oil Red O staining

Blood samples were taken immediately after sacrification. Blood glucose was measured using a glucose analyzer (Accu-Chek; Roche Diagnostics, Mannheim,

Germany). Serum insulin, leptin and adiponectin were measured using ultrasensitive insulin (#10-1249-01; Mercodia, Uppsala, Sweden), leptin and adiponectin (#RAB0334 and #RAB1115; Millipore, Saint Louis, USA) ELISA kits, respectively. Serum NEFAs were determined using a free fatty acid quantification kit (MAK044, Sigma-Aldrich, Saint Louis, USA). Serum triglycerides were assessed using ADVIA® TRIG reagents (Siemens Healthcare Diagnostics Inc., Tarrytown NY, USA).

Lipid droplets were visualised using Oil Red O (0.5% in propylene glycol) after fixation of mouse liver cryosections (10 µm thick) with 4% formalin and dehydration with 100% propylene glycol.

Offspring beta cell mass, beta cell proliferation and pancreatic insulin content

For beta cell mass, P1-P26 offspring pancreata were fixed in 4% formalin, dehydrated (10-30% sucrose in PBS), embedded in Tissue-Tek® QC.T. compound (Sakura Finetek, Umkirch, Germany) and cut into serial 10 µm cryosections. Whole section panorama images (6-10 sections/pancreas) were captured at 10x magnification using an ApoTome System (Carl Zeiss Microscopy, Oberkochen, Germany).

For assessment of beta cell proliferation cryosections were permeabilized (0.2% Triton-X-100 in PBS), blocked with 10% FCS-PBS, incubated with anti-Ki67 antibody (1:200), followed by incubation with 2nd fluorocrome-coupled antibody (Alexa-Fluor633 IgG, 1:1000). Nuclei were stained with DAPI.

For beta cell proliferation a total number of cells (DAPI$^+$/GFP$^+$) / condition was counted as following: CD WT (P1: 1891; P6: 5252; P11: 14380; P26: 4407); CD *Ffar1*$^{-/-}$ (P1: 3547; P6: 10295; P11: 9980; P26: 5298); HFD WT (P1: 2677; P6: 6047; P11: 4281; P26: 5823); HFD *Ffar1*$^{-/-}$ (P1: 4190; P6: 4016; P11: 4576; P26: 6985)

Islet isolation and insulin secretion

P6-/P11-offspring pancreata were minced in collagenase solution (0.5 mg/ml, NB8, Serva Electrophoresis, Heidelberg, Germany) and digested for 20 min at 37ºC. Islets of adult mice were isolated by collagenase injection (1 mg/ml) into the pancreas followed by digestion for 10 min at 37ºC. Islets were washed with Hank's balanced salt solution containing (in mmol/l): 137 NaCl, 5 KCl, 1.25 $CaCl_2$, 0.81 $MgSO_4$, 1.2 Na_2HPO_4, 0.44 KH_2PO_4, 4 $NaHCO_3$, 10 Hepes pH 7.25, 2.8 glucose and 2.25 mg/ml BSA (fraction V, #A3294; Sigma-Aldrich) and collected by hand picking. Isolated islets were cultured in RPMI1640 medium containing 5 mmol/l glucose and supplemented with 10 % FCS, 10 mmol/l HEPES, 2 mmol/l L-glutamine and 1 mmol/l Na-pyruvate.

KRB contains (in mmol/l): 135 NaCl, 4.8 KCl, 1.2 Mg_2SO_4, 1.2 KH_2PO_4, 2.6 $CaCl_2$, 5 $NaHCO_3$, 10 HEPES (pH 7.4), 2.8 glucose and 0.5 % BSA-V.

For the insulin secretion assays performed with 60 µmol/l palmitate and/or TUG-469, the concentration of BSA in KRB was reduced to 0.05 %.

Test substances were added during cell culture or during the 1-2h incubation in KRB as indicated: pertussis toxin (100 ng/ml) was added for 18-20 h during culture; palmitate (60 µmol/l at 0.05% BSA and 600 µmol/l at 0.5% BSA), TUG-469 (FFA1 agonist, 10 µmol/l), exendin-4 (100 nmol/l), exendin-(9-39) (1 µmol/l) and forskolin (1 µmol/l) were added during the 1h incubation. FR900359 (1 µmol/l), YM-254890 (100 nmol/l) and H1152 (ROCK inhibitor, 1µmol/l) were added to the pre- and incubation in KRB, CT3 toxin (RhoA inhibitor, 2 µg/ml) was added to culture medium 2h previous to and during the insulin secretion assay. Secreted insulin and insulin content after extraction with acid-ethanol were measured with an ELISA kit (#10-1247-10; Mercodia, Uppsala, Sweden).

INS-1E cell culture, siRNA treatment, western blotting and immunostaining

INS-1E cells were cultured in RPRMI1640 containing in mmol/l; 11 glucose, 10 HEPES, 2 L-glutamine, 1 Na-pyruvate, 0.05 2-mercaptoethanol and 10 % FCS. The cells were transfected with 20 nmol/l non-targeting siRNA (#D-001810-10; Dharmacon, Lafayette, USA) or Gnaq siRNA (L-092425; Dharmacon) using DharmaFect3 (#T-2003; Darmacon) and were kept 3 d in culture before before lysing for RNA isolation, protein extraction or for insulin secretion assays. For protein lysates, INS-1E cells were lysed in RIPA buffer containing: 25 mmol/l Tris/HCl (pH 7.5), 150 mmol/l NaCl, 2 mmol/l EDTA, 10 mmol/l NaF, 1 mmol/l Na3VO4, 10 % glycerol, 1 % Nonidet-P40, 0.1 % SDS, 0.1 % C24H39NaO4 and protease inhibitors as previously described [30].Cellular proteins were separated on 10 % SDS-PAGE and blotted on nitrocellulose membrane. Membranes were blocked with 5 % milk-TBS-Tween (0.15 %), incubated with anti-Gq antibody and HRP-labelled 2nd antibody. Protein bands were visualised with ChemiDoc Touch Imaging System (BioRad Laboratories, Hercules, USA) and quantified using ImageLab software (version 5.2.1; BioRad).

For immunostaining, INS-1E cells seeded on poly-ornithine coated coverslips were pre-incubated for 1h in KRB containing 2.8 mmol/l glucose ± FR900359 (1 µmol/l) and then incubated for 10 min in KRB with 2.8 or 12 mmol/l glucose ± 600 µmol/l palmitate.

Cells were fixed with 4 % formalin, permeabilized with 0.2 % Triton-X100-PBS, blocked with 10 % FCS-PBS, incubated with anti-Gq antibody and fluorochrome-coupled 2nd antibody (Alexa Fluor 488 IgG). Nuclei were stained with DAPI. Cells were examined with an ApoTome System (Carl Zeiss Microscopy; 63x objective).

FACS sorting of GFP- and tdTomato-fluorescent islet cells

Isolated islets were digested with trypsin to single cell suspension. Fluorescent activated cell sorting (FACS) was performed with a BD FACSAria cell sorter (BD Biosciences; Heidelberg; Germany) using BD FACSDiva Software. Islet cells were separated in green (beta cells) and red (non-beta cells) fluorescent fractions. Successful separation of beta from non-beta cells was confirmed by the assessment of relative mRNA levels of insulin, glucagon and somatostatin using RT-PCR (ESM Fig. 1b).

Cytosolic [Ca^{2+}] measurement

KRB-Henseleit solution (KHB) contains (in mmol/l): 120 NaCl, 4.8 KCl, 1.2 $MgCl_2$, 2.5 $CaCl_2$, 24 $NaHCO_3$, 5 HEPES (pH 7.4), 2.8 glucose and 0.1 % BSA. Fluorescence was measured at 500 to 550 nm (BP 525/50 HE) with alternating 340 (BP 340/30) and 380 nm (BP 387/15) excitation using an inverted microscope (10x objective; Axio Observer 7, Carl Zeiss Microscopy). Images were taken every 2 s for max 45 min. AUC was calculated using OriginPro 2022.

ESM Table 1: List of the used primers.

ESM Table 2: RNAseq results showing expression level (normalised counts) for genes with a -0.5 > Log_2FC > +0.5 (*Ffar1* KO vs WT) in CD WT (n=3) and *Ffar1* KO (n=3) P6-islets.

ESM Table 3: RNAseq results showing expression level (normalised counts) for the top 5000 genes with the highest mean expression across all samples (n=3 CD WT and n=3 *Ffar1* KO P6-islets).

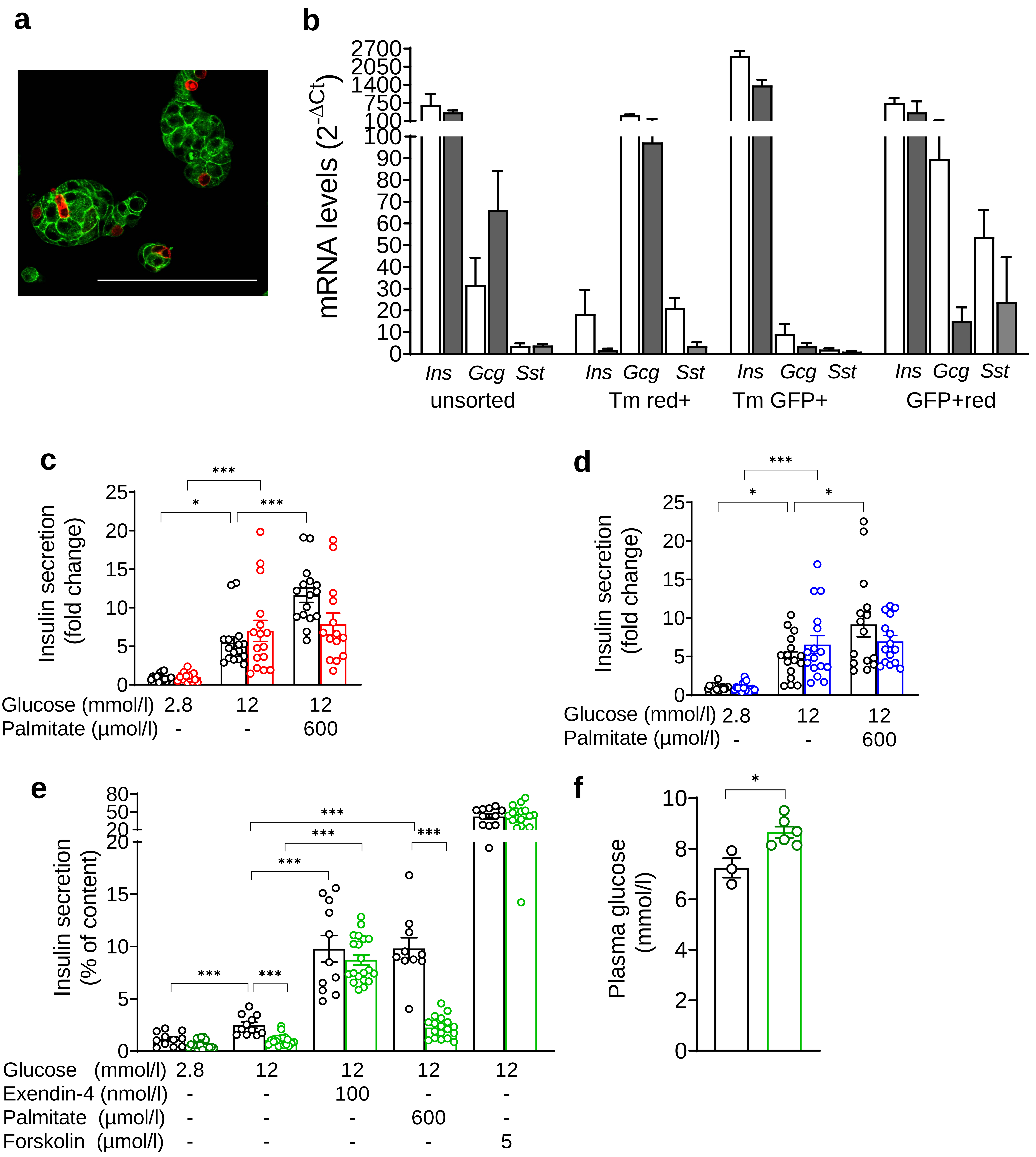

ESM Fig. 1 Transgenic RIP-Cre ROSA mT/mG mouse islets display similar insulin secretion as C57BL/6 mouse islets. (**a**) Representative confocal image of isolated WT ROSA mT/mG mouse islet cell clusters displaying green (GFP^{+}) and red (Tm^{+}) fluorescence. Scale bar, 100 µm. (**b**) Relative mRNA levels in unsorted, and FACS-sorted red (Tm^{+}) and green (GFP^{+}) islet cells of WT (white bars) and *Ffar1*$^{-/-}$ (grey bars) ROSA mT/mG mice. (**c**-**e**) Insulin secretion in isolated islets from (**c**) adult mT/mG WT (black) and *Ffar1*$^{-/-}$ (red), (**d**) adult C57BL/6 WT (black) and *Ffar1*$^{-/-}$ (blue) and (**e**) 6 d old C57BL/6 WT (black) and *Ffar1*$^{-/-}$ (green) littermates. Results are expressed as mean ± SEM for the given number of replicates (3-4 / experiment) of n = 3-6 independent experiments. (**f**) Fed plasma glucose of 6 d old C57BL/6 WT (white, n=3) and *Ffar1*$^{-/-}$ (green; n=6) littermates. Significant effects are as indicated.

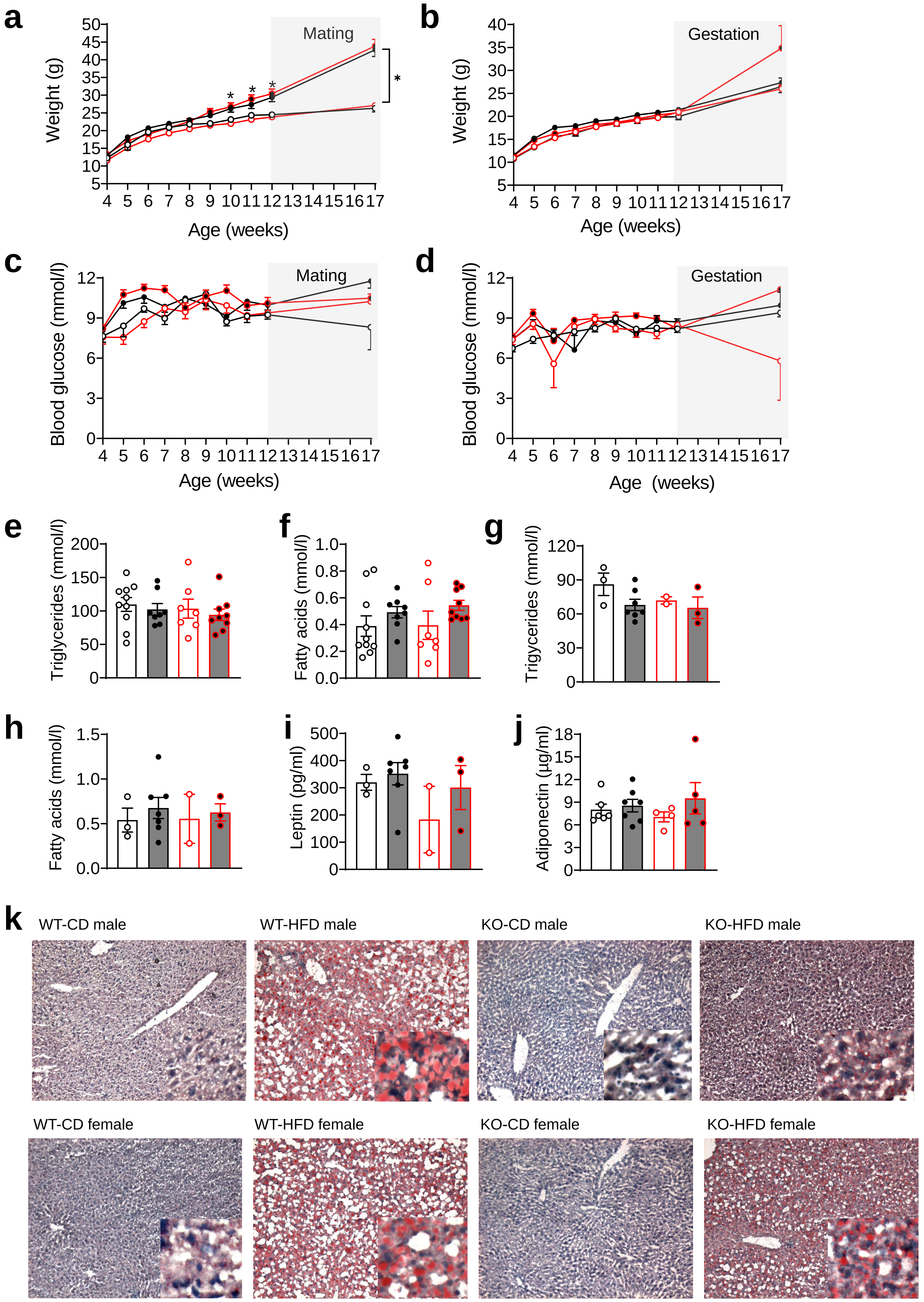

ESM Fig. 2 Effect of HFD on metabolic variables in F0 (parental) generation. Weight gain and blood glucose concentrations of WT (black lines) and *Ffar1*$^{-/-}$ (red lines) (**a**,**c**) male and (**b**,**d**) female mice fed chow (white dots) or high fat (black dots) diet. Results are expressed as mean ± SEM for n = 8-15 mice / group. (**e**-**j**) Serum triglycerides and NEFAs in (**e**,**f**) male and (**g**,**h**) female mice and (**i**,**j**) leptin and adiponectin levels in female mice of F0 generation expressed as mean ± SEM for n = 2-10 mice / group. (**k**) Liver steatosis detected with oil red O staining in cryosections of parental mouse livers collected at the end of the feeding period. Note that *Ffar1*$^{-/-}$ (KO) mice fed HFD display less fat droplets than WT mice, as previously described (ref 19).

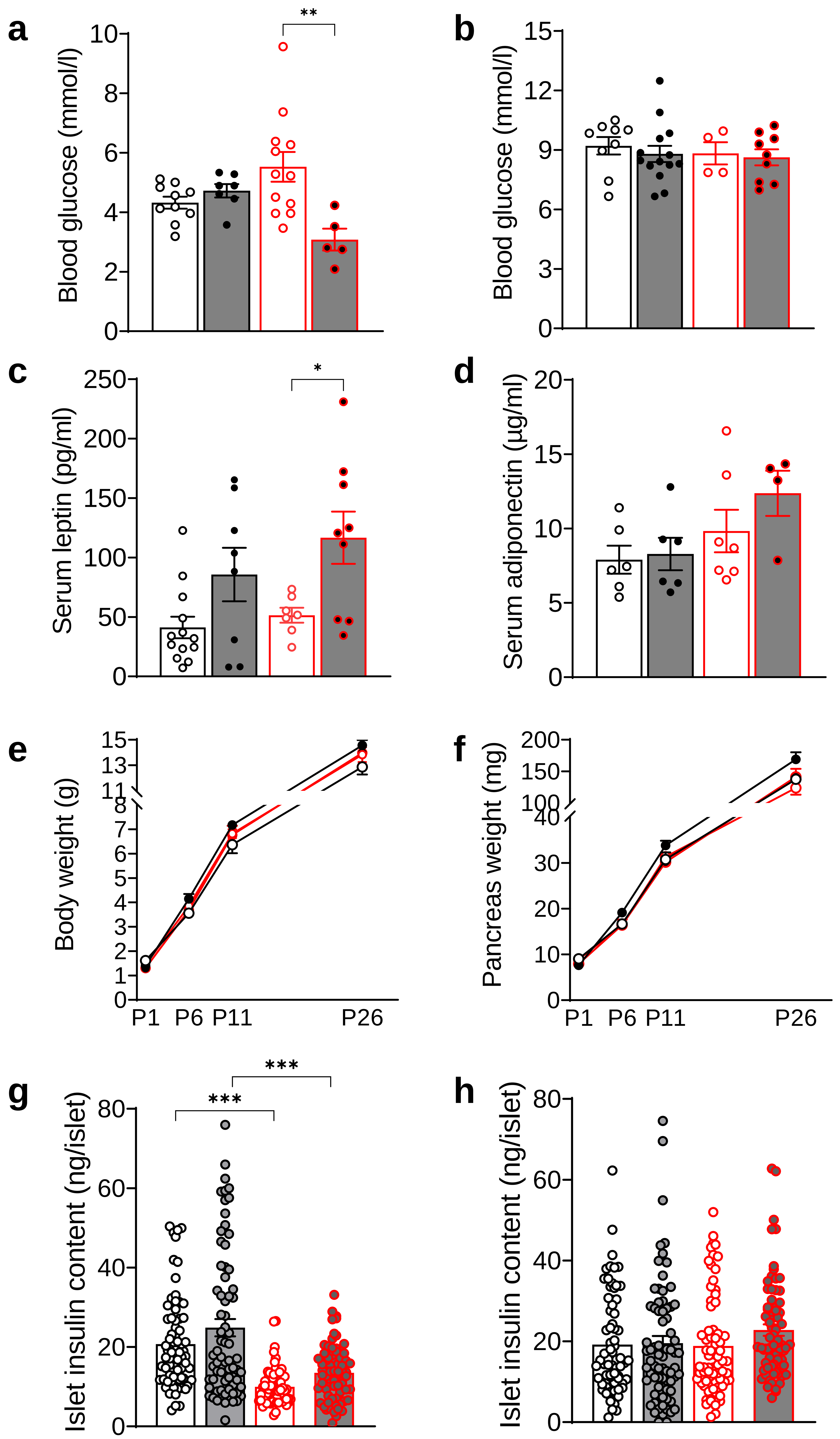

ESM Fig. 3 Effects of *Ffar1* deletion and parental HFD feeding on offspring blood variables, body and pancreatic weight, and islet insulin content. Concentrations of plasma (**a**, **b**) glucose, (**c**) leptin and (**d**) adiponectin in (**a**) P1-, (**b**) P26- and (**c**, **d**) P6-offspring. (**e**) Body weight and (**f**) pancreas weight of P1-P26 offspring. Results are expressed as mean ± SEM for n = 4-22 mice / group. (**g**, **h**) Insulin content / islet in (**g**) P6- and (**h**) P11-islets used for GSIS in Fig 1. Black lines and dots represent data from WT, red lines and dots from *Ffar1*$^{-/-}$ mice. White and grey bars represent CD and HFD offspring, respectively. Results are expressed as mean ± SEM of the respective number of replicates (n = 70-72) for each genotype and diet. Significances are as indicated.

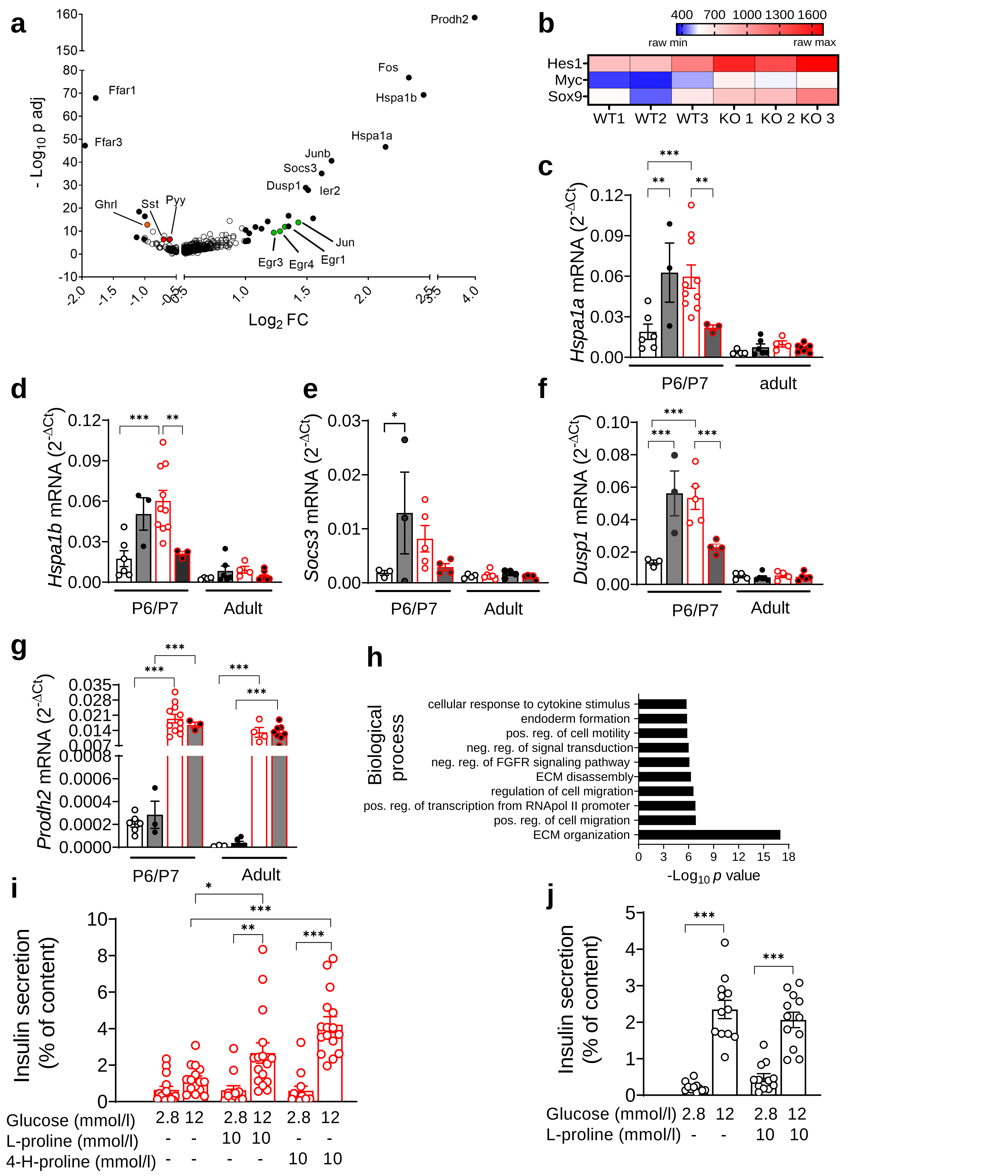

ESM Fig. 4 Transcriptome analysis and GSIS of WT and *Ffar1*$^{-/-}$ P6-islets. (**a**) Volcano plot showing differentially expressed genes (-0.5 > Log$_2$FC > 0.5; p adj <0.05) in *Ffar1*$^{-/-}$ vs WT P6-islets. (**b**) RNAseq-based heat map showing expression level of genes associated with an immature beta cell phenotype in CD WT and *Ffar1*$^{-/-}$ P6-islets. (**c**-**g**) Semiquantitative analysis of cellular mRNA levels of P6/P7- and adult islets of WT (black line and dots) and *Ffar1*$^{-/-}$ (red line and dots) offspring from CD (white columns) and HFD fed (grey columns) progenitors. Results are given as mean ± SEM for n = 3-11 independent islet preparations. (**h**) Gene ontology enrichment analysis of differentially expressed genes (-0.5 > Log$_2$FC > 0.5; p adj <0.05) of *Ffar1*$^{-/-}$ P6-islets (**i**, **j**) GSIS of (**i**) *Ffar1*$^{-/-}$ and (**j**) WT P6-islets. Results are expressed as mean ± SEM for the given number of replicates (3-4 / experiment) from n = 3-5 independent experiments. Significances are as indicated.

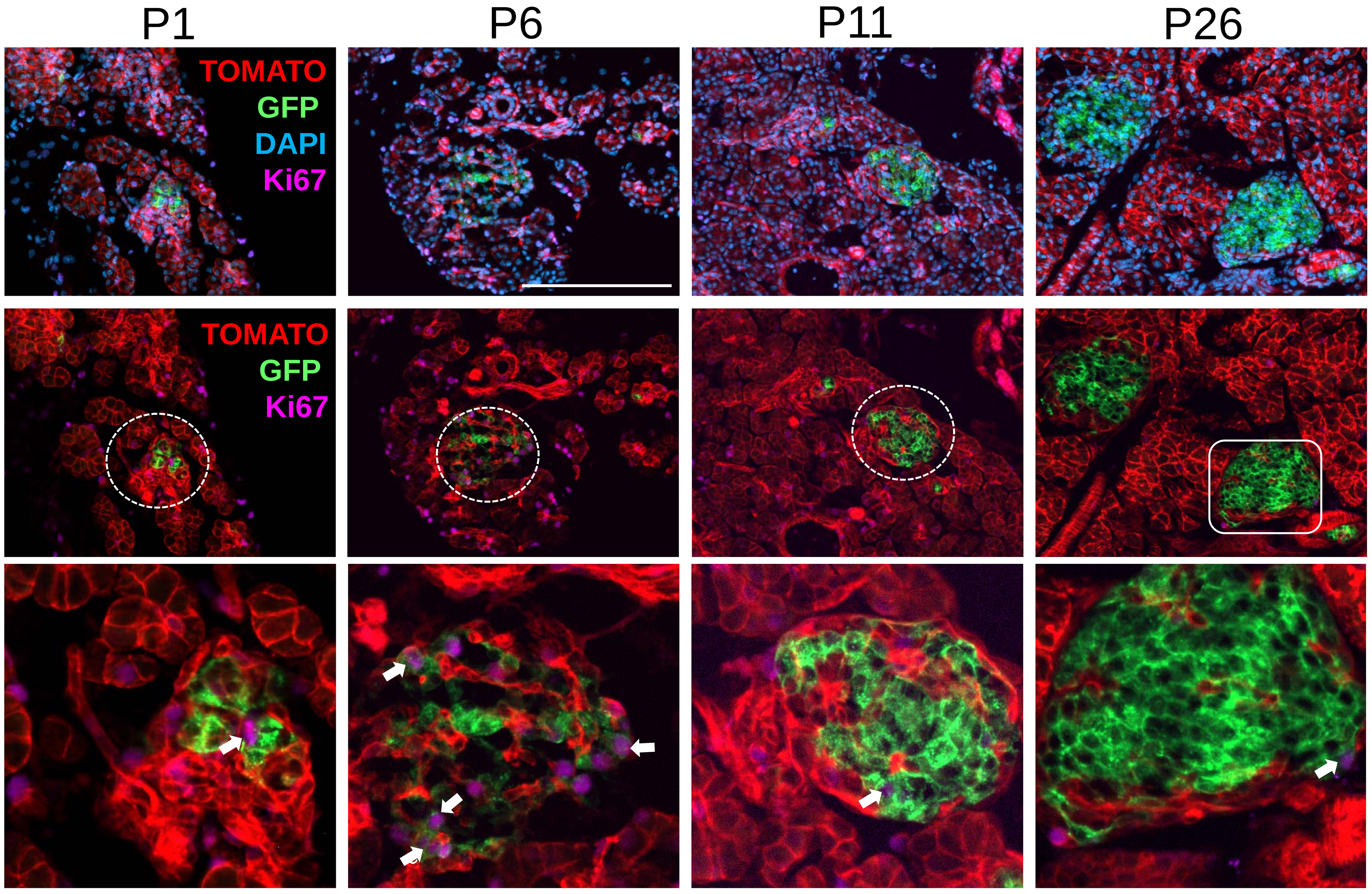

ESM Fig. 5 Proliferative beta cells detected by Ki67 immunostaining (magenta) in pancreatic sections of P1-P26 offspring. The insulin producing cells express GFP (green; see ESM Fig. 1b). Note the high number of proliferating cells in the exocrine areas (Tomato$^+$, red) of the pancreatic sections. White arrowheads indicate proliferative beta cells. Scale: 200 µm.

ESM Table 1: Primers

Gene	Fwd Seq 5'-3'	Rev Seq 5'-3'	Species
Dusp1	cactgcccaggtacaggaag	gtgcctgacagtgcagaatc	mouse
Fos	agatctgcgcaaaagtcctg	gggacagcctttcctactacc	mouse
Ffar1	CATCACTCTGCCCCTGAAG	AAGGCAAAGACTGGGCAGA	mouse
Hspa1b	tgtacacagggtggcagtgt	aaccagtatgttctttgcatttaatc	mouse
Prodh2	tcggatcaggtagggaatca	catgtctccctggcattagg	mouse
Rsp13	TGCTCCCACCTAATTGGAAA	CTTGTGCACACAACAGCATTT	mouse
Socs3	aacttgctgtgggtgaccat	atttcgcttcgggactagc	mouse
Ins	GAAGTGGAGGACCCACAAGT	AGTGCCAAGGTCTGAAGGTC	mouse
Jun	tttctcaccaactgcttgga	ccaaatgctccccaaaatac	mouse
Gcg	CCAATGCCACCACAACCT	GGCCAGCAGGAGTACTTGTC	mouse
Sst	CCCAGACTCCGTCAGTTTCT	GGGCATCATTCTCTGTCTGG	mouse
Gnaq	tctgaacgacttggaccgtg	cctcctacatcgaccattctga	rat
Rps13	CTGACGACGTGAAGGAACAA	TCACAAAACGGACCTGTGC	rat

ESM Table 2: RNAseq results of CD offspring WT and FFA1 KO P6-islets

Gene	WT1	WT2	WT3	KO1	KO2	KO3	Log2FC	padj
	♀	♂	♀	♂	♂	♂	(KOvsWT)	
Prodh2	25.6	29.2	14.1	1153.9	1205.4	1227.3	3.99	7.6334E-160
Hspa1b	395.3	515.6	387.9	4682.5	3033.0	4162.0	2.45	4.67316E-70
Fos	6419.9	4280.0	6384.9	33540.5	25522.1	36521.7	2.33	1.41906E-77
Hspa1a	286.6	414.5	356.7	3142.2	1939.5	2858.6	2.14	2.07891E-47
Junb	3194.5	3262.4	4215.6	14928.5	11945.3	13416.3	1.70	2.46572E-41
Socs3	917.4	753.4	945.1	3099.7	2541.2	3430.2	1.62	7.55134E-36
Cxcl1	24.5	15.5	54.4	411.9	166.3	344.2	1.55	2.69552E-16
Ier2	1735.8	1717.3	2341.6	6791.7	5356.6	7137.5	1.51	1.39888E-28
Dusp1	2133.2	1888.6	2522.9	7978.5	5714.9	6748.0	1.49	1.16882E-29
Jun	3412.9	3028.2	4678.1	22535.5	14281.1	21563.7	1.43	1.6076E-14
Cp	263.2	291.5	406.0	961.8	993.9	1246.3	1.35	2.18957E-17
Zfp36	1411.8	1212.6	1820.6	7636.7	5517.8	9533.3	1.35	8.03831E-13
Egr1	6628.7	5459.8	8475.5	28528.9	22969.6	36782.5	1.32	1.26125E-12
Egr4	10.7	5.5	21.2	245.2	98.6	99.6	1.28	1.21011E-10
Egr3	53.3	43.7	74.6	242.0	182.8	436.6	1.23	4.47505E-10
Gadd45b	483.8	321.6	532.0	1264.3	900.5	1009.9	1.18	6.39006E-15
Tagln	329.3	397.2	429.2	1005.3	923.1	1461.9	1.13	9.36663E-12
Scn1b	374.0	396.3	503.8	1025.4	1147.9	952.0	1.08	1.61718E-12
Arc	347.4	324.3	343.6	1077.5	689.0	798.9	1.03	8.97096E-10
Nr4a1	2038.4	1867.6	2319.4	7923.3	5505.5	10728.0	1.02	1.04704E-06
Rgs1	36.2	27.3	17.1	110.4	112.9	137.7	1.02	2.2494E-06
Fosb	1785.8	1055.9	1817.6	7587.9	5079.4	11695.3	1.01	1.78723E-06
Mgp	325.0	286.1	364.7	662.4	718.7	762.7	1.00	3.15137E-11
Gja5	25.6	13.7	35.3	79.6	106.8	183.9	1.00	2.8598E-06
Sele	18.1	5.5	25.2	152.9	44.2	221.9	0.99	2.00465E-06
Thbs1	2254.7	1801.1	1781.4	4540.2	3505.3	4313.3	0.97	6.42794E-12
Lmod1	51.1	56.5	57.4	136.9	150.9	285.3	0.92	1.84917E-05
Cntnap2	431.5	295.2	367.8	633.7	728.0	747.3	0.90	1.45207E-08
Itih5	690.5	761.6	1012.6	1582.8	1593.5	2046.1	0.90	5.7048E-08
Btg2	7289.4	6000.9	6820.1	11914.7	11070.5	12693.5	0.87	3.60068E-15
Plcd3	44.8	86.5	39.3	155.0	166.3	192.9	0.87	3.00584E-05
Ccn1	630.8	573.0	1079.1	3105.0	2465.2	4816.9	0.87	8.97613E-05
C1s1	596.7	591.3	708.3	1088.1	1234.2	1510.8	0.86	7.60022E-08
Mfap5	7.5	4.6	11.1	70.1	52.4	42.6	0.86	0.001006038
Ccl3	4.3	10.0	10.1	46.7	47.2	96.9	0.85	0.00024275
Kdm5d	2.1	1634.4	0.0	1866.2	1637.7	1804.3	0.84	4.07334E-09
Htra1	339.9	348.9	379.8	841.8	630.4	737.3	0.84	4.74313E-07
Nfkbiz	1144.4	1005.8	1190.9	2685.7	1600.7	2433.8	0.84	8.40151E-07
Depp1	385.7	288.8	424.2	986.2	789.6	523.5	0.84	1.21494E-05
Gm42427	0.0	6.4	0.0	24.4	101.6	48.0	0.84	0.000285454
Uty	5.3	1618.0	0.0	1799.3	1767.0	1756.3	0.81	4.71587E-09
Itga8	309.0	297.0	336.5	531.8	600.7	835.1	0.81	1.23421E-05
Bhlhe40	980.3	843.6	817.1	1624.2	1433.4	2091.4	0.80	1.20739E-06
Gli1	99.1	92.9	91.7	184.7	207.4	298.9	0.80	0.000174362
Gm12762	12.8	0.0	4.0	42.5	48.3	53.4	0.80	0.000723855
Sema3g	476.3	582.1	478.6	826.9	1136.6	1327.9	0.78	1.3596E-05
Rrad	198.2	136.7	158.2	313.2	290.6	395.8	0.78	7.529E-05
Angptl2	858.8	818.1	963.2	1390.6	1531.9	1854.1	0.77	8.92455E-07
Hmox1	304.7	242.3	348.6	666.6	391.2	799.8	0.77	0.000198083
Ccl11	58.6	36.4	55.4	116.8	105.8	162.1	0.77	0.000745756
Veph1	25.6	33.7	29.2	75.4	77.0	192.0	0.77	0.001448815
Sulf1	147.0	123.0	196.5	280.2	279.3	375.0	0.76	0.000174362
Sox9	564.7	424.5	657.9	813.1	844.0	1115.0	0.75	1.3596E-05
Apold1	894.0	1073.2	1026.7	1734.6	1753.7	2313.3	0.74	3.7668E-06
Creb5	127.9	87.5	133.0	222.9	188.9	306.2	0.74	0.000654377
Tnxb	529.6	370.8	515.9	980.9	1048.3	1834.2	0.74	0.001676233
Pgk1	671.3	635.9	522.9	1155.0	1164.3	1130.4	0.73	2.07304E-06
Rest	381.5	345.3	637.8	826.9	663.3	1044.4	0.73	0.000198083
Ppp1r15a	2022.4	1822.0	1821.7	3589.1	2854.4	3403.0	0.72	3.19522E-07
Nfib	2738.4	2664.7	3460.9	4737.7	4270.3	6487.1	0.72	2.6657E-05
Meox2	206.7	190.4	244.8	345.0	384.0	510.0	0.72	0.000292942
Hmcn2	71.4	53.8	71.5	199.6	97.5	195.6	0.72	0.002324394
Mamdc2	32.0	80.2	75.6	124.2	147.9	335.1	0.72	0.003366305
Ltbp4	13201.0	12568.5	12775.8	20368.9	20755.9	23842.7	0.71	1.94279E-09
Zfp14	306.9	291.5	324.4	576.4	630.4	487.3	0.71	6.04323E-05
Ar	218.4	197.7	202.5	373.7	345.0	480.1	0.71	0.000292942
Acta2	1119.9	1635.3	1404.5	3055.1	2699.3	4504.4	0.71	0.002511708
Ces2g	69.3	92.9	112.8	187.9	179.7	260.9	0.71	0.00323306
Zfp36l1	2856.7	2806.9	3672.5	4696.3	4793.9	6120.3	0.70	7.27673E-06
Crb1	90.6	105.7	123.9	308.9	195.1	203.8	0.70	0.001917875
Foxc2	108.7	48.3	86.6	135.9	156.1	191.1	0.70	0.003104732

Zfpm2	73.5	63.8	50.4	135.9	129.4	182.1	0.70	0.003491841
Eln	4633.0	4363.8	6867.5	26038.5	10207.0	25806.4	0.70	0.003501754
Fbln5	384.7	420.0	626.7	1421.4	944.6	1814.3	0.70	0.003810752
Lama3	613.8	598.5	612.6	985.1	986.7	1269.9	0.69	3.21725E-05
Col4a4	237.6	113.9	215.6	284.5	290.6	298.0	0.69	0.000389113
Col4a5	350.6	426.4	444.3	727.2	623.2	1103.2	0.69	0.000692762
Procr	189.7	205.0	254.9	494.7	325.5	446.5	0.69	0.000875141
Grip2	43.7	61.0	53.4	97.7	138.6	160.3	0.69	0.003908353
Map3k20	900.4	900.1	982.4	1613.5	1437.5	1649.4	0.68	1.78723E-06
Phlda1	1252.0	1191.6	1295.7	2273.8	1837.9	2150.3	0.68	1.92487E-06
Ldb2	468.8	366.2	573.3	680.4	730.0	773.5	0.68	4.66404E-05
Jag1	943.0	958.4	1168.8	1668.7	1436.4	2394.0	0.68	0.000420558
Hspb1	181.1	198.6	232.7	458.6	353.2	358.7	0.68	0.000680164
Ddit4l	109.8	124.8	125.9	209.1	248.5	279.9	0.68	0.00145884
Kcnk5	132.1	122.1	142.1	227.2	230.0	331.5	0.68	0.00183858
Rnd1	116.1	84.7	88.7	212.3	162.2	219.2	0.68	0.003147664
Pisd-ps1	2162.0	1577.9	1856.9	6774.8	5507.5	3023.5	0.68	0.005984067
Lgr6	29.8	57.4	45.3	93.4	110.9	149.5	0.68	0.006208196
Abcb1a	267.5	239.6	255.9	419.3	429.2	504.5	0.67	0.000296241
Col5a3	413.4	430.0	622.7	677.3	849.1	1148.5	0.67	0.001371466
1110002E22R	86.3	49.2	58.4	132.7	173.5	119.6	0.67	0.006719051
Podn	22.4	21.0	18.1	71.1	48.3	80.6	0.67	0.00821151
Plk2	3384.2	3178.6	3638.3	5437.2	5021.9	5593.1	0.66	3.20973E-08
Hes1	834.3	845.4	1118.4	1538.2	1361.5	1690.2	0.66	0.000111479
Postn	2801.3	2683.0	3724.9	4445.7	4367.8	6106.7	0.66	0.000158685
Gm43305	47.9	1465.8	28.2	1118.9	1562.7	962.8	0.66	0.001035226
Vtn	527.4	753.4	810.1	1451.1	997.0	1569.7	0.66	0.001058978
Myof	458.2	637.7	611.6	974.5	881.0	1524.4	0.66	0.001293857
Dchs2	35.2	43.7	41.3	105.1	73.9	139.5	0.66	0.009552336
Cavin2	700.1	742.5	867.5	1370.4	1095.5	1500.0	0.65	0.000174938
Mrc2	358.0	354.4	440.3	561.6	593.5	995.4	0.65	0.001763145
Ltbp1	289.8	329.8	366.7	534.0	498.0	840.6	0.65	0.002469916
Ntrk3	120.4	116.6	121.9	204.9	201.2	326.1	0.65	0.004643978
Npas4	111.9	104.8	67.5	298.3	154.0	202.9	0.65	0.00692976
Fbln2	1976.6	2122.7	2625.7	5125.1	3508.4	6025.2	0.65	0.00821151
Antxr2	351.6	373.5	438.3	626.3	633.5	731.0	0.64	0.000292942
Pltp	635.1	532.9	693.2	800.4	1009.3	1185.7	0.64	0.00052514
Enpp1	297.3	225.0	298.2	408.7	403.5	533.5	0.64	0.001172904
Megf6	343.1	274.2	369.8	508.5	441.5	770.8	0.64	0.002817902
Lox	141.7	165.8	254.9	471.3	512.4	958.3	0.64	0.00923998
Smoc2	21.3	31.9	80.6	107.2	116.0	163.0	0.64	0.01061761
Inpp4b	1208.3	1034.0	1112.3	1736.7	1605.8	1879.5	0.63	1.84917E-05
Gja1	1111.4	974.8	1090.2	1485.1	1794.8	1698.3	0.63	1.84917E-05
Cxcl12	1886.0	2150.9	2139.0	3443.6	3020.7	4211.8	0.63	8.99497E-05
Lamb2	2232.3	2132.7	2363.7	3375.7	3160.4	4418.4	0.63	9.24474E-05
Ccn2	869.5	799.9	1005.5	1553.0	1148.9	1587.8	0.63	0.000357736
Fosl2	1094.3	943.8	1096.2	1685.7	1342.0	2123.1	0.63	0.000566063
Cebpd	274.9	350.7	341.6	639.0	530.8	587.8	0.63	0.000781468
Ier5l	263.2	266.0	361.7	518.0	441.5	564.3	0.63	0.00145884
Ackr3	297.3	237.8	332.5	615.7	361.4	508.1	0.63	0.003198872
Zfp566	171.6	156.7	124.9	291.9	312.1	259.1	0.63	0.003491841
Icam1	233.4	237.8	303.3	480.9	345.0	603.2	0.63	0.003607153
Mrc1	134.3	131.2	153.1	295.1	206.4	283.5	0.63	0.004643978
Fn1	1438.5	1301.9	1328.0	6667.5	2417.0	4389.4	0.63	0.01259157
Clec3b	37.3	30.1	73.6	92.4	90.4	179.3	0.63	0.01327041
Ogn	71.4	69.2	110.8	123.1	176.6	239.1	0.63	0.01472604
Trpv4	47.9	61.0	81.6	164.5	98.6	183.0	0.63	0.01873788
Ddx3y	1.1	4123.3	0.0	4057.2	4073.1	3990.8	0.62	1.20012E-06
Ptprb	6356.0	5979.1	5999.0	9730.1	8147.3	12556.7	0.62	0.00024275
Ccdc80	1597.2	1843.9	2138.0	2919.2	2813.3	3554.3	0.62	0.000243823
Mapk4	247.2	223.2	207.6	405.5	413.8	369.6	0.62	0.001597271
Arhgap6	284.5	285.2	219.6	559.4	427.1	467.4	0.62	0.002174287
Cldn2	488.0	362.6	390.9	529.7	703.3	859.6	0.62	0.002752785
Cilp	364.4	313.4	514.9	576.4	554.4	819.7	0.62	0.003010062
Myh11	1479.0	1728.2	1554.7	3503.1	3089.5	6277.0	0.62	0.0187242
Heyl	1604.7	1357.4	1730.0	2106.1	2188.0	2615.9	0.61	5.68959E-05
Eif2s3y	4.3	2481.6	0.0	2289.7	1915.9	2268.1	0.61	0.000123464
Adamts12	788.5	741.6	768.8	1099.8	1110.9	1713.7	0.61	0.001763145
Ripor2	193.9	223.2	232.7	409.8	337.8	419.4	0.61	0.00277992
Slit3	609.5	596.7	815.1	1121.0	824.5	1519.0	0.61	0.003810752
Ngf	70.3	61.0	96.7	133.8	138.6	172.1	0.61	0.01295023
Efhd1	94.8	60.1	87.7	145.4	128.3	181.2	0.61	0.0161273
Dcn	644.7	667.8	1057.9	1408.7	1766.0	2399.4	0.61	0.0179323
Hsd11b2	42.6	22.8	54.4	72.2	81.1	124.1	0.61	0.02756251
Xirp1	6.4	17.3	5.0	35.0	28.7	86.0	0.61	0.3532349
Epas1	9695.4	8734.9	10276.0	12995.4	13715.4	15479.7	0.60	2.4721E-06

Klf6	1916.9	1581.5	2212.6	2746.2	2490.9	2719.1	0.60	1.70628E-05
Adgrl4	1497.1	1195.3	1444.8	1880.0	1908.7	2159.4	0.60	2.62498E-05
Spry1	1179.6	1171.6	1559.7	1855.6	1999.1	2100.5	0.60	0.000216499
Hapln4	732.0	662.3	582.4	1093.4	1168.4	1009.0	0.60	0.000410568
Akap12	1721.9	1790.2	2265.0	2847.1	2675.7	3662.0	0.60	0.000694757
Prrx1	864.2	719.7	857.4	1077.5	1180.8	1506.3	0.60	0.000781468
Muc1	434.7	614.9	681.1	1003.2	816.3	1343.3	0.60	0.005471306
Tmem100	93.8	113.0	109.8	205.9	163.3	261.8	0.60	0.01194656
Erbb4	34.1	37.4	60.5	72.2	89.3	202.0	0.60	0.02160751
Cndp2	1999.0	2124.5	1948.6	3327.9	3309.2	3252.6	0.59	1.04704E-06
Ebf1	1269.1	1181.6	1478.1	1804.6	1891.3	2196.5	0.59	0.000176353
Gm13889	813.0	601.3	892.7	1105.1	951.8	1124.1	0.59	0.000299009
Gsn	2005.4	1949.6	2352.6	2812.0	3047.4	4036.1	0.59	0.000713513
Adamts4	464.6	295.2	345.6	540.3	498.0	708.3	0.59	0.003998878
Hoxa3	98.0	104.8	124.9	195.3	173.5	241.8	0.59	0.01451644
Sik1	753.3	632.3	611.6	1403.4	1023.7	1974.6	0.59	0.02517549
Adamts1	1147.6	1124.2	1488.2	2537.1	2110.0	3980.9	0.59	0.02688844
Krt23	46.9	51.0	87.7	118.9	115.0	157.6	0.59	0.02994081
Gm2762	55.4	79.3	31.2	107.2	137.6	136.8	0.59	0.03550936
Avpr1a	30.9	41.9	24.2	78.6	78.0	80.6	0.59	0.04328307
Jund	8350.7	7324.6	8981.3	13182.2	11629.0	10671.8	0.58	1.84917E-05
Pcdh18	1123.1	931.1	1069.0	1530.7	1411.8	1650.3	0.58	0.0001015
Aoc3	1648.4	1672.6	1706.8	2377.9	2367.7	3578.7	0.58	0.001763145
Cdh11	532.8	444.6	621.7	704.9	728.0	1027.1	0.58	0.003107776
Klf4	1028.2	1109.6	1273.5	1829.0	1469.3	2300.7	0.58	0.003185355
Pdgfc	142.8	197.7	212.6	262.2	348.1	437.5	0.58	0.01396085
Tnfrsf19	214.2	215.9	331.5	355.6	376.8	634.0	0.58	0.01449732
3930402G23I	68.2	108.4	42.3	196.4	143.7	143.1	0.58	0.02294965
Nrk	132.1	196.8	222.7	397.0	418.9	773.5	0.58	0.02517549
Bc1	52.2	68.3	154.2	153.9	274.1	183.9	0.58	0.02775675
Col4a2	43215.2	43650.9	44430.1	63532.2	63194.8	77263.4	0.57	1.9088E-05
Cavin1	3943.6	3779.8	4312.3	5430.8	5875.1	6560.5	0.57	3.81615E-05
Synm	253.6	134.8	232.7	281.3	271.1	338.8	0.57	0.00821151
Snai1	190.7	123.0	199.5	227.2	290.6	272.6	0.57	0.008853909
C1ra	151.3	183.1	167.3	230.4	341.9	355.1	0.57	0.01396085
Fabp4	312.2	260.6	480.6	494.7	445.6	769.9	0.57	0.01583764
Ier3	153.4	172.2	299.2	756.9	388.1	779.0	0.57	0.02688844
Smtnl2	71.4	94.7	115.9	195.3	128.3	257.2	0.57	0.0303123
Tcf21	77.8	95.7	107.8	126.3	195.1	250.0	0.57	0.04492379
Cxcl10	11.7	22.8	18.1	92.4	50.3	36.2	0.57	0.04499574
Acan	0.0	6.4	2.0	27.6	18.5	45.3	0.57	0.2337901
Col15a1	13854.2	13301.0	13727.9	19257.4	19191.1	23135.3	0.56	3.81615E-05
Ednra	1109.2	883.7	1066.0	1417.2	1323.5	1634.9	0.56	0.00052514
Mylk	2162.0	2286.7	2327.4	3128.4	3227.1	4703.7	0.56	0.002192886
Nr2f2	1085.8	1024.0	1377.3	1591.3	1548.3	2048.0	0.56	0.002192886
Nr4a2	607.4	695.1	586.4	1021.2	900.5	1347.8	0.56	0.003521661
Zfp503	485.9	423.6	720.4	744.1	721.8	929.3	0.56	0.00692976
Cd14	100.2	113.0	148.1	204.9	211.5	226.4	0.56	0.01901951
Ppp1r1b	110.8	134.8	198.5	233.5	239.2	344.2	0.56	0.02527724
Adamtsl3	34.1	20.0	48.4	74.3	56.5	114.1	0.56	0.04807213
Hk2	44.8	40.1	36.3	94.5	81.1	87.0	0.56	0.05030942
Cd93	8223.9	7262.7	7438.8	11100.5	10276.8	12529.6	0.55	5.98708E-05
Lamb1	8376.2	7965.1	8499.7	11752.3	11295.3	14060.3	0.55	8.42983E-05
Dnajb1	1867.9	1634.4	1593.9	2818.4	2257.8	2684.7	0.55	0.00052514
Vgf	2561.6	2794.1	1998.0	4527.5	4040.3	3903.0	0.55	0.00052514
Itga5	1591.9	1495.0	1659.4	2332.2	2063.8	2778.0	0.55	0.000779722
Rasd1	2802.4	1997.9	2722.4	3925.6	3680.9	2705.5	0.55	0.001595752
Crim1	1113.5	1117.8	1318.9	1650.7	1651.0	2067.9	0.55	0.001597271
Mrvi1	505.1	483.8	529.0	729.3	750.6	853.2	0.55	0.00183858
Stab1	1687.8	1604.3	1757.2	2698.4	2046.3	3057.9	0.55	0.002212904
Timp2	2571.2	2307.6	3324.9	3748.3	3247.6	4904.8	0.55	0.00390914
Ddr2	769.3	874.6	902.8	1261.1	1173.6	1759.9	0.55	0.005286117
Ror1	541.3	387.2	551.1	626.3	627.3	872.3	0.55	0.006315382
Fbxl7	238.7	244.2	313.3	428.9	389.1	448.4	0.55	0.009143741
Egr2	46.9	77.4	122.9	537.1	352.2	1143.1	0.55	0.009242254
Atf3	132.1	55.6	212.6	584.9	445.6	1570.6	0.55	0.01391177
Gli2	99.1	138.5	169.3	233.5	233.1	273.5	0.55	0.02600997
Kcne4	85.2	106.6	123.9	169.8	173.5	251.8	0.55	0.03209814
Fbn2	954.7	917.4	1082.1	1590.2	1565.8	2562.4	0.55	0.04589599
Srpx	125.7	70.1	109.8	131.6	164.3	240.0	0.55	0.0488048
Ctnna3	70.3	75.6	84.6	147.6	106.8	211.0	0.55	0.05261984
Zfp385b	42.6	20.0	37.3	79.6	54.4	87.9	0.55	0.06013759
Gdf15	9.6	30.1	5.0	36.1	55.4	56.2	0.55	0.1264488
Nid1	10227.1	9805.4	11446.8	14066.5	14054.2	18120.9	0.54	0.000566063
Mecom	489.1	547.5	605.5	773.9	818.3	1030.8	0.54	0.005017428
Pcolce	726.7	744.3	910.8	1047.7	1142.8	1522.6	0.54	0.006053721

Ltbp2	321.8	180.4	295.2	363.0	353.2	346.0	0.54	0.00824175
Cnnm1	260.0	186.8	248.9	334.4	340.9	344.2	0.54	0.009193955
Thsd7a	344.2	397.2	412.1	638.0	508.2	794.4	0.54	0.01209201
Palld	432.6	378.1	416.1	582.8	531.9	862.3	0.54	0.01327041
Lrrc55	372.9	369.9	416.1	601.9	471.3	861.4	0.54	0.0187242
Inf2	396.4	399.0	404.0	665.6	472.3	900.3	0.54	0.01947763
Flnc	148.1	192.2	203.5	330.1	212.5	490.9	0.54	0.04295524
Selp	24.5	25.5	54.4	78.6	51.3	163.0	0.54	0.05941783
Emilin2	40.5	42.8	41.3	76.4	78.0	118.7	0.54	0.07528257
Fat2	6.4	34.6	4.0	86.0	20.5	72.5	0.54	0.2820858
Fstl1	6197.2	6004.6	7631.2	8977.5	8716.1	10641.9	0.53	0.000654377
Ppp1r16b	852.4	645.0	694.2	987.2	1056.5	1098.7	0.53	0.00194577
Entpd1	1015.5	1202.6	1267.5	1870.4	1558.6	2153.0	0.53	0.004643978
Msrb3	805.6	745.2	1006.5	1166.6	1099.7	1418.4	0.53	0.004690872
Vim	4492.3	4953.2	5826.7	6759.9	7142.1	9611.2	0.53	0.005126846
Eef1a2	920.6	757.1	1372.3	1525.4	1278.3	1313.4	0.53	0.007330061
Kcnab1	211.0	164.9	167.3	318.5	260.8	292.6	0.53	0.02250729
Fxyd1	160.9	147.6	234.8	279.2	270.0	303.4	0.53	0.02367061
Reln	290.9	377.2	309.3	467.1	480.5	798.9	0.53	0.02577712
Gm42031	30.9	265.1	13.1	187.9	261.8	216.5	0.53	0.04849873
Lamb3	72.5	68.3	82.6	135.9	104.7	183.9	0.53	0.05427952
Osr2	57.5	87.5	168.3	174.1	173.5	311.6	0.53	0.05864978
Myocd	32.0	37.4	32.2	71.1	50.3	143.1	0.53	0.06925594
Fbn1	2352.7	2543.6	2861.5	4851.2	4286.7	8351.2	0.53	0.06961068
Dusp2	13.9	25.5	42.3	79.6	50.3	96.0	0.53	0.1101843
Ttpa	30.9	47.4	46.3	87.0	89.3	90.6	0.53	0.116853
Gm46378	12.8	15.5	19.1	31.8	56.5	58.0	0.53	0.1629149
Cav1	3325.6	3344.4	3501.2	4804.5	4785.7	5412.0	0.52	8.34105E-05
Col4a1	80941.8	78359.1	80662.7	109892.9	110534.4	131871.5	0.52	0.0001015
Lama4	6720.4	6440.0	6461.4	9016.7	9007.7	11015.1	0.52	0.000389113
Arhgap31	2637.2	2253.0	2310.3	3470.2	3082.3	3881.2	0.52	0.000905904
Pdgfrb	4213.2	4196.2	4647.8	5862.9	5855.6	7578.6	0.52	0.001422473
Rgs7bp	655.3	508.4	666.0	780.2	840.9	868.6	0.52	0.00194577
Gas6	2004.3	2038.9	2671.0	2880.0	3115.2	3801.5	0.52	0.004103919
Gpc6	643.6	594.0	650.9	952.2	793.7	1097.8	0.52	0.006730881
Cygb	911.0	834.5	1128.5	1138.0	1448.8	1534.4	0.52	0.007093967
Gata2	267.5	287.9	348.6	456.5	442.5	519.9	0.52	0.01327041
Rsad1	411.3	288.8	315.4	449.0	469.2	616.8	0.52	0.01831861
Eps8l1	377.2	470.1	398.0	687.9	963.1	516.3	0.52	0.02753048
Pcsk5	219.5	190.4	182.4	319.5	264.9	394.0	0.52	0.02857151
Nfkbia	1466.2	1541.5	1816.6	4042.3	2509.4	2585.1	0.52	0.05961024
Vcam1	66.1	82.9	141.1	155.0	168.4	215.6	0.52	0.06458512
Svep1	237.6	247.8	380.9	528.6	601.7	1129.5	0.52	0.06580215
Pi15	595.6	544.8	694.2	869.4	1616.1	2408.5	0.52	0.06580215
Mfap4	412.4	480.1	664.0	874.7	1202.3	1896.7	0.52	0.0672658
C2cd4b	2914.3	2867.9	3032.7	7254.6	4836.0	4249.9	0.52	0.06925594
Hmcn1	403.8	318.9	419.1	867.3	577.0	1453.8	0.52	0.06996552
Itgb4	361.2	492.0	536.0	1025.4	660.2	1279.9	0.52	0.07357338
Xdh	92.7	95.7	42.3	147.6	150.9	170.3	0.52	0.07624166
Col5a1	2213.1	2281.2	2886.6	4409.6	4318.5	8065.0	0.52	0.07727106
Wfdc1	45.8	71.1	79.6	101.9	129.4	169.4	0.52	0.09382053
Pax1	2.1	0.0	7.1	17.0	27.7	39.9	0.52	0.4155967
Cald1	4040.5	4090.5	4594.4	5827.9	5569.1	7228.1	0.51	0.00194577
Epha2	696.9	718.8	816.1	1072.2	1040.1	1206.5	0.51	0.003283841
Apln	742.7	733.4	902.8	1180.4	1018.5	1285.3	0.51	0.005377833
Atp1a2	1010.1	856.4	1166.7	1368.3	1178.7	1772.6	0.51	0.01005807
Cxcr4	430.5	300.6	437.3	468.1	599.6	521.7	0.51	0.01036034
Pisd-ps2	290.9	225.0	292.2	448.0	490.8	302.5	0.51	0.03149681
Pvr	318.6	289.7	266.0	426.7	389.1	613.2	0.51	0.03224859
Map3k7cl	131.1	165.8	148.1	294.0	169.4	365.0	0.51	0.05918102
Bmp2	87.4	94.7	88.7	156.0	147.9	173.9	0.51	0.06805127
Grhl2	106.6	119.3	153.1	211.2	146.8	361.4	0.51	0.07298313
Myl9	898.3	960.2	1127.5	1537.1	1577.1	2504.5	0.51	0.08350627
Susd5	61.8	41.0	91.7	117.8	79.1	183.0	0.51	0.1053109
Lrg1	22.4	21.9	23.2	40.3	64.7	63.4	0.51	0.1154432
Bmx	49.0	23.7	42.3	93.4	55.4	87.0	0.51	0.116853
1700016P03R	25.6	25.5	12.1	56.3	45.2	67.9	0.51	0.1410729
Fbxl22	18.1	34.6	22.2	48.8	49.3	98.7	0.51	0.1448335
Tnf	6.4	10.9	7.1	26.5	34.9	36.2	0.51	0.2646427
Ccl2	8.5	4.6	6.0	42.5	16.4	38.9	0.51	0.6294316
Sparcl1	11033.7	10581.6	12172.2	14947.6	15175.5	16918.0	0.50	0.000167433
Lamc1	16866.5	15417.3	16095.7	21861.4	20968.4	26541.9	0.50	0.000713513
Sat1	939.8	965.7	1007.6	1511.6	1400.5	1398.5	0.50	0.001481171
Spry4	1528.0	1329.2	1294.7	1893.8	1905.7	2223.7	0.50	0.002638719
Ehd2	2656.4	2756.8	2954.1	3839.6	3730.2	4896.6	0.50	0.002778469
Pld1	854.6	918.3	970.3	1270.7	1242.4	1682.9	0.50	0.009888083

Kirrel2	323.9	331.6	335.5	528.6	480.5	517.2	0.50	0.01080649
Lrrc32	664.9	706.0	716.4	1070.0	866.6	1354.1	0.50	0.01483869
Peak1	1338.3	1156.1	1376.3	1674.1	1543.2	2443.8	0.50	0.01636862
Trim16	263.2	254.2	294.2	365.2	433.3	432.1	0.50	0.02147165
Tpm2	411.3	492.0	413.1	613.6	642.7	943.8	0.50	0.02451139
Adamts16	339.9	431.8	430.2	636.9	509.3	849.6	0.50	0.0274417
Plscr2	248.3	165.8	195.5	307.8	317.3	276.3	0.50	0.03112825
Arhgef26	122.5	152.1	209.6	263.3	231.0	320.6	0.50	0.06170682
Serpinf1	187.5	139.4	326.4	278.1	267.0	498.2	0.50	0.07357338
Col6a3	1964.9	1891.3	2310.3	3223.9	3224.0	5487.2	0.50	0.08915548
Cpz	8.5	10.0	42.3	40.3	61.6	103.3	0.50	0.1144469
Adam33	18.1	21.0	36.3	54.1	43.1	103.3	0.50	0.1219702
Igfbp6	3.2	10.0	4.0	30.8	24.6	29.0	0.50	0.239798
Dpp6	2214.2	2156.4	1946.6	1432.0	1348.1	1539.8	-0.50	0.001006038
Folr1	643.6	506.5	694.2	353.5	394.3	321.5	-0.50	0.01295023
Lmo3	192.9	168.5	201.5	113.6	105.8	99.6	-0.50	0.04987158
Ccl27a	292.0	288.8	324.4	115.7	163.3	235.5	-0.50	0.05986343
Gm47283	5070.9	2645.6	4805.0	453.3	1015.5	1727.3	-0.50	0.06287562
Gm4070	89.5	137.6	74.6	35.0	78.0	42.6	-0.50	0.1137793
Gm17249	84.2	81.1	118.9	31.8	49.3	51.6	-0.50	0.1622385
Tspan8	4021.4	3369.0	3405.5	2359.8	2490.9	1973.7	-0.51	0.001614256
Lrp8os2	72.5	52.8	62.5	25.5	20.5	30.8	-0.51	0.09382053
Gm30238	76.7	61.9	50.4	27.6	29.8	10.0	-0.51	0.1269841
4930405N21I	59.7	51.0	60.5	22.3	18.5	27.2	-0.52	0.08816894
Ly6h	575.4	505.6	538.0	376.8	328.6	265.4	-0.53	0.0113856
Igf2bp1	497.6	399.9	401.0	262.2	205.4	289.8	-0.53	0.0174013
Gm11824	92.7	62.9	168.3	52.0	35.9	44.4	-0.53	0.07624166
Fam92b	688.3	770.7	718.4	508.5	484.6	464.7	-0.54	0.002039488
Gm42777	114.0	177.6	123.9	97.7	68.8	77.0	-0.54	0.04466603
9330162G02I	111.9	106.6	87.7	47.8	53.4	54.3	-0.54	0.0535933
Gm14966	93.8	109.3	65.5	44.6	34.9	51.6	-0.54	0.06707427
4930578M01	512.5	526.6	492.7	340.8	297.8	335.1	-0.55	0.004050613
Sv2b	859.9	952.9	983.4	594.5	686.9	471.0	-0.55	0.004673339
Cpb2	377.2	307.0	296.2	180.5	217.7	159.4	-0.55	0.01584984
4933433G15I	40.5	59.2	30.2	13.8	6.2	16.3	-0.55	0.2698135
A530020G20	556.2	610.4	517.9	295.1	303.9	444.7	-0.56	0.008340702
2900052L18R	409.2	547.5	601.5	310.0	362.4	271.7	-0.56	0.01051363
Gm49748	392.1	373.5	292.2	203.8	240.3	191.1	-0.56	0.01097299
Gm45623	310.1	273.3	284.1	132.7	178.7	172.1	-0.57	0.01110027
Prss45	9.6	50.1	17.1	1.1	0.0	0.0	-0.57	0.01991955
Gip	56.5	66.5	41.3	19.1	22.6	7.2	-0.57	0.6325935
Gm4673	283.4	361.7	312.3	201.7	206.4	192.9	-0.58	0.005342584
Gm45104	75.7	30.1	82.6	14.9	17.5	25.4	-0.58	0.03431048
Hectd2os	127.9	82.9	84.6	43.5	49.3	27.2	-0.58	0.04637112
Gm21781	624.4	685.1	626.7	388.5	312.1	475.5	-0.59	0.003451351
Gm43952	2138.5	9453.7	2867.5	2786.5	1869.7	3398.5	-0.59	0.0271573
Nnat	14492.4	14313.1	14514.8	9838.4	8714.1	8825.9	-0.60	3.47507E-07
Ovol2	258.9	339.8	361.7	190.0	197.1	159.4	-0.60	0.006063011
Gm26788	267.5	340.7	222.7	130.6	215.6	103.3	-0.60	0.01317082
5730405O15I	152.4	157.6	167.3	60.5	90.4	87.0	-0.60	0.01901951
Sbsn	172.6	154.9	162.2	76.4	42.1	101.4	-0.60	0.02994081
Pyy	180765.0	158508.3	152090.1	100678.7	103050.3	96851.6	-0.61	4.74313E-07
Atp5l	921.7	961.1	866.5	613.6	586.3	515.4	-0.61	0.000182141
Pde6c	300.5	348.9	371.8	214.4	191.0	178.4	-0.61	0.003634089
Gm21887	3964.9	1864.9	3696.7	397.0	556.5	1126.8	-0.61	0.0108036
Gm16008	418.8	416.3	367.8	213.4	197.1	271.7	-0.62	0.002512729
Nlrc5	151.3	208.6	122.9	49.9	116.0	40.8	-0.62	0.01945662
Gm12758	116.1	121.2	139.0	64.8	61.6	39.9	-0.63	0.01477323
D7Ertd128e	38.4	32.8	60.5	1.1	2.1	9.1	-0.65	0.005671138
Tmem147os	328.2	351.7	308.3	176.2	166.3	201.1	-0.67	0.000692762
Ffar2	3301.1	3307.0	3167.7	2001.0	1829.7	1984.5	-0.69	1.09538E-08
Sst	108269.8	104030.9	89894.9	59454.8	66136.4	52001.3	-0.70	3.60997E-07
Kcnk9	907.8	1545.1	990.4	811.0	529.8	800.7	-0.73	4.93278E-05
Ccl28	1999.0	1208.9	1951.6	363.0	338.8	709.2	-0.75	0.001443304
Slc4a1	213.1	185.8	130.0	47.8	84.2	74.3	-0.76	0.00154112
Gm43652	155.6	218.6	95.7	71.1	72.9	33.5	-0.77	0.001481171
Tmem267	626.5	408.1	873.5	180.5	284.4	241.8	-0.80	2.87816E-05
Prr29	251.5	351.7	300.3	158.2	141.7	143.1	-0.82	1.52824E-05
Chst8	370.8	322.5	248.9	108.3	158.1	136.8	-0.82	5.51049E-05
Gpr179	1472.6	1699.1	1352.1	898.1	786.5	780.8	-0.87	1.63002E-10
Tsix	119.3	15.5	51.4	2.1	6.2	1.8	-0.88	4.00363E-05
Haus5	206.7	209.5	162.2	76.4	59.6	72.5	-0.95	3.71994E-06
Ghrl	5236.1	4602.5	4712.3	2055.1	2499.1	1962.8	-0.96	1.62452E-13
5830444B04R	246.1	303.4	289.2	56.3	102.7	119.6	-0.99	1.08027E-06
AI480526	3810.4	3876.4	3693.7	1653.9	1728.0	1948.3	-1.00	3.65181E-17
Gm45250	262.1	316.1	226.7	98.7	96.5	120.5	-1.00	8.22842E-08

Trpc7	292.0	198.6	308.3	104.0	61.6	71.6	-1.01	3.12559E-07
Gm26917	17756.2	18104.8	16862.4	7063.5	7151.4	8871.1	-1.09	3.25385E-19
Gm16299	100.2	118.4	70.5	11.7	12.3	20.8	-1.13	4.84436E-08
Ffar1	11396.0	10522.3	10353.6	2883.1	2701.4	2664.8	-1.78	1.04836E-68
Ffar3	1404.4	1321.0	1270.5	261.1	290.6	235.5	-1.95	5.81989E-48
Xist	70246.9	37.4	55319.7	19.1	10.3	27.2	-3.30	1.60412E-77

ESM Table 3: RNAseq results: top 5000 genes with the highest mean expression across all samples

Gene	WT1	WT2	WT3	KO1	KO2	KO3	Log2FC	padj
	♀	♂	♀	♂	♂	♂	(KOvsWT)	
Ins2	3391690.6	3357782.0	3292605.3	3120726.8	3751943.3	2894697.4	-0.04	0.9662
Ins1	1927217.7	1951862.8	1897016.6	1781368.4	2121077.3	1696487.1	-0.05	0.9986
Gcg	975286.6	1213072.6	1044685.2	1441111.4	1394882.7	1082514.1	0.14	0.7818
Chga	504568.4	425995.3	471924.7	451718.8	500795.0	434686.9	0.09	0.8809
Iapp	410859.3	379475.6	377348.7	357289.4	418842.4	315147.5	-0.06	0.9334
Gm42418	348922.5	291027.8	499433.9	174855.0	232518.1	368217.7	-0.21	0.7795
mt-Co1	293007.1	295808.9	298992.3	271335.3	301938.1	244542.5	-0.11	0.8314
Pyy	180765.0	158508.3	152090.1	100678.7	103050.3	96851.6	-0.61	0.0000
Scg2	173164.5	158014.5	154460.9	146777.2	165531.5	135538.1	-0.08	0.9155
Pcsk2	161785.6	165428.5	158048.8	165724.7	175937.7	145676.4	-0.02	1.0000
Ctrb1	157719.4	285325.7	313510.2	339735.7	225661.4	361549.4	0.13	0.8758
Ppy	157432.8	105873.9	107046.4	87877.6	91459.3	73939.2	-0.34	0.1744
mt-Cytb	156218.1	164865.5	168443.7	164449.7	180171.0	158105.4	0.02	1.0000
Cpe	151743.9	151776.8	144918.3	143121.3	153447.6	130985.7	-0.08	0.8698
mt-Nd5	134887.0	143879.1	138294.7	160511.4	170077.0	136083.4	0.11	0.8316
Gnas	122076.0	135260.8	131701.3	136263.7	131805.7	116654.5	-0.06	0.9237
Peg3	119091.4	111998.7	105375.9	125897.7	110811.6	129690.4	0.11	0.8184
Pnliprp1	112819.6	212378.1	249112.4	309422.3	198048.9	336983.9	0.23	0.7470
Sst	108269.8	104030.9	89894.9	59454.8	66136.4	52001.3	-0.70	0.0000
Acly	105055.0	104272.3	100422.8	113369.4	117954.7	111110.3	0.12	0.7546
Chgb	94520.0	84911.2	89025.4	82049.7	89900.7	79001.5	-0.03	1.0000
mt-Nd1	92236.5	101664.0	102357.3	90448.6	92401.9	88937.9	-0.15	0.6698
Eef1a1	90033.0	91863.2	94160.9	87809.6	90841.2	76366.7	-0.10	0.8830
Hspa5	86284.4	98275.9	74816.8	76070.1	78292.2	74485.4	-0.30	0.0543
Atp2a2	84381.4	79947.9	72836.0	80715.4	82499.8	79332.2	0.02	1.0000
Col4a1	80941.8	78359.1	80662.7	109892.9	110534.4	131871.5	0.52	0.0001
Eef2	73725.9	70358.5	76508.5	71231.6	77828.1	71735.4	0.05	0.9576
Xist	70246.9	37.4	55319.7	19.1	10.3	27.2	-3.30	0.0000
Cel	70120.1	136672.9	139413.1	178359.1	110450.2	184264.2	0.19	0.7821
Abcc8	68074.3	67953.4	60609.3	61409.1	59818.8	63067.2	-0.13	0.7271
mt-Nd4	66072.1	70309.3	72627.4	65265.7	73016.7	63997.4	-0.05	0.9542
Malat1	61499.9	61639.9	49630.0	59410.2	40823.8	78112.1	0.01	1.0000
mt-Nd2	60338.5	64989.8	65691.4	57573.8	63282.0	54618.1	-0.13	0.7352
Meg3	59485.0	60986.7	58114.6	56078.0	47494.6	63931.3	-0.10	0.8631
Hsp90b1	59331.5	64323.8	53216.9	58081.2	60577.6	51647.2	-0.14	0.6881
Syt13	58387.5	56675.8	53763.0	52485.8	56635.9	48710.7	-0.10	0.8214
G6pc2	58290.5	45178.6	40262.8	44325.7	50655.0	38672.0	-0.04	0.9690
Maged1	55039.5	55556.1	53768.1	60717.0	61887.7	54302.9	0.08	0.8874
Pcsk1n	53721.4	54071.2	61148.4	52106.8	66374.6	49874.6	0.03	1.0000
Cplx2	52523.8	49684.6	52099.6	42635.7	47789.3	43700.8	-0.15	0.5937
Ccnd2	51452.9	45932.1	43823.5	50103.7	54064.9	50645.4	0.15	0.6872
Atp2a3	51194.0	49576.2	43756.0	43027.5	42878.3	42983.5	-0.18	0.4679
Hsp90ab1	50740.1	50612.9	54196.3	58285.0	56881.2	54783.9	0.15	0.6653
Ptprn2	49804.5	48499.3	44222.5	43381.0	44824.1	39604.9	-0.17	0.5618
Eif4g2	49203.5	49196.3	47625.0	49657.8	49623.1	49925.3	0.02	1.0000
Ddx5	48832.7	46281.9	47153.5	49591.0	50628.3	48113.8	0.08	0.8591
Scg3	46977.6	44131.0	41125.3	44541.2	48848.9	45467.1	0.06	0.9422
Hyou1	46053.8	54660.6	40407.9	49141.9	48435.2	46538.6	-0.13	0.6727
Pdia3	44021.8	50939.0	43727.8	47472.1	48501.9	41350.4	-0.12	0.7636
Aplp1	43854.5	44880.7	46622.5	40678.3	44680.3	36681.1	-0.13	0.7682
Cd164	43523.1	40535.1	40409.9	37931.0	40165.7	35383.1	-0.10	0.8366
Col4a2	43215.2	43650.9	44430.1	63532.2	63194.8	77263.4	0.57	0.0000
Ece1	42201.9	43714.6	44075.4	43040.2	44959.6	41935.5	-0.01	1.0000
Pcsk1	41834.3	41669.4	37056.8	42362.9	43964.7	39977.2	0.02	1.0000
P4hb	41818.3	45520.3	39630.1	44604.9	46094.1	41943.6	-0.02	1.0000
Ero1lb	41530.6	41335.0	36985.3	42244.0	46755.4	44699.9	0.11	0.8011
Ttr	41240.8	47013.4	39498.1	58374.2	61194.6	44563.1	0.23	0.4740
Psap	40437.3	37875.0	41243.2	38923.5	43171.0	40447.3	0.09	0.8518
Cela2a	40140.0	74453.5	75269.2	77319.5	52497.0	73317.8	0.02	1.0000
Nedd4	39859.8	38599.2	38761.6	41655.9	40198.5	43652.8	0.11	0.7896
Spock2	39729.8	34113.3	31197.9	27940.8	29150.6	28848.9	-0.23	0.3583
Ddx17	39625.4	39666.9	34803.9	33184.8	31653.9	34410.3	-0.23	0.2556
Gm23935	39351.5	36226.9	63755.9	22726.5	27084.8	57274.8	-0.12	0.9277
Atp1a1	39209.8	38006.1	35794.3	38369.4	39197.4	37074.2	0.01	1.0000
Nisch	39192.8	39629.6	35132.4	37888.5	35901.5	40335.0	-0.04	0.9780
Gnao1	39045.7	39602.3	36379.7	36916.2	34863.5	38070.6	-0.10	0.8339
Glp1r	38817.7	33590.4	35487.0	37697.5	39672.8	42203.6	0.21	0.3390
Lars2	38700.5	32042.6	58148.9	22318.9	27822.0	61128.8	-0.06	0.9877
Slc30a8	38159.2	39124.0	33652.3	35921.5	34454.8	34095.1	-0.14	0.6695
Actb	38062.2	34201.7	39845.7	44535.9	45271.7	43143.8	0.33	0.0101
Ptprf	37357.9	37181.7	35725.8	37655.0	36612.1	40049.7	0.04	0.9593

Calm1	37245.0	36498.4	37120.3	37578.6	37442.7	35716.5	0.01	1.0000
Ubb	37161.9	37110.6	37429.6	35891.8	39472.6	28410.5	-0.09	0.9160
Ctnnb1	37064.9	38165.6	42188.3	36983.0	37437.6	39542.4	-0.01	0.9986
Cpb1	36549.2	70366.7	70400.7	78960.6	50559.5	83365.6	0.11	0.9343
Prkar1a	36436.2	35691.2	33766.1	35697.5	37477.6	33914.9	0.00	1.0000
Srrm2	36351.0	35215.7	31613.0	34676.3	27952.4	36975.5	-0.07	0.9503
Pam	36272.1	36842.8	34879.5	37699.6	38028.0	35810.7	0.02	1.0000
Mlxipl	32732.4	31508.7	28598.4	27418.6	23806.4	28386.0	-0.22	0.4117
Dst	32506.5	30697.0	27299.6	33026.7	26625.8	34567.9	0.04	0.9925
Calr	31907.7	34965.1	29952.5	33368.5	34566.8	33106.0	-0.03	0.9820
Gfpt1	31578.4	30678.8	27715.8	29757.1	29807.7	31201.2	-0.01	0.9980
Ubr4	31412.2	32153.7	22339.5	36103.0	30404.3	41081.3	0.18	0.7043
Hnrnpa2b1	30968.9	30277.9	29788.3	30652.0	30530.6	32385.0	0.04	0.9780
Son	30512.9	29870.7	27322.8	29974.7	26738.8	32741.0	0.00	1.0000
Rian	30487.3	28859.4	26830.1	25617.1	24524.1	23806.4	-0.21	0.3663
Papss2	29848.0	29289.5	28782.8	27565.0	29734.8	28448.5	-0.03	0.9838
Hdlbp	29756.3	30513.9	25465.9	30662.6	28980.2	33268.1	0.04	0.9884
Hspg2	29715.8	29842.4	27273.4	45969.0	36552.5	59868.9	0.45	0.1629
Itm2b	29438.8	29327.7	30880.5	27852.7	30570.6	26083.6	-0.06	0.9253
App	29356.8	30867.3	31330.9	30886.6	31757.6	32313.5	0.04	1.0000
Dync1h1	29323.7	28826.7	25361.1	33005.4	27156.7	40075.9	0.19	0.6337
Sptbn1	29309.9	27426.4	27979.7	33743.2	31263.7	38227.3	0.29	0.1394
Enpp2	29270.4	27662.4	29854.8	25609.7	27428.8	23711.3	-0.11	0.8316
Gm13456	29235.3	28729.2	31024.6	29274.1	35334.8	24812.8	0.03	0.9783
Eif4g1	29127.7	29863.4	26243.7	30146.7	28150.6	30743.8	0.00	1.0000
Copa	28914.6	30827.3	26866.4	30561.8	30357.1	31504.6	0.01	1.0000
Prrc2b	28817.6	28724.6	26752.5	28859.1	26151.5	31360.6	0.01	1.0000
Plxnb2	28771.8	27009.2	26757.6	25396.3	25942.0	27050.0	-0.05	0.9649
Rimbp2	28759.0	27472.9	23399.4	24680.8	22302.2	24955.9	-0.17	0.6585
Slc2a2	28755.8	24449.2	25607.0	27726.4	32034.8	28132.4	0.22	0.3739
Cltc	28295.5	27123.0	25869.9	31233.7	30075.7	32901.3	0.20	0.4103
Dnmt3a	28220.9	26888.9	26326.3	26431.3	24851.6	28920.4	-0.01	1.0000
Canx	28127.1	28016.8	27703.7	28068.2	30305.7	27310.0	0.03	1.0000
mt-Rnr2	28087.7	26851.5	31906.2	22993.0	26754.2	25871.6	-0.10	0.8474
Prrc2a	27515.5	27464.7	24777.7	27914.3	25414.3	29822.6	0.02	0.9881
Cpa1	26992.3	51394.6	50643.6	62689.3	39768.3	76172.8	0.19	0.8599
Atp5b	26896.4	28185.3	26900.7	28658.4	29389.9	26518.3	0.01	1.0000
Nkx6-1	26857.0	22886.8	25804.4	23297.6	24744.8	20116.3	-0.04	0.9660
Tpt1-ps3	26827.2	28671.8	30696.1	27661.6	29896.0	24058.2	-0.07	0.9794
Atp5a1	26754.7	27739.8	27321.8	28561.8	29321.1	27953.1	0.05	0.9382
Hadh	26708.9	24680.6	24336.4	22342.3	26366.1	19350.9	-0.11	0.8948
Rap1gap2	26622.6	25280.9	23428.6	21314.7	21565.0	23226.7	-0.18	0.5657
Rpl4	26597.0	26866.1	27334.9	25623.5	27300.4	24226.7	-0.06	0.9377
Gng12	26055.7	27298.9	23041.7	28808.1	29726.6	28126.1	0.09	0.8473
Kcnk16	25551.7	25545.1	24152.1	21917.6	23644.1	19378.1	-0.21	0.3921
Surf4	25442.0	26788.7	23576.7	23328.4	24722.2	23128.9	-0.15	0.6189
Prss2	25403.6	52112.5	50951.9	52261.8	33997.9	71428.4	0.11	0.9466
Clps	24944.3	42234.2	49467.8	51656.7	35011.3	47409.1	0.08	0.9649
Slc6a6	24908.1	24322.5	23960.6	24112.9	22789.9	24765.7	-0.02	1.0000
Vegfa	24719.5	22729.2	21223.1	22415.5	20884.2	20004.9	-0.10	0.8214
Mia3	24569.3	24840.9	20912.8	24591.7	24234.5	25994.8	0.02	0.9878
Sparc	24242.2	24343.5	25835.7	31275.1	35225.9	38195.6	0.46	0.0012
Pkm	24091.9	26103.6	24698.1	24311.4	25252.0	22099.1	-0.11	0.8522
Amy2a3	24081.3	39007.4	49424.5	39900.2	31559.4	46324.9	0.03	0.9956
Gnai2	23998.1	23525.4	24953.1	22735.0	24235.5	22711.4	-0.02	1.0000
Insrr	23980.0	21486.5	19395.4	21487.7	20806.2	21859.9	-0.01	1.0000
Ddx3x	23945.9	18497.5	21856.8	19459.1	19682.9	20867.2	0.07	0.9168
Hnrnpu	23831.9	23184.7	22668.9	25065.1	23919.3	25750.2	0.10	0.8717
Mlec	23789.3	23771.4	21727.9	20160.8	20094.6	21017.6	-0.20	0.3426
Baiap3	23468.6	23422.5	21800.4	18033.5	17569.8	16813.9	-0.39	0.0028
Disp2	23461.1	22219.0	21008.5	23098.1	22252.9	22985.8	0.03	0.9854
Rpn2	23053.0	25250.9	21843.7	25154.3	25441.0	24397.0	0.00	1.0000
Slc38a10	22986.9	24811.8	22420.1	19136.4	19482.7	19581.0	-0.32	0.0141
Hap1	22925.1	23107.2	21240.2	20517.5	21286.7	19908.9	-0.15	0.6108
Mafa	22919.8	26062.6	22502.7	23063.0	24242.7	23784.7	-0.11	0.7571
Chic1	22818.6	18453.7	17287.6	19294.6	20394.5	18439.7	0.04	0.9881
Chd4	22802.6	22697.3	22294.1	23900.6	22803.2	25402.4	0.08	0.8938
Il1r1	22746.1	22384.8	21563.6	19863.6	21667.6	18834.6	-0.14	0.6661
Wdr6	22643.8	22891.3	22914.8	23209.5	24064.1	22817.3	0.03	0.9966
Lman1	22527.7	23090.8	19696.7	19915.6	21323.7	20493.1	-0.14	0.7075
Rgs5	22396.6	20247.5	21837.7	25123.5	25649.4	26374.3	0.31	0.0227
Tm9sf3	22329.5	21737.1	20910.8	20228.7	21335.0	20789.3	-0.06	0.9713
Ptprn	22161.1	23391.5	21231.2	25147.9	25201.7	22376.2	0.06	0.9486
Prkacb	22066.3	19555.2	19065.9	20095.0	21990.0	18045.7	0.02	1.0000
Ank	22040.7	22584.3	23176.7	20881.6	20943.8	19621.8	-0.13	0.7427
Map1b	21998.1	20082.6	22345.5	23507.8	20220.9	26556.4	0.17	0.6287

Cdh1	21961.9	21960.3	21360.1	22565.2	23303.2	22581.8	0.05	0.9186
Mrfap1	21923.5	22086.0	22754.6	21183.1	23223.2	17510.4	-0.09	0.8744
Csnk1a1	21909.7	20675.7	20357.6	20680.9	21218.9	18789.4	-0.03	1.0000
Selenop	21871.3	20202.9	20443.2	20553.6	22527.0	19432.5	0.03	0.9686
Sec61a1	21836.2	22112.4	19073.0	20053.6	19718.8	18391.7	-0.16	0.6108
Huwe1	21718.9	22311.9	18409.0	24202.1	20113.1	27772.8	0.12	0.8429
Twsg1	21667.8	20156.4	18506.7	17659.8	18918.0	17332.0	-0.15	0.6702
Sphkap	21627.3	18604.0	15583.8	19465.5	20332.8	18547.5	0.06	0.9493
2210010C04R	21567.6	44665.7	45697.6	48663.2	33491.7	58274.7	0.13	0.8759
Syt7	21550.6	21356.2	19147.5	17782.9	18742.4	20423.4	-0.15	0.6875
Cltrn	21537.8	23003.4	20584.3	18613.1	19961.2	14285.0	-0.31	0.1721
Kif5b	21440.8	20529.0	19162.6	20582.2	19806.1	19152.6	-0.04	1.0000
Sez6l2	21422.7	21763.5	20568.2	23446.3	24295.1	22004.0	0.09	0.8075
Usp9x	21364.1	20108.1	17540.5	22494.1	20798.0	24026.5	0.15	0.7271
Ywhae	21339.6	20749.5	21227.1	20915.6	21439.7	19201.5	-0.02	1.0000
Morf4l2	21295.9	21461.0	20582.3	21172.4	22291.9	19146.2	-0.03	0.9965
Slc7a2	21161.7	22987.0	23462.9	23018.5	22152.3	23879.8	0.01	1.0000
Pax6	21006.1	21246.9	20996.4	19230.9	18924.1	18216.0	-0.16	0.5550
Ddx6	20986.9	19147.0	19363.2	19505.8	19439.6	20154.4	0.03	1.0000
Slc16a10	20948.6	17796.0	16627.6	18133.3	18461.1	16120.0	-0.03	1.0000
Sptan1	20909.1	21558.5	22145.0	23128.9	21379.1	26696.8	0.12	0.7910
Prnp	20899.5	20263.0	21274.5	19317.9	21271.3	18139.0	-0.05	0.9649
Ddb1	20785.5	21062.0	20185.3	20626.8	20916.0	21252.2	0.00	1.0000
Serp1	20696.0	20537.2	18474.5	18277.6	19001.1	17896.3	-0.14	0.6480
Glul	20615.0	18700.6	19832.7	19251.1	21309.3	18797.5	0.06	0.9343
Wnk1	20463.7	21387.2	19418.6	22581.1	20072.0	24267.5	0.07	0.9377
Tmbim6	20340.1	19731.9	19857.9	19222.4	20818.5	18679.8	-0.01	1.0000
Myh9	20256.0	18930.2	18984.3	26659.6	24713.0	30857.9	0.47	0.0015
Lamp1	20143.0	20390.6	20552.1	20197.9	21413.0	17861.8	-0.04	0.9649
Gpx3	20123.8	23104.5	18696.2	25801.8	25898.9	19611.8	0.08	0.9313
H3f3b	20049.2	22139.7	23463.9	20002.6	22089.6	20711.4	-0.07	0.9649
Sh3pxd2a	20022.6	16877.6	13809.5	16669.4	15612.8	17988.7	-0.01	1.0000
Uggt1	19918.2	22237.2	19497.2	21546.1	21822.7	23092.7	0.01	0.9981
Gse1	19916.1	19009.4	18325.4	18758.5	17390.2	18750.4	-0.05	0.9335
Macf1	19803.1	19246.3	16203.5	22900.6	17028.7	26176.8	0.18	0.7168
Sez6l	19769.0	16477.7	17173.7	16132.2	17876.8	18772.2	0.06	0.9515
Dnajc3	19647.5	21638.7	17976.8	16830.7	17419.9	17414.4	-0.29	0.0658
Rrbp1	19622.0	19925.9	18831.2	22921.9	21940.7	26329.9	0.24	0.3087
Madd	19507.9	14639.3	13782.3	15665.2	15928.1	14639.1	0.03	0.9823
Ids	19492.0	16738.3	17278.5	19492.0	19893.4	19029.4	0.19	0.4655
Cela1	19455.7	29122.7	28470.4	28659.5	20726.1	31254.6	-0.03	1.0000
Copg1	19434.4	19745.6	17820.6	19774.4	18897.4	19231.4	-0.02	1.0000
Cnot6l	19433.4	18537.5	18521.8	19029.2	18826.6	19398.9	0.04	1.0000
Eif3a	19430.2	18526.6	17841.7	21269.0	20129.5	22151.6	0.18	0.5071
Ptbp3	19388.6	17320.4	18040.2	18023.9	18889.2	18165.3	0.07	0.9215
Itgb1	19335.3	19355.6	20321.3	20542.9	21321.6	21824.6	0.12	0.6983
Prpf8	19188.3	18865.5	16326.4	21697.9	20357.5	23865.3	0.21	0.4223
Nckap1	19157.4	18342.6	18301.2	19471.9	19181.9	18833.7	0.06	0.9452
Reg1	19122.2	29627.4	35277.4	43200.5	29843.7	45731.6	0.24	0.7066
Gm15459	19113.7	19056.8	18035.2	21606.6	20018.7	19876.3	0.10	0.8075
Igfbp7	18882.5	17132.7	19386.3	19127.9	20243.5	17071.1	0.10	0.8795
Golgb1	18852.6	17837.9	15018.6	17876.4	16015.3	20767.6	0.04	0.9684
Mir6236	18812.2	15757.1	33573.7	12762.9	12891.9	26199.5	-0.11	0.9343
Resp18	18804.7	18937.5	18557.1	15377.5	18277.3	11851.1	-0.26	0.3583
Slc7a14	18699.2	16716.4	15598.9	16177.9	15792.5	17086.5	-0.03	0.9695
Pdia6	18670.4	22772.9	18743.5	20376.3	20522.8	19268.5	-0.14	0.6193
Bsg	18656.6	19315.6	19075.0	17335.0	19427.2	16005.9	-0.12	0.8445
Cyb5r3	18642.7	18607.7	18708.2	18229.9	19329.7	17541.2	-0.02	1.0000
Arf1	18626.7	19059.6	18428.1	18132.2	18536.0	16538.5	-0.09	0.8560
Spint2	18517.0	19633.5	18679.0	17499.5	18701.3	15642.7	-0.16	0.6422
Pabpc1	18423.2	17708.5	18318.3	18811.6	19301.0	19793.0	0.11	0.7639
Pnmal2	18423.2	17582.8	17811.5	16394.4	18107.9	16079.3	-0.06	0.9474
Serinc1	18393.4	18104.8	18095.6	17467.7	18537.0	17097.4	-0.03	0.9933
Camk2n1	18388.1	17815.1	20517.8	17118.4	17082.1	15501.4	-0.11	0.8068
Scg5	18352.9	18062.9	18030.2	17015.4	19940.6	13725.2	-0.08	0.9649
Sec31a	18351.8	18056.5	16835.2	19462.3	18866.6	20311.1	0.11	0.7896
Flnb	18274.1	15897.4	12362.7	18654.5	16642.7	20944.2	0.21	0.6642
Ddost	18214.4	20826.0	17979.8	19429.4	20097.7	18009.5	-0.09	0.8295
2900097C17R	18213.3	17006.1	16960.1	16652.4	17571.9	17750.4	0.02	1.0000
Ssr3	18208.0	19622.6	17448.8	17584.4	18409.7	16917.1	-0.13	0.7108
Ddr1	18177.1	18183.1	18360.6	15767.1	16916.8	15567.5	-0.17	0.4954
Tle5	18168.6	19278.2	18940.0	18595.0	19146.9	17226.0	-0.06	0.9316
Golga4	18162.2	18245.1	15930.4	17564.3	16380.9	19578.3	-0.02	1.0000
Map2	18145.1	16803.9	16156.1	17103.6	15675.5	16802.1	-0.03	0.9810
Tspan7	18102.5	19166.1	18185.3	17329.7	17807.0	16327.5	-0.14	0.6413
Rap1b	18027.9	16271.8	16010.0	15611.0	16756.7	13120.1	-0.10	0.8710

Chd3	17994.9	18450.1	18474.5	18268.1	17033.9	18754.0	-0.03	1.0000
Gtf2i	17959.7	17268.5	17921.3	17952.8	18683.9	18761.3	0.09	0.8983
Dynll2	17889.4	16678.1	18628.6	16887.0	18739.3	17988.7	0.08	0.8830
Fndc3a	17861.7	17101.8	16085.6	17325.4	17135.5	16892.7	0.00	1.0000
Ppp1cb	17809.5	16374.8	17436.7	16045.2	17353.2	14906.3	-0.03	0.9927
Rpl8	17796.7	19419.4	20509.7	19444.3	20909.9	17451.5	-0.01	1.0000
Itpkb	17792.4	18240.5	17607.0	17173.6	18266.0	19607.3	0.01	1.0000
Mtch1	17782.8	17801.4	19598.9	17481.5	18590.4	15320.2	-0.06	0.9559
Hnrnph1	17763.7	17802.3	17619.1	18580.2	16314.1	19245.9	0.02	1.0000
Gm26917	17756.2	18104.8	16862.4	7063.5	7151.4	8871.1	-1.09	0.0000
Taok2	17741.3	17714.9	17523.4	16424.2	15001.9	15644.5	-0.16	0.5981
Amy2a4	17734.9	29611.0	35047.7	30595.7	22594.8	39662.0	0.06	0.9818
Iqgap1	17680.5	15873.7	13531.4	18042.0	17122.2	19718.7	0.18	0.5914
Rack1	17675.2	19255.4	19156.6	18168.3	19002.2	15061.2	-0.12	0.8599
Hbb-bs	17567.6	16175.2	19244.3	12400.9	15039.9	9325.8	-0.32	0.4437
Cbx6	17522.8	16513.2	17957.6	16593.0	17168.4	16099.2	0.00	1.0000
Gnb1	17470.6	17352.3	18214.5	17873.2	18348.1	17376.4	0.04	0.9850
Txnip	17425.9	13524.2	20035.2	17831.8	17186.9	12915.4	0.12	0.8262
Zfp516	17352.4	18464.7	16628.7	16908.2	15513.3	17436.1	-0.13	0.7319
Tubb5	17348.1	17807.8	16562.2	19375.3	17890.2	19527.6	0.09	0.8602
Rpn1	17308.7	20608.3	17349.1	18265.9	18575.0	17071.1	-0.16	0.5618
Lgals3bp	17278.8	17646.6	16395.9	15942.2	16816.2	14702.5	-0.14	0.7539
Pclo	17230.9	16876.7	14985.3	19022.8	14914.7	19751.3	0.08	0.9244
Spcs3	17076.4	18072.9	15926.4	17722.4	18544.2	15516.8	-0.04	1.0000
Gjd2	17065.7	15677.8	17345.0	15672.6	17976.4	13986.0	0.00	1.0000
Gnb2	17018.8	17320.4	18072.5	15530.4	16037.9	14066.7	-0.17	0.5492
Kif1b	16887.8	15871.9	16128.9	17148.1	15645.7	18672.5	0.09	0.8705
Insm1	16867.5	14947.2	14785.8	14268.2	14297.6	11318.5	-0.16	0.6701
Lamc1	16866.5	15417.3	16095.7	21861.4	20968.4	26541.9	0.50	0.0007
Pkd1	16822.8	15497.4	12546.0	14960.3	12548.0	16323.8	-0.06	0.9731
Scgn	16778.0	16864.9	17229.2	14181.1	16401.4	13312.1	-0.19	0.5550
Ptms	16767.4	15102.1	18750.6	16635.4	16613.9	16303.0	0.09	0.8455
Fam120a	16748.2	15902.9	15154.6	16293.6	15587.2	16537.6	0.02	1.0000
Cyfip2	16693.9	15569.4	14534.0	15908.3	16267.9	16680.7	0.06	0.9500
Arcn1	16677.9	16774.7	15348.1	16210.8	15530.7	15717.9	-0.07	0.9012
Mprip	16652.3	17081.7	16594.4	17608.8	16559.5	18341.0	0.04	0.9850
Unc80	16650.2	15660.5	13933.4	16597.2	14327.4	16597.4	0.02	1.0000
Aff4	16632.1	14826.9	14178.3	16467.7	15127.2	18037.6	0.13	0.7648
Marcks	16629.9	16000.3	20545.0	19089.7	18875.9	16790.3	0.14	0.7619
Copb2	16592.6	16628.9	14849.3	16037.8	16435.3	15770.4	-0.04	1.0000
Creb3l2	16559.6	15124.8	14467.5	15311.7	15341.8	16047.6	0.03	0.9662
Sytl4	16534.0	16881.3	16020.1	17516.5	18762.9	16521.3	0.06	0.9343
Brd2	16476.5	16356.5	15958.6	16465.6	15955.8	15230.6	-0.04	1.0000
Trim35	16457.3	16874.0	16879.5	15715.1	16489.7	14992.4	-0.09	0.8406
Uba1	16448.8	16769.2	16070.5	17551.5	17125.3	17756.8	0.06	0.9662
Stt3a	16412.5	16997.9	15190.9	16135.4	16452.7	16755.0	-0.03	0.9831
Ssr1	16388.0	16261.8	14739.5	15400.8	15580.0	15858.3	-0.05	0.9981
H1f0	16308.1	17806.0	17880.0	13597.3	16138.5	14871.9	-0.23	0.3287
Anapc5	16234.6	16908.6	15961.7	15482.6	15141.6	14952.5	-0.14	0.6393
Tcf25	16190.9	16087.8	15623.1	16078.1	15940.4	15845.6	-0.01	1.0000
Rad21	16091.8	15485.6	14481.6	14878.6	15370.5	15693.4	-0.01	1.0000
Actn4	16065.2	15763.5	15809.5	18250.0	18402.5	19501.3	0.23	0.2093
Tmed8	15949.0	15312.5	14213.5	14199.2	13423.8	14036.8	-0.13	0.7420
Castor2	15925.6	16788.4	16121.9	18919.9	17909.7	17901.7	0.12	0.7470
Celf3	15925.6	15526.6	14120.9	15141.8	14639.5	12811.3	-0.11	0.7977
Nktr	15896.8	15109.3	12760.7	14510.2	11092.0	16679.8	-0.07	0.9649
Ahcyl1	15886.2	14900.7	14985.3	16216.1	15123.1	15457.9	0.06	0.9186
Tgoln1	15870.2	14877.0	14638.7	14405.1	16424.0	14475.2	0.02	1.0000
Kif12	15863.8	16050.4	14412.0	13864.8	12870.4	11613.8	-0.29	0.1654
Calm3	15807.3	15013.7	16395.9	14115.3	15215.5	14025.9	-0.06	0.9311
Sf3b1	15774.3	15227.8	14141.0	16041.0	14833.5	16172.6	0.04	0.9729
Ncl	15767.9	16183.4	15610.0	17262.8	17189.9	18028.5	0.11	0.7741
Arf3	15754.0	14255.7	15354.1	14224.7	15058.4	14638.2	0.02	1.0000
Vwa5b2	15719.9	15344.4	13252.3	12343.6	11772.8	11535.0	-0.32	0.0522
Ranbp2	15704.0	15030.1	13183.8	14778.8	12992.6	16644.5	-0.01	1.0000
Vcp-rs	15683.7	16313.7	14983.3	15629.1	16360.3	16111.9	-0.01	1.0000
Eif4a2	15631.5	15403.6	15621.1	14709.8	16049.2	13535.9	-0.06	0.9521
Tnks2	15609.1	15393.6	15543.5	14174.8	13733.9	13999.6	-0.13	0.6256
Rab1a	15597.4	15188.6	15367.2	15074.9	15804.9	14070.3	-0.02	1.0000
Gm12715	15584.6	14870.7	15511.3	15685.3	16623.2	16927.1	0.13	0.7075
Rbm39	15489.8	15033.7	14746.5	15407.2	14161.0	14507.8	-0.03	0.9874
Rps3	15481.3	16461.3	17672.5	15676.8	17135.5	11539.6	-0.13	0.8754
Pcbp2	15474.9	15648.7	16541.0	15432.7	16902.5	15794.0	0.03	0.9973
Uso1	15466.3	16048.6	13903.2	14181.1	14698.0	13878.3	-0.14	0.6226
Atp8b1	15455.7	15939.3	14956.1	15981.5	15515.3	14972.4	-0.03	1.0000
Cela3b	15421.6	31439.5	32299.1	32954.5	19432.4	28041.8	0.00	1.0000

Gm12183	15311.8	14445.2	14589.4	16539.9	17050.3	15482.4	0.16	0.5731
Smarcc2	15296.9	14920.8	14495.7	15315.9	14367.4	15525.9	0.01	1.0000
Sqstm1	15261.8	15031.0	15012.5	14237.4	15115.9	13376.5	-0.07	0.8787
Pak3	15251.1	14575.5	13405.5	14654.6	14963.9	15045.8	0.03	1.0000
Sfpq	15211.7	14054.4	15006.5	15210.8	13368.4	14247.8	0.01	1.0000
Ap1s2	15136.0	14283.0	14474.5	13759.7	14338.6	13196.2	-0.05	0.9839
Trp53inp2	15021.0	14235.7	13146.6	13650.4	14157.9	14314.8	-0.02	1.0000
Srsf6	15011.4	14967.2	15021.6	14778.8	14051.2	15041.3	-0.03	0.9857
Lman2	15007.1	15593.1	14063.4	12911.5	13687.7	13007.8	-0.21	0.2888
Arfgef3	14987.9	13976.9	12251.8	15531.4	13958.7	16798.5	0.13	0.8339
Pafah1b1	14985.8	14278.5	14650.8	14244.8	14201.1	14735.1	0.01	1.0000
Itpr3	14921.9	15636.8	14179.3	14597.3	14042.9	15167.2	-0.08	0.8816
Rbp4	14892.0	13979.7	15157.6	10484.8	10502.7	8696.3	-0.46	0.0016
Frzb	14865.4	14513.5	14924.9	12702.4	14581.0	12208.9	-0.13	0.7535
Caprin1	14846.2	14014.3	13844.8	15018.7	14631.3	15153.6	0.08	0.8429
Pja2	14761.0	13510.5	13810.5	13641.9	14034.7	13966.1	0.03	0.9896
Eif4b	14759.9	13276.4	14930.9	13544.2	14836.6	12817.6	0.03	1.0000
Camk2b	14752.4	14672.1	13995.9	14038.9	14263.7	13700.7	-0.06	0.9067
Snrnp70	14723.7	14290.3	13436.7	13911.5	11818.0	14483.3	-0.08	0.9542
Snd1	14707.7	15779.9	15179.8	17526.0	17448.7	16429.8	0.12	0.7873
Map3k15	14682.1	16511.4	15232.2	17156.6	16902.5	15726.0	0.02	1.0000
Tsc22d1	14633.1	14353.2	15389.4	15428.4	16589.3	14969.7	0.11	0.7977
Hdac6	14620.3	14478.9	14604.5	13341.5	13298.5	12635.5	-0.14	0.7073
Glg1	14617.1	13700.0	13754.1	15955.0	15829.5	17491.4	0.24	0.2338
Numa1	14610.7	15009.1	13973.8	15158.8	14661.0	16205.2	0.04	1.0000
Bace2	14591.5	13428.5	14385.8	10755.5	13127.1	10949.9	-0.20	0.4745
Tmem59	14577.7	14068.0	14926.9	11653.6	14144.6	11353.9	-0.17	0.6256
Hnrnpab	14524.4	15013.7	13920.4	15570.7	14831.5	14685.3	0.01	1.0000
Sort1	14506.3	13968.7	13312.8	13349.9	14083.0	13834.8	-0.02	1.0000
Nnat	14492.4	14313.1	14514.8	9838.4	8714.1	8825.9	-0.60	0.0000
Tmem30a	14491.4	13682.7	13709.8	12952.9	13791.4	11640.1	-0.09	0.8727
Nfe2l1	14474.3	13929.6	14231.7	13687.5	14256.5	13909.1	0.00	1.0000
Fkbp9	14473.3	15564.0	15022.6	14835.0	14785.3	14158.1	-0.08	0.8791
Slc25a3	14449.8	15724.3	14647.8	15849.9	16180.6	15425.3	0.02	1.0000
Hnrnpl	14447.7	13915.9	13082.1	13490.1	13349.9	13256.9	-0.05	0.9559
Rsrp1	14420.0	13303.7	12646.8	14823.4	14069.6	13977.9	0.09	0.9096
Rcan3	14409.3	13795.7	13209.0	13424.3	13112.7	12927.2	-0.06	0.8998
Srpr	14402.9	15426.4	13461.9	14991.1	15732.0	13999.6	-0.03	0.9925
Kpnb1	14335.8	14396.9	14268.0	16013.4	14844.8	16663.5	0.13	0.7713
Gng4	14303.8	13186.2	11697.7	12524.1	12562.4	11601.1	-0.09	0.8710
Prrc2c	14216.5	13671.8	12352.6	16171.5	13026.5	18477.8	0.19	0.5988
Cnbp	14216.5	14243.9	15304.7	13495.4	14445.4	12288.6	-0.09	0.8560
Ap3d1	14205.8	12979.4	12502.7	12879.7	12509.0	13404.5	-0.01	1.0000
Atp6v1b2	14187.7	13335.6	13680.6	13619.6	14508.1	12391.0	0.01	1.0000
Ncor1	14182.4	14458.9	13488.1	15068.6	13495.7	15717.9	0.03	0.9915
Selenot	14172.8	12309.8	13380.3	12335.1	14057.3	11195.4	0.00	1.0000
Plxnd1	14167.5	13556.1	13227.2	15934.8	14840.7	19389.0	0.26	0.3225
Ubr5	14108.8	13395.7	12890.6	14925.3	13462.8	16274.0	0.14	0.7535
Tshz1	14102.5	14072.6	14308.3	14351.0	14153.8	13013.2	-0.02	1.0000
Rab2a	14084.3	13936.9	14383.8	13408.3	14531.7	12358.4	-0.05	0.9952
Hnrnpk	14079.0	13780.2	13748.1	13995.4	14059.4	13440.8	0.00	1.0000
Gstz1	14073.7	14461.6	12720.4	11804.3	11651.6	11140.1	-0.29	0.0762
Nbr1	14069.4	13969.7	13759.1	12771.4	13271.8	12695.3	-0.11	0.7817
Gns	14044.9	13710.0	13379.3	13348.9	14046.0	14001.4	0.01	1.0000
5330417C22R	14040.7	13908.6	13839.7	14610.0	14543.0	13464.3	0.03	1.0000
Dcaf7	14018.3	13059.5	13105.2	14165.2	14567.6	14954.3	0.14	0.6461
Arhgef12	13950.1	12515.7	12245.8	13782.0	13362.2	15476.9	0.16	0.6313
Birc6	13946.9	13315.5	10910.8	14580.3	11713.2	16880.9	0.11	0.9217
Ret	13932.0	13538.7	14193.4	12385.0	12761.5	12195.3	-0.12	0.7048
Tram1	13932.0	14257.5	13146.6	13220.4	13452.6	12269.6	-0.12	0.7406
Akr1a1	13929.8	15056.5	14839.2	13479.5	14511.1	11851.1	-0.15	0.7353
Sec24d	13917.0	14121.8	12277.0	12631.3	12754.4	13173.6	-0.11	0.7619
Adgrg1	13909.6	13860.3	12750.6	13412.6	13837.6	13404.5	-0.02	0.9810
Ubqln2	13869.1	12764.4	12925.9	13201.3	13106.5	12092.1	0.00	1.0000
Col15a1	13854.2	13301.0	13727.9	19257.4	19191.1	23135.3	0.56	0.0000
Plvap	13844.6	13670.8	13344.0	13731.0	16574.9	16196.1	0.16	0.6372
Saraf	13829.7	13004.9	12397.9	11465.7	13117.8	10601.2	-0.13	0.7749
H2-K1	13820.1	15006.4	13654.4	12879.7	14193.9	9913.7	-0.22	0.5723
Eif5	13789.2	13528.7	13010.5	12348.9	13090.1	11929.9	-0.11	0.7952
Try5	13741.2	29982.7	25103.2	26099.1	19231.1	34912.1	0.07	0.9810
Ndfip1	13707.1	13409.4	14284.1	12912.6	13995.7	11854.8	-0.06	0.9561
Anxa4	13691.2	13246.3	13073.0	11922.2	12363.2	11900.0	-0.13	0.6695
Fbxl16	13645.3	12776.2	12593.4	12236.4	12636.3	11322.2	-0.08	0.9371
Itpr1	13628.3	13281.8	12553.1	13757.6	12434.0	13265.0	-0.01	1.0000
Nsd1	13587.8	13229.9	12014.1	13896.6	11993.5	15161.7	0.05	1.0000
Ncor2	13561.2	14048.9	12953.1	15496.4	13649.7	17821.1	0.15	0.7362

Syp	13554.8	13129.7	14029.2	11765.1	12883.7	11563.1	-0.12	0.7337
Ptpn11	13545.2	12794.4	11682.6	13047.4	13945.4	13394.6	0.07	0.9649
Arhgdia	13507.9	13660.8	13380.3	12912.6	13355.0	13294.0	-0.05	0.9559
Sec16a	13449.3	12044.7	10829.2	13197.1	12193.8	14429.0	0.12	0.8217
Manf	13439.7	14417.9	11851.8	11772.5	11885.7	11892.8	-0.24	0.2533
Kmt2d	13423.7	13167.0	10849.3	13619.6	10327.1	15381.8	0.02	0.9965
Papola	13383.2	14015.2	13797.4	13599.4	13316.0	12872.8	-0.07	0.9490
Eif4h	13307.6	13526.9	13669.5	12879.7	13689.7	13094.8	-0.03	1.0000
Hp1bp3	13306.5	12878.2	12383.8	11767.2	12444.3	11958.9	-0.09	0.8602
Ogdh	13292.6	13210.8	12426.1	14381.8	13932.0	15025.9	0.12	0.7427
Trip12	13286.2	12458.3	11503.2	14244.8	13320.1	15457.9	0.18	0.5220
Eif4g3	13279.9	13397.5	12240.8	14067.5	13163.0	14783.1	0.07	0.9343
Atp2b1	13260.7	12533.0	11735.0	13529.3	12966.9	13802.2	0.09	0.9057
H2-D1	13241.5	13842.1	12460.4	11483.8	13367.3	9132.9	-0.23	0.5060
Tnpo1	13210.6	11836.9	11268.5	14042.1	13720.5	15238.7	0.24	0.2779
Ltbp4	13201.0	12568.5	12775.8	20368.9	20755.9	23842.7	0.71	0.0000
Adgrl1	13198.9	13388.4	12106.8	12724.7	11722.5	13048.6	-0.08	0.8826
Kdr	13174.4	12403.6	12307.3	15837.1	16547.2	17525.8	0.39	0.0039
Emb	13165.8	12321.6	12909.8	11995.4	13201.0	11210.8	-0.03	1.0000
Hnrnpul2	13139.2	12899.2	12621.6	12565.5	12804.7	13642.8	0.01	1.0000
Usp34	13117.9	13263.6	11347.1	14240.6	12519.2	15585.6	0.10	0.8833
Sar1a	13114.7	12665.1	11757.1	11903.1	12547.0	11956.2	-0.05	0.9304
Lrp11	13114.7	13294.6	12586.4	12874.4	13613.8	11132.9	-0.07	0.9377
Cst3	13106.2	14277.6	14584.3	12770.3	14115.8	10449.0	-0.16	0.6983
Etnk1	13105.1	12256.9	12418.1	12663.1	12395.0	12771.4	0.03	0.9649
Dbpht2	13095.5	13320.1	14952.1	11534.7	12980.2	9847.6	-0.20	0.5571
Ucp2	13082.7	11979.1	13281.6	12844.7	13177.4	12643.7	0.09	0.8597
Gdi1	13024.1	13527.8	13087.1	12904.1	13105.5	12115.6	-0.08	0.8773
BC005537	12979.4	11211.1	12606.5	11996.5	12191.7	11719.8	0.07	0.9007
Pdia4	12876.0	14226.6	11680.6	11635.6	11376.5	11980.7	-0.24	0.1800
Rab27a	12854.7	12909.2	13089.1	12014.5	12543.9	10732.5	-0.12	0.7648
Tceal9	12805.7	13263.6	12931.9	13237.4	14408.5	11584.8	-0.01	1.0000
Rplp0	12799.3	13783.8	14121.9	13632.3	13932.0	12291.3	-0.04	0.9515
Tjp1	12787.6	12039.2	10857.4	15065.4	14183.6	16196.1	0.30	0.1148
Nav2	12772.7	12057.4	10592.4	12486.9	11110.5	14812.1	0.08	0.8987
Tns3	12743.9	12072.9	10811.0	11654.7	11380.6	12343.0	-0.03	1.0000
Setd5	12733.2	12064.7	10694.2	14280.9	12755.4	14967.9	0.20	0.5681
Ptma	12713.0	13818.4	13965.7	13158.9	13763.7	13525.0	-0.03	1.0000
Gm1821	12709.8	12730.7	12049.3	12114.3	13000.8	10634.7	-0.08	0.9126
Vps35	12670.4	12699.7	12313.3	11723.7	12561.3	11359.3	-0.09	0.8605
Ehmt2	12669.3	12323.4	11972.8	10851.1	11111.6	11190.8	-0.15	0.6188
Laptm4a	12661.8	12795.3	13612.0	12239.6	13758.5	11551.3	-0.03	0.9972
Tmsb4x	12642.7	13234.5	15313.8	13206.6	13969.0	11319.4	-0.05	0.9775
Cdc42	12622.4	12663.2	12996.4	12784.1	13161.0	11793.2	-0.01	1.0000
Ldlr	12616.0	13802.9	11453.9	14318.1	12880.7	12623.8	-0.03	1.0000
Col3a1	12612.8	12691.5	19636.2	25034.3	28663.9	49344.7	0.49	0.1196
Cds2	12601.1	11503.5	12527.9	10855.3	11315.9	11413.6	-0.05	0.9915
Nucb1	12594.7	13544.2	12365.7	12933.8	13728.8	12546.8	-0.04	0.9810
Arid1a	12584.1	12116.6	12236.7	14252.3	12255.4	14983.3	0.17	0.6281
Xbp1	12570.2	13475.0	12700.2	12149.3	13081.9	11378.3	-0.12	0.7629
Smarca4	12557.4	12229.6	11929.4	14549.5	13381.7	14695.3	0.20	0.3711
Tln1	12494.5	12493.8	11013.6	14300.0	12517.2	16788.5	0.20	0.5601
Neurod1	12481.8	12497.4	12834.2	12991.1	13537.8	11349.3	0.01	1.0000
Eif4a-ps4	12481.8	12694.2	12388.9	12900.9	12320.0	11452.6	-0.05	0.9694
Phactr1	12480.7	11306.7	11390.4	13480.5	13702.1	15042.2	0.28	0.1196
Kif1a	12466.8	12035.6	12760.7	13070.8	13170.2	14689.8	0.16	0.5699
Rhoa	12464.7	12549.4	12683.1	12289.5	13347.8	11729.8	-0.01	1.0000
Aco2	12424.2	12551.2	12256.9	12622.8	13217.4	11784.1	0.00	1.0000
Kidins220	12379.5	11381.4	10783.8	12322.4	11338.5	12593.9	0.08	0.9110
Hspa8	12378.4	12243.3	11339.0	14450.8	13227.7	12860.2	0.14	0.6861
Hba-a1	12377.3	11225.6	13735.0	8426.5	8620.6	9001.6	-0.37	0.0122
Tm4sf4	12369.9	11227.5	13030.7	12124.9	14201.1	9619.3	0.05	0.9896
Arfgef1	12359.2	12154.0	10727.4	12374.4	11696.8	12903.6	0.03	0.9972
Nkx2-2	12353.9	12504.7	12351.6	12126.0	12803.6	11337.6	-0.04	1.0000
Rere	12341.1	11911.7	11708.8	14156.7	12876.5	14706.1	0.20	0.3906
Rtn4	12329.4	11093.6	14099.7	12296.9	12292.3	11173.6	0.06	0.9162
Ptbp1	12304.9	12212.3	11695.7	12710.9	11990.5	12596.6	0.03	0.9748
Cnot1	12269.7	12204.1	10564.2	13251.2	11607.5	14050.4	0.09	0.8937
Uaca	12260.1	10761.0	11772.2	12183.3	12688.6	12890.1	0.19	0.4140
Bmp1	12226.0	11581.9	11579.8	9730.1	9683.3	9528.7	-0.25	0.1402
Ganab	12215.4	12731.6	11497.2	12755.5	12975.1	12386.4	0.01	1.0000
Slc43a2	12213.2	12546.6	12881.6	12715.1	12573.7	13732.4	0.05	0.9395
Zfhx2	12213.2	11329.5	9118.4	11805.4	9920.5	12110.2	0.01	1.0000
Itm2c	12211.1	13540.6	12342.5	12124.9	13186.6	10418.2	-0.15	0.7277
Rtn3	12210.0	11969.0	12637.7	11628.1	12146.5	12026.9	-0.01	1.0000
Ctsb	12202.6	12214.1	13219.1	12318.1	12479.2	11921.8	0.00	1.0000

Golim4	12179.1	11461.6	10799.0	10905.2	10534.5	11378.3	-0.06	0.9126
Zbtb20	12169.6	10630.8	10567.2	13774.6	11993.5	16881.8	0.34	0.1346
Herc2	12165.3	12026.4	10068.5	12085.6	10470.9	14472.4	0.05	0.9728
Atrx	12163.2	11810.5	11044.8	12875.4	11685.5	14241.5	0.12	0.8010
Fam174b	12163.2	12420.0	11301.7	10468.9	10866.2	10518.7	-0.20	0.3465
Fasn	12160.0	12067.4	10194.4	12723.6	11070.5	13773.2	0.06	0.9877
Mycbp2	12156.8	12062.9	10699.2	12858.5	11132.1	14947.1	0.10	0.8775
Srp72	12140.8	12726.1	11624.1	11953.0	11935.0	12165.4	-0.07	0.9162
Kmt2a	12136.5	12249.6	10281.1	12076.1	9399.9	13232.4	-0.05	0.9624
Mcf2l	12134.4	11933.5	10955.1	11039.0	11223.5	11046.8	-0.09	0.8558
2610507B11R	12126.9	12555.7	11476.0	11638.7	10924.7	12384.6	-0.09	0.8714
9530068E07R	12126.9	11817.8	11978.8	10712.0	11756.4	9567.7	-0.14	0.7190
Epb41l3	12122.7	11951.7	12764.7	12086.7	12262.5	11834.8	0.01	1.0000
Srsf1	12104.6	12031.0	11260.4	11318.2	10957.5	11428.1	-0.09	0.9057
Cmtm4	12103.5	11686.6	10546.1	11934.9	11359.0	12071.2	0.02	1.0000
Tm9sf2	12099.2	11334.1	10876.5	11062.3	11979.2	10912.8	0.00	1.0000
Rab7	12068.3	11092.6	11799.5	11018.8	11808.7	10583.1	-0.01	1.0000
Lasp1	12049.2	12684.2	12764.7	11456.2	12085.9	12508.7	-0.07	0.9126
Zdhhc2	12047.0	10411.2	11697.7	10276.8	11853.9	10436.3	0.03	0.9966
Ubc	12043.8	11665.7	12246.8	14981.5	15259.6	14241.5	0.32	0.0253
Tmpo	12033.2	12109.3	10919.9	11348.9	10764.5	11454.4	-0.10	0.8826
Stxbp1	12030.0	11097.2	11396.4	13037.9	12524.4	12965.2	0.19	0.3625
March6	12024.6	11779.6	11337.0	12380.8	12592.1	12735.2	0.09	0.8445
Fth1	12008.7	12852.7	13945.5	13019.8	13958.7	13687.1	0.07	0.9343
Zmiz1	12004.4	11364.1	11514.3	12584.6	11393.9	14364.7	0.14	0.7414
Grb10	11993.7	13233.5	13385.3	11949.8	12243.0	12689.9	-0.09	0.8292
Nfat5	11958.6	11093.6	10024.1	11634.5	9117.6	13852.0	0.05	0.9599
Sipa1l3	11916.0	11545.4	10578.3	11415.8	10573.5	12245.1	-0.01	1.0000
Calm2	11877.6	11927.1	12766.7	10783.1	11716.3	10176.4	-0.13	0.8036
Pcbp1	11830.7	11386.0	11526.4	11454.0	11680.4	10509.7	-0.02	1.0000
Wls	11800.9	11417.9	11039.8	10669.6	11577.7	10564.9	-0.06	0.9831
Sel1l	11788.1	13415.8	12278.0	13632.3	11609.5	16572.0	0.08	0.9377
Atp1b1	11786.0	10584.3	11348.1	11229.0	11529.4	10759.7	0.06	0.9340
Dynlt1-ps1	11765.7	12645.9	7495.2	10868.1	12079.8	8639.3	-0.05	1.0000
Serinc3	11749.7	11251.2	12483.6	11898.8	12588.0	12396.4	0.11	0.7830
Purb	11741.2	10523.2	10050.3	11369.1	10582.8	13067.6	0.12	0.8104
Ube2d3	11724.2	11309.5	11014.6	10813.9	11345.7	9814.1	-0.08	0.9096
Dag1	11722.0	11414.2	11129.4	12422.2	12605.5	12922.7	0.14	0.6191
Pdx1	11717.8	11808.7	10412.1	10668.5	11230.7	10152.8	-0.12	0.7470
Eif5a	11697.5	13157.9	12209.5	12373.3	12718.4	10833.0	-0.11	0.8730
Spcs2	11665.6	11559.1	11681.6	10410.5	11376.5	9534.2	-0.14	0.7420
Mbnl2	11649.6	10721.9	10879.6	11182.3	10895.9	11451.7	0.05	0.9633
Larp1	11629.3	11109.0	11229.2	12300.1	11564.4	12806.7	0.12	0.7502
Ocrl	11626.1	11972.7	11092.1	12033.6	11029.4	11480.7	-0.04	0.9731
Rnf130	11620.8	12362.6	10870.5	12956.1	12681.5	11184.5	0.01	1.0000
Hipk3	11613.3	10488.6	10406.0	12082.5	11887.8	11618.4	0.16	0.6576
Ywhag	11579.2	10082.3	11205.0	10340.5	10207.0	10444.5	0.02	1.0000
Ankrd11	11566.5	11690.3	11078.0	12350.0	10746.0	13585.7	0.06	0.9234
Bag6	11544.1	12175.8	11338.0	11690.8	11509.9	11736.1	-0.05	0.9525
Sesn3	11539.8	11727.6	10947.1	12492.2	12057.2	12416.3	0.07	0.9445
Gm15500	11534.5	11960.8	13756.1	11167.4	12465.8	9795.0	-0.10	0.8791
Hbb-bt	11534.5	9390.8	13642.3	7966.9	7692.5	6265.2	-0.39	0.0385
Ttc3	11530.2	11281.2	11677.5	11212.0	11008.9	11761.5	0.00	1.0000
Pum2	11518.5	11475.3	11040.8	11613.3	11226.5	12084.8	0.02	1.0000
Tnr	11514.3	11399.7	9873.0	12193.9	11602.3	12871.0	0.10	0.8455
Acin1	11495.1	11158.2	10948.1	11796.9	10690.6	11634.7	0.02	1.0000
Anxa5	11489.7	11580.9	11521.4	11789.5	12702.0	11718.0	0.06	0.9194
Ywhab	11459.9	11021.6	11438.7	11368.0	11734.8	11151.0	0.04	0.9737
Bptf	11436.5	10813.9	10337.5	11916.9	10726.5	13079.4	0.12	0.7873
mt-Rnr1	11431.1	10063.2	12076.5	8987.0	10168.0	9659.2	-0.09	0.8698
Sf3b2	11424.7	11847.9	11590.9	12077.2	12382.7	12219.8	0.05	0.9372
Zc3h7b	11417.3	11805.1	11467.0	11105.8	10821.0	11713.5	-0.06	0.9630
Ffar1	11396.0	10522.3	10353.6	2883.1	2701.4	2664.8	-1.78	0.0000
March8	11385.3	10818.4	11190.9	10091.0	11053.0	9796.8	-0.07	0.9557
Trim44	11378.9	10956.0	10861.4	11241.7	11103.3	12040.4	0.06	0.9474
Cyb5b	11340.6	11500.8	10221.6	12702.4	12203.0	12208.0	0.11	0.7932
Dhx9	11327.8	11275.8	10379.8	12083.5	11927.8	13005.1	0.13	0.7873
Wdr26	11323.5	10878.5	11385.3	11323.5	10637.2	11256.0	0.02	1.0000
Csde1	11313.9	10798.4	11203.0	12153.6	12369.3	11532.3	0.14	0.7148
Abhd2	11302.2	10675.4	9997.9	11588.8	11003.7	12603.8	0.12	0.7830
Sdk2	11297.9	11266.6	9830.7	11957.2	10661.8	13233.3	0.09	0.8702
Tmem65	11283.0	9709.7	9377.3	10526.2	10489.3	9456.3	0.05	0.9810
Aldoa	11274.5	11429.7	11693.7	11235.4	11622.9	10356.6	-0.04	0.9789
Mbtps1	11267.0	11761.3	11234.2	10146.2	10290.1	9972.6	-0.19	0.3384
Arhgef17	11266.0	10191.6	11019.6	10885.0	8915.3	10891.0	-0.01	1.0000
Isl1	11263.8	12147.6	11256.4	11396.7	11426.8	9557.7	-0.14	0.7428

Srsf5	11253.2	10978.8	11323.9	10836.2	10345.6	11749.7	0.00	1.0000
Shc1	11253.2	10139.7	10623.6	9086.8	9872.3	9319.5	-0.11	0.7648
Hectd1	11248.9	10941.4	9980.8	12170.6	11461.7	13129.2	0.15	0.6689
Rpsa-ps10	11219.1	12180.4	8048.3	12132.4	12034.6	10205.3	-0.01	1.0000
Ubl3	11211.6	11536.3	11572.8	10854.3	11512.0	9306.8	-0.11	0.8727
Flna	11203.1	12043.8	11438.7	15318.0	13484.4	20945.1	0.40	0.0679
Clstn1	11196.7	11369.6	11793.4	10277.8	10824.1	11166.4	-0.08	0.8911
Tmed10	11169.0	11570.9	10910.8	10827.7	11061.2	10856.6	-0.07	0.8899
Ccnd1	11164.8	10661.7	8975.3	10738.6	10880.5	11862.9	0.07	0.9108
Ewsr1	11140.2	11155.5	10526.9	11425.4	10560.2	11275.1	0.00	1.0000
Igf2r	11139.2	10187.1	10130.9	11886.1	11490.4	13545.8	0.24	0.2820
Ghr	11117.9	10618.0	10456.4	10382.9	10831.2	10228.9	-0.02	1.0000
Ssr2	11115.7	13661.7	11676.5	11606.9	12701.0	10344.8	-0.18	0.5643
Hid1	11115.7	11907.1	10019.1	10060.2	9682.3	10337.6	-0.21	0.3907
Fkbp1a	11112.5	10978.8	12128.9	10974.2	12017.1	11036.8	0.03	0.9858
Rnf10	11111.5	11146.4	11305.8	11329.8	11210.1	11440.8	0.02	1.0000
Cep170b	11111.5	10842.1	10157.1	10708.8	10492.4	11374.7	0.01	1.0000
Dcaf8	11106.1	11290.3	11712.8	10775.7	10992.4	10513.3	-0.07	0.8884
Sdf4	11103.0	11147.3	11118.3	9966.8	10573.5	8922.8	-0.17	0.6025
Mib1	11074.2	9914.7	10219.6	10582.5	10347.6	11685.4	0.11	0.8144
Gars	11074.2	11707.6	11103.2	10766.2	11330.3	10532.3	-0.09	0.8494
Gdi2	11063.5	11825.1	11328.9	11292.7	11692.7	11659.1	-0.02	1.0000
Sparcl1	11033.7	10581.6	12172.2	14947.6	15175.5	16918.0	0.50	0.0002
Fam151a	11028.4	10958.7	11203.0	9120.8	9586.8	8837.6	-0.24	0.1957
Picalm	11018.8	10580.6	10681.1	11446.6	11217.3	11816.7	0.11	0.7536
Copb1	11018.8	11145.5	10592.4	11135.6	11241.9	11056.8	0.00	1.0000
Rab3d	11018.8	10950.5	10386.9	10277.8	10956.5	9882.0	-0.07	0.8911
Ddx39b	11014.5	11673.9	10810.0	10852.1	10273.7	10404.6	-0.13	0.6807
Dnajb9	11013.4	10204.4	8910.8	8254.5	8897.9	8054.1	-0.25	0.3134
Slc2a5	11008.1	10867.6	9832.7	9647.3	10190.6	9327.7	-0.14	0.7033
Tmod2	10997.5	9463.7	8401.0	11762.9	10925.7	10975.3	0.21	0.4734
Pappa2	10966.6	17263.0	8561.2	16565.4	13309.8	15756.8	-0.02	1.0000
Dicer1	10943.1	11166.4	10198.5	11620.7	10702.9	11983.4	0.04	0.9742
Rps14	10916.5	11473.4	13177.8	10683.4	11769.7	6703.6	-0.15	0.9148
Sec24c	10914.4	10505.0	9960.7	10778.9	10083.8	10657.3	0.00	1.0000
Tmed7	10912.2	10658.1	10253.9	9792.7	10183.4	9293.2	-0.12	0.8150
Abcd3	10893.0	10656.3	10633.7	10896.7	10884.6	10826.7	0.03	0.9764
Zfr	10854.7	10686.3	10724.4	10416.9	10108.4	10009.7	-0.07	0.9522
Cpd	10848.3	9963.0	9917.3	12439.1	11420.6	12807.6	0.26	0.1701
Selenof	10830.2	11738.6	11187.9	11089.9	11477.1	9415.5	-0.11	0.8314
Luc7l2	10798.2	10646.2	9683.6	11336.2	9692.6	11725.2	0.04	0.9622
Cadps	10793.9	9683.3	10217.6	9814.0	10243.9	9582.2	0.01	1.0000
Tmem215	10789.7	11601.0	11639.3	10827.7	12203.0	9597.6	-0.08	0.9343
Glud1	10782.2	11299.4	12204.5	11097.4	11363.1	9879.3	-0.06	0.9788
Gipr	10767.3	10279.1	9593.9	9677.0	9352.7	8711.7	-0.14	0.6860
Sf1	10764.1	10853.0	10065.5	11160.0	10362.0	11862.0	0.04	0.9839
2310022B05R	10762.0	11221.1	10737.5	9203.6	10275.8	9296.0	-0.20	0.3896
Gbf1	10750.3	10854.0	9452.9	11332.0	10837.4	11637.4	0.06	0.9486
Ddc	10749.2	10400.3	9536.5	10111.2	11083.8	9719.9	-0.01	1.0000
Rpl3-ps1	10734.3	11468.0	12204.5	11264.0	11628.0	10257.9	-0.05	0.9713
Hnrnpul1	10718.3	10641.7	10234.7	11334.1	11289.2	11955.3	0.11	0.7538
Ankrd17	10711.9	10500.5	10072.5	11391.4	10463.7	11942.6	0.09	0.8598
Gm7964	10706.6	10976.9	9906.3	10906.3	11036.6	10807.7	0.00	1.0000
Mapk3	10699.1	10577.9	11435.7	9967.9	10915.4	9133.8	-0.08	0.9483
Cxxc4	10699.1	10294.6	9953.6	9663.2	9917.4	9129.3	-0.10	0.8028
Ubr3	10678.9	10219.0	9252.4	11548.5	10822.0	11861.1	0.15	0.6827
Ppp2r1a	10672.5	10534.2	10804.0	10899.9	10827.1	10676.3	0.03	0.9660
Magt1	10639.4	11468.9	10342.5	11425.4	11503.8	11617.4	0.02	1.0000
Slc7a8	10585.1	10636.2	10318.3	11769.3	11728.6	10580.3	0.09	0.8554
Cdc42bpb	10568.0	10505.0	10079.6	10170.6	9880.5	10987.9	-0.02	0.9896
Pcnx	10563.8	10782.9	9537.5	10619.7	9708.0	11153.7	-0.02	0.9856
Fam102a	10536.1	9769.8	9688.6	9063.4	9343.5	9401.9	-0.08	0.8922
Fus	10490.3	10772.9	10437.2	12507.1	11402.1	14060.3	0.21	0.4658
Maz	10482.8	10679.0	10723.4	10127.1	9857.9	9626.6	-0.10	0.8395
Mafb	10473.2	13026.7	12901.7	13227.9	14136.4	12023.2	0.03	1.0000
Ivns1abp	10457.2	10495.9	10063.4	11953.0	11008.9	11760.6	0.13	0.6983
Cfl1	10445.5	11198.3	12332.4	11454.0	12020.2	11652.8	0.06	0.9897
Zim1	10434.9	9744.3	10490.6	10413.7	9184.3	12115.6	0.09	0.9126
Hnrnpm	10427.4	10065.9	10778.8	10080.4	10495.5	10803.2	0.04	0.9729
Aplp2	10425.3	9880.1	11704.7	11563.4	11915.5	13321.2	0.26	0.2150
Oaz1-ps	10421.0	10317.4	11093.2	10237.5	11162.9	8816.8	-0.04	0.9841
Polr2a	10419.9	10329.2	9550.6	11715.2	11122.8	14022.3	0.23	0.4091
Rps6ka2	10402.9	9594.0	9962.7	8564.5	8530.3	8387.5	-0.17	0.4851
Vamp2	10380.5	10042.2	10604.5	9306.5	9951.3	9775.1	-0.06	0.9503
Akt1	10346.4	11324.0	11299.7	11215.2	10286.0	10496.1	-0.07	0.9189
Tnrc6a	10333.6	10128.8	9230.2	10542.2	9432.8	10943.6	0.03	0.9955

Mxd4	10332.6	10184.3	11254.4	10368.1	10471.9	9635.6	-0.01	1.0000
Adcy6	10323.0	10036.8	9732.0	10260.9	9547.8	10626.5	0.01	0.9951
Pkn1	10299.5	9796.3	9468.0	9536.9	9425.6	10214.4	-0.01	1.0000
H2afy	10287.8	9767.1	10028.2	9143.1	9335.3	8402.9	-0.12	0.7535
Nrd1	10276.1	10822.1	9619.1	10496.5	10363.0	10827.6	-0.02	1.0000
Dennd4c	10261.2	9242.3	8494.7	10027.3	9532.4	9868.4	0.07	0.8910
Tnrc18	10261.2	10754.6	10240.8	10810.7	9189.5	11631.9	-0.02	1.0000
Myo18a	10251.6	10127.9	10100.7	10329.9	10392.8	11622.0	0.08	0.9334
Krt7	10235.6	10166.1	9904.2	8804.4	9120.7	7740.7	-0.22	0.3140
Nid1	10227.1	9805.4	11446.8	14066.5	14054.2	18120.9	0.54	0.0006
Hepacam2	10223.9	9621.3	9981.8	8900.0	10080.7	7168.3	-0.14	0.8031
Abca1	10209.0	8336.8	10056.4	9947.7	9839.4	11059.5	0.25	0.2109
Napa	10201.5	10157.9	9669.5	9136.7	9863.0	8780.6	-0.12	0.7764
4833439L19Ri	10199.4	9136.7	9054.9	9731.1	10222.4	9557.7	0.09	0.8759
Stk32a	10194.0	8779.5	7745.1	8075.1	8565.2	7674.6	-0.11	0.8378
Fkbp4	10168.5	9813.6	9632.2	9987.0	10566.3	9279.6	0.02	1.0000
Ap2b1	10167.4	10116.9	9537.5	10149.4	10265.5	11477.1	0.07	0.9280
Gprasp1	10166.3	9547.5	10066.5	9440.3	9958.5	9728.9	0.01	1.0000
Nav1	10161.0	9498.3	9138.5	11046.4	9122.7	11813.1	0.14	0.7682
Nrep	10159.9	11940.8	13043.8	10688.7	12032.6	11929.0	-0.04	0.9972
Kmt2e	10147.2	9913.8	9785.4	10630.3	9891.8	11464.4	0.10	0.9029
Atp6ap2	10141.8	9278.8	9463.9	9173.8	10581.7	8714.4	0.02	1.0000
Serbp1	10138.6	10167.0	11038.7	10420.1	11043.8	10945.4	0.07	0.8726
Irak1	10133.3	10439.4	9804.5	10644.1	10249.1	10230.7	0.00	1.0000
Dnm2	10118.4	10188.9	9722.9	9535.8	9905.1	9490.7	-0.07	0.9051
Fndc3b	10106.7	10109.6	8907.8	10180.2	9486.2	10950.8	0.02	1.0000
Baz1b	10104.5	10155.2	9208.0	11187.6	9802.4	11345.7	0.09	0.8809
Zswim8	10097.1	9639.6	9353.1	10478.5	9429.7	11026.0	0.09	0.8558
Sepsecs	10093.9	9963.9	9614.1	9769.4	9921.5	9305.0	-0.04	1.0000
Nbea	10088.6	10280.0	9732.0	11770.4	10126.9	12193.5	0.14	0.7075
Ppp1r37	10068.3	10086.9	10607.5	9179.1	9566.3	8892.9	-0.12	0.6983
Fbxo21	10059.8	9452.8	9980.8	8733.3	9590.9	8378.4	-0.09	0.8406
Sdha	10053.4	9710.6	10007.0	10181.2	10779.9	10175.5	0.08	0.8575
Tpd52	10045.9	10501.4	10106.8	9043.3	9279.8	8260.7	-0.22	0.3030
Gm6789	10031.0	10258.1	10863.4	10070.8	10733.7	9019.7	-0.04	0.9731
Map4k4	10011.8	10160.7	10550.1	10765.1	10092.0	11301.3	0.07	0.9158
Grk3	10002.2	9836.3	10095.7	8842.6	8046.7	9776.0	-0.14	0.7873
Kmt2c	9985.2	9737.0	8077.6	10372.3	8147.3	11955.3	0.07	0.9500
Pdcd6ip	9949.0	9513.8	9184.9	9782.1	9772.7	10067.7	0.05	0.9944
Eif3c	9941.5	9906.5	9715.8	10747.0	10716.3	11258.8	0.13	0.6871
Leng8	9940.4	10561.5	9201.0	9291.7	8001.5	9480.7	-0.20	0.5273
Ncstn	9936.2	10433.1	9948.6	9515.7	9589.9	9381.1	-0.12	0.7321
Ash1l	9909.5	9256.0	8532.0	9655.8	8520.0	11173.6	0.07	0.9422
Hcfc1	9906.3	9978.5	8761.7	10563.4	9232.6	11297.7	0.06	0.9267
Ubxn4	9904.2	9857.3	9315.8	8680.2	9210.0	8528.8	-0.15	0.6112
Tardbp	9903.2	10136.1	9181.8	9484.9	8948.2	9979.8	-0.08	0.9377
Rps9	9884.0	10198.9	11028.7	10039.0	10946.2	9300.5	-0.02	1.0000
Stat3	9876.5	9852.7	10218.6	10522.0	10372.3	9520.6	0.03	0.9896
Amy2a2	9876.5	15682.4	19607.0	16156.7	12178.4	18382.7	0.02	1.0000
Pdxdc1	9874.4	9857.3	8884.6	9533.7	9325.0	8986.2	-0.07	0.9522
Eid1	9866.9	9883.7	9835.7	8534.8	10065.3	7828.6	-0.15	0.7368
Klhl7	9863.7	9515.7	10215.6	8820.3	9287.0	8459.9	-0.10	0.8554
Fam91a1	9859.5	9457.4	9129.4	9455.1	9283.9	9267.9	-0.02	1.0000
Meis2	9858.4	10219.0	10295.2	9504.0	9497.5	8575.9	-0.14	0.7470
Gtf3c1	9856.3	9454.6	9145.6	9115.5	8851.7	9963.5	-0.02	1.0000
Lpl	9831.8	11983.6	11771.2	11127.1	11531.5	10111.1	-0.10	0.8598
Pank3	9827.5	9424.6	9510.3	9682.3	9349.6	9597.6	0.01	0.9956
Abca7	9796.6	9873.7	9278.6	8817.2	8055.9	7879.3	-0.23	0.2983
Trim28	9795.5	9563.9	9884.1	10033.7	9753.2	9805.9	0.04	0.9831
Ctbp1	9781.7	10357.4	10335.5	10380.8	10405.1	9194.5	-0.04	0.9731
Entpd3	9779.5	10196.2	9438.8	9206.7	9955.4	9100.3	-0.10	0.8816
Lamp2	9767.8	9502.9	10056.4	9642.0	10494.5	9502.5	0.05	0.9717
Stag2	9762.5	10027.7	9765.2	9497.6	9681.3	9430.9	-0.06	0.9269
Gorasp2	9751.8	10028.6	9298.7	9699.3	9750.1	10016.9	-0.02	1.0000
Ctnna1	9749.7	10119.7	10109.8	10796.9	10997.6	11305.9	0.12	0.7470
Csnk1d	9744.4	9752.5	9970.7	9679.1	9905.1	9661.9	0.00	1.0000
Podxl	9739.1	10000.3	10147.1	11920.0	12350.8	13262.3	0.30	0.0611
Celf1	9736.9	9481.0	9258.4	9433.9	9484.1	9730.7	0.01	1.0000
Myo6	9733.7	8446.1	8433.2	9167.5	9041.6	9467.1	0.10	0.8253
Ppp1ca	9724.1	10557.9	10045.3	9801.2	10456.5	8511.6	-0.11	0.8314
Ywhaz	9713.5	8877.0	8983.3	9224.8	9639.2	9291.4	0.07	0.9593
Epas1	9695.4	8734.9	10276.0	12995.4	13715.4	15479.7	0.60	0.0000
Pde3b	9689.0	8811.4	8887.6	8849.0	8899.9	8693.6	-0.01	1.0000
Hnrnpa0	9684.7	9247.8	10253.9	9717.3	9728.5	9805.0	0.06	0.9379
Txndc5	9682.6	10127.9	9582.8	9457.3	9587.8	9431.8	-0.08	0.8665
Rps5	9673.0	10673.6	11651.3	10073.0	10834.3	10245.2	-0.04	0.9877

Mapk1	9669.8	9175.8	9179.8	9696.1	9592.0	9582.2	0.06	0.9690
Scarb2	9668.7	9055.6	9823.6	9307.6	10212.1	9490.7	0.08	0.8910
Eef1a1-ps1	9667.7	10167.0	10296.2	9261.9	9846.6	8311.4	-0.14	0.7830
Atxn2l	9664.5	9958.4	8513.8	10184.4	9296.2	10469.8	0.02	1.0000
Usp19	9659.1	9476.5	9435.7	9902.1	9269.6	9176.4	-0.01	1.0000
Ctdsp2	9658.1	9947.5	11006.5	10118.6	10426.7	10777.8	0.06	0.9527
Scaf11	9650.6	9659.6	8762.7	10307.6	10172.1	10820.4	0.11	0.7961
Arpc5	9647.4	9473.7	9913.3	9125.0	9566.3	8147.4	-0.08	0.8710
Prss3	9646.4	20167.4	18562.1	18505.9	11993.5	20759.4	0.02	1.0000
Vdr	9626.1	7245.4	6250.9	7491.3	8316.7	7149.3	0.04	1.0000
Dstn	9620.8	10057.7	9498.2	9696.1	10018.1	9036.9	-0.06	0.9831
Ccni	9593.1	9727.9	10710.3	9128.2	10184.4	9369.3	-0.03	0.9731
Sec63	9592.0	9157.6	8609.5	9076.2	8874.3	9122.9	-0.02	1.0000
Ubap2l	9590.9	9615.0	9214.1	10230.1	9371.2	10494.3	0.06	0.9490
Zmynd11	9577.1	9822.7	9081.1	9234.3	8982.1	9350.3	-0.08	0.8762
Trrap	9576.0	9076.5	8007.0	9688.7	7995.3	10531.4	0.05	0.9805
Ufm1	9575.0	9076.5	8138.0	8689.8	9398.9	7729.9	-0.06	0.9557
Supt5	9555.8	9561.2	8708.3	9583.6	9379.4	10227.1	0.03	0.9895
Pecam1	9554.7	9185.9	10150.1	12502.8	12029.5	12843.9	0.40	0.0025
Erbin	9548.3	9616.8	8680.1	9237.5	8396.8	9520.6	-0.07	0.9232
Lrrc58	9538.7	7287.3	8904.8	9056.0	9505.7	9715.3	0.31	0.0492
Slc4a7	9536.6	9348.9	7926.4	11149.4	10452.4	10497.9	0.19	0.5250
Nfic	9525.9	9026.4	9304.8	10316.1	9778.8	12612.0	0.23	0.3766
Rbbp4	9524.9	9647.8	9615.1	9456.2	9487.2	9455.4	-0.02	0.9792
Cpa2	9523.8	17803.2	18245.8	23691.5	15305.8	25810.9	0.22	0.7608
Rpl19-ps11	9507.8	10604.3	10920.9	9674.9	10386.7	8029.7	-0.15	0.7910
Fat1	9505.7	9451.9	7956.6	10851.1	8899.9	12456.2	0.18	0.6967
Ftl1	9505.7	8810.5	9612.1	9704.6	10327.1	9570.4	0.14	0.6681
Pea15a	9497.2	9790.8	10125.9	9286.4	10006.8	8593.1	-0.07	0.9344
Phip	9488.7	9365.3	8565.2	9513.5	8516.9	9967.1	0.00	1.0000
Sympk	9483.3	9626.8	9063.9	9167.5	9189.5	8706.3	-0.08	0.8783
Ucn3	9479.1	8262.1	8972.3	11299.0	13583.0	11007.0	0.45	0.0032
Hipk1	9476.9	8741.3	8415.1	8916.9	8650.4	9591.2	0.04	0.9545
Por	9476.9	8725.8	8451.4	8573.0	9120.7	7638.4	-0.05	0.9681
Cct5	9476.9	10849.4	10414.1	9671.7	10299.4	8707.2	-0.15	0.6881
Akap11	9475.9	9230.5	8304.3	10014.6	9165.8	10226.2	0.09	0.9343
Fxyd6	9464.1	9790.8	10204.5	8329.9	9471.8	8027.0	-0.17	0.6170
Ndst1	9455.6	9712.4	9639.3	9326.7	9123.8	10872.0	0.01	1.0000
Vapb	9436.4	9516.6	9691.7	9455.1	9774.7	9473.5	0.01	1.0000
Sgsm1	9435.4	8029.8	7973.8	7199.4	7500.5	6646.6	-0.17	0.6067
Rpl14-ps1	9432.2	9729.7	8827.2	9999.7	10627.9	10076.7	0.08	0.9370
Usp48	9417.3	9015.5	8105.8	8023.1	7769.5	8988.0	-0.11	0.8366
Herc1	9415.1	9043.7	7998.0	10429.6	8525.2	11472.5	0.15	0.7982
Tpr	9409.8	9479.2	8681.1	10786.3	9634.0	11845.7	0.17	0.6185
Arf4	9408.7	10034.0	9214.1	9230.1	9762.4	8738.9	-0.10	0.8429
Amfr	9405.5	10036.8	10239.8	9914.8	10082.7	8969.9	-0.05	1.0000
Spen	9398.1	8101.7	7639.3	8786.4	7912.2	9933.6	0.10	0.8462
Hist1h1c	9398.1	11980.9	13851.8	7374.5	9712.1	7358.5	-0.34	0.4321
Mki67	9388.5	9563.9	5474.0	9231.2	7577.5	13260.5	0.13	0.9096
Ndrg1	9386.4	9369.9	10337.5	8275.8	9298.3	7238.0	-0.17	0.7112
Lrba	9376.8	9502.9	8807.0	10670.6	9859.9	11309.5	0.15	0.6550
Smg1	9374.6	8689.4	7335.0	9278.9	8246.9	10709.9	0.11	0.8759
Eif4ebp2	9366.1	8771.3	9057.9	9523.1	9644.3	10360.2	0.15	0.6469
Kdm6b	9365.1	7755.6	7954.6	8494.5	7862.9	9476.2	0.11	0.8884
Cacna1d	9358.7	9543.0	8037.2	9148.4	8001.5	9978.9	-0.05	0.9731
Firre	9355.5	9764.4	8281.1	8704.6	7397.8	8342.2	-0.22	0.4158
Hspa4	9350.1	8896.2	8489.6	9690.8	9161.7	9584.9	0.08	0.8470
C1galt1	9349.1	8667.5	8437.2	8327.8	8637.1	7422.8	-0.09	0.8791
Esyt1	9344.8	8394.2	8640.8	7751.4	8114.5	8345.8	-0.06	0.9395
Atf6	9340.5	9092.9	7905.3	8030.6	8220.2	9391.1	-0.07	0.9466
Zfp106	9339.5	9357.1	7968.7	8613.3	8101.1	10256.1	-0.03	1.0000
Gabarap	9326.7	9470.1	9484.1	8975.3	9804.5	8000.7	-0.08	0.9582
Cacna2d1	9317.1	8354.1	8211.6	8902.1	8686.4	8780.6	0.06	0.9503
Pofut2	9315.0	9672.4	9112.3	8831.0	9331.2	8158.3	-0.12	0.8113
Parva	9311.8	8691.2	9745.1	9027.3	9275.7	10672.7	0.12	0.7861
Slc38a4	9305.4	9903.8	8632.7	10927.5	11260.4	9330.4	0.10	0.8429
Nipal3	9278.7	9950.2	10050.3	8247.1	8350.6	8315.0	-0.24	0.2295
Ctsl	9273.4	8867.0	10651.8	9021.0	9605.3	8706.3	0.01	1.0000
Bclaf1	9268.1	8879.8	8878.6	9346.9	8782.9	9440.9	0.04	0.9679
Smarca2	9242.5	8767.7	8558.2	9408.4	8563.1	9719.9	0.07	0.9096
Gnaq	9234.0	9014.6	9376.3	9546.4	9589.9	9767.0	0.09	0.8511
Akap9	9228.7	9189.5	8226.7	9795.9	8449.2	10986.1	0.09	0.9175
Eif3b	9222.3	9324.3	8432.2	9481.7	9593.0	10160.1	0.07	0.9289
Gch1	9216.9	8820.5	8372.8	7483.9	8243.8	6646.6	-0.22	0.4180
Ireb2	9214.8	8632.0	8558.2	8827.8	8790.1	8710.8	0.02	1.0000
Supt6	9211.6	8529.0	8597.4	10092.1	9389.7	10574.0	0.21	0.3900

Gm8730	9210.5	9840.9	10236.7	9764.1	10395.9	8973.5	-0.02	1.0000
Cand1	9202.0	8707.6	8438.3	9717.3	9500.6	9950.8	0.15	0.6130
Hspa9	9201.0	9399.0	9129.4	9543.3	9425.6	9160.1	0.00	1.0000
Zdbf2	9197.8	8821.5	7627.2	9250.3	7913.2	9890.1	0.04	1.0000
Fryl	9180.7	9642.3	7840.8	9130.3	7579.5	10055.9	-0.07	0.9884
Ppp2ca	9175.4	8786.8	9157.6	9602.7	9140.2	8153.8	0.02	1.0000
Tuba1a	9174.3	9731.6	11769.2	8900.0	8753.1	8799.6	-0.14	0.7192
Utrn	9160.5	8727.6	8141.0	10563.4	9562.2	12130.1	0.27	0.2597
Rph3al	9148.7	9265.1	9067.0	7528.5	8226.4	7138.4	-0.25	0.2054
Srsf11	9147.7	9170.4	8087.6	8900.0	7879.3	9543.2	-0.04	0.9557
Impad1	9141.3	8639.2	8945.1	8896.8	9043.7	8865.7	0.04	0.9715
Srsf2	9134.9	9400.9	8924.9	9613.3	8544.7	9376.6	-0.03	1.0000
Msi2	9123.2	8050.7	8365.7	8430.8	8146.3	8189.1	0.02	0.9858
Muc4	9117.8	8191.0	6581.3	8494.5	5939.8	8016.1	-0.07	0.9553
Prepl	9111.5	8420.6	7963.7	8159.0	8115.5	7829.5	-0.06	0.9422
Pbrm1	9104.0	9106.6	8296.2	9815.0	9275.7	10423.6	0.11	0.7812
Baz2a	9067.8	8501.7	8364.7	9421.2	8407.1	9589.4	0.09	0.8773
Tbl1x	9060.3	8622.9	7877.0	8766.2	8678.1	8440.9	0.00	1.0000
Gm9794	9053.9	10379.3	11297.7	9741.8	10792.2	10362.0	-0.01	1.0000
Trio	9052.8	8765.0	7601.0	9357.5	8377.3	10680.9	0.11	0.8515
Smim14	9052.8	9092.9	8411.1	8069.8	7878.3	7632.0	-0.19	0.4786
Tulp4	9048.6	8592.8	7556.6	8522.1	7899.9	9311.3	0.01	1.0000
Plekha6	9044.3	8093.5	8348.6	8249.2	7459.4	8719.9	-0.01	1.0000
Gck	9041.1	8921.7	9256.4	7457.3	7290.0	6932.8	-0.29	0.0693
Kcnj11	9033.7	9589.4	9211.0	7948.8	8749.0	7443.6	-0.22	0.3362
Mtss2	9031.5	7467.7	7703.7	7702.5	8100.1	7852.1	0.05	0.9603
Nr3c1	9025.1	8396.9	8534.0	9238.6	9227.5	9227.1	0.12	0.7053
Ldb1	9020.9	8848.8	9632.2	8384.1	8554.9	7583.1	-0.11	0.8000
Nipbl	9013.4	8572.7	8168.2	9216.3	8421.5	10334.0	0.11	0.8455
Os9	9003.8	9676.9	8937.0	9830.9	10375.4	9484.4	0.04	0.9778
Epm2aip1	9003.8	8079.0	7865.0	7962.6	7434.7	8129.3	-0.05	0.9500
Azin1	8992.1	8458.0	7427.7	8940.3	8895.8	8805.9	0.07	0.9382
Dctn1	8987.8	8718.5	8722.4	9084.7	9236.7	9514.2	0.08	0.8775
Prrc1	8980.4	8481.6	7694.7	8182.4	8293.1	8411.0	-0.03	0.9810
Ptov1	8978.3	9307.0	9705.8	8940.3	8958.4	7969.9	-0.10	0.8445
Idh2	8971.9	9297.0	9462.9	8926.5	9896.9	8714.4	-0.02	1.0000
Epcam	8961.2	9274.2	9945.6	8522.1	8822.9	7928.2	-0.13	0.7420
Neo1	8960.1	9382.6	8829.2	9912.7	9456.4	10281.4	0.08	0.8833
Celsr3	8956.9	8383.3	6833.2	8103.8	6053.8	8033.3	-0.13	0.8316
Gm10123	8951.6	10420.3	9933.5	9841.6	10024.2	8603.9	-0.10	0.8148
Rab37	8941.0	8722.2	9485.1	6855.4	7091.8	5967.2	-0.37	0.0274
Rmnd5a	8937.8	8275.8	9203.0	8517.8	8768.5	7960.8	0.01	1.0000
Cmip	8918.6	8877.9	8936.0	9005.1	8935.9	9475.3	0.04	0.9820
Scnn1b	8916.5	7340.1	7780.3	7218.5	7664.7	7005.3	-0.03	1.0000
Tspan3	8915.4	8990.0	9491.1	8534.8	9455.4	7568.6	-0.07	0.9256
Lin7c	8911.1	7848.5	8242.8	8067.7	8412.2	7904.7	0.03	0.9932
Slc25a4	8894.1	9301.6	9911.3	9383.0	9692.6	8440.0	-0.02	1.0000
Anxa6	8881.3	8770.4	8682.1	8371.3	8350.6	9191.8	-0.02	1.0000
Cenpb	8878.1	9332.5	8967.2	9015.7	9296.2	8729.8	-0.04	0.9785
Ip6k1	8878.1	8977.2	9712.8	8637.8	9037.5	8407.4	-0.05	0.9998
Ybx1	8873.8	9187.7	9739.0	10283.2	9079.6	7896.5	-0.02	0.9948
Ubtf	8873.8	8534.5	9215.1	8281.1	8506.7	8235.3	-0.04	0.9522
Cope	8852.5	10223.5	9269.5	9063.4	9357.9	7155.6	-0.20	0.5181
Pde4dip	8844.0	7633.5	6774.8	8870.2	8152.4	8735.3	0.14	0.7636
Actr2	8839.7	8035.2	8603.5	8535.9	8919.4	8578.6	0.09	0.8461
Sec23b	8837.6	9181.3	7567.7	7832.1	8029.2	8268.8	-0.15	0.6665
Atxn7l3b	8828.0	8356.8	9034.7	8229.1	8737.7	7817.7	-0.03	1.0000
Eprs	8827.0	8940.8	8322.4	10232.2	10194.7	10679.1	0.20	0.3320
Rdx	8824.8	8335.0	8338.5	9127.1	9165.8	10228.0	0.17	0.5639
Ern1	8821.6	8619.2	7716.8	8507.2	8671.0	8746.1	0.01	1.0000
Rab14	8820.6	8024.3	8090.6	8739.7	8795.2	8488.0	0.10	0.8440
Gm4366	8817.4	9366.2	9447.8	9583.6	10104.3	8974.4	0.03	1.0000
Cdk16	8804.6	8736.7	8164.2	7971.1	7861.9	7424.6	-0.15	0.6661
Gm15427	8796.1	9347.1	11350.1	9259.8	9759.3	8857.6	-0.02	1.0000
Itga6	8795.0	7770.1	8320.4	8530.5	7684.2	9584.0	0.11	0.8429
Abr	8788.6	8540.9	7886.1	8529.5	8448.1	8498.9	0.00	1.0000
Fem1b	8779.0	8143.7	8244.8	8654.7	9056.0	8871.1	0.11	0.7675
Glyr1	8763.0	8699.4	8390.9	8033.7	8177.1	7870.3	-0.11	0.8396
Npepl1	8747.0	9298.8	8675.0	7959.4	7877.3	7108.5	-0.25	0.2313
Usp7	8740.6	8943.5	8450.3	9170.7	8276.7	8943.6	-0.02	0.9931
Gnai3	8735.3	8620.1	8460.4	8738.6	8913.3	8137.5	0.00	1.0000
Hip1r	8727.9	9803.5	8777.8	7891.5	7194.5	7181.9	-0.35	0.0139
Kdm1a	8724.7	8484.4	8535.0	8977.5	9253.1	8425.5	0.06	0.9152
Cct7	8724.7	9432.8	9035.7	9308.7	9469.8	8434.6	-0.04	0.9810
Fam20b	8716.1	8398.7	8672.0	8479.6	8498.5	7933.7	-0.02	1.0000
Thrap3	8710.8	8902.5	8409.0	9507.2	8985.1	10242.5	0.10	0.8316

Hook1	8709.7	8603.7	7280.6	8642.0	7853.7	8358.5	-0.03	0.9966
Lpgat1	8703.3	7345.6	7370.2	7373.5	7590.8	7786.0	0.03	1.0000
Srpk2	8697.0	8452.5	9196.9	8046.5	7868.0	8281.5	-0.07	0.9126
Psme4	8695.9	8607.4	7218.1	8863.9	8197.6	8778.8	0.02	1.0000
Specc1l	8687.4	8978.2	8312.3	9571.9	9330.1	9793.2	0.09	0.8822
Ecpas	8667.1	8469.8	7896.2	8654.7	8201.7	9023.3	0.03	1.0000
Trak1	8662.9	8676.6	7528.4	9001.9	8448.1	9661.0	0.07	0.9468
Top2b	8662.9	8722.2	8973.3	8938.2	8405.0	8844.9	0.00	1.0000
Arhgap35	8659.7	8306.7	8444.3	8517.8	8464.6	9560.4	0.08	0.9456
Dgkd	8658.6	9092.9	8675.0	7751.4	7800.3	8043.3	-0.19	0.3583
Atp8b2	8636.2	8806.9	7586.9	6961.6	6919.3	7095.8	-0.29	0.0907
Cdc42bpa	8619.2	8192.8	8122.9	8895.7	8239.7	9420.9	0.10	0.8415
Mast3	8617.0	8456.1	7840.8	7418.1	7374.2	7678.2	-0.16	0.6042
Jak1	8607.5	8348.6	8666.0	9428.6	9396.9	9454.5	0.16	0.5345
Scd2	8606.4	9523.9	8509.8	10610.1	9485.2	11223.4	0.14	0.7648
Ampd2	8606.4	8721.2	7506.3	7767.3	7428.6	7484.4	-0.17	0.5170
Scaf1	8603.2	8178.3	8125.9	8585.7	8211.0	8633.8	0.04	0.9471
B230219D22R	8593.6	8438.8	7714.8	8325.7	8154.5	8124.8	-0.03	1.0000
Abca3	8587.2	8079.9	7392.4	8125.0	7476.8	8064.1	-0.03	1.0000
Ppp6r3	8586.1	8391.5	8708.3	8563.5	8004.6	8671.9	0.00	1.0000
Pip5k1c	8574.4	8406.9	8674.0	8398.9	8088.8	8412.8	-0.02	0.9850
Maged2	8562.7	8872.5	8304.3	8005.1	8486.1	7193.7	-0.14	0.6967
Tax1bp1	8560.6	8270.3	8393.9	8044.4	8509.8	7930.0	-0.02	1.0000
Kif21a	8558.4	8488.9	9048.8	9280.0	9124.8	8897.4	0.09	0.9035
Trabd	8549.9	8898.0	7449.8	7085.8	6763.2	6919.2	-0.31	0.0601
Tagln2	8538.2	8474.4	7993.9	7793.8	8038.5	7673.7	-0.10	0.7865
Gosr2	8531.8	8338.6	7647.3	7765.2	8160.7	7675.5	-0.07	0.8848
Mef2d	8530.7	7732.8	7404.5	8024.2	7773.6	8358.5	0.05	0.9483
Ivd	8521.1	8742.2	7790.4	7260.9	6991.2	7377.5	-0.25	0.1794
Flt1	8516.9	8093.5	7837.8	11115.4	11023.3	11885.6	0.45	0.0005
Msl1	8515.8	8073.5	8582.3	8039.1	7820.8	7929.1	-0.03	0.9850
Rundc3a	8513.7	8645.6	8775.8	7661.1	7902.9	6408.3	-0.21	0.4525
Tor1aip2	8511.6	7958.7	7822.6	7573.0	7727.4	7744.4	-0.05	0.9521
Snrnp200	8508.4	8682.1	7837.8	10088.9	9371.2	10373.8	0.19	0.5100
Psmd2	8500.9	8824.2	8470.5	9587.8	9554.0	9227.1	0.10	0.7841
Cic	8495.6	8828.7	8258.9	8294.9	8004.6	9723.5	-0.01	1.0000
Plxnb1	8493.4	7885.8	7660.4	7430.8	6873.1	8197.2	-0.07	0.9323
Myo10	8481.7	8271.2	7933.5	9074.1	8239.7	10065.0	0.13	0.8207
Rbm5	8478.5	8038.9	8129.9	8438.2	7479.9	7997.1	-0.02	1.0000
Pla2g2f	8474.3	7879.5	8244.8	7739.7	7579.5	7015.2	-0.08	0.8710
Kctd12b	8474.3	8482.6	8203.5	6998.7	7748.9	7120.3	-0.20	0.3690
Araf	8470.0	8184.6	7443.8	7888.3	7485.0	7444.6	-0.09	0.8702
Dgkz	8464.7	7874.0	8090.6	7306.6	6764.3	7249.8	-0.14	0.6196
Osbp	8449.8	8036.2	7506.3	8622.9	8337.3	8651.9	0.08	0.8591
Ago2	8444.4	7286.4	6677.1	7751.4	6694.4	9110.3	0.08	0.9715
Trp53bp1	8421.0	7883.1	7185.9	8340.5	7722.2	8958.1	0.07	0.8949
Rnpepl1	8406.1	7792.9	8192.4	7772.6	7579.5	7576.8	-0.04	1.0000
Igf1r	8402.9	7126.9	8140.0	8833.1	9072.4	10236.1	0.33	0.0399
Glb1l2	8400.7	8554.5	7483.1	6072.0	6714.0	4918.3	-0.44	0.0127
Hba-a2	8396.5	7587.0	9938.5	5677.1	6373.1	6182.8	-0.32	0.0875
Usp33	8390.1	7946.0	7700.7	7721.7	7462.5	7095.8	-0.09	0.8614
Gsk3b	8385.8	7100.5	8132.0	8424.4	8179.1	8901.0	0.22	0.3305
Fam135a	8385.8	8463.4	7662.4	8798.1	8178.1	8407.4	0.01	1.0000
Abat	8383.7	7772.9	8043.3	7533.8	8120.6	7578.6	-0.01	1.0000
Rock1	8378.4	8676.6	8609.5	8998.7	8823.9	8554.1	0.02	1.0000
Lamb1	8376.2	7965.1	8499.7	11752.3	11295.3	14060.3	0.55	0.0001
Rad23b	8367.7	8627.4	8221.6	8346.9	8746.9	8421.0	-0.01	1.0000
Dap	8364.5	8642.0	8308.3	8378.7	8575.5	8159.2	-0.04	0.9841
Nsd3	8363.4	7801.1	7025.7	8300.2	7189.3	8473.5	0.03	0.9925
Hnrnpdl	8361.3	8335.0	7292.7	9137.7	8696.6	9649.2	0.14	0.7332
Vcp	8361.3	8671.1	7823.6	8225.9	8183.2	8709.0	-0.04	0.9884
Ep300	8357.0	8179.2	7778.3	9519.9	8502.6	10312.2	0.19	0.5012
Gaa	8354.9	7636.2	8569.2	6925.5	7312.6	6976.3	-0.12	0.7503
Clic4	8352.8	8294.9	8502.7	9507.2	9030.3	9465.3	0.16	0.6196
Jund	8350.7	7324.6	8981.3	13182.2	11629.0	10671.8	0.58	0.0000
Kras	8340.0	7598.9	7193.9	8150.5	8737.7	7716.3	0.10	0.8649
Ppp6r1	8338.9	8140.9	8379.8	7684.5	7545.6	7243.5	-0.12	0.8031
Rac1	8330.4	8130.0	8528.9	8758.8	8640.2	8262.5	0.06	0.9334
Sec62	8327.2	8012.5	8667.0	8401.0	9325.0	8073.2	0.08	0.8631
Chd7	8320.8	7872.2	7297.7	7856.5	7393.7	8190.0	-0.01	1.0000
Sorbs2	8303.8	8148.2	7717.9	8979.6	8261.3	9240.7	0.11	0.8113
Add1	8298.4	8482.6	8739.5	8337.3	8448.1	8573.1	-0.01	1.0000
Plekha1	8294.2	8155.5	7719.9	7315.1	7052.8	7525.2	-0.15	0.6171
Zfp704	8292.1	8097.2	7668.5	8497.6	7798.2	9611.2	0.09	0.8946
Cd81	8287.8	8792.3	9493.2	8067.7	9308.6	8457.2	-0.03	0.9785
Pkp4	8265.4	8596.4	8426.2	8583.6	8797.2	8892.0	0.03	0.9956

Ppm1l	8260.1	8312.2	7696.7	8514.6	7981.0	8786.0	0.02	1.0000
Aak1	8258.0	8463.4	7090.1	9325.6	8069.3	11283.2	0.18	0.6613
Pgrmc1	8247.3	8099.9	8671.0	6756.7	7871.1	5853.1	-0.23	0.4658
Tle3	8237.7	7911.3	8440.3	7563.5	7545.6	7641.1	-0.06	0.9263
Pdpk1	8229.2	7695.4	7445.8	8012.5	7612.4	8247.1	0.04	0.9541
Cd93	8223.9	7262.7	7438.8	11100.5	10276.8	12529.6	0.55	0.0001
Ssr4	8220.7	9162.2	7633.2	7857.5	8456.4	5399.3	-0.24	0.5238
Dnajc5	8216.4	8414.2	7914.3	7869.2	7728.4	8324.1	-0.07	0.9326
Irf2bp2	8211.1	7161.6	8498.7	7853.3	8022.0	6500.7	0.02	1.0000
Ap1b1	8210.0	7880.4	7685.6	7914.9	8080.6	7881.1	0.01	1.0000
Braf	8208.9	7793.8	7438.8	9128.2	8073.4	9509.7	0.17	0.6082
Srrm1	8198.3	8705.8	7672.5	9016.7	8541.6	10389.2	0.11	0.8399
Alkbh5	8196.2	7251.8	7035.7	7610.2	7599.0	7850.3	0.07	0.9278
Srrt	8181.2	8872.5	7468.0	8091.1	7540.5	8039.6	-0.14	0.6741
Nf2	8177.0	8597.3	8092.7	7679.2	7842.4	7929.1	-0.12	0.7033
Chd8	8174.8	8167.3	7609.0	8407.4	7602.1	9453.6	0.06	0.9649
Prss1	8172.7	16825.7	16755.6	19465.5	11189.6	20207.8	0.10	0.9556
Hdac5	8165.3	6690.6	7237.3	5664.4	6668.8	5019.8	-0.22	0.5153
Atxn10	8137.5	8471.6	8542.0	8588.9	9026.2	8543.3	0.04	0.9805
Tmed3	8133.3	9111.2	7768.2	8547.5	9157.6	6892.0	-0.10	0.9244
Mbd6	8132.2	7815.7	7919.4	7930.8	7431.7	7796.0	-0.02	1.0000
Actr3	8132.2	7626.2	8126.9	7390.5	7713.0	6536.1	-0.08	0.8618
St18	8131.2	8022.5	7083.1	8440.3	8424.5	7444.6	0.02	0.9858
Rbbp7	8125.8	8265.7	8278.1	7950.9	8418.4	8498.9	0.01	1.0000
Cap1	8123.7	7708.2	8038.3	7733.3	8342.4	7274.3	0.00	1.0000
Naga	8123.7	7516.0	7742.0	6462.7	7302.3	6182.8	-0.17	0.6100
Ostc	8122.6	8585.5	7416.6	7113.4	7650.4	6194.6	-0.24	0.3532
Rapgef4	8112.0	7650.8	7276.5	8255.6	8435.8	7886.6	0.09	0.8714
Fkbp8	8110.9	8627.4	8692.2	7798.1	7897.8	6841.3	-0.18	0.5669
Mtcl1	8108.8	7651.7	7114.3	7814.0	7372.1	7970.8	0.01	1.0000
Atp6ap1	8105.6	8254.8	8261.9	8149.5	8778.8	7810.5	0.00	1.0000
Pcm1	8104.5	7885.8	7308.8	8518.9	7542.5	8782.4	0.07	0.9717
Kdm5c	8099.2	6507.5	7617.1	6921.2	6523.0	7111.2	0.04	1.0000
Nfasc	8094.9	8319.5	7987.9	8029.5	7617.5	8270.6	-0.05	0.9325
Phf2	8075.7	7877.6	7902.2	7529.5	7526.1	7969.0	-0.04	0.9562
Ddx50	8074.7	7823.9	6832.2	7472.2	7067.2	7143.8	-0.09	0.8378
Deptor	8066.2	7634.4	8005.0	7940.3	7796.2	8014.3	0.04	1.0000
Gls	8065.1	7598.9	8275.0	8307.6	7890.6	7396.5	0.03	0.9931
Wfs1	8063.0	7971.5	7265.5	7092.2	7476.8	7367.6	-0.11	0.7790
Golph3	8051.2	7169.8	6874.5	7091.1	7768.4	6998.9	0.01	1.0000
Srp68	8038.5	7461.3	7234.2	7683.4	7664.7	7373.0	0.02	0.9931
Tspan13	8028.9	8090.8	7969.7	8695.1	9181.3	7283.3	0.05	0.9805
Kdelr2	8028.9	7837.5	7126.4	7302.3	7770.5	7440.0	-0.05	0.9369
H13	8026.7	8237.5	7477.0	7397.9	7120.6	7249.8	-0.16	0.5716
Pafah1b2	8024.6	7343.8	7935.5	7630.4	7883.4	7347.6	0.04	0.9560
Hectd4	8020.3	7303.7	6763.7	7731.2	6250.9	8594.9	0.03	1.0000
Aacs	8018.2	8287.6	8081.6	6776.9	7078.5	6378.5	-0.27	0.1208
Rab6a	8015.0	6752.5	6714.3	7441.4	8130.9	7123.9	0.13	0.7795
Vat1l	8013.9	8382.3	8878.6	8061.3	8489.2	7766.1	-0.05	0.9495
Krt8	7987.3	8106.3	8082.6	7206.8	7551.8	6376.6	-0.18	0.5100
Rnase1	7986.2	15277.9	15301.7	17028.2	10658.8	16850.1	0.07	0.9817
H3f3a	7974.5	8319.5	8988.4	7379.8	8256.1	6572.3	-0.15	0.7648
Fam129b	7960.7	7894.9	8305.3	7430.8	7969.7	7574.1	-0.05	1.0000
Rev3l	7953.2	7601.6	6776.8	7834.2	6792.0	8412.8	0.02	1.0000
Ilrun	7948.9	8125.4	8148.1	7533.8	7595.9	7160.1	-0.12	0.7494
Gm6472	7947.9	7906.8	9414.6	7444.6	7979.9	6578.6	-0.12	0.8240
Setd2	7941.5	7629.8	7688.6	8252.4	7205.8	9141.1	0.09	0.9093
Sept7	7938.3	7945.0	8175.3	8118.7	8476.9	8452.7	0.06	0.9304
Ube3c	7935.1	7229.0	7205.0	7509.3	7854.7	7643.8	0.07	0.8830
Mia2	7931.9	7549.7	7487.1	7501.9	7626.7	7210.0	-0.02	0.9850
Senp6	7929.8	7966.9	7717.9	7939.3	8067.2	7606.7	-0.01	1.0000
Cdh5	7923.4	7705.4	8349.6	10377.6	10291.2	11372.0	0.42	0.0015
Txlna	7923.4	8187.4	7139.5	7915.9	7537.4	8348.5	-0.03	1.0000
Hmgn3	7921.2	7047.7	7579.8	6458.4	7138.0	5744.4	-0.13	0.7682
Zbtb7a	7914.8	7277.3	7362.2	7442.5	7654.5	7412.8	0.03	0.9896
4932438A13R	7913.8	7413.9	6171.3	7877.7	6975.8	9148.3	0.11	0.8826
G3bp2	7906.3	6928.3	7109.3	7679.2	7771.5	7786.9	0.14	0.7531
Pycr2	7903.1	7388.4	7138.5	6712.1	7180.1	5249.9	-0.19	0.6936
Usp47	7902.1	8089.0	7323.9	7950.9	7724.3	8144.7	-0.02	1.0000
Zfp664	7892.5	7849.4	7903.2	7636.7	7837.2	7453.6	-0.04	0.9778
Chd9	7886.1	7544.2	6785.9	7579.4	6934.7	8134.7	0.01	1.0000
Gga2	7882.9	8140.9	8160.2	7644.2	8097.0	7563.2	-0.06	0.9433
Ccnl2	7877.6	7996.1	7207.0	7511.5	6217.0	6853.1	-0.19	0.5487
Nlgn2	7868.0	8197.4	8058.4	7797.0	7507.6	7803.2	-0.08	0.8748
Slc29a1	7862.6	7857.6	7796.4	7173.9	7691.4	6926.4	-0.10	0.8085
Eng	7855.2	8499.9	8394.9	9975.3	9645.3	11102.1	0.25	0.2494

Gm6560	7854.1	8440.6	8207.5	8024.2	8372.2	7126.6	-0.09	0.8777
Hspa13	7853.0	7842.1	6143.0	6414.9	6389.5	6719.9	-0.21	0.4776
Mapre2	7849.9	7778.3	7800.5	7991.3	7900.9	7922.8	0.03	0.9931
Gm13509	7844.5	8176.4	7945.6	7360.7	7739.7	7362.1	-0.11	0.7770
Clcn3	7834.9	7239.9	6913.8	7258.8	6952.2	7028.8	-0.03	0.9881
Prpf19	7833.9	7753.7	7389.4	7318.3	7554.9	7177.3	-0.07	0.9232
Kcnh2	7832.8	7880.4	7281.6	6742.9	6864.9	6479.9	-0.21	0.3320
Nono	7829.6	8079.0	8087.6	8253.5	7989.2	8238.9	0.02	1.0000
Peg10	7827.5	8241.1	7937.5	8402.1	7465.5	8854.8	0.01	1.0000
Notch3	7825.3	7533.3	7043.8	10182.3	9575.5	14256.0	0.49	0.0082
Hjurp	7824.3	5888.0	4546.1	8027.4	6569.2	9151.0	0.23	0.7321
Atp6v1e1	7815.8	7727.3	8215.6	7076.2	7883.4	6021.6	-0.14	0.7864
Mapk8ip2	7814.7	7634.4	8037.2	7230.2	7511.7	7325.9	-0.06	0.9498
Pkdcc	7812.6	7820.2	8104.8	6750.3	6733.5	5181.0	-0.29	0.2227
Rbm25	7806.2	7516.0	7362.2	8645.2	7680.1	8802.3	0.14	0.7581
Dnajc10	7794.4	8253.0	7128.4	8078.3	7721.2	7918.3	-0.04	0.9789
2900026A02R	7793.4	7617.1	7574.8	8349.0	7597.0	9863.0	0.15	0.7304
Srebf2	7790.2	9215.0	8227.7	9718.4	8583.7	8648.3	-0.01	1.0000
Prpf4b	7760.3	7670.8	6943.0	7852.2	7016.8	8158.3	0.01	1.0000
Smap2	7755.0	8085.3	7689.6	7918.0	7942.0	7424.6	-0.05	0.9559
Dpp9	7752.9	7449.5	7072.0	6615.5	6226.2	6568.7	-0.19	0.4658
Arl1	7734.8	7756.5	7423.6	6765.2	7716.1	5910.2	-0.16	0.6955
Ep400	7728.4	7770.1	6550.1	8720.6	7126.7	9838.5	0.14	0.7910
Preb	7708.1	7535.1	6302.2	6763.1	6404.9	6845.8	-0.14	0.7811
Rps6-ps4	7701.7	8194.7	8579.3	8289.6	8885.5	7291.5	0.00	1.0000
Pcdh1	7694.3	6771.6	6671.0	7331.0	6918.3	7725.3	0.09	0.8719
Agrn	7691.1	8260.3	8574.3	9138.8	8652.5	11666.4	0.22	0.5100
Gramd1a	7687.9	7189.8	7550.6	8072.0	7905.0	7713.6	0.12	0.7192
Mlf2	7683.6	7362.9	7708.8	7080.5	7784.9	7139.3	-0.01	1.0000
Pnmal1	7683.6	7882.2	7159.7	7517.8	7622.6	6914.7	-0.08	0.8937
Ski	7681.5	7351.1	7926.4	8109.1	7908.1	8943.6	0.15	0.6415
Mtpn	7680.4	6795.3	7543.5	7299.2	7348.5	7275.2	0.08	0.8746
Serpinh1	7679.4	7972.4	8479.6	8752.4	8701.8	9738.9	0.17	0.6049
Rps16-ps2	7679.4	8860.6	9770.2	8711.0	9397.9	8941.8	0.03	1.0000
Vps37a	7671.9	7423.0	7332.0	6981.8	7055.9	7440.0	-0.05	0.9642
Ap2a1	7670.8	7567.9	7531.5	7124.0	7424.5	7480.8	-0.04	0.9515
Rrp1	7669.8	6951.1	7539.5	7213.2	7331.0	6946.4	0.03	1.0000
Mdh2	7666.6	7968.7	7489.1	7737.6	8070.3	7535.1	-0.02	0.9810
Tmem229b	7657.0	7715.5	7485.1	8543.3	9682.3	8776.0	0.21	0.3720
Cul7	7650.6	7783.8	7189.9	7255.6	6925.5	7187.3	-0.11	0.7873
Kmt2b	7648.5	7057.7	6283.1	7415.9	6626.7	8175.5	0.06	0.9572
Maco1	7642.1	7682.7	7673.5	7650.5	7886.5	7495.3	0.00	1.0000
Cox4i1	7641.0	8114.5	8682.1	7751.4	8490.2	7188.2	-0.05	1.0000
Snap25	7636.7	7491.4	7753.1	7390.5	6927.5	7740.7	-0.03	1.0000
Arhgap5	7629.3	7288.2	7483.1	7694.1	7417.3	7563.2	0.04	0.9729
Trip11	7628.2	7140.6	6045.3	7699.4	6953.2	8072.3	0.08	0.9525
Safb	7618.6	7257.2	6517.9	7441.4	6649.3	7339.5	-0.02	1.0000
Spag9	7611.2	7261.8	7268.5	7941.4	6868.0	8378.4	0.08	0.9590
Yme1l1	7611.2	7390.2	7374.3	7001.9	7401.9	6641.1	-0.07	0.9227
Ppm1b	7611.2	6956.6	6699.2	6597.5	6756.1	5851.3	-0.11	0.8144
Man2a2	7608.0	7188.0	7064.0	6623.0	5972.6	6665.6	-0.15	0.6478
Llgl2	7605.8	7705.4	7891.2	6077.3	5977.8	5701.8	-0.35	0.0110
Baz2b	7604.8	6511.1	6401.0	7578.3	7125.7	7824.1	0.17	0.5780
Bex3	7604.8	7756.5	7435.7	7488.1	8233.6	6498.9	-0.05	1.0000
Slc8a1	7603.7	7007.6	6694.2	8867.1	7627.8	9337.6	0.26	0.2775
Gm5771	7602.6	15767.1	16029.2	15906.1	9992.4	19377.2	0.06	0.9884
Cct2	7601.6	7463.1	7788.4	7780.0	7619.6	7387.5	0.02	1.0000
Cdh2	7598.4	6789.9	7691.7	6960.5	7237.6	6517.0	0.00	1.0000
Atp2b4	7590.9	6730.7	6522.9	7405.3	7092.8	8181.8	0.14	0.7374
Hmgcr	7590.9	8523.5	7568.7	9185.5	8395.8	8116.6	0.03	0.9986
Uck2	7590.9	7891.3	7340.0	7474.3	6830.0	6974.5	-0.13	0.7033
Sun1	7588.8	7632.6	7398.5	7267.3	6842.3	6849.5	-0.12	0.7619
Fubp1	7566.4	7665.4	6938.0	7414.9	6697.5	7447.3	-0.08	0.8816
Eml5	7563.2	7958.7	6729.4	8113.4	6019.9	8345.8	-0.05	0.9896
Ppp1r1a	7555.8	7804.8	6800.0	7041.2	7559.0	6300.6	-0.13	0.8198
Actr1b	7547.2	7341.0	7190.9	7342.7	7524.1	6851.3	-0.02	1.0000
Jup	7543.0	7319.2	7247.3	8113.4	7955.3	7899.2	0.12	0.7984
Fam234a	7543.0	7024.0	7006.5	5892.6	6886.5	5714.5	-0.18	0.6128
Adra2a	7541.9	6841.8	7297.7	7241.8	7170.9	5261.6	-0.07	0.9498
Rfx6	7536.6	7909.5	7989.9	7644.2	6551.7	6573.2	-0.17	0.6112
Apc	7534.4	7119.7	6315.3	8073.0	6850.5	9065.9	0.15	0.7977
Casc4	7533.4	7051.3	7031.7	7184.5	7391.6	6888.4	0.01	1.0000
Inpp4a	7532.3	7217.1	7468.0	7091.1	6638.0	7277.9	-0.05	0.9457
Ppp2r5c	7516.3	7809.3	7984.9	7911.7	8136.0	7676.4	0.02	1.0000
Tollip	7512.1	7271.8	7381.3	6953.1	7224.3	6524.3	-0.07	0.9503
Ubn2	7509.9	6815.4	6015.1	7189.8	5913.1	8419.2	0.06	0.9579

Trpc4ap	7503.5	7559.7	7378.3	7137.8	7284.8	6842.2	-0.08	0.8759
D5Ertd579e	7502.5	5913.5	5988.9	5297.1	5228.2	5198.2	-0.18	0.5937
Mdh1	7501.4	7730.0	7440.8	7482.8	7973.8	6930.1	-0.04	1.0000
Arfgef2	7500.4	7386.6	6712.3	7494.5	7121.6	8003.4	0.03	0.9877
Pi4ka	7496.1	7607.1	6766.7	8429.7	8296.2	8606.7	0.15	0.6912
Clock	7495.0	6911.0	6068.5	7545.4	6816.6	7598.5	0.08	0.9280
Ppp1r9b	7491.8	7017.6	7658.4	7512.5	7630.8	7860.3	0.11	0.7873
Golga3	7488.6	6650.5	5789.4	7143.1	6522.0	7418.3	0.07	0.9810
Actg1	7474.8	6595.8	8080.6	7888.3	8194.5	8420.1	0.26	0.1766
Tspan5	7459.9	7005.8	8574.3	7111.3	7713.0	6364.9	-0.02	1.0000
Gm6136	7453.5	7770.1	8355.6	7736.5	7985.1	7276.1	-0.02	1.0000
Rnf187	7453.5	7129.7	7871.0	6791.7	7405.0	6326.8	-0.07	0.9314
Prr12	7441.7	6818.1	7216.1	7881.9	7192.4	8690.0	0.18	0.6128
Pcx	7439.6	7635.3	7092.2	7232.3	7395.7	6684.6	-0.09	0.8732
Prps1	7427.9	7016.7	7255.4	6717.4	7283.8	6155.6	-0.07	0.9748
Myt1	7417.2	8002.4	7176.8	7326.8	6448.0	6566.0	-0.20	0.4117
Lats1	7415.1	6808.1	6275.0	7669.6	7015.8	8012.5	0.14	0.7636
Atp2c1	7414.0	7321.9	7649.3	7032.7	7002.5	6690.0	-0.08	0.8802
Bzw1	7404.5	7405.7	7604.0	7524.2	7292.0	7491.7	0.00	1.0000
Wdfy3	7402.3	7153.4	6311.3	8141.0	6875.2	9371.1	0.17	0.7007
Ly6e	7395.9	8339.5	8219.6	8424.4	9308.6	9411.9	0.12	0.7896
Cul4a	7394.9	7096.9	6654.9	7299.2	7172.9	6764.3	0.00	1.0000
Arpc2	7393.8	7782.0	8000.0	7871.3	7873.2	7457.2	-0.01	1.0000
Gna11	7387.4	7700.0	7561.7	7138.9	7224.3	6552.4	-0.13	0.7056
Ube4b	7386.3	7481.3	7223.1	7912.7	7226.3	8229.9	0.06	0.9256
Rab10	7381.0	6611.3	7151.6	6888.3	6879.3	6824.1	0.04	1.0000
Edem3	7378.9	7251.8	6497.7	7000.9	7079.5	7608.5	0.00	1.0000
Slc24a2	7377.8	6574.9	6196.5	6986.0	6507.6	7413.8	0.07	0.9343
Cd47	7375.7	6073.8	7157.7	6741.8	7317.7	6656.5	0.14	0.6862
Tmed4	7369.3	6780.8	6987.4	6324.7	6893.6	5684.6	-0.11	0.8456
Rangap1	7367.2	7797.5	7210.0	7783.2	7746.9	7790.6	0.01	1.0000
Ttc28	7362.9	7237.2	7114.3	9018.9	8825.0	10353.0	0.34	0.0398
Stt3b	7355.4	8089.0	7168.7	7875.6	8009.7	8661.0	0.03	1.0000
Foxp4	7352.2	6541.2	6302.2	6351.2	6459.3	6299.7	-0.04	0.9834
Cd24a	7346.9	6768.9	8127.9	5729.1	6010.6	5739.9	-0.22	0.3119
Med13	7343.7	6790.8	6954.1	7488.1	7419.3	8649.2	0.18	0.5557
Pik3r1	7338.4	6416.3	6956.1	7141.0	6717.0	8196.3	0.15	0.7441
Hnrnpc	7327.7	7023.1	7246.3	6930.8	7598.0	6443.7	-0.01	1.0000
Epn1	7324.5	7669.0	7687.6	7703.6	7641.1	7560.5	0.00	1.0000
Cct6a	7319.2	7364.7	7457.9	7332.1	7096.9	6999.8	-0.04	0.9778
Il6ra	7314.9	6809.9	6289.1	6995.6	7587.7	7017.0	0.07	0.9605
Cct8	7307.5	7261.8	7310.8	7701.5	7807.5	7543.3	0.07	0.8868
Ncoa2	7306.4	7524.2	7401.5	7648.4	7016.8	8012.5	0.01	1.0000
Akt3	7304.3	6447.3	6699.2	6891.5	6607.2	6787.9	0.05	0.9649
Adgrf5	7299.0	7298.2	7103.2	9194.0	8174.0	9936.3	0.29	0.1613
Btg2	7289.4	6000.9	6820.1	11914.7	11070.5	12693.5	0.87	0.0000
Atp9a	7288.3	7525.1	7636.2	6925.5	7094.9	7065.9	-0.09	0.8188
Herpud1	7288.3	7485.0	5710.8	6557.1	6000.4	6202.7	-0.20	0.5510
Hnrnpd	7280.9	7104.2	7559.7	7747.1	7291.0	8297.8	0.11	0.7896
Tug1	7279.8	6405.4	6416.1	6627.2	6374.1	6483.5	0.01	1.0000
Tmx4	7272.3	6672.3	7078.1	7819.3	8227.4	7263.4	0.19	0.4411
Lrrn1	7261.7	6594.9	5788.4	8184.5	8721.3	7352.2	0.26	0.3117
Atp6v0a1	7261.7	7634.4	7478.1	7570.9	7443.0	8021.5	0.01	1.0000
Abca2	7260.6	7178.9	6650.9	6789.6	6608.2	7189.1	-0.05	0.9572
Taok1	7259.5	6586.7	6427.2	7414.9	6632.8	8223.5	0.14	0.7414
Ubn1	7248.9	7284.6	6884.6	7570.9	7451.2	7507.0	0.05	0.9451
Mtss1	7246.8	7040.4	7138.5	7245.0	7073.3	7160.1	0.02	1.0000
Cacna1a	7244.6	7135.1	6468.5	6654.8	5204.6	6029.7	-0.22	0.5195
Synrg	7242.5	6862.8	6739.5	7229.1	6839.2	7445.5	0.06	0.9866
Dazap2	7238.2	6649.6	6936.0	6270.5	6742.7	6279.7	-0.05	0.9553
Cul3	7235.0	6917.4	6983.3	7465.8	7387.5	7428.2	0.09	0.8859
Zmynd8	7229.7	7006.7	7127.4	7260.9	7057.9	7531.5	0.05	0.9645
Sdcbp	7222.2	7070.5	7446.8	7142.1	7353.6	7131.2	0.02	1.0000
Ttll7	7218.0	6820.8	6152.1	6528.5	6786.9	6566.0	-0.03	0.9681
Ckap5	7213.7	6702.4	6630.7	7395.8	7380.3	7765.2	0.15	0.6143
Metap2	7213.7	7075.0	7445.8	7045.5	7515.9	6994.4	0.02	1.0000
Dmxl2	7212.7	7850.3	6108.8	8038.0	6994.3	8550.5	0.04	0.9679
Lrrc59	7211.6	7956.9	6144.1	7905.3	6844.4	8334.0	0.00	1.0000
Mtmr3	7206.3	7266.3	7269.5	7190.9	7162.6	6983.5	-0.03	0.9980
Dhx15	7204.1	6881.9	6594.4	7163.3	7117.5	7140.2	0.05	0.9660
Mcl1	7200.9	6764.4	7277.6	7876.6	7518.9	7772.4	0.17	0.5077
Esrp1	7200.9	7166.1	6243.8	7064.6	6918.3	6910.1	-0.03	0.9980
Mga	7195.6	6680.5	5780.3	7099.6	6174.9	7976.2	0.08	0.9286
Txnrd1	7195.6	6884.6	7117.4	7258.8	7227.3	6979.0	0.05	0.9662
Agfg1	7194.5	7057.7	6665.0	7282.2	7167.8	7421.0	0.05	0.9681
Anapc1	7193.5	7003.0	6347.6	7703.6	7436.8	8671.0	0.17	0.6283

9330182L06Ri	7187.1	6819.9	6248.8	6599.6	6567.1	6122.1	-0.07	0.9557
Carmil3	7181.8	7778.3	6631.7	6963.7	5996.3	6164.7	-0.24	0.3321
Sik3	7180.7	6996.7	5799.5	7790.7	7581.6	7858.5	0.15	0.7319
Ssrp1	7180.7	7471.3	6852.4	7928.7	7850.6	8295.1	0.10	0.8051
Cyhr1	7180.7	7175.2	7423.6	6965.8	7319.7	6299.7	-0.06	0.9400
Rab1b	7176.4	7710.0	7396.4	6957.3	7316.7	6245.3	-0.15	0.7420
Gcn1	7174.3	7365.6	6092.7	7142.1	6365.9	7912.8	-0.02	1.0000
Fbxo3	7173.2	6391.8	7163.7	6661.2	7180.1	6586.8	0.07	0.9649
Mapk8ip3	7171.1	6765.3	5772.3	6384.1	5628.7	6412.9	-0.11	0.8370
Dync1i2	7165.8	6886.4	6953.1	6434.0	7107.2	6740.8	-0.03	1.0000
Afdn	7163.6	7532.4	6894.7	8163.3	7329.0	9191.8	0.13	0.7821
Rab3gap2	7158.3	6964.8	6084.6	6777.9	6570.2	7181.9	-0.01	1.0000
Sbno1	7157.2	6579.4	6615.6	7282.2	6701.6	7476.3	0.10	0.8396
Tmem106b	7155.1	7325.6	7140.5	6946.7	7148.3	6723.6	-0.07	0.8826
Anp32e	7151.9	6928.3	6997.5	6818.3	7050.7	6951.8	0.00	1.0000
Tmem248	7150.9	6768.9	6178.3	6331.0	6382.3	6604.9	-0.06	0.9155
Mark2	7146.6	6169.5	6672.0	6596.4	6620.5	6606.7	0.07	0.8710
Agpat3	7145.5	7027.6	6846.3	6981.8	7190.4	6741.7	-0.01	1.0000
Kcnh6	7141.3	7331.9	6431.2	5786.5	4576.3	5758.0	-0.38	0.0468
Capns1	7132.7	6944.7	7529.4	6966.9	7361.8	7113.9	0.03	1.0000
Slc44a1	7127.4	6498.3	6216.6	7006.2	7393.7	6611.2	0.09	0.8440
Alg2	7118.9	6767.1	6515.8	6075.2	6280.7	6155.6	-0.12	0.7428
Ccny	7117.8	6472.8	6775.8	7056.1	7253.0	6796.9	0.10	0.7896
Gm7536	7117.8	8162.8	8532.0	7725.9	8339.3	6237.2	-0.11	0.9263
St13	7115.7	6493.8	7552.6	6619.8	7321.8	6419.2	0.04	0.9649
Notch1	7106.1	7038.6	6137.0	7893.6	7016.8	9445.4	0.19	0.6170
Col6a6	7104.0	7070.5	6300.2	6805.5	6468.6	8362.1	0.04	1.0000
Tab2	7100.8	7203.5	6865.5	7046.5	6779.7	7008.0	-0.04	0.9642
Fam20c	7099.7	7203.5	7077.1	6782.2	7151.4	6477.2	-0.07	0.9117
Arhgef1	7096.5	7205.3	6987.4	7079.4	6501.4	7064.1	-0.06	0.9500
Cxxc5	7096.5	6644.1	7166.7	6072.0	6239.6	5749.8	-0.14	0.6695
Ociad2	7092.2	7078.7	7545.6	6308.7	7151.4	5956.4	-0.12	0.7865
Soga1	7085.9	7046.8	5907.3	7089.0	5623.5	7831.3	-0.01	1.0000
Slc3a2	7081.6	7558.8	7370.2	7841.6	7946.1	7464.5	0.04	0.9813
Vdac1	7080.5	6595.8	7655.4	7022.1	7484.0	6756.2	0.08	0.9495
Zfp207	7080.5	7089.6	6505.8	6779.0	6389.5	6434.6	-0.10	0.8316
Gnl3l	7076.3	6615.9	6712.3	6498.8	7287.9	6258.9	0.01	1.0000
Rap1gap	7076.3	7466.8	7921.4	6376.7	5774.5	6658.3	-0.23	0.3422
Cd34	7068.8	7150.6	7328.9	7418.1	7632.9	7359.4	0.06	0.9479
Desi1	7067.7	7284.6	6986.4	7493.4	7338.2	7405.6	0.03	1.0000
Zfp512b	7066.7	7079.6	6398.0	7469.0	6797.1	7033.3	0.01	1.0000
Larp4b	7062.4	6393.6	6296.2	6931.9	7119.5	7553.2	0.15	0.6333
Bex2	7062.4	7160.7	7542.5	7270.5	8174.0	6341.3	0.01	1.0000
Rpl41	7049.6	8140.9	8281.1	8118.7	8910.2	7809.6	0.03	0.9967
Zfp148	7046.4	6551.2	6625.7	7183.5	6720.1	6778.8	0.06	0.9343
Cttn	7039.0	6977.5	7354.1	6434.0	6961.4	6778.8	-0.05	0.9542
Cbx5	7037.9	6561.2	6060.4	6978.6	6884.4	8231.7	0.15	0.7319
Maob	7037.9	7191.6	7587.9	8155.8	8568.3	7227.2	0.13	0.7989
Dpp8	7031.5	7028.6	6312.3	7206.8	6826.9	7761.6	0.05	0.9649
Slc25a51	7029.4	6225.0	6622.6	5867.1	6364.9	6499.8	-0.01	1.0000
Tmem214	7029.4	7504.1	6068.5	6694.1	6690.3	6550.6	-0.14	0.6900
Tbc1d16	7026.2	7461.3	7533.5	6055.0	5803.2	6646.6	-0.25	0.2462
Prlr	7025.1	6536.6	5015.6	8215.3	8411.2	7796.0	0.29	0.2486
Tmem131	7019.8	6847.3	6423.1	7808.7	7098.0	8358.5	0.17	0.6185
Zcchc14	7017.7	6458.3	6671.0	6649.5	6300.2	7092.2	0.04	0.9654
Top1	7016.6	6291.5	7037.8	6986.0	7026.1	6894.7	0.12	0.7383
Syne2	7015.5	7173.4	5583.9	7536.9	5815.5	8992.5	0.09	0.9582
Pten	7011.3	6448.2	6728.4	7033.8	6384.4	6851.3	0.05	0.9927
Dyrk2	7011.3	5518.1	6117.9	5844.8	5850.5	5560.5	0.02	1.0000
Lpp	7008.1	6233.2	6301.2	7693.0	7102.1	9607.5	0.31	0.1605
Tnks	7005.9	6537.5	6384.9	7510.4	6971.7	8315.9	0.19	0.5345
Slc38a5	6995.3	8079.9	5653.4	9275.7	7608.3	7581.3	0.07	0.9515
Ctrl	6992.1	11857.0	12847.3	27920.7	14377.7	20917.0	0.40	0.2743
R3hdm2	6984.6	7034.0	6872.5	6924.4	6723.2	7598.5	0.01	1.0000
Tbc1d1	6984.6	7344.7	6280.1	7401.1	7030.2	7070.5	-0.02	0.9931
Zcchc18	6984.6	7119.7	7465.0	6527.4	7193.5	5913.8	-0.11	0.8333
Ndufs2	6980.4	7121.5	6317.4	6694.1	6850.5	5910.2	-0.11	0.8333
Aamp	6975.0	6769.8	6989.4	6051.8	6859.8	5896.6	-0.11	0.8759
Kat6a	6974.0	6487.4	6211.6	6938.2	6372.0	7724.4	0.10	0.9209
Tpm4	6967.6	7063.2	6561.2	7523.1	7628.8	7815.0	0.11	0.7427
Kctd12	6963.3	6735.2	6418.1	7406.4	7175.0	8017.9	0.15	0.6710
Tmed9	6953.7	7138.8	6886.6	6635.7	7151.4	6868.5	-0.04	0.9731
Csnk1g2	6947.3	7298.2	7450.9	6734.4	6897.7	6137.5	-0.13	0.7433
Ptpa	6945.2	7291.8	7234.2	6572.0	6971.7	6594.0	-0.11	0.8455
Spop	6944.1	6685.1	6941.0	6920.2	7203.7	6977.2	0.06	0.9250
Retreg2	6938.8	6987.6	7391.4	6310.9	6811.5	5781.6	-0.14	0.7734

Tusc3	6938.8	6917.4	7003.5	5632.5	6443.9	4829.6	-0.26	0.3153
E330009J07Ri	6934.5	6499.3	6000.0	6187.7	6008.6	6171.9	-0.08	0.9500
Tenm3	6933.5	5945.3	5544.6	6318.3	6092.8	7230.8	0.11	0.8599
Golga2	6930.3	6439.1	6394.9	6827.8	6636.9	6899.3	0.06	0.9343
Kdm2a	6928.2	6733.4	6916.9	7523.1	6997.3	7635.7	0.12	0.7337
Brd3	6925.0	6266.0	6682.1	6979.6	6770.4	6971.7	0.12	0.7562
Yipf6	6918.6	6495.6	6209.5	6544.4	6594.9	6519.8	0.01	1.0000
Ptprt	6918.6	7024.9	6348.6	6768.4	5983.9	6696.4	-0.10	0.8330
Stk11	6917.5	6615.9	7092.2	6473.3	6640.0	6125.7	-0.05	0.9343
Bmpr1a	6914.3	6546.6	7115.3	6825.7	6661.6	6423.7	0.01	1.0000
Cdkn1b	6912.2	6525.7	6712.3	6325.7	6423.4	5652.9	-0.09	0.8769
Lrp6	6911.1	6854.6	6321.4	7242.9	6449.1	7857.6	0.07	0.9370
Cirbp	6911.1	7947.8	8586.4	7041.2	7325.9	6191.9	-0.18	0.5892
Ociad1	6910.0	6928.3	6828.2	6649.5	7184.2	6407.4	-0.03	0.9921
Spint1	6902.6	6697.9	7316.8	5702.6	6788.9	5548.8	-0.15	0.7621
Srsf7	6900.5	7099.6	6631.7	7801.3	7225.3	6988.0	0.05	0.9543
Ftl1-ps1	6900.5	6664.1	7298.7	6799.2	7495.3	6517.0	0.04	0.9831
Nmt2	6900.5	6375.4	6109.8	6155.9	6668.8	6289.7	0.00	1.0000
Phf20l1	6899.4	6192.2	5816.6	7483.9	6416.2	7229.9	0.16	0.6804
Hdgf	6895.1	6903.7	7052.9	7269.4	7578.5	7538.8	0.10	0.7771
Pitpnm2	6888.7	7412.1	6290.2	6878.8	5771.4	7465.4	-0.10	0.8462
Krt18	6886.6	7386.6	7265.5	6319.4	6551.7	6739.0	-0.16	0.6433
Pik3c2a	6884.5	6035.5	5882.1	7725.9	6198.5	6158.4	0.12	0.8410
Srebf1	6883.4	7617.1	6840.3	7833.1	7739.7	7213.6	0.01	1.0000
Sstr3	6882.3	6696.0	6144.1	6039.1	6134.9	5881.2	-0.14	0.7168
Il6st	6878.1	7119.7	7286.6	8335.2	7921.4	9963.5	0.26	0.2719
Dnajc13	6870.6	6432.7	5846.8	6997.7	6232.4	7048.7	0.07	0.9158
Pcnx3	6870.6	6655.9	6305.3	6245.0	5827.9	6381.2	-0.10	0.7989
Grk2	6868.5	6884.6	6513.8	6212.1	5842.2	5679.2	-0.20	0.4145
Ip6k2	6866.4	7136.1	6901.7	6376.7	6763.2	5516.2	-0.17	0.6256
Rps15	6865.3	7543.3	8595.4	7662.2	7919.4	6377.5	-0.04	0.9895
Dctn2	6857.8	7106.9	7466.0	6456.3	6889.5	6104.9	-0.12	0.7682
Arfgap3	6854.6	6786.2	6019.1	5902.2	6181.1	5931.0	-0.15	0.6631
Clptm1l	6846.1	7567.0	6447.3	7285.4	7052.8	6643.8	-0.08	0.8850
Degs1	6840.8	6906.5	6891.7	7145.2	7397.8	6300.6	0.01	1.0000
Mapk8ip1	6838.7	7632.6	6791.9	6544.4	6130.8	6102.2	-0.25	0.1759
Tmem127	6834.4	6013.7	6457.4	5971.2	6120.5	5790.6	-0.03	0.9936
Dnajb11	6833.3	7904.1	6322.4	6632.5	6956.3	6667.4	-0.18	0.4717
Klc1	6826.9	6728.8	7271.5	7126.1	5691.3	7114.8	-0.03	1.0000
Megf8	6826.9	6118.4	6176.3	5345.9	5048.6	5761.6	-0.18	0.5331
Tcp1	6820.5	6902.8	7174.8	7191.9	7074.3	7453.6	0.06	0.9392
Tcf4	6817.3	6091.1	7493.2	7920.2	7483.0	8837.6	0.34	0.0490
Fxr2	6809.9	6430.9	6633.7	6469.0	6520.9	5729.9	-0.05	0.9731
Chtop	6803.5	6582.2	6898.7	6906.4	7161.6	6805.1	0.07	0.9534
Rps23-ps1	6795.0	7544.2	8283.1	7059.3	7984.1	6042.4	-0.09	0.9244
Brd1	6790.7	6671.4	7040.8	7064.6	6656.5	6577.7	0.01	0.9967
Ap1g1	6789.6	6520.2	6202.5	7230.2	6957.3	7561.4	0.14	0.6583
Rnf145	6781.1	6679.6	6601.5	6078.4	6626.7	6104.0	-0.08	0.8726
Gpd2	6781.1	5612.8	6791.9	5119.8	5259.0	5354.0	-0.12	0.7168
Slc16a12	6763.0	6532.0	5892.2	6265.2	6525.0	6263.4	-0.03	1.0000
Atf4	6758.7	6766.2	6303.3	8373.4	7971.7	7585.9	0.22	0.2743
Eif1	6748.1	6713.3	6507.8	7241.8	7475.8	6469.9	0.07	0.9662
Rbbp6	6743.8	6737.0	6615.6	6874.5	6254.0	7002.5	0.00	1.0000
Timp3	6736.4	6468.3	7083.1	7735.5	8654.5	9786.0	0.37	0.0355
Slc33a1	6736.4	6589.4	6042.3	6142.1	6415.2	5338.6	-0.12	0.8470
Acat1	6734.2	7197.1	7438.8	7429.7	7190.4	6058.7	-0.05	1.0000
Ppp1r10	6732.1	6451.9	6096.7	6297.1	6255.0	6524.3	-0.02	1.0000
Paxip1	6731.0	6165.8	5789.4	5988.2	6150.3	5793.3	-0.04	0.9731
Tmem184b	6722.5	6282.4	6146.1	6254.6	6305.3	6295.1	0.00	1.0000
Lama4	6720.4	6440.0	6461.4	9016.7	9007.7	11015.1	0.52	0.0004
Gm10288	6718.2	7126.0	8064.5	7149.5	7587.7	5993.5	-0.05	0.9834
Pttg1ip	6718.2	6736.1	7066.0	5757.8	6660.6	6027.0	-0.12	0.7784
Dock6	6715.0	6930.2	6543.0	6735.5	5818.6	7323.2	-0.05	0.9805
Rabl6	6711.9	6167.6	6232.7	6320.4	5925.4	6183.7	-0.01	1.0000
Dapk1	6708.7	6562.1	6760.7	6068.8	5960.3	6327.7	-0.10	0.8742
1700086L19Ri	6706.5	7465.8	6750.6	6378.8	6016.8	5023.4	-0.30	0.1574
Gm9800	6684.1	6816.3	7155.6	6668.6	6841.3	6173.8	-0.05	0.9522
Gm12346	6679.9	6418.2	6363.7	6665.4	6766.3	7197.3	0.09	0.8648
Akap13	6677.8	5297.6	5272.5	7337.4	7071.3	7806.0	0.40	0.0175
Zfp91	6676.7	6148.5	5951.6	6262.0	6256.0	6338.6	0.02	0.9817
Oga	6675.6	6731.6	6738.5	6800.2	6497.3	7009.8	0.01	1.0000
Rps6ka3	6674.6	5734.9	5478.1	6244.0	6284.8	6546.0	0.12	0.8445
Arl6ip1	6663.9	5941.7	5866.0	5386.3	5568.1	5137.5	-0.14	0.7056
Zzef1	6661.8	6737.0	5893.2	7083.7	6201.6	7540.6	0.05	0.9682
Tlk1	6658.6	6133.9	6282.1	6373.5	6601.0	6266.1	0.05	0.9521
Eef1b2	6657.5	7062.3	7778.3	6837.4	7453.2	5782.5	-0.07	0.9475

Agap1	6654.3	5865.2	6295.2	6905.3	6566.1	7426.4	0.21	0.3477
Top2a	6645.8	6766.2	4103.8	6509.4	6150.3	8700.9	0.14	0.8884
Heg1	6638.3	6287.9	6261.9	8240.7	7717.1	8873.9	0.36	0.0244
Arl8b	6638.3	5977.2	5947.6	5767.4	6336.1	5903.8	0.00	1.0000
Ncoa1	6637.3	6679.6	5979.8	7190.9	6324.8	7286.0	0.06	0.9498
Ube3a	6635.1	5663.8	5789.4	6752.5	6147.2	6648.4	0.17	0.6683
Pum1	6635.1	6291.5	6525.9	6809.8	6491.2	7396.5	0.11	0.7873
Egr1	6628.7	5459.8	8475.5	28528.9	22969.6	36782.5	1.32	0.0000
Klhl9	6628.7	5636.5	5567.7	6267.3	6404.9	5606.7	0.08	0.9059
Usp22	6625.5	5738.5	6516.9	6350.1	6155.4	6368.5	0.10	0.8501
Rpl10a-ps1	6625.5	7170.7	7879.1	7405.3	7936.8	7128.4	0.05	0.9395
Polr2m	6625.5	6819.9	6989.4	6775.8	7170.9	6786.1	0.02	1.0000
Mon2	6620.2	6571.2	5878.1	6641.0	6096.9	6662.0	-0.01	1.0000
Prox1	6617.0	6261.5	5829.7	6977.5	6451.1	6960.9	0.11	0.8222
Npepps	6613.8	6518.4	6757.7	6500.9	6836.1	6437.3	0.01	1.0000
Ap3b2	6613.8	6754.3	6462.4	6823.6	6543.5	6629.4	-0.01	1.0000
Fbrsl1	6612.8	5863.4	6264.0	6125.1	5911.0	6270.7	0.04	0.9850
Col1a2	6611.7	6987.6	9301.7	12379.7	13714.4	26276.5	0.44	0.1865
Rapgef1	6610.6	6835.4	5849.9	6409.6	6336.1	6968.1	-0.03	0.9864
Zfp652	6608.5	6133.0	6041.3	6892.6	6644.1	7667.4	0.18	0.5628
Mtdh	6605.3	6153.1	6631.7	6234.4	6520.9	6204.5	0.02	1.0000
Acox1	6603.2	5960.8	6464.5	5993.5	6277.6	6327.7	0.04	0.9839
Mapre1	6594.6	6154.9	6329.4	6491.3	6277.6	7547.8	0.11	0.7873
G3bp1	6590.4	6826.3	6410.1	6676.0	6663.6	6881.2	-0.01	1.0000
Dsp	6588.2	6297.9	6456.4	7171.8	7416.3	8538.7	0.26	0.2196
Stk25	6587.2	6359.9	6511.8	5978.6	6509.6	5675.6	-0.07	0.9214
Ppp3r1	6581.9	6273.3	6380.8	5827.9	6332.0	5657.5	-0.08	0.9069
Mdm4	6576.5	6290.6	5720.9	6215.3	5259.0	6796.9	-0.04	1.0000
Kdelr1	6573.3	6570.3	6560.2	6371.4	6917.3	6327.7	-0.01	1.0000
Dync2h1	6569.1	6205.9	5426.7	7216.4	6408.0	8105.8	0.20	0.5644
Sptbn4	6568.0	6370.8	6575.3	6056.1	5707.7	5850.4	-0.11	0.8175
Wapl	6566.9	6070.2	6077.6	6425.5	5944.9	6288.8	0.03	0.9810
Sv2a	6560.5	5949.9	6623.7	5818.3	6440.8	5827.7	0.00	1.0000
Ptp4a2	6555.2	6462.8	6751.6	6264.2	6532.2	6349.5	-0.02	0.9850
Tspyl4	6553.1	6033.7	6066.5	5669.7	6103.0	5014.4	-0.11	0.8479
Impdh1	6552.0	7225.3	6611.6	6043.4	6575.3	5746.2	-0.20	0.3794
Inppl1	6543.5	6410.0	6127.9	5562.5	5827.9	5449.1	-0.17	0.5275
Ubr2	6535.0	6795.3	6572.3	7146.3	6428.5	7411.0	0.04	0.9770
Pfn1	6522.2	6655.9	6784.9	6595.4	7157.5	6972.6	0.05	0.9948
Plpp3	6521.1	6728.8	7179.8	7203.6	7794.1	7435.5	0.14	0.6752
Ppib	6513.7	7616.2	7520.4	7017.9	6886.5	7306.9	-0.08	0.9372
Zfp638	6511.5	6180.4	5856.9	6661.2	5822.7	5954.6	-0.01	1.0000
Slk	6508.3	6595.8	6372.8	6840.6	6306.3	7457.2	0.06	0.9312
Msn	6503.0	5795.9	6215.6	7648.4	7295.1	8156.5	0.36	0.0133
Pds5a	6499.8	6475.6	5725.9	6689.8	6338.2	7134.8	0.06	0.9525
Atp11b	6498.7	5439.7	5536.5	5631.5	5943.9	5743.5	0.06	0.9500
Mcam	6494.5	6352.6	6566.2	7341.6	6963.5	7570.5	0.18	0.4507
Mgrn1	6492.3	6309.8	6239.8	6126.2	6166.7	6089.5	-0.04	0.9781
Yy1	6487.0	6109.3	6539.0	6223.8	6220.1	5643.9	-0.03	1.0000
Arglu1	6487.0	6558.5	6024.2	6176.0	5517.8	6441.0	-0.10	0.9082
Sirpa	6484.9	6485.6	7161.7	6831.0	7187.3	6865.8	0.09	0.8791
Tet3	6484.9	6184.0	5925.4	6223.8	5720.1	7458.1	0.06	0.9463
Atp6v1a	6481.7	6054.7	5863.0	6264.2	6377.2	5918.3	0.03	1.0000
Tnrc6b	6479.6	5527.2	6760.7	7580.5	6442.9	8184.6	0.33	0.0740
Mast4	6472.1	5954.5	5181.8	6403.2	6026.0	7122.1	0.12	0.8339
Bcar1	6471.0	6247.8	6504.8	6518.9	6987.1	6127.6	0.05	0.9559
Brd4	6467.8	5917.1	6070.5	7591.1	6783.8	8283.3	0.30	0.1366
Wsb2	6463.6	6235.1	6082.6	5951.0	5945.9	5507.1	-0.10	0.8455
Wdr1	6461.4	5812.3	6431.2	6197.3	6514.8	6229.9	0.10	0.8395
Ets2	6459.3	6703.3	6872.5	7456.3	7537.4	8267.9	0.19	0.4579
Chmp4b	6459.3	6627.7	7081.1	6086.9	6469.6	5540.6	-0.13	0.7790
Ky	6457.2	5206.5	4862.5	5427.7	6178.0	6042.4	0.13	0.8113
Rhob	6456.1	6244.2	7358.2	8320.4	7753.0	8552.3	0.34	0.0363
Luc7l3	6455.1	6277.9	6739.5	6405.3	6054.8	6491.7	0.00	1.0000
Ltbp3	6447.6	6738.9	6965.2	6521.0	6335.1	7317.7	0.00	1.0000
Ica1	6444.4	6842.7	6361.7	5848.0	6271.4	5989.0	-0.16	0.6345
Cnot6	6440.1	6203.2	6070.5	6474.3	6001.4	6662.9	0.04	0.9884
Clint1	6432.7	5875.2	6093.7	6448.9	5956.2	6170.1	0.06	0.9474
Tmx1	6432.7	6011.9	6124.9	5573.1	5962.4	5585.0	-0.08	0.9452
Ipo7	6423.1	6308.8	6140.0	6883.0	6503.5	7222.6	0.11	0.8358
Fos	6419.9	4280.0	6384.9	33540.5	25522.1	36521.7	2.33	0.0000
Nid2	6418.8	6267.9	6324.4	7332.1	7375.2	7658.3	0.23	0.2514
Paip2	6418.8	6507.5	6970.3	6292.8	6972.7	5361.3	-0.07	0.9521
Gm10275	6408.2	7020.4	7572.8	6907.4	7376.2	5904.7	-0.05	0.9748
Ubqln1	6405.0	6051.0	6327.4	6496.6	6612.3	6468.1	0.09	0.8783
Bcor	6402.8	6206.8	6024.2	6322.5	6224.2	6711.8	0.04	0.9662

Ywhah	6390.1	5653.8	6337.5	5953.1	6354.6	5968.1	0.08	0.9280
Pja1	6390.1	6365.3	6414.1	6160.1	6977.8	5667.4	-0.02	1.0000
Rnf141	6380.5	6446.4	6357.7	5855.5	6178.0	5700.9	-0.11	0.7518
Zfp445	6373.0	6018.2	6008.0	6784.3	5917.2	7182.8	0.12	0.8103
Suco	6371.9	6251.5	5640.3	5766.3	6081.5	5764.3	-0.08	0.9155
Try4	6364.5	14433.4	8942.0	13482.6	8829.1	13475.2	0.07	0.9723
Med25	6362.4	6346.2	6280.1	6071.0	6206.7	6171.0	-0.04	1.0000
Scarb1	6358.1	6046.5	6554.1	6188.8	6345.4	6196.4	0.03	0.9841
Ptprb	6356.0	5979.1	5999.0	9730.1	8147.3	12556.7	0.62	0.0002
Nt5dc2	6351.7	7062.3	6171.3	6203.6	6541.5	6112.2	-0.14	0.6770
Klhdc10	6350.6	6320.7	6099.7	6767.3	6517.8	6861.2	0.08	0.8705
Ro60	6350.6	5885.2	5753.1	6297.1	6088.7	6398.4	0.08	0.8712
U2af2	6344.2	6660.5	6184.4	6129.3	5988.0	5851.3	-0.13	0.6601
Sf3b3	6334.6	6320.7	5633.2	7246.1	6849.5	7667.4	0.19	0.5490
Ube2z	6333.6	5724.0	6041.3	5685.6	5707.7	5045.2	-0.07	0.9196
Hnrnpr	6331.5	6073.8	5570.8	5590.1	5577.3	6172.8	-0.06	0.9516
Bcr	6329.3	5591.9	5482.1	5726.0	5877.2	6349.5	0.08	0.9099
Gm9843	6329.3	6449.1	7299.7	6079.4	7030.2	5848.6	-0.04	0.9936
Itgav	6326.1	5030.7	5008.5	5752.5	6049.6	5692.8	0.16	0.6868
Sgpl1	6326.1	6212.3	6729.4	5717.5	5719.0	5351.3	-0.15	0.6469
Emilin1	6325.1	6476.5	5724.9	6451.0	7249.9	7498.9	0.13	0.7470
Etf1	6321.9	6040.1	5716.9	6345.9	6244.7	6260.7	0.05	0.9559
Lbh	6320.8	6015.5	5923.4	6723.8	7218.1	7205.4	0.21	0.4006
Tnrc6c	6315.5	6109.3	5685.6	6345.9	5581.4	6558.7	0.01	1.0000
Elp3	6311.2	6196.8	5737.0	5584.8	6139.0	5585.0	-0.09	0.8455
Gm11478	6306.9	6827.2	7378.3	6703.6	7447.1	6535.2	0.01	1.0000
Zc3h14	6304.8	6457.3	6108.8	6470.1	6267.3	6249.8	-0.02	1.0000
Nsd2	6301.6	6245.1	5758.2	6711.1	5868.9	7286.0	0.08	0.9155
Zmiz2	6288.8	5969.0	6216.6	6533.8	6042.5	6553.3	0.08	0.8816
Tent5c	6286.7	5541.8	5657.4	5680.3	5672.8	5206.4	-0.02	1.0000
Smad5	6284.6	5541.8	5925.4	6636.8	6364.9	7472.6	0.25	0.2424
Samd4b	6281.4	5749.5	5396.5	5461.6	5474.7	6197.3	-0.01	0.9986
Hmgn2	6280.3	6638.6	6184.4	6050.8	6117.4	5820.5	-0.13	0.6872
Sema6d	6279.2	5689.4	5725.9	6371.4	6015.8	6817.8	0.15	0.7470
Pbxip1	6278.2	7214.4	6653.9	7710.0	7444.0	6375.7	0.01	1.0000
Wbp2	6267.5	6031.9	6715.3	5648.5	6298.1	5847.7	-0.03	0.9660
Clu	6266.5	7658.1	9173.8	10930.7	10936.0	14871.0	0.41	0.2190
Cep350	6265.4	6075.6	5431.7	6461.6	5535.2	7113.9	0.07	0.9516
Phf3	6265.4	6355.3	6039.3	6699.4	6082.5	6998.9	0.05	0.9592
Gm8203	6263.3	6611.3	5745.1	6202.6	6536.3	6170.1	-0.05	0.9679
Emp1	6262.2	6539.3	6568.2	6397.9	6847.4	7133.9	0.05	0.9559
Sh3glb1	6259.0	6112.1	6011.1	6794.9	6528.1	7085.9	0.14	0.7198
Tpm1	6250.5	6545.7	6894.7	6654.8	6472.7	7442.7	0.06	0.9521
Vps13d	6244.1	6414.5	6000.0	6497.7	6049.6	7235.3	0.05	1.0000
Gatm	6241.9	5683.0	6435.2	5939.3	5947.0	6159.3	0.06	0.9446
Safb2	6241.9	5887.0	5249.4	6253.5	5117.4	6422.8	0.02	1.0000
Uqcrc1	6241.9	6622.2	6304.3	6185.6	6454.2	5737.2	-0.09	0.8558
Nr2c2	6234.5	5950.8	5881.1	6175.0	5774.5	6097.7	0.01	1.0000
B4galt6	6227.0	5637.4	5652.4	6206.8	5976.7	5739.9	0.07	0.9654
Rps3a2	6223.8	6618.6	7327.9	6310.9	7081.5	5805.1	-0.05	0.9729
Pip4k2c	6222.8	5999.1	6172.3	5937.2	6203.7	5614.9	-0.02	1.0000
Smc3	6219.6	6594.9	6154.1	7326.8	6479.9	7376.6	0.10	0.8062
Slc12a7	6219.6	5692.1	5787.4	5408.6	6031.2	5958.2	0.02	0.9945
Hadha	6215.3	6181.3	6357.7	6800.2	6958.3	6907.4	0.14	0.6049
Tnpo2	6214.2	5683.9	5485.1	5447.8	5358.6	5560.5	-0.06	0.9235
Zfp871	6213.2	5379.6	5072.0	6123.0	5876.1	7061.4	0.20	0.6120
Zbtb4	6213.2	5820.5	5859.9	6009.4	6035.3	5835.9	0.03	1.0000
Pak2	6212.1	5609.2	5371.3	6359.7	6183.1	6257.1	0.14	0.7075
Ogt	6212.1	5809.6	5512.3	6274.8	4954.1	6635.7	0.03	0.9839
Fbxw11	6207.8	5986.3	6398.0	6027.4	6397.7	5868.5	0.02	1.0000
Prkaa1	6202.5	5550.9	5291.7	5809.8	5890.5	5816.9	0.06	0.9244
Bace1	6202.5	5916.2	5913.3	5410.7	5689.3	5361.3	-0.10	0.7830
Fip1l1	6201.5	6418.2	6021.1	6234.4	6092.8	5849.5	-0.07	0.9216
Gpc4	6200.4	6594.9	6929.9	7006.2	7316.7	6689.1	0.08	0.8938
Xiap	6200.4	6295.2	6191.4	6393.7	6126.7	6991.7	0.05	0.9748
Isoc1	6199.3	6702.4	6244.8	6233.4	6325.8	5971.8	-0.10	0.8714
Rnase4	6198.3	5907.1	6718.4	6221.7	6551.7	5779.7	0.04	0.9516
Srsf3	6198.3	6791.7	6480.6	6672.8	6442.9	6056.9	-0.07	0.9263
Fstl1	6197.2	6004.6	7631.2	8977.5	8716.1	10641.9	0.53	0.0007
Arhgef11	6195.1	6097.5	6145.1	6541.2	6248.8	6477.2	0.07	0.9237
Atp13a3	6192.9	5755.9	4904.8	6153.8	5554.7	6057.8	0.04	0.9557
Peg13	6192.9	5667.5	5533.5	5493.5	5851.5	5814.2	0.01	1.0000
Tmem63a	6189.7	5938.1	5518.4	5588.0	5794.0	5316.9	-0.08	0.9007
Ush1c	6189.7	5505.3	5230.2	4598.6	4211.8	4481.8	-0.29	0.1464
Lnpep	6187.6	5048.0	5270.5	6440.4	5625.6	6993.5	0.26	0.2922
Ankrd40	6176.9	5385.1	5633.2	5590.1	6113.3	5360.4	0.05	0.9545

Rtcb	6175.9	6272.4	6502.7	6083.7	6440.8	5834.1	-0.03	0.9850
Ogdhl	6173.8	5420.6	5963.7	5320.4	5705.7	5229.9	-0.02	0.9868
Ago1	6173.8	5810.5	5737.0	5342.7	5262.1	5702.7	-0.09	0.8314
Gmppa	6171.6	6236.9	5446.8	5223.8	5377.1	4628.5	-0.25	0.2753
Psip1	6169.5	5973.6	6054.4	6034.9	6066.1	5291.5	-0.04	0.9729
Efcab1	6167.4	5820.5	5338.0	5669.7	5971.6	4857.7	-0.07	0.9385
Furin	6165.2	5925.3	6020.1	5833.2	5814.5	6299.7	0.01	1.0000
Ilf3	6164.2	6614.0	6370.8	6758.8	6662.6	6624.8	0.02	0.9848
Ube2o	6163.1	5651.1	5723.9	5849.1	5989.1	5709.1	0.04	0.9556
Bmpr2	6161.0	5245.7	5884.1	6593.2	6340.2	7798.7	0.32	0.0892
Cln6	6161.0	6407.2	6586.4	5966.9	7165.7	5933.7	-0.01	1.0000
Api5	6159.9	5539.0	5931.5	6064.6	6227.3	5623.9	0.08	0.8816
Dlst	6158.8	6195.0	6028.2	6294.9	6263.2	6280.6	0.02	0.9857
Ssbp4	6158.8	5728.5	5694.7	4757.8	5137.9	3970.9	-0.28	0.2241
Riok3	6157.8	6298.8	6394.9	6681.3	6462.4	6594.0	0.06	0.9126
Nus1	6156.7	5804.1	5667.5	5453.1	5696.4	4908.4	-0.11	0.7896
Ints3	6152.4	6247.8	5738.0	5917.0	5848.4	6159.3	-0.05	0.9342
Arpc1a	6147.1	6348.0	6669.0	5782.2	6183.1	5352.2	-0.13	0.7775
Grik5	6139.7	6009.1	6182.3	5408.6	4969.5	5153.8	-0.20	0.3657
Etl4	6138.6	6095.7	6660.9	7163.3	6370.0	7239.8	0.16	0.6116
Mboat2	6138.6	5028.9	5656.4	4653.8	4571.1	4495.4	-0.15	0.6381
Rims3	6136.5	6383.6	6687.1	6054.0	6164.6	6130.3	-0.06	0.9498
Mbp	6131.1	6108.4	6266.0	5886.2	5204.6	5314.2	-0.15	0.6287
Vat1	6130.1	6085.6	7170.8	5522.1	5999.3	5877.6	-0.08	0.9152
Atl3	6122.6	5988.2	6115.8	6814.0	6422.4	7311.4	0.17	0.5458
Ythdf3	6119.4	5468.0	5521.4	6356.5	6177.0	6476.3	0.18	0.5347
Atp6v0d1	6113.0	6253.3	6085.6	5882.0	6304.3	5681.9	-0.06	0.9462
Limd1	6112.0	6347.1	6250.9	5636.8	5988.0	5738.1	-0.12	0.7619
Dennd5a	6110.9	5962.7	6224.7	6043.4	5945.9	6969.9	0.07	0.9057
Ralgapb	6109.8	5903.4	5516.4	6568.8	5979.8	6710.0	0.11	0.8150
Capzb	6108.8	6087.5	6117.9	6156.9	6288.9	5586.8	-0.02	1.0000
Atp6v0b	6108.8	6040.1	5972.8	5330.0	6013.7	4282.5	-0.18	0.6218
Nptxr	6107.7	6224.1	6227.7	5877.8	5959.3	5916.5	-0.07	0.9148
Npc2	6104.5	5570.9	5892.2	5390.5	6446.0	5124.0	0.00	1.0000
Slc25a5	6104.5	7040.4	6147.1	6548.6	6702.7	6140.2	-0.09	0.8558
Anp32a	6101.3	6294.3	6856.4	6095.4	6304.3	5624.9	-0.07	0.9731
Tubb4b	6100.2	6050.1	5299.7	5607.1	5690.3	5660.2	-0.08	0.8710
Cdk4	6097.0	6435.5	6286.1	6284.3	6318.7	6186.4	-0.03	0.9877
Chpf	6096.0	6041.0	5299.7	5744.0	5877.2	5860.4	-0.04	0.9884
Ascc3	6093.8	6103.9	5154.6	6465.8	6468.6	6794.2	0.11	0.8213
Prkce	6090.6	5782.3	6291.2	6410.6	6270.4	6274.3	0.11	0.7873
Itfg1	6084.2	6630.4	6581.3	6119.8	6174.9	5132.1	-0.16	0.6674
Khsrp	6083.2	6041.9	5812.6	6007.3	5969.6	6595.8	0.03	0.9925
Scfd1	6078.9	6657.8	5793.4	6466.9	6569.2	5682.8	-0.06	0.9148
Abl1	6076.8	5866.1	5851.9	6327.8	5935.7	6619.4	0.09	0.9108
Rbfox2	6074.7	5759.5	5769.2	6307.7	5853.5	6844.9	0.12	0.8370
Mef2a	6073.6	5790.5	5987.9	6064.6	5909.0	6262.5	0.06	0.9474
Slc30a5	6070.4	5999.1	5444.8	4860.8	5235.4	4866.7	-0.24	0.2740
Ap2a2	6066.1	5828.7	6423.1	6507.2	6321.7	6734.4	0.14	0.7336
Tmem176b	6064.0	6438.2	6494.7	6035.9	6881.3	5789.7	-0.04	0.9858
Rfk	6061.9	5794.1	5927.4	5604.9	6459.3	5133.0	-0.02	1.0000
4930402H24R	6060.8	5375.0	6000.0	5336.4	5396.6	5580.5	0.00	1.0000
Rai1	6055.5	5414.2	5060.9	5429.8	5077.3	6235.3	0.03	1.0000
Sec22b	6054.4	5825.1	5499.2	5985.0	6174.9	5452.8	0.01	1.0000
Odf2	6053.3	5523.5	4804.0	5434.0	5019.8	5841.3	-0.02	1.0000
Ankrd10	6052.3	5346.8	5045.8	5166.5	4677.9	4847.7	-0.12	0.7976
Mroh1	6050.1	5650.2	5209.0	5169.7	5256.0	5450.0	-0.09	0.8946
Nek9	6048.0	5838.8	5764.2	5991.3	5850.5	6314.1	0.05	0.9471
Gm6863	6048.0	6099.3	6329.4	5678.2	6029.1	5193.7	-0.11	0.7908
Cpeb4	6042.7	5401.5	5514.3	5890.5	5455.2	5873.9	0.07	0.9334
Hsp90aa1	6034.2	5839.7	6195.4	7078.4	6868.0	7403.8	0.26	0.1492
Stard7	6034.2	5724.0	5540.5	5687.7	6015.8	5683.7	0.01	1.0000
Pink1	6031.0	6385.4	6073.5	5149.5	5351.5	4624.9	-0.30	0.0913
Ppp2r5a	6029.9	5605.5	6179.3	5766.3	5998.3	4947.3	-0.03	1.0000
Pnisr	6027.8	6304.3	6027.2	5934.0	4959.2	6497.1	-0.10	0.8816
Setd3	6025.6	6344.4	6231.7	5899.0	6283.7	5591.3	-0.08	0.9278
Ank2	6020.3	5020.7	5589.9	5880.9	4902.8	6869.4	0.16	0.7420
Cd2ap	6017.1	6212.3	5622.1	5947.8	5845.3	6412.0	-0.02	1.0000
Lars	6010.7	6386.3	5796.5	5964.8	5780.6	5936.4	-0.10	0.8366
Cbfa2t3	6006.5	5335.9	5660.4	5886.2	5371.0	6078.6	0.09	0.8801
Upf1	6004.3	5065.3	5148.6	6146.3	5450.0	5677.4	0.15	0.7108
Mbd2	6002.2	6292.4	6449.3	5982.8	6360.8	5382.1	-0.08	0.9148
Plekhg3	6001.1	5556.3	5753.1	6340.6	6106.1	6627.5	0.17	0.5886
Ctsd	5996.9	6244.2	6283.1	5665.4	6130.8	5369.4	-0.11	0.8059
Ptprs	5992.6	5864.3	6306.3	5971.2	5567.1	6316.9	0.01	1.0000
Stard10	5992.6	5839.7	5949.6	5461.6	5161.5	4708.2	-0.18	0.5423

Arid2	5991.5	5835.1	6035.2	6370.3	5500.3	6489.9	0.06	0.9342
Git1	5989.4	6279.7	5929.4	5774.8	5964.4	6354.9	-0.05	0.9487
Mthfd1	5984.1	5679.3	5808.5	6762.0	6799.2	5858.5	0.16	0.6807
Slc35f5	5979.8	5565.5	6170.3	5320.4	5793.0	4929.2	-0.07	0.9362
Phldb2	5972.4	5210.2	5306.8	5938.3	5628.7	6542.4	0.18	0.5901
Glis3	5971.3	5746.7	4544.1	6159.1	5529.1	6297.8	0.07	0.9439
Cbarp	5969.2	5650.2	5229.2	4851.2	5075.3	4556.0	-0.20	0.4627
Mau2	5968.1	6253.3	5749.1	5696.2	5144.0	5458.2	-0.18	0.5345
Elovl5	5964.9	5598.2	6098.7	5646.3	6264.2	5581.4	0.04	0.9557
Klhl32	5964.9	5867.0	5197.0	5088.0	5095.8	5511.6	-0.14	0.7182
Eif5b	5958.5	5553.6	5867.0	6383.0	6378.2	6560.5	0.19	0.3702
Txnl1	5958.5	5669.3	5525.4	5369.3	5757.0	4931.0	-0.08	0.9100
Foxo1	5954.3	5336.8	6279.1	6545.5	6322.8	6570.5	0.24	0.1879
Gm14586	5953.2	6560.3	6924.9	6203.6	6651.3	5498.9	-0.09	0.8770
Hif1a	5950.0	5684.8	5656.4	6109.2	6076.3	6223.6	0.10	0.8262
Fmnl2	5950.0	5597.3	6096.7	5907.5	5653.3	5832.3	0.04	0.9858
Rab11a	5950.0	5291.2	5607.0	5014.7	5344.3	4797.9	-0.08	0.8772
Cox7a2l	5950.0	5185.6	6177.3	4417.1	5629.7	4334.1	-0.14	0.7896
Rab18	5948.9	5448.8	6062.4	5636.8	6168.7	5102.2	0.02	0.9823
Vezf1	5947.9	5513.5	5741.0	5832.1	5853.5	6027.0	0.08	0.8767
Nap1l4	5946.8	5787.7	5852.9	5614.5	5839.2	5466.3	-0.04	0.9552
Dop1b	5936.1	5708.5	5093.2	5827.9	5448.0	5923.8	0.01	1.0000
Matr3	5936.1	5698.5	5894.2	5577.3	5993.2	5133.9	-0.04	0.9896
Cad	5934.0	6698.8	4782.9	6059.3	5722.1	6832.2	-0.05	0.9858
Wdr13	5929.7	5776.8	5772.3	5751.4	5295.0	5317.8	-0.08	0.8899
Arhgap21	5928.7	5900.7	5221.1	6307.7	5198.5	6463.6	0.03	0.9956
Strn4	5928.7	6093.8	6165.2	6083.7	6190.3	5889.3	-0.01	1.0000
Ube2q1	5927.6	5698.5	5612.1	5261.0	5542.4	4936.5	-0.11	0.7780
Tapbp	5920.2	5539.9	6101.7	5414.9	6210.8	5077.8	-0.01	1.0000
Fam219a	5919.1	5657.5	5663.5	5496.7	4942.8	5290.6	-0.10	0.8314
Trappc11	5915.9	5288.5	5196.0	5939.3	6154.4	5676.5	0.14	0.7263
Mtmr6	5914.8	5348.6	5461.9	5580.5	5483.9	5263.4	0.01	0.9980
Col4a3bp	5906.3	5449.8	5710.8	5800.3	5852.5	5796.9	0.08	0.8560
Eif3f	5906.3	5951.7	6466.5	5611.3	6410.0	6081.4	0.01	1.0000
Ankrd52	5901.0	5486.2	4757.7	5550.8	4706.6	6169.2	0.01	1.0000
Sacm1l	5899.9	5478.0	5589.9	5795.0	6112.3	5460.9	0.06	0.9382
Cpsf6	5899.9	5826.9	5493.2	6038.0	5321.7	6257.1	0.01	1.0000
Ddx42	5898.8	5930.8	5747.1	6118.7	6080.4	6148.4	0.04	0.9703
Ttc39b	5897.8	5439.7	5140.5	6153.8	5596.8	6130.3	0.12	0.8049
Jmjd1c	5892.4	6035.5	5453.9	6673.9	5991.1	7137.5	0.13	0.8204
Hook3	5890.3	5576.4	5232.2	5618.7	4874.0	6255.3	0.00	1.0000
Map7d1	5888.2	5364.1	5807.5	5704.7	5336.0	6122.1	0.07	0.9232
Dlg4	5881.8	6069.2	5773.3	5770.5	5159.4	5536.1	-0.13	0.7147
Set	5876.5	5843.3	6275.0	6089.0	6070.2	5795.1	0.03	0.9804
Ppp4r1l-ps	5875.4	6404.5	5205.0	5451.0	4271.3	5774.3	-0.23	0.4786
Bpnt1	5874.3	5349.5	5897.2	5504.1	6001.4	4660.2	-0.01	0.9982
Cerk	5872.2	5283.9	5509.3	5983.9	5786.8	5644.8	0.11	0.7869
Slitrk6	5870.1	5365.0	5359.2	5237.6	5481.8	4090.5	-0.12	0.8575
Kifc3	5863.7	6115.7	6357.7	5549.7	5670.8	5787.0	-0.10	0.8314
Sqle	5862.6	5794.1	4930.0	6141.0	6317.6	5129.4	0.03	0.9748
Sars	5860.5	5827.8	5520.4	5775.8	5935.7	5434.6	-0.02	1.0000
Dtx3	5860.5	5734.9	6022.1	5342.7	5759.1	4908.4	-0.10	0.8141
Prkaca	5859.4	5442.5	6031.2	5940.4	5925.4	5304.2	0.05	0.9395
Mgat3	5859.4	5260.3	5317.9	4768.4	5093.7	4691.0	-0.12	0.7830
Actn1	5858.4	5537.2	5841.8	7067.7	6694.4	7846.7	0.33	0.0525
Uhmk1	5858.4	5299.4	5552.6	5795.0	5472.6	6189.1	0.11	0.7811
Rpa1	5855.2	6236.0	5626.2	6412.8	6395.7	5910.2	0.01	1.0000
Spc25	5854.1	5806.9	4167.2	5909.6	6423.4	5795.1	0.09	0.9269
Wipi1	5854.1	5042.5	4393.9	4962.7	5090.7	4889.4	-0.02	0.9931
Dagla	5853.0	5693.0	6106.8	5196.2	5018.8	5271.6	-0.14	0.6871
Rnf14	5852.0	5898.9	5757.2	5335.3	5739.6	4748.1	-0.14	0.7255
Ssb	5850.9	6180.4	5983.9	6147.4	6149.2	5559.6	-0.04	0.9794
Tacc1	5848.8	5048.9	5152.6	5692.0	5407.9	6447.3	0.17	0.5985
Ctcf	5847.7	5721.2	5703.8	5682.4	5669.7	5718.1	-0.01	1.0000
Cluh	5846.6	5324.9	5448.8	5411.7	4913.0	5210.9	-0.05	0.9731
Fam210b	5845.6	6077.4	6308.3	5778.0	6754.0	6161.1	0.03	0.9972
Nfx1	5843.4	5760.4	5381.3	5583.7	5176.9	5433.7	-0.08	0.8948
Tom1l2	5841.3	6082.9	6366.7	5762.0	5478.8	6037.0	-0.07	0.8868
Mink1	5836.0	5944.4	5642.3	5530.6	5273.4	5697.3	-0.10	0.8396
Pgrmc2	5836.0	6195.0	6136.0	5661.2	5941.8	5193.7	-0.13	0.7310
Edem1	5834.9	5946.3	5404.5	5844.8	4933.6	6517.0	-0.03	1.0000
Ralbp1	5833.8	5918.0	6298.2	5762.0	6073.3	5306.9	-0.05	0.9679
Cacfd1	5831.7	5387.8	5063.0	4654.9	4936.6	5040.6	-0.13	0.7571
Pcyox1	5815.7	4970.6	5398.5	4858.7	5381.2	5229.9	0.03	1.0000
Clta	5812.5	5592.8	6036.2	5232.3	5947.0	4875.8	-0.07	0.9096
Rnf11	5809.3	5496.2	5946.6	5657.0	6041.4	5217.3	0.02	0.9850

Unc13a	5808.3	6287.0	4871.5	6181.4	5429.5	6108.5	-0.05	0.9529
Kctd20	5808.3	6066.5	5861.9	5603.9	5722.1	5572.3	-0.09	0.8413
Megf9	5806.1	5100.8	5415.6	5919.2	5590.7	5174.7	0.10	0.8624
Mark3	5806.1	5630.1	5340.0	5597.5	5635.9	5508.0	-0.01	0.9986
Adipor1	5802.9	6047.4	6274.0	5487.1	6062.0	5138.5	-0.11	0.8267
Kcnb1	5801.9	5325.9	4906.8	4957.4	4891.5	5133.0	-0.08	0.8899
Qrich1	5799.7	5510.8	5052.9	5787.5	5700.5	5658.4	0.05	0.9679
Sdf2l1	5799.7	8176.4	5205.0	6487.1	6514.8	5422.9	-0.29	0.1838
Zc3h7a	5794.4	5610.1	4407.0	5545.5	4590.6	5791.5	-0.04	0.9937
Nectin3	5791.2	5706.7	5542.5	5171.8	5290.9	4590.5	-0.17	0.5723
Tcaf1	5789.1	5304.9	5554.6	5455.3	5885.4	5713.6	0.08	0.8515
Spock1	5780.6	6568.5	5582.9	5985.0	6203.7	6643.8	-0.04	0.9887
Svil	5774.2	5693.9	5556.7	6320.4	5931.6	6996.2	0.16	0.7091
Slc35b1	5772.0	6442.8	5312.8	5942.5	5891.5	5292.4	-0.13	0.7147
Enah	5771.0	5215.6	5131.5	5502.0	5373.0	6317.8	0.11	0.8366
Syvn1	5769.9	6149.4	4541.0	4980.8	4341.1	4787.9	-0.30	0.1838
Plec	5766.7	5467.1	5473.0	6459.5	5514.7	8571.3	0.26	0.4425
Cab39	5765.6	5043.4	5482.1	5477.6	5521.9	5226.3	0.08	0.9090
Rasgrf1	5765.6	5529.0	5010.6	4740.8	5020.8	5357.6	-0.12	0.8207
Ncam1	5762.5	4657.2	5947.6	6377.7	6100.0	5913.8	0.33	0.0268
Hnrnpf	5762.5	5873.4	5508.3	5458.4	5762.2	5227.2	-0.08	0.8758
Mllt6	5752.9	5228.4	5903.3	4950.0	5013.6	5518.0	-0.04	0.9916
Prune2	5749.7	6814.5	6488.6	6784.3	6564.0	7285.1	0.03	0.9729
Dab2ip	5748.6	5742.2	6349.6	5917.0	5713.9	6471.8	0.06	0.9342
Lrp1	5747.5	5206.5	5260.4	7323.6	5840.2	9496.1	0.32	0.4470
Sgms2	5746.5	5181.0	4928.9	5186.7	5674.9	5070.5	0.03	1.0000
Pomt2	5743.3	5447.9	5080.1	5229.2	4957.2	5172.9	-0.08	0.8934
Gapvd1	5734.7	5387.8	5477.1	5617.7	5159.4	5699.1	0.02	0.9893
Avl9	5734.7	5232.0	5273.5	5283.3	4982.8	4805.1	-0.06	0.9117
Prdx2	5734.7	5764.1	6105.8	5502.0	5729.3	4838.6	-0.10	0.8599
Xpo1	5733.7	6169.5	5795.4	6442.5	6267.3	7152.0	0.10	0.8316
0610040J01Ri	5731.6	5325.9	5164.7	5126.2	5827.9	4605.9	-0.04	0.9933
Rab22a	5729.4	5500.8	5447.8	5263.1	5238.5	5396.6	-0.05	0.9279
Pebp1	5728.4	6080.2	6240.8	5667.6	6272.5	4970.0	-0.09	0.8556
Jpt2	5728.4	6022.8	5393.4	5564.6	5512.7	5521.6	-0.10	0.7837
Dido1	5723.0	5019.7	4831.2	5483.9	5108.1	6029.7	0.12	0.8240
Ccnt2	5722.0	4902.2	4768.7	5352.3	4853.5	5058.7	0.03	0.9895
Rab5c	5722.0	5802.3	5681.6	5437.2	5941.8	5029.8	-0.07	0.8871
Prdx6	5719.8	5834.2	5785.4	5554.0	5938.8	5656.6	-0.03	1.0000
Tbc1d9b	5718.8	5756.8	5496.2	5936.1	6071.2	5917.4	0.05	0.9487
Tead1	5709.2	4846.7	4446.3	6095.4	5424.4	6796.0	0.27	0.3088
Ibtk	5709.2	5720.3	4784.9	5781.2	5182.0	6155.6	0.02	0.9932
Dnah9	5709.2	5637.4	4113.8	5290.7	4973.6	5920.1	-0.02	1.0000
Slc7a5	5709.2	5593.7	5243.3	5133.6	5221.1	5723.6	-0.05	0.9660
Cltb	5701.7	5727.6	5363.2	5551.9	5870.0	5027.9	-0.05	0.9402
Cct3	5693.2	5903.4	5860.9	5793.9	5789.9	5730.8	-0.03	0.9931
Arhgef16	5693.2	5241.1	5158.7	4936.2	5357.6	4553.3	-0.08	0.8773
Znrf2	5693.2	5443.4	5667.5	5148.5	5204.6	4552.4	-0.13	0.7655
Ssbp3	5688.9	5335.9	6033.2	5249.3	5591.7	5147.5	-0.02	0.9884
Ralgapa1	5686.8	5585.5	4698.2	6532.7	5782.7	7056.9	0.20	0.5559
Acbd3	5685.7	4979.7	5059.9	5561.4	5436.7	5042.4	0.08	0.8730
Bcl9	5683.6	5601.9	5716.9	5911.7	5912.1	6279.7	0.10	0.8314
Pld3	5677.2	5592.8	5382.3	5344.9	5506.5	4987.2	-0.07	0.8796
Tspan15	5674.0	5759.5	6168.2	4918.1	5111.2	4223.6	-0.25	0.2833
Slc25a39	5666.6	5774.1	5653.4	5926.6	6036.3	5649.3	0.03	1.0000
Slc38a2	5665.5	5266.6	4993.4	6694.1	5986.0	7026.1	0.28	0.1692
Ppp4r3a	5660.2	5558.2	5403.5	5548.7	5518.8	5743.5	0.01	1.0000
Asah1	5655.9	5711.2	5553.6	5620.9	5911.0	5124.0	-0.03	0.9877
Phf12	5654.8	5355.9	5296.7	5464.8	5263.2	5634.8	0.02	1.0000
Npnt	5653.8	5621.9	6049.3	5406.4	5481.8	4645.7	-0.11	0.8108
Pkd2	5651.6	5314.0	5071.0	5611.3	5238.5	5838.6	0.06	0.9269
Abcb10	5651.6	5464.3	5833.7	5067.8	5149.2	4673.8	-0.14	0.6812
Tmem181b-ps	5647.4	5312.2	1437.8	4665.5	3999.2	4628.5	0.02	1.0000
Otud5	5645.2	5522.6	5263.5	5270.6	5404.8	4766.2	-0.09	0.8711
Ppm1g	5642.0	5798.7	5364.2	5769.5	5825.8	5501.7	-0.02	1.0000
Slc22a23	5641.0	3676.0	3291.7	3853.4	4491.0	4082.3	0.02	0.9981
Nucb2	5638.9	6615.9	5239.3	6538.0	6212.9	6200.9	-0.03	0.9885
Puf60	5637.8	5866.1	5926.4	5695.2	6129.7	5517.1	-0.02	0.9922
Chd6	5635.7	5618.3	5602.0	6262.0	5283.7	6938.2	0.12	0.8316
Lmna	5635.7	5418.8	5464.0	5727.0	5865.9	5937.3	0.10	0.8316
Alcam	5634.6	6195.0	5738.0	6486.0	6520.9	6794.2	0.10	0.8315
Cd63-ps	5634.6	5683.9	6108.8	5594.3	6650.3	4633.9	-0.02	0.9922
Uqcrc2	5633.5	6083.8	5897.2	5972.2	6077.4	5300.6	-0.06	0.9312
Sec14l1	5632.5	4993.3	5210.1	5433.0	5456.2	5454.6	0.10	0.7836
Strap	5632.5	5700.3	5685.6	5571.0	5921.3	5144.8	-0.04	0.9649
Daam1	5631.4	5580.9	5169.8	5951.0	5859.7	5915.6	0.08	0.8880

Ets1	5630.3	4507.8	5619.1	7164.3	6225.2	6790.6	0.49	0.0003
Dysf	5628.2	5284.9	5386.4	6035.9	5058.8	7296.0	0.17	0.6759
Slc39a9	5626.1	5053.5	4780.8	5399.0	5206.7	5101.3	0.04	0.9557
Otud4	5625.0	4567.9	4299.2	5384.1	5141.0	5873.0	0.20	0.5520
Tsc2	5616.5	5281.2	5205.0	5631.5	5432.6	5882.1	0.09	0.8455
Nomo1	5614.3	6407.2	5677.6	6148.4	6011.7	6364.0	-0.03	0.9944
Map7	5613.3	5288.5	5300.7	5092.2	4762.1	5125.8	-0.08	0.8946
Tecr	5613.3	6023.7	5891.2	5448.9	5924.4	5115.8	-0.11	0.8144
Zzz3	5612.2	5606.4	5342.0	5861.8	5599.9	5951.8	0.05	0.9554
Epb41	5612.2	5209.2	5256.4	5153.8	4924.3	5585.0	0.00	1.0000
Fmn2	5605.8	5862.4	6716.3	6315.1	6798.2	5957.3	0.09	0.9292
Brd8	5604.8	5760.4	5359.2	5762.0	5365.8	5813.3	-0.02	0.9893
Armcx2	5604.8	5712.1	5522.4	4999.9	5465.4	5544.2	-0.09	0.8479
Scamp1	5603.7	5109.9	5302.8	5364.0	5610.2	4902.0	0.03	0.9932
Gm10052	5601.6	5511.7	6049.3	5877.8	5762.2	6537.0	0.11	0.8081
Tcf20	5599.4	5417.9	4469.5	5038.1	4746.7	5441.0	-0.07	0.9528
Adam22	5598.4	4823.9	5065.0	4727.0	4742.6	5249.9	0.00	1.0000
Dot1l	5598.4	5505.3	4771.8	5402.2	4640.9	5934.6	-0.03	0.9848
Fem1a	5598.4	5085.3	4656.9	4813.0	4544.4	4938.3	-0.09	0.8690
Galnt1	5596.2	5443.4	5826.7	6015.8	5783.7	5589.5	0.08	0.8946
Reep5	5596.2	6280.6	7064.0	6648.4	6191.3	6740.8	0.05	0.9810
Ppp3cb	5595.2	5533.6	5430.7	5688.8	5527.0	5576.8	0.02	1.0000
Atp5c1	5592.0	5787.7	5867.0	5519.0	5794.0	4508.9	-0.12	0.8013
Map4	5590.9	5488.0	5902.2	6360.8	6013.7	7704.5	0.24	0.4186
Nptn	5590.9	5565.5	5622.1	5549.7	5880.2	5433.7	0.01	1.0000
Alg10b	5589.8	5667.5	5283.6	5345.9	5104.0	5511.6	-0.08	0.9057
Setd1b	5587.7	4575.2	4599.5	5144.2	4534.2	5573.2	0.11	0.8702
Jade1	5585.6	6196.8	5445.8	5698.4	5354.5	5702.7	-0.12	0.7514
Eif3d	5584.5	5521.7	4968.2	5496.7	5458.2	5299.7	-0.02	1.0000
Rps27rt	5580.2	6135.8	6725.4	5076.3	6150.3	4863.1	-0.17	0.6631
Igsf9b	5579.2	5470.7	4900.7	4532.8	3701.5	4997.2	-0.26	0.3583
Nell1	5569.6	5668.4	5965.7	6299.2	7315.6	5549.7	0.15	0.7623
Arih1	5568.5	5208.3	5292.7	5293.9	5432.6	5393.0	0.04	0.9884
Cdc37	5568.5	5624.7	5817.6	5723.8	5855.6	5408.4	0.01	1.0000
Rps24	5565.3	5989.1	5918.4	5625.1	5663.6	5107.7	-0.11	0.7682
Derl1	5563.2	5455.2	5068.0	4974.4	5422.3	4841.4	-0.09	0.8742
Crk	5562.1	5020.7	5035.7	5420.2	5455.2	5672.0	0.12	0.7817
Wipi2	5561.1	6282.4	6340.5	5543.4	5693.4	4949.1	-0.19	0.5550
Wac	5557.9	5110.9	5563.7	5835.3	5568.1	5422.0	0.11	0.7903
Ttyh3	5557.9	5512.6	5889.1	5544.4	5463.4	6796.0	0.09	0.9064
Pdcd4	5555.7	5419.7	5943.6	6290.7	6249.9	5636.6	0.14	0.7191
Gm12174	5554.7	5993.6	5951.6	6093.2	6460.3	5388.4	0.01	1.0000
Sec23ip	5550.4	4583.4	4346.6	5098.6	5054.7	5372.1	0.14	0.7805
Psmd1	5546.1	6220.5	5635.2	5934.0	5938.8	5878.5	-0.05	0.9263
Ipo5	5545.1	6003.7	5573.8	6995.6	6503.5	7152.9	0.19	0.4286
Dusp11	5545.1	5280.3	5187.9	5138.9	4873.0	5343.2	-0.04	0.9498
Zfand3	5544.0	5822.4	5752.1	5680.3	6030.1	5369.4	-0.02	1.0000
Xpo6	5543.0	5582.8	4945.1	5299.2	5185.1	5571.4	-0.04	0.9498
Ei24	5534.4	5560.9	5773.3	5276.9	5885.4	5083.2	-0.04	0.9831
Appbp2	5533.4	4767.4	4881.6	5522.1	5422.3	5253.5	0.15	0.6836
Tut4	5524.8	5526.3	5365.2	6044.4	5569.1	6067.8	0.09	0.8727
Rab5a	5522.7	5386.0	5617.1	5407.5	5525.0	4931.9	-0.03	0.9731
Rpl32	5522.7	6207.7	6673.0	6008.3	6433.7	5407.5	-0.05	1.0000
Mapk14	5522.7	5602.8	5223.2	5292.8	5177.9	5493.5	-0.06	0.9078
Amy2a5	5519.5	9198.6	11269.5	9685.5	7736.6	15073.0	0.11	0.9056
Rgs11	5517.4	5435.2	4839.3	4866.1	4491.0	4246.3	-0.23	0.3626
Dpp3	5516.3	5736.7	4966.2	4723.9	4792.9	4817.8	-0.23	0.2665
Mta2	5514.2	5537.2	5793.4	5593.3	5526.0	5369.4	-0.01	0.9967
Rpl31-ps8	5513.1	5929.9	6235.7	5486.0	6058.9	4795.2	-0.11	0.8314
Epb41l4b	5512.1	4980.6	5172.8	4675.0	5225.2	5034.3	-0.01	1.0000
Ankrd13a	5512.1	5649.3	5225.2	5382.0	5256.0	5294.2	-0.07	0.8714
Rab5b	5508.9	5491.7	5761.2	5348.0	5519.8	5206.4	-0.04	0.9877
Psmd3	5508.9	6022.8	5912.3	5711.1	5673.9	5047.9	-0.11	0.8031
Vamp3	5507.8	5337.7	5791.4	5239.8	5898.7	4917.4	-0.01	1.0000
Wnt4	5504.6	5744.9	6335.5	6173.9	6455.2	5840.4	0.08	0.9017
Prdm2	5504.6	5228.4	4700.2	5754.6	5040.3	5591.3	0.06	0.9281
Bcl2l2	5504.6	5249.3	5021.6	4716.4	5012.6	4194.6	-0.16	0.6666
Nucks1	5501.4	4488.6	5282.6	5383.1	5604.0	5538.8	0.24	0.1685
Foxo3	5501.4	5779.5	5837.8	5463.8	5290.9	5462.7	-0.09	0.8396
Gm10925	5498.2	5429.7	6057.4	5654.8	5810.4	5423.8	0.04	0.9877
Armcx4	5496.1	5343.2	5042.8	5911.7	5230.3	6513.4	0.13	0.7648
Zfp740	5493.9	5293.1	5446.8	5610.2	5376.1	5286.1	0.03	1.0000
Ptp4a3	5491.8	4986.0	5443.8	5165.5	5349.4	5246.2	0.06	0.9515
Irf2bpl	5487.5	5821.4	5483.1	5062.5	5382.3	5419.2	-0.12	0.7676
Mafg	5484.3	5760.4	5186.9	5401.1	5368.9	5262.5	-0.09	0.8264
Cdc42se2	5483.3	5426.1	5145.6	5172.9	5109.1	4741.7	-0.10	0.8329

Man2a1	5482.2	5048.0	5218.1	5637.8	5347.3	6153.8	0.15	0.6644
Vps13b	5481.1	5048.9	4326.4	5853.3	5031.1	6447.3	0.17	0.6955
Nploc4	5472.6	5446.1	5141.5	5366.1	5371.0	5601.3	0.00	1.0000
Aldh9a1	5472.6	5561.8	5919.4	4943.6	5340.2	5027.9	-0.12	0.7524
mt-Co3	5471.6	5255.7	6056.4	5601.8	5701.6	5056.0	0.03	1.0000
Adam10	5467.3	5579.1	6112.8	6068.8	5882.3	6112.2	0.09	0.8470
Kdm5a	5457.7	5447.9	4935.0	5677.1	5441.8	6025.2	0.07	0.9273
Gak	5457.7	5224.7	4824.2	5262.1	5196.4	5410.2	0.02	1.0000
Phc2	5452.4	5488.9	5795.4	5401.1	5466.4	5199.1	-0.04	0.9863
Erp44	5446.0	5707.6	5031.7	4923.4	5253.9	5075.0	-0.14	0.6875
Actr1a	5444.9	5203.8	5399.5	5297.1	5331.9	5428.3	0.03	0.9939
Mid1ip1	5441.7	5821.4	5795.4	5515.8	5466.4	4792.4	-0.13	0.7449
Nap1l1	5439.6	5052.5	5288.6	5386.3	5127.6	5261.6	0.04	0.9656
Rplp1	5432.1	6229.6	6746.6	6456.3	6628.7	5786.1	0.02	1.0000
Arfgap1	5432.1	5305.8	4486.6	5207.9	5037.3	4974.5	-0.04	0.9739
Cacna1c	5428.9	5078.1	4419.1	5911.7	5002.4	6329.5	0.16	0.6840
Ccar1	5427.9	4959.6	4889.7	5094.3	4846.3	4876.7	-0.01	1.0000
Scly	5425.7	4902.2	4765.7	4468.0	4823.7	4266.2	-0.11	0.7863
Arhgap1	5419.3	5191.9	5271.5	5416.0	5408.9	5575.0	0.06	0.9232
Nbas	5418.3	6098.4	5042.8	6363.9	6224.2	7033.3	0.12	0.7572
Calu	5414.0	5412.4	5737.0	6324.7	6235.5	7055.1	0.24	0.2595
Cpt1a	5414.0	5570.0	6155.1	6245.0	6083.5	6576.8	0.16	0.6155
Ddx1	5414.0	5668.4	5267.5	5676.1	5776.5	5304.2	-0.01	0.9988
Mecp2	5411.9	4924.1	4597.5	5378.8	4990.0	6036.1	0.13	0.7873
Atp5g3	5411.9	5638.3	6056.4	5793.9	6356.6	5733.5	0.07	0.9260
Clcn4	5409.8	5060.7	5102.2	4812.0	5268.3	5227.2	0.00	1.0000
Edem2	5407.6	6024.6	5713.8	5032.8	5559.9	5314.2	-0.16	0.6258
Derl2	5406.6	5734.9	5140.5	5175.0	5210.8	5275.2	-0.11	0.7503
Tmem94	5403.4	4815.7	4844.3	4989.2	4907.9	4962.7	0.03	1.0000
Ncoa6	5402.3	5106.3	5253.4	6011.5	5619.4	6468.1	0.21	0.3493
Lsm14a	5402.3	5189.2	5572.8	5439.3	5699.5	5274.3	0.06	0.9343
Capza2	5400.2	5223.8	5529.5	5452.1	5724.2	4960.0	0.03	0.9741
Lrp10	5400.2	5143.6	4765.7	5050.8	5267.3	5262.5	0.01	1.0000
Sdc3	5399.1	5234.7	5950.6	5671.8	5960.3	6487.1	0.17	0.5985
Tgfbr1	5398.0	5160.0	5307.8	5290.7	5162.5	5334.1	0.02	0.9834
Mapre3	5395.9	5157.3	5428.7	4660.2	4806.2	4626.7	-0.13	0.6967
Ppp1r15b	5394.8	4853.9	4840.3	5412.8	4991.1	5269.8	0.09	0.8461
Sox4	5391.6	5435.2	7142.5	6266.3	5974.7	6953.6	0.17	0.7075
Lrpprc	5390.6	5570.0	5540.5	5452.1	5137.9	5317.8	-0.06	0.9301
Ppp2r5b	5390.6	4741.9	5311.8	4344.9	4506.4	4287.0	-0.12	0.7038
Crocc	5390.6	5195.6	4789.9	4692.0	3880.1	4653.0	-0.21	0.5211
Snrk	5387.4	4668.1	5550.6	6325.7	6048.6	6479.9	0.37	0.0070
Arsb	5386.3	5393.3	5409.6	5756.7	5788.8	5447.3	0.07	0.8946
Gm28661	5383.1	5618.3	5726.9	5293.9	5618.4	5777.9	-0.01	1.0000
Ano6	5375.7	5046.2	5094.2	5806.6	5987.0	6147.5	0.22	0.2901
Gabarapl1	5372.5	5244.8	6335.5	4900.1	6032.2	4397.5	-0.06	0.9592
Brwd1	5367.1	5093.5	4391.9	5214.3	4497.2	5799.7	0.03	1.0000
Prkar1b	5367.1	5217.4	5658.4	4683.5	4702.5	4431.9	-0.17	0.5311
Aars	5365.0	5485.3	4946.1	5767.4	5601.0	6003.5	0.08	0.8816
Clip1	5361.8	5213.8	5128.4	5640.0	5328.9	5691.0	0.08	0.8507
Galnt10	5361.8	5511.7	5151.6	4714.3	4809.3	4893.9	-0.18	0.5016
Sp1	5359.7	4730.0	4756.7	5562.5	5225.2	5970.9	0.20	0.4336
Tspan2	5359.7	5150.9	5592.9	5155.9	5675.9	4835.0	0.01	1.0000
Ipo9	5358.6	5785.9	5135.5	6167.6	5811.4	6646.6	0.11	0.8049
Gatad2b	5353.3	5481.6	5551.6	6216.4	5961.3	6994.4	0.20	0.4648
Oip5os1	5349.0	5051.6	4920.9	5645.3	5304.2	6548.7	0.18	0.6115
Sp3	5348.0	5448.8	5141.5	5757.8	5468.5	5112.2	0.01	1.0000
Pccb	5345.8	5344.1	5440.8	5038.1	5572.2	4646.6	-0.07	0.9474
Gm10698	5345.8	5535.4	5098.2	5166.5	5393.5	4625.8	-0.11	0.8021
Casr	5343.7	4978.8	4269.0	5458.4	5359.7	5064.2	0.09	0.8727
Tial1	5343.7	5188.3	4766.7	5307.7	5166.6	5303.3	0.02	1.0000
Gpi1	5342.6	5163.7	5179.8	5291.8	5318.6	5470.9	0.05	0.9404
Ppfia1	5342.6	5193.8	4815.1	5342.7	5124.5	5667.4	0.05	0.9609
Ppp4r3b	5341.6	4948.7	4672.0	5399.0	5205.7	5104.9	0.07	0.8934
Grsf1	5341.6	5010.6	5380.3	5264.2	5547.6	5042.4	0.06	0.9498
Tut7	5339.4	5388.7	4874.5	5699.4	5136.9	5568.7	0.03	0.9764
Tpi1	5334.1	5360.5	5432.7	4996.7	5061.9	5090.4	-0.08	0.8506
Ufl1	5333.0	5306.7	5040.8	5243.0	4929.5	5013.5	-0.06	0.9162
Kmt5b	5327.7	5120.0	5151.6	5340.6	4864.8	5610.4	0.03	0.9679
Mrs2	5325.6	5035.2	4328.4	4928.7	4755.9	5283.4	0.00	1.0000
Dnajc7	5324.5	5376.0	5575.8	4955.3	5036.2	4739.9	-0.12	0.7470
Abcf1	5321.3	5342.3	5282.6	5560.4	5769.3	5901.1	0.10	0.7920
Cabp7	5320.3	4509.6	5166.7	3758.9	4006.4	3354.1	-0.28	0.1628
Ctdspl	5314.9	5089.9	5545.6	5320.4	5330.9	5743.5	0.08	0.8826
Nup98	5312.8	4959.6	4739.5	5864.0	5666.7	6238.1	0.23	0.3045
Unc13b	5311.7	5800.5	4845.3	4878.8	4294.9	5325.9	-0.21	0.4720

Jade3	5309.6	5233.8	5352.1	5614.5	5840.2	5634.8	0.11	0.7795
Cabin1	5308.5	5127.2	4897.7	5344.9	4926.4	5750.8	0.05	0.9377
Mkrn1	5308.5	5280.3	5591.9	5063.6	5555.8	4539.7	-0.06	0.9244
Sumo3	5308.5	5667.5	5512.3	5280.1	5978.8	4528.9	-0.08	0.8866
Prpf40a	5307.5	5188.3	5007.5	5452.1	5143.0	5470.9	0.04	0.9496
Cul1	5304.3	5551.8	5240.3	5181.4	5190.3	5074.1	-0.09	0.8037
Mybbp1a	5300.0	5497.1	4814.1	5952.1	5451.0	6104.0	0.09	0.8339
Srek1	5297.9	5088.1	4990.4	5861.8	4508.5	6150.2	0.10	0.8719
Khdrbs1	5297.9	5114.5	5208.0	5322.6	5306.3	5676.5	0.08	0.8919
Cnn3	5295.7	5143.6	5868.0	5302.4	5589.7	5365.8	0.06	0.9511
Mbnl1	5293.6	4878.5	4627.7	5668.6	5433.6	5909.3	0.19	0.4893
Nars	5291.5	5209.2	5033.7	5101.8	4805.2	5189.2	-0.04	0.9483
Appl2	5289.4	5130.9	5386.4	5010.5	4513.6	4941.0	-0.09	0.8429
Tpcn1	5288.3	5338.6	4725.4	5617.7	4857.6	5691.0	0.03	1.0000
Ube2j1	5286.2	4516.9	4560.2	4947.8	4646.1	4948.2	0.08	0.9126
Npdc1	5284.0	5645.6	5401.5	5365.0	5258.0	4833.2	-0.11	0.7992
Myh10	5279.8	5099.9	4847.3	5995.6	5644.1	7274.3	0.27	0.2820
Gm13050	5274.4	4875.8	5370.3	5034.9	5350.4	4997.2	0.05	0.9316
Bbx	5273.4	5288.5	5470.0	5479.7	5452.1	5415.6	0.04	0.9766
Nfkb1	5273.4	5027.0	4789.9	5045.5	5155.3	5042.4	0.01	1.0000
Helz	5270.2	4974.2	5058.9	5663.3	5072.2	6177.4	0.15	0.6860
Kif1c	5268.0	5369.6	5408.5	5776.9	5622.5	5978.1	0.10	0.8218
Zfp568	5267.0	5212.0	4531.0	5726.0	5096.8	5023.4	0.03	0.9792
Scyl1	5267.0	5252.1	4930.0	4981.8	4998.2	4474.5	-0.11	0.8247
Sept9	5264.8	5161.0	5089.1	5560.4	5837.1	6082.3	0.16	0.5985
Qsox1	5264.8	5744.9	5282.6	5785.4	5738.5	5349.5	-0.01	0.9919
Pitpnm3	5263.8	4560.6	3605.0	4161.2	3672.7	3864.0	-0.18	0.6986
Qdpr	5261.6	5516.3	6127.9	5692.0	6148.2	4933.7	0.01	1.0000
Tef	5259.5	4623.5	5066.0	5213.2	5276.5	5816.9	0.19	0.4260
Arf5	5255.3	5539.9	5345.1	4941.5	5311.4	4040.7	-0.18	0.6413
Ktn1	5249.9	5462.5	5107.3	6142.1	5855.6	6137.5	0.14	0.6576
Ywhaq	5248.9	5678.4	4825.2	5531.7	6177.0	5135.7	0.01	1.0000
Dcaf11	5248.9	5314.0	5294.7	5044.4	5005.4	4827.8	-0.09	0.8175
Ptpra	5247.8	4979.7	5389.4	5421.3	5296.0	4784.3	0.04	0.9884
Ttyh2	5246.7	4672.6	4272.0	4413.9	4542.4	4578.7	-0.05	0.9681
Robo2	5245.7	5847.0	5685.6	6356.5	5283.7	5778.8	0.00	1.0000
Kansl1	5243.5	4891.3	5200.0	5527.4	5444.9	5531.6	0.15	0.6241
Eif3h	5241.4	5109.0	5207.0	4744.0	5116.3	3769.8	-0.15	0.7589
Socs7	5240.3	4314.6	4730.5	4196.3	4415.0	4537.0	0.00	1.0000
Trim25	5240.3	5172.8	5436.8	4658.0	4460.2	4493.5	-0.18	0.4524
Tmem164	5239.3	5017.0	4820.1	4969.1	5183.1	5093.2	0.02	0.9923
Tmed2	5239.3	5518.1	4987.4	5119.8	5316.5	4441.9	-0.12	0.7948
Dvl3	5238.2	4761.0	4423.2	4939.4	4737.5	5268.9	0.06	0.9557
Ghrl	5236.1	4602.5	4712.3	2055.1	2499.1	1962.8	-0.96	0.0000
Cnot7	5233.9	5478.0	5578.8	5064.6	5104.0	4833.2	-0.12	0.7619
Man1a	5232.9	4738.2	4429.2	5125.1	5300.1	4747.2	0.08	0.8822
Sub1	5232.9	4887.6	5388.4	4653.8	5081.4	4095.0	-0.09	0.8520
Ppm1a	5230.7	5336.8	5660.4	5217.5	5510.6	5076.0	-0.02	0.9877
Gigyf1	5229.7	5058.9	5050.9	5003.0	4788.8	4904.8	-0.05	0.9649
Pbx1	5228.6	5023.4	5498.2	5147.4	5237.5	5669.2	0.07	0.9114
Dmxl1	5225.4	5245.7	4688.1	5181.4	4716.9	5739.9	0.00	1.0000
Arid1b	5224.4	4920.4	5014.6	6086.9	5449.0	6118.5	0.23	0.2820
Htt	5224.4	5265.7	4512.8	5888.4	4444.8	6133.9	0.07	0.9532
Copz1	5222.2	5754.0	5446.8	5335.3	5784.7	5554.2	-0.03	0.9850
Cbl	5216.9	4517.8	4294.2	5081.6	4509.5	6099.5	0.17	0.6907
Stx7	5216.9	4669.9	4827.2	4386.3	4656.3	4307.0	-0.08	0.8772
Papss1	5216.9	6195.0	6732.5	4793.9	5982.9	5831.4	-0.13	0.7873
Scap	5213.7	5427.9	5207.0	5127.2	4942.8	4872.2	-0.11	0.7648
Fam168b	5212.6	5101.7	5372.3	5779.0	5340.2	6043.3	0.14	0.6879
Scrn1	5212.6	5261.2	5378.3	5180.3	5455.2	5142.1	0.00	1.0000
Ylpm1	5211.6	4958.7	4460.4	5597.5	5154.3	6263.4	0.18	0.6240
Zdhhc20	5211.6	4782.0	4849.4	4650.6	4604.0	4491.7	-0.06	0.9343
Gm4204	5207.3	5078.1	5052.9	5287.5	5437.7	5187.4	0.06	0.9500
Ppig	5206.2	5079.0	5177.8	5360.8	5588.6	5260.7	0.08	0.8548
Trp53i11	5204.1	5030.7	5521.4	4881.0	5085.5	4790.6	-0.04	0.9835
Vstm2l	5202.0	5231.1	5800.5	5126.2	5772.4	4243.5	-0.06	0.9729
Zranb1	5200.9	4486.8	5129.5	4750.4	5523.9	4736.3	0.11	0.8207
Oaz2	5200.9	5048.9	5650.4	4736.6	5547.6	4447.3	-0.05	0.9477
Fbrs	5200.9	5153.7	5323.9	4898.0	4774.4	5140.3	-0.06	0.9132
Smg7	5199.8	5079.0	4841.3	5412.8	5414.1	6027.0	0.13	0.7319
Szrd1	5197.7	4660.8	5251.4	4735.5	5083.5	4379.4	0.00	1.0000
Nbeal2	5196.7	3934.7	4618.6	5026.4	4527.0	4990.8	0.23	0.3300
Pdzd8	5194.5	5158.2	4920.9	5243.0	4993.1	5469.1	0.02	0.9820
Dlg5	5192.4	5176.4	5457.9	5199.4	4591.7	5447.3	-0.03	0.9948
Tvp23b	5188.1	5173.7	5200.0	4557.2	5531.1	4609.5	-0.07	0.9395
Rlim	5182.8	4412.1	4085.6	5204.7	4928.4	5104.9	0.17	0.6582

Farp1	5182.8	5249.3	4825.2	5780.1	5130.7	6393.9	0.13	0.7535
Zc3h4	5182.8	5136.4	4969.3	5369.3	4959.2	5572.3	0.04	0.9711
Becn1	5181.7	5121.8	5218.1	5003.0	5276.5	4744.4	-0.03	0.9966
Mta1	5179.6	5369.6	5698.7	5888.4	5711.8	5698.2	0.09	0.8510
Ankrd44	5175.3	5429.7	5504.3	5597.5	5211.8	5873.9	0.03	0.9672
Prkcb	5172.1	5220.2	4924.9	5352.3	4967.4	5323.2	0.00	1.0000
Suds3	5172.1	4979.7	5275.5	4981.8	4955.1	4531.6	-0.05	0.9649
Crtc1	5172.1	4951.4	4401.0	4526.4	4371.9	5113.1	-0.07	0.9152
Larp4	5167.9	4403.9	4334.5	5117.7	4529.0	4803.3	0.10	0.8719
Dhx30	5162.6	5570.0	5319.9	5411.7	4985.9	5134.8	-0.09	0.8632
Skp1a	5161.5	5200.1	5602.0	5177.1	5527.0	4863.1	-0.01	1.0000
Rsf1	5158.3	5207.4	4993.4	5713.2	5106.1	5481.7	0.06	0.9399
Zfp706	5158.3	4992.4	5469.0	5086.9	5079.4	5099.5	0.02	1.0000
Rsrc2	5156.2	5155.5	5254.4	5326.8	5286.8	4999.9	0.01	1.0000
Foxa2	5150.8	5147.3	5116.4	4401.2	5091.7	3967.3	-0.17	0.6074
Luc7l	5148.7	5540.9	5339.0	5655.9	4959.2	5283.4	-0.05	0.9624
Stub1	5147.6	5620.1	5739.0	5747.2	5829.9	4864.0	-0.03	0.9818
Tshz2	5144.4	4843.9	5582.9	5115.6	5557.8	5917.4	0.15	0.6312
Flii	5143.4	4830.3	4640.8	4896.9	5027.0	5200.0	0.05	0.9592
Tox3	5143.4	5238.4	5185.9	5699.4	5596.8	5018.0	0.05	0.9662
Tmem259	5142.3	5353.2	5551.6	5307.7	5513.7	4585.9	-0.05	0.9690
Tprgl	5142.3	5163.7	5319.9	4620.9	5174.8	4217.3	-0.13	0.7795
Scrib	5142.3	5254.8	4581.3	4671.8	4345.2	4573.2	-0.18	0.5407
Wdr90	5142.3	6162.2	4251.9	5414.9	4423.3	5115.8	-0.21	0.4982
Cep170	5141.2	4744.6	4614.6	5347.0	5363.8	5782.5	0.19	0.4632
Ddx46	5138.0	5127.2	4606.5	5243.0	5083.5	5654.7	0.06	0.9534
Syncrip	5137.0	4836.6	4863.5	5521.1	5248.8	5810.5	0.17	0.5557
Zmym2	5129.5	4990.6	5097.2	5416.0	5273.4	5629.4	0.11	0.7896
Atp13a1	5123.1	5169.2	4597.5	5185.6	4574.2	4929.2	-0.06	0.9496
March7	5121.0	4814.8	4900.7	4792.9	4462.3	4749.9	-0.05	0.9681
Cotl1	5117.8	5605.5	5247.3	4754.6	4938.7	4372.2	-0.22	0.3197
Sh3bgrl	5115.7	4946.9	5315.8	5205.8	5431.5	5097.7	0.07	0.9230
Smarca1	5114.6	5283.0	4843.3	4952.1	4710.8	4305.1	-0.15	0.6670
Slc40a1	5113.5	4546.0	5720.9	4374.6	4794.9	4797.0	0.00	1.0000
Tmtc3	5112.5	4065.0	3510.3	4548.7	4619.4	4598.6	0.13	0.7896
Akap8l	5109.3	4972.4	4977.3	4598.6	4151.2	4236.3	-0.19	0.4848
Dcaf5	5108.2	5098.1	5150.6	4917.1	5184.1	4742.6	-0.04	0.9831
Erlin2	5106.1	5033.4	4941.0	4384.2	4844.2	4585.9	-0.12	0.7494
Rtl5	5105.0	5099.0	4688.1	4201.6	4384.2	4137.6	-0.24	0.2563
Cd200	5101.8	4915.9	4991.4	4884.2	5408.9	4695.5	0.02	1.0000
Raly	5101.8	4910.4	5115.3	5106.0	5253.9	4518.0	0.01	1.0000
Aff1	5100.8	4668.1	5018.6	5400.1	4835.0	5870.3	0.16	0.6120
Taf1	5097.6	4823.9	4110.8	5240.8	5015.7	5825.0	0.14	0.7274
Slc30a9	5093.3	4879.4	4887.6	4588.0	4783.7	4585.0	-0.07	0.9196
Grina	5092.2	4650.8	5124.4	4462.7	4649.2	4595.9	-0.04	0.9559
Anpep	5090.1	5658.4	4882.6	5883.1	5398.7	6124.8	0.05	0.9299
Sesn2	5089.0	4857.6	4865.5	4644.2	5039.3	4170.2	-0.07	0.9395
Mlxip	5086.9	4415.7	4146.1	4484.0	4443.8	5050.6	0.06	0.9377
mt-Atp6	5084.8	5208.3	5827.7	5421.3	5504.4	5154.8	0.03	1.0000
Cldn4	5081.6	5486.2	5516.4	4248.3	5088.6	3793.4	-0.27	0.2677
Mpzl1	5078.4	5413.3	5678.6	5259.9	5286.8	5082.3	-0.05	0.9662
Gm47283	5070.9	2645.6	4805.0	453.3	1015.5	1727.3	-0.50	0.0629
Amotl1	5067.7	5465.2	5052.9	6370.3	5887.4	7456.3	0.25	0.2814
Pitpnm1	5064.5	5128.2	4194.4	4054.0	3971.5	3950.1	-0.30	0.1177
Eif3l	5063.5	5406.9	5004.5	5281.2	5201.5	5472.7	-0.01	1.0000
Dock7	5062.4	4939.6	4871.5	5044.4	4746.7	5724.5	0.06	0.9516
Abca5	5060.3	4383.9	4693.2	5308.8	4940.7	5289.7	0.20	0.4006
Stim1	5058.1	5040.7	4419.1	4728.1	5444.9	4974.5	0.01	1.0000
Sgpp1	5058.1	4767.4	4829.2	4822.6	4955.1	3941.0	-0.06	0.9340
Cry2	5057.1	4731.0	4467.5	4865.0	4766.2	5078.7	0.05	0.9507
Clk1	5056.0	4625.3	4529.0	6152.7	5453.1	6316.9	0.32	0.0700
Ndrg3	5054.9	4670.8	4971.3	5056.1	5179.0	4838.6	0.09	0.8395
Myo1c	5048.5	4691.8	4346.6	5270.6	5088.6	5720.9	0.17	0.6172
St5	5045.3	4987.9	5304.8	5038.1	5097.8	5402.9	0.04	0.9752
Sin3a	5045.3	5239.3	4677.1	5383.1	4876.1	5242.6	-0.01	1.0000
Pard3	5044.3	5059.8	4182.4	3860.8	3910.9	3936.5	-0.32	0.0852
Klf13	5041.1	4508.7	4834.2	5280.1	5265.2	5726.3	0.23	0.2814
Hif1an	5037.9	4812.0	4841.3	4389.5	4387.3	4597.7	-0.11	0.7809
Bod1l	5033.6	4595.2	4956.2	5695.2	5389.4	6094.0	0.28	0.1308
C77080	5030.4	4764.7	4849.4	4754.6	5004.4	4383.9	-0.02	1.0000
Asxl2	5029.4	4900.4	4416.1	5301.3	4479.7	5398.4	0.05	0.9785
Lrp5	5028.3	5394.2	5730.0	5411.7	5120.4	6121.2	0.04	0.9676
2510009E07R	5027.2	4756.5	5127.4	4676.1	4519.8	4976.3	-0.02	1.0000
Mageh1	5027.2	4879.4	4881.6	4800.3	5218.0	3921.1	-0.07	0.9559
Tsnax	5027.2	4901.3	4845.3	4350.2	4872.0	3980.9	-0.14	0.7514
Arap1	5025.1	5661.1	5049.9	4923.4	5357.6	5212.7	-0.10	0.7873

Dusp3	5024.0	4533.3	4388.9	5322.6	5393.5	5598.6	0.23	0.3035
Tnfaip1	5023.0	4437.6	4677.1	4739.8	5021.9	5306.9	0.15	0.6876
Man1a2	5021.9	4842.1	4706.3	4878.8	4589.6	5061.5	0.00	1.0000
Klhdc2	5019.8	4443.1	5100.2	4578.4	4854.5	3866.7	-0.03	1.0000
Ankhd1	5018.7	4695.4	3974.8	4935.1	4403.8	5694.6	0.09	0.9178
Zwint	5014.4	5087.2	5336.0	5089.0	5335.0	4605.0	-0.02	1.0000
Rnh1	5014.4	5326.8	5426.7	4855.5	4998.2	4198.3	-0.16	0.6560
Atrn	5012.3	4833.0	4754.6	5088.0	4926.4	5610.4	0.10	0.8633
Stx5a	5012.3	5594.6	5080.1	4701.6	4682.0	4992.6	-0.19	0.4336
Epb41l1	5010.2	4120.6	4568.2	4390.5	4423.3	5232.7	0.13	0.7517
Hexa	5008.0	4809.3	5107.3	4596.5	5308.3	4105.9	-0.05	0.9522
Arhgef2	5005.9	5271.2	4844.3	5486.0	5387.4	5769.8	0.08	0.8619
Kcnma1	5005.9	4520.5	4423.2	4800.3	4479.7	4960.0	0.06	0.9343
Vapa	5004.9	5222.9	5393.4	4782.2	5033.2	4576.9	-0.11	0.7767
Pck2	5004.9	5183.7	5454.9	4751.5	4975.7	4461.8	-0.12	0.7821
Ik	5001.7	5004.3	4849.4	5012.6	5058.8	5283.4	0.03	0.9933
Gm6204	5001.7	5367.8	6233.7	5090.1	5670.8	3848.6	-0.13	0.8398
Anxa7	5000.6	4364.7	4607.5	4326.8	4614.2	4165.7	-0.02	1.0000
Ahnak	4999.5	4544.2	4426.2	6462.7	5621.5	10493.4	0.35	0.4177
Dnaja2	4998.5	4499.6	4851.4	4698.4	5086.5	4557.9	0.06	0.9334
5031439G07R	4997.4	4876.7	5112.3	4477.6	4359.6	4573.2	-0.12	0.7192
Pigt	4996.3	5287.6	4963.2	4584.8	5041.4	4924.7	-0.10	0.8247
Capn7	4996.3	4587.9	4408.0	4307.7	4236.4	4108.6	-0.11	0.8011
Rraga	4994.2	4526.9	4944.1	4659.1	5020.8	4244.5	0.01	1.0000
Tbc1d22b	4994.2	4976.9	4491.7	4625.1	4364.7	4441.9	-0.13	0.7470
Cdc42bpg	4994.2	4887.6	4485.6	4149.6	4008.5	4403.0	-0.20	0.4708
Psmb4	4989.9	5347.7	5468.0	5141.0	5488.0	4520.7	-0.07	0.9458
Ccdc47	4988.9	4703.6	4565.2	4607.1	4899.7	4914.7	0.03	0.9807
Kdm5b	4987.8	4988.8	5069.0	5411.7	5425.4	5646.6	0.13	0.6860
Smc6	4986.7	5340.4	4807.0	5577.3	5430.5	5403.8	0.05	0.9694
Immt	4985.7	4884.0	5116.4	5421.3	5144.0	5274.3	0.10	0.8340
Apoe	4983.5	4991.5	7853.9	7507.2	7438.8	7688.2	0.41	0.0658
Tmem245	4983.5	4569.7	4354.6	5049.8	4203.5	5945.5	0.12	0.8540
Pxdn	4982.5	5357.7	5363.2	6878.8	6668.8	9311.3	0.44	0.0251
Prkcsh	4981.4	4925.9	4762.7	5386.3	5382.3	5189.2	0.10	0.8124
Ppp1r12a	4980.3	4899.5	4988.4	5722.8	5273.4	6165.6	0.20	0.4654
Clptm1	4978.2	5058.9	4714.3	5101.8	5056.8	4879.4	-0.01	1.0000
Cog6	4978.2	4768.3	4077.6	4516.9	4525.9	4115.8	-0.10	0.8455
Map3k2	4977.1	4727.3	4356.7	4904.3	4678.9	5363.1	0.07	0.9042
Pdap1	4976.1	5558.2	5670.5	5339.6	5510.6	4992.6	-0.06	0.9554
Cog1	4975.0	4883.1	4816.1	5089.0	5085.5	4604.0	0.01	1.0000
Ergic1	4975.0	5249.3	4966.2	5195.2	5052.7	4907.5	-0.04	0.9486
Btg1	4972.9	4934.1	5765.2	6419.1	6530.2	6042.4	0.31	0.0753
Usp5	4970.8	5237.5	5027.7	5249.3	4791.9	4749.0	-0.07	0.8864
Skiv2l	4970.8	5036.1	4684.1	4674.0	4712.8	4493.5	-0.11	0.7811
Ahctf1	4969.7	4252.7	4134.0	5102.8	4431.5	5133.9	0.16	0.6839
Setx	4968.6	5032.5	4164.2	5027.5	4554.7	5691.9	0.04	0.9884
Ankrd13c	4968.6	4946.9	4817.1	4750.4	4629.6	4185.6	-0.12	0.7636
Ctdsp1	4967.6	5191.9	5036.8	4633.6	5058.8	4783.4	-0.09	0.8314
Irgq	4964.4	4346.5	4165.2	4025.4	4038.2	4340.5	-0.07	0.9237
Pgap1	4963.3	4511.4	3708.8	5285.4	5022.9	5606.7	0.21	0.5386
Atf2	4962.2	4814.8	4621.6	5076.3	5236.5	5131.2	0.09	0.8316
Ahcyl2	4962.2	4244.5	4640.8	4072.1	4611.2	4150.3	-0.01	0.9966
Syt14	4961.2	4739.2	4244.8	5299.2	4775.4	5000.8	0.08	0.9126
Znfx1	4960.1	4828.4	4554.1	5141.0	5331.9	5260.7	0.11	0.7953
Gspt1	4960.1	4935.9	4744.6	5161.2	4997.2	5040.6	0.04	0.9794
Lrpap1	4958.0	5021.6	4954.1	4705.8	5223.1	4478.1	-0.06	0.9521
Prmt1	4956.9	5449.8	5092.2	4978.6	5236.5	4445.5	-0.13	0.7764
Ahdc1	4955.8	4640.8	4979.3	5257.8	5114.3	5469.1	0.16	0.5417
Creld2	4952.6	5955.4	4352.6	4198.4	4209.7	4058.8	-0.43	0.0022
Nup210	4950.5	5386.9	4668.0	5617.7	5039.3	5642.1	0.03	0.9694
Atf6b	4950.5	5372.3	4899.7	4822.6	5232.3	4630.3	-0.11	0.7831
Sbf1	4950.5	4790.2	4433.2	4325.8	3857.5	4403.9	-0.17	0.5895
Cs	4949.4	4531.4	4583.4	5143.2	4995.2	5366.7	0.17	0.5873
Chka	4947.3	4696.3	4291.2	4728.1	3910.9	4959.1	-0.04	0.9662
Ctsf	4943.1	4659.9	5070.0	3869.3	4962.3	3818.7	-0.14	0.7812
Atg13	4938.8	5418.8	5244.3	4606.0	4824.7	4695.5	-0.18	0.4727
Uba5	4937.7	4931.4	4141.0	4142.1	4105.0	4238.1	-0.20	0.4689
Poldip3	4936.7	4962.4	5202.0	5133.6	5185.1	4795.2	0.02	1.0000
Ranbp3	4936.7	5081.7	4752.6	5032.8	4922.3	4540.6	-0.06	0.9457
Ankfy1	4935.6	4693.6	4485.6	5079.5	4466.4	5239.0	0.06	0.9516
Srprb	4932.4	5080.8	4466.5	4471.2	4481.8	4405.7	-0.16	0.5914
Wasf2	4926.0	4495.9	4856.4	5744.0	5252.9	6512.5	0.32	0.0976
Ptpn1	4926.0	4577.0	4782.9	3990.3	4381.2	4318.7	-0.11	0.7619
Plpp5	4926.0	5251.1	4475.5	4260.0	4474.6	4225.4	-0.24	0.2572
Nf1	4923.9	4526.9	4146.1	5410.7	4977.7	6300.6	0.26	0.3061

Pdha1	4923.9	4884.9	5055.9	4822.6	5405.9	5124.9	0.06	0.9244
Map2k2	4923.9	5196.5	5044.8	4933.0	4774.4	4477.2	-0.12	0.7442
Zdhhc5	4922.8	4874.0	4658.9	4680.3	4695.4	4746.3	-0.04	0.9731
Vars	4919.6	5422.4	4889.7	5631.5	5434.6	5764.3	0.06	0.9402
Anks1	4914.3	4891.3	4448.3	4780.1	4759.0	4792.4	-0.02	0.9831
Dlg1	4913.2	4992.4	4776.8	4813.0	4864.8	4549.7	-0.06	0.9303
Vps4b	4912.2	4471.3	4756.7	4487.1	4927.4	4676.5	0.05	0.9332
Nol4	4912.2	4447.6	4523.9	4467.0	4783.7	3965.5	-0.02	1.0000
Trappc10	4912.2	4879.4	4377.8	4536.0	4427.4	4547.9	-0.10	0.8518
Ncald	4910.0	4482.2	4045.3	4362.9	4918.2	3912.0	-0.02	1.0000
Psmb1	4906.8	5395.1	5086.1	4664.4	5035.2	3830.5	-0.21	0.5343
Gm9616	4904.7	5181.0	5700.7	5508.3	5844.3	5728.1	0.12	0.7873
Atp6v1c1	4900.4	4498.6	4596.5	4205.8	4706.6	4018.0	-0.07	0.9457
Akap8	4899.4	4621.6	4276.1	5019.0	4570.1	5228.1	0.09	0.8874
Rcc2	4899.4	5294.9	5086.1	5593.3	5141.0	5564.2	0.04	0.9681
Agap3	4895.1	5007.9	4892.7	4668.7	4655.3	4597.7	-0.10	0.8304
Myo1b	4890.8	4986.0	4970.3	5230.2	5612.2	5564.2	0.12	0.7406
Psd3	4885.5	4232.6	4187.4	4371.4	4003.3	4699.2	0.02	0.9857
Usp4	4885.5	5076.2	4756.7	4928.7	4868.9	5367.6	0.00	1.0000
Pom121	4883.4	4733.7	4357.7	5084.8	4859.6	5483.6	0.11	0.7865
Qsox2	4880.2	4598.9	4549.1	4324.7	4455.1	4177.4	-0.09	0.8705
Smad4	4878.1	4574.3	4424.2	4580.6	4573.2	4883.0	0.03	0.9804
Fbxo11	4878.1	4659.9	5004.5	4849.1	4669.7	4784.3	0.02	1.0000
U2surp	4873.8	4577.0	4489.7	4808.8	4881.2	4893.0	0.08	0.9096
Tasor	4872.7	4615.3	4409.1	5059.3	4717.9	5186.5	0.10	0.8473
Neat1	4871.7	4537.8	4572.3	4964.8	4242.6	5195.5	0.06	0.9525
Suz12	4869.5	4860.3	4733.5	4517.9	4430.5	4543.4	-0.10	0.8207
Ddx24	4865.3	4606.1	4652.9	5124.1	5207.7	4919.3	0.13	0.7074
Anp32b	4864.2	4897.7	5038.8	5227.0	5307.3	4999.9	0.07	0.8762
Rabac1	4864.2	4641.7	5031.7	4296.1	4935.6	3481.8	-0.13	0.8314
Traf3	4863.1	4561.5	4211.6	4663.4	4492.1	4991.7	0.04	0.9542
Ube2r2	4862.1	4760.1	5473.0	4635.8	4883.3	4575.1	-0.03	0.9715
Coro2b	4858.9	5181.0	4970.3	5625.1	5359.7	5700.0	0.10	0.8213
Gm13680	4858.9	5134.5	5746.1	5125.1	5649.2	5231.7	0.04	0.9557
Zdhhc3	4855.7	4820.2	4841.3	4976.5	4667.6	4839.5	0.00	1.0000
Opa1	4854.6	4794.7	4628.7	4844.9	4442.8	5165.6	0.01	1.0000
Cflar	4853.5	4550.6	4164.2	4270.6	3939.7	4681.9	-0.07	0.9422
Ate1	4852.5	4500.5	4458.4	4642.1	4636.8	4335.0	0.01	1.0000
Rc3h1	4851.4	4314.6	4530.0	5283.3	4797.0	5270.7	0.21	0.3907
M6pr	4849.3	5157.3	4694.2	4891.6	5097.8	4581.4	-0.07	0.9053
Wdr82	4847.2	4347.4	4493.7	4999.9	4876.1	4565.1	0.12	0.7795
Nr1d2	4846.1	4941.4	4218.6	5233.4	5767.3	5661.1	0.17	0.6143
Man2b1	4845.0	4527.8	4514.8	4948.9	4932.5	5077.8	0.12	0.7546
Sgpp2	4845.0	4368.4	4205.5	4336.4	4278.5	4289.7	-0.03	0.9988
Pitpnb	4845.0	5282.1	5247.3	5145.3	5048.6	4933.7	-0.05	0.9551
Atp11a	4842.9	5136.4	4180.3	4618.8	4357.6	5633.9	-0.03	1.0000
Efcab14	4841.8	4549.7	4682.1	4792.9	4849.4	4730.9	0.06	0.9376
Rufy3	4841.8	4791.1	4998.5	5080.5	4940.7	4891.2	0.04	0.9649
Hbp1	4838.6	5291.2	5160.7	4212.2	4747.7	4106.8	-0.24	0.2595
Ghitm	4837.6	4566.1	5028.7	4816.2	5030.1	4661.1	0.06	0.9395
Gm11808	4837.6	5748.6	5649.3	4969.1	5582.5	4678.3	-0.14	0.7544
Cdc42se1	4836.5	4442.2	4863.5	4047.7	4400.7	3822.4	-0.12	0.7427
Fam13b	4836.5	4025.8	3893.2	3840.7	3574.1	3740.8	-0.12	0.7896
Fxr1	4831.2	4285.5	4688.1	4537.0	4499.2	4172.0	0.02	1.0000
Tie1	4830.1	4850.3	4592.4	5803.4	5886.4	6624.8	0.31	0.0848
Polr2b	4830.1	4352.9	4165.2	4485.0	4763.1	4744.4	0.08	0.8639
Sowahc	4829.0	4318.3	4495.7	4114.5	4368.8	3928.3	-0.07	0.9314
Rnf40	4828.0	4526.9	4510.8	5152.7	4688.2	4733.6	0.09	0.8472
Mtmr7	4826.9	4663.5	4727.4	4707.9	4763.1	4481.8	-0.01	1.0000
Tbl2	4825.8	4446.7	3532.5	4126.2	4009.5	4468.2	-0.05	0.9681
Gm10073	4823.7	5532.7	6061.4	5539.1	5958.3	5089.5	0.00	1.0000
Supt16	4818.4	4739.2	4883.6	5052.9	4977.7	5547.0	0.12	0.7896
Qk	4816.3	4380.2	4667.0	5064.6	5005.4	5883.0	0.24	0.3265
Mical2	4816.3	5196.5	4440.3	5391.6	4699.5	5471.8	0.02	1.0000
Dnajc14	4816.3	4637.1	4687.1	4547.6	4891.5	4372.2	-0.01	1.0000
Kif13b	4815.2	4744.6	4590.4	5044.4	5059.9	5473.6	0.12	0.7702
Tmem263	4815.2	4098.7	3876.1	4085.9	4127.6	3835.9	-0.04	0.9649
Dld	4814.1	4505.0	4427.2	4222.8	4691.2	4096.8	-0.05	0.9343
Oxct1	4812.0	4554.2	4806.0	5450.0	6048.6	5126.7	0.25	0.2751
Pnn	4808.8	4930.5	4654.9	5831.0	4672.8	6144.8	0.16	0.7161
Keap1	4808.8	4546.9	4226.7	3951.1	4514.6	4227.2	-0.09	0.8868
Tfrc	4806.7	5404.2	5392.4	7160.1	5046.5	5616.7	0.13	0.7896
Uba2	4804.5	5102.7	5085.1	5010.5	5123.5	5114.9	0.00	1.0000
4931406P16R	4803.5	4587.0	4856.4	5051.9	4845.3	5737.2	0.16	0.6666
Skil	4801.3	4362.0	4408.0	4550.8	3944.8	4709.1	0.00	1.0000
Map3k7	4800.3	5202.9	4492.7	4726.0	4493.1	4951.0	-0.11	0.8204

Rpl7	4797.1	5174.6	5413.6	5443.6	5527.0	4827.8	0.03	1.0000
Trim56	4796.0	4307.3	4093.7	4890.5	4662.5	5307.8	0.17	0.5855
Eaf1	4796.0	4600.7	4629.7	4695.2	4619.4	4242.6	-0.03	0.9778
Igsf1	4794.9	6460.1	4406.0	5977.5	6001.4	4873.1	-0.11	0.8529
Zyg11b	4792.8	4064.1	4333.5	4825.8	4620.4	4962.7	0.20	0.4441
Tpm3	4792.8	4615.3	4450.4	4901.1	4771.3	4939.2	0.07	0.9114
Nt5c3	4792.8	5053.5	5194.9	4885.2	5161.5	4045.2	-0.09	0.8726
Erp29	4791.7	4994.2	5069.0	4544.5	4870.9	4043.4	-0.14	0.7255
Syne1	4784.3	4843.9	4110.8	5397.9	4524.9	6104.0	0.14	0.7873
Rnf44	4782.2	4410.3	4673.0	4654.9	4664.6	4828.7	0.08	0.8934
Slc35e1	4782.2	4640.8	3821.6	4326.8	4285.7	4683.8	-0.04	0.9629
Gm4149	4782.2	5439.7	5903.3	5184.6	5747.8	4812.4	-0.04	0.9895
Rbm15b	4782.2	4778.3	4175.3	4044.5	3693.2	3945.5	-0.25	0.2424
Usp8	4777.9	4659.0	4323.4	4659.1	4693.3	4672.9	0.01	1.0000
Rock2	4774.7	4807.5	4714.3	5643.2	5036.2	6271.6	0.21	0.4724
Sod2	4773.6	4221.7	4585.4	4459.5	4666.6	4412.0	0.07	0.8816
Swi5	4773.6	4888.6	4624.7	4614.5	4585.5	4107.7	-0.12	0.7768
Mindy2	4772.6	4362.9	4314.3	4743.0	4195.3	4381.2	0.02	0.9931
Mfn2	4771.5	4715.5	4580.3	4643.2	4979.8	5085.9	0.05	0.9314
Prkca	4769.4	4237.2	3920.4	5118.8	4895.6	5231.7	0.23	0.3426
Lmtk2	4765.1	4289.1	4655.9	4743.0	4480.8	4802.4	0.10	0.8276
Atp6v0e2	4763.0	4685.4	5143.6	4508.4	5294.0	4729.9	0.03	0.9695
Gde1	4758.7	4582.5	4538.0	4482.9	4896.6	4153.9	-0.02	1.0000
Thoc2	4757.6	5057.1	4669.0	5262.1	5123.5	5638.4	0.08	0.8868
Camsap2	4756.6	4375.7	4719.4	4859.7	4369.9	5209.1	0.11	0.8292
Far1	4756.6	4811.1	4640.8	4764.2	4660.4	4874.0	-0.01	1.0000
Chmp4c	4754.4	3389.9	4270.0	3952.1	4398.6	3738.1	0.16	0.6708
Cdhr1	4753.4	5013.4	4638.8	4815.2	5358.6	5233.6	0.04	0.9715
Plcg1	4752.3	4732.8	4895.7	5219.6	4937.7	5660.2	0.14	0.6871
Pacc1	4751.3	5340.4	4855.4	4884.2	5236.5	5032.5	-0.06	0.9155
Chp1	4745.9	4566.1	5065.0	4748.3	4900.7	4541.5	0.03	0.9925
Scpep1	4743.8	4075.9	4197.5	4358.7	4849.4	4724.5	0.15	0.6422
Atp6v1h	4743.8	4728.2	4750.6	4527.5	4832.9	4192.8	-0.06	0.9455
Erap1	4742.7	4577.9	4440.3	4827.9	4881.2	4606.8	0.05	0.9263
Sidt2	4742.7	4567.9	4671.0	4555.1	4590.6	5130.3	0.05	0.9468
Cggbp1	4738.5	4329.2	4290.2	4778.0	4662.5	5040.6	0.14	0.7319
Nxf1	4737.4	4897.7	4534.0	4721.7	4018.7	4768.9	-0.10	0.8396
Rab3gap1	4736.3	4608.9	4355.7	4889.5	4898.7	4811.5	0.07	0.9051
Sltm	4735.3	4751.9	4303.3	4885.2	4427.4	4778.0	-0.01	1.0000
Pitpna	4734.2	4503.2	4467.5	4427.7	4767.2	4654.8	0.03	0.9731
Myadm	4733.1	4358.3	4805.0	5075.2	5091.7	5854.0	0.25	0.2398
Snx4	4729.9	4569.7	4585.4	4118.8	4360.6	3631.2	-0.16	0.6552
Tes	4727.8	4229.0	3766.2	3813.1	3920.2	3211.0	-0.19	0.6350
Fyttd1	4722.5	4359.3	4114.8	4477.6	4190.2	4082.3	-0.04	0.9942
Abce1	4720.4	4508.7	4026.2	4781.2	4549.6	4897.5	0.07	0.9334
Pfkfb2	4717.2	4167.0	3608.0	4605.0	4051.6	4567.8	0.07	0.9515
Gpsm1	4717.2	4593.4	4461.4	3994.6	4201.5	3983.6	-0.16	0.5751
Enpp5	4712.9	4477.7	4786.9	4473.3	4957.2	4398.4	0.03	0.9748
Oxr1	4711.8	4439.4	4688.1	4124.1	5001.3	4255.3	-0.01	1.0000
Trim41	4707.6	4888.6	4827.2	4222.8	4243.6	3828.7	-0.23	0.3019
Abcc5	4706.5	5051.6	4689.2	4755.7	4225.1	4722.7	-0.12	0.7795
Zranb2	4703.3	4782.0	4603.5	4381.0	4199.4	4288.8	-0.14	0.6784
Crebbp	4699.0	4270.0	4548.1	5482.9	4754.9	5804.2	0.27	0.2062
Tasor2	4699.0	4575.2	4014.1	4835.3	4321.6	4825.1	0.03	0.9991
Psmc2	4699.0	4867.6	4841.3	4874.6	4897.6	4451.9	-0.03	0.9893
Gm15772	4693.7	5290.3	5623.2	4986.1	5161.5	4435.6	-0.10	0.8316
Slc25a44	4693.7	4257.2	4105.8	4013.7	4036.2	3704.6	-0.11	0.7811
Mtmr4	4691.6	4359.3	3825.7	3958.5	3619.3	4028.9	-0.15	0.7038
Hdac2	4690.5	4870.3	4821.1	4749.3	4971.6	4625.8	-0.02	1.0000
Shoc2	4690.5	4543.3	4495.7	4317.3	4242.6	3982.7	-0.11	0.7982
Cmtm6	4689.5	4304.6	4805.0	4243.0	4508.5	3859.5	-0.05	0.9649
Rcbtb1	4688.4	4343.8	4117.9	4312.0	4341.1	4390.3	0.00	1.0000
Nrp1	4687.3	4572.4	5063.0	6234.4	6006.5	7133.9	0.43	0.0041
Rhobtb1	4684.1	4405.7	3848.9	4664.4	4659.4	4523.4	0.07	0.9382
Arfip2	4684.1	4790.2	4539.0	4278.0	4070.1	3835.9	-0.21	0.3386
Ppfibp1	4682.0	4747.4	4248.9	5176.1	4956.2	5181.0	0.11	0.7845
Mast2	4679.9	5108.1	3965.7	5823.6	4631.7	5784.3	0.11	0.8240
Parp1	4679.9	4836.6	4633.7	5056.1	4874.0	5264.4	0.07	0.9205
Tspyl1	4676.7	4075.0	4678.1	4393.7	4560.8	3969.1	0.05	0.9474
Elk3	4675.6	4372.0	4556.2	4840.6	5206.7	5232.7	0.19	0.4000
Hs6st1	4670.3	4709.1	5067.0	4358.7	4730.3	4414.7	-0.07	0.9096
Dnm1l	4669.2	4788.3	4496.7	4950.0	4460.2	4853.1	0.00	1.0000
Tor1b	4667.1	4775.6	4335.5	4148.5	4471.5	4249.0	-0.13	0.6955
Snx2	4664.9	4331.9	4838.3	4538.1	4788.8	3943.7	0.01	1.0000
Fam117a	4664.9	4209.8	4266.0	3926.6	4558.8	3620.4	-0.07	0.9457
Pkn2	4660.7	4358.3	4257.9	4833.2	4785.7	4774.3	0.12	0.7524

Xpo7	4660.7	4520.5	4298.2	4764.2	4467.4	4956.4	0.06	0.9511
Atad1	4660.7	5042.5	5028.7	4734.5	5055.7	4576.0	-0.06	0.9400
Stxbp2	4655.4	4454.9	4628.7	3980.8	4218.9	4061.5	-0.12	0.7534
Lifr	4651.1	3850.9	3784.4	5257.8	5108.1	5716.3	0.40	0.0148
Myef2	4650.0	4782.0	4449.4	4767.4	4484.9	4873.1	-0.01	0.9988
Smc1a	4646.8	5025.2	4345.6	5576.3	5334.0	6189.1	0.19	0.5014
Smim7	4646.8	4726.4	4578.3	4661.2	4787.8	3998.1	-0.06	0.9515
Mapk15	4645.8	4115.1	4719.4	3529.6	3624.4	2827.8	-0.30	0.1732
Tnpo3	4644.7	4345.6	4084.6	4456.4	4496.2	4604.0	0.05	0.9592
Rnf4	4642.6	4595.2	4565.2	4668.7	4366.8	4726.3	0.00	1.0000
Stip1	4634.0	5222.0	4718.4	4994.6	4837.0	4421.1	-0.11	0.7910
Eln	4633.0	4363.8	6867.5	26038.5	10207.0	25806.4	0.70	0.0035
Smim10l1	4631.9	4280.0	4597.5	4792.9	5022.9	4526.1	0.13	0.6901
Hivep1	4631.9	4536.0	4121.9	4447.9	4013.6	4917.4	-0.01	0.9991
Cmpk1	4631.9	4366.5	4559.2	4183.5	4758.0	3650.3	-0.06	0.9521
Wars	4628.7	4858.5	4254.9	4646.4	4404.8	4703.7	-0.06	0.9126
Kdm3b	4627.7	4213.5	4371.8	4387.4	4215.9	4549.7	0.04	0.9515
Ppp1r2	4626.6	4555.1	4150.1	4564.6	4816.5	4248.1	0.00	1.0000
Aftph	4626.6	4547.8	4378.8	4189.9	4278.5	3997.2	-0.12	0.7702
Slc36a1	4622.3	4665.4	4861.4	3889.5	3925.3	4077.8	-0.22	0.3016
Pnpla8	4618.1	4294.6	4046.3	4410.7	4420.2	3978.2	-0.01	1.0000
Fbxw8	4614.9	4460.4	4477.6	4499.9	4495.1	4326.9	-0.01	1.0000
Esyt2	4613.8	4173.4	4173.3	4812.0	4462.3	4992.6	0.16	0.6375
Ccdc50	4607.4	4374.7	4433.2	4270.6	3916.0	4970.0	0.00	1.0000
Arhgef7	4602.1	4610.7	4646.8	5170.8	4938.7	5736.3	0.18	0.5644
Tmem167b	4599.9	4284.6	3906.3	3882.1	4221.0	3892.1	-0.09	0.8598
Usp15	4598.9	4293.7	4357.7	4813.0	4660.4	4429.2	0.09	0.8495
Paip2b	4598.9	4441.2	4237.8	4180.4	4769.3	4499.0	0.01	0.9986
Lonp1	4597.8	4390.2	4615.6	4580.6	4383.2	4411.1	0.01	1.0000
Cpsf7	4595.7	4567.0	4484.6	5092.2	4790.8	5086.8	0.12	0.7619
Setd7	4593.6	3881.9	4277.1	4422.4	4471.5	4718.2	0.18	0.4658
Ndufs1	4592.5	4537.8	4433.2	4546.6	4479.7	4414.7	-0.02	0.9893
Rps10-ps2	4592.5	4775.6	5064.0	4600.7	5116.3	4051.5	-0.05	0.9654
Phrf1	4591.4	4426.7	4203.5	4756.8	4489.0	4912.9	0.09	0.8548
Zdhhc8	4591.4	4197.1	3783.4	4375.7	4371.9	4268.9	0.04	0.9542
Idh3g	4589.3	4687.2	4364.7	4369.3	4896.6	4308.8	-0.04	0.9594
Cdc73	4587.2	4028.6	4125.9	4515.8	4382.2	4756.2	0.15	0.6935
Rabgap1	4587.2	4315.5	4485.6	4546.6	4386.3	4322.4	0.02	1.0000
Pura	4586.1	3978.4	3983.9	4728.1	4631.7	4987.2	0.23	0.3174
Med12	4585.0	4558.8	3944.6	4801.4	4201.5	5152.9	0.06	0.9557
Add3	4581.8	4584.3	4501.7	5081.6	4757.0	4836.8	0.09	0.8720
Eloa	4578.6	4896.8	4574.3	5062.5	5004.4	4684.7	0.02	0.9895
Tns1	4577.6	4631.7	5441.8	7001.9	6363.8	10419.1	0.42	0.2285
Coro1c	4576.5	4078.7	4448.3	4800.3	4775.4	5070.5	0.22	0.2904
Dpp4	4576.5	3753.4	3800.5	3554.0	3624.4	3406.6	-0.10	0.8314
Fam193b	4575.4	4534.2	4333.5	4510.5	4207.6	4611.3	-0.02	1.0000
Slc22a17	4575.4	4519.6	4590.4	3875.7	4327.8	3494.5	-0.19	0.5103
Gtf3c4	4573.3	4213.5	3774.3	4377.8	4322.6	4696.4	0.08	0.9280
Kdm4a	4572.2	4980.6	4492.7	4601.8	4725.1	4774.3	-0.06	0.9343
Arl5a	4570.1	4083.2	4198.5	4379.9	4542.4	4440.1	0.10	0.7896
Tbrg1	4570.1	4711.8	4260.9	4850.2	4514.6	4561.5	-0.01	1.0000
Ggcx	4568.0	4256.3	3594.9	4287.6	3610.1	4162.0	-0.06	0.9343
Tmc4	4568.0	4623.5	4495.7	4189.9	4201.5	4326.9	-0.11	0.7535
Fnip2	4565.8	4134.2	4213.6	4580.6	4412.0	4653.0	0.12	0.7524
Zfp292	4564.8	4694.5	4132.0	4882.0	4261.0	5085.9	0.03	0.9980
Kif3b	4563.7	4113.3	4225.7	4439.4	4386.3	4315.1	0.07	0.9096
Cdk5rap3	4563.7	4852.1	4452.4	4305.6	4497.2	4105.0	-0.15	0.6670
Selenos	4563.7	4925.0	4361.7	4079.5	4320.6	3640.3	-0.25	0.2613
Sdhd	4562.7	4782.0	5106.3	4183.5	4669.7	3494.5	-0.19	0.5855
Hmgn1	4557.3	5158.2	4892.7	4803.5	4799.1	4968.2	-0.07	0.9342
Cct4	4556.3	4340.1	4531.0	4536.0	4624.5	4637.6	0.07	0.9155
Psma7	4554.1	5263.9	4799.0	4237.7	4725.1	3617.7	-0.26	0.3138
Zmym4	4552.0	4206.2	4201.5	4815.2	4292.9	4755.3	0.12	0.8031
Cog7	4552.0	4550.6	4024.2	4017.9	4337.0	3952.8	-0.12	0.7770
Nudcd3	4550.9	4631.7	4353.6	4478.6	4498.2	4360.4	-0.05	0.9670
Gna12	4548.8	4727.3	4759.7	4330.0	4442.8	4352.2	-0.10	0.8314
Smc4	4547.7	4308.2	3450.9	4577.4	4326.7	5082.3	0.12	0.8445
Btbd7	4546.7	3866.4	4113.8	4680.3	4551.6	4934.7	0.24	0.2533
Stox2	4545.6	4391.1	3958.7	3783.3	3511.5	4662.0	-0.11	0.8316
Lmbrd2	4544.5	4370.2	4199.5	4460.6	4146.0	4551.5	0.00	1.0000
Maml3	4538.1	4496.8	4335.5	4942.5	4612.2	4755.3	0.08	0.8938
Zmpste24	4537.1	4350.1	4112.8	4402.2	4387.3	4291.6	0.00	1.0000
Itprid2	4528.6	4020.4	4258.9	4451.0	4377.1	4515.3	0.12	0.7656
Emc10	4527.5	4472.2	4680.1	4394.8	4697.4	4188.3	-0.02	1.0000
Atp5f1	4526.4	4672.6	4533.0	4412.8	4558.8	4191.0	-0.08	0.8899
Ubr1	4523.2	4346.5	3988.9	4896.9	4867.8	5280.7	0.19	0.5060

Ubfd1	4523.2	4059.5	3738.0	3822.6	3884.2	3700.1	-0.09	0.8894
Dgcr2	4521.1	4355.6	4867.5	4222.8	4636.8	4520.7	0.02	0.9896
Pfkl	4520.0	4329.2	4655.9	4447.9	4724.1	4352.2	0.04	0.9508
Aldh18a1	4520.0	4628.0	4208.5	4757.8	4750.8	4534.3	0.02	1.0000
Gmpr	4519.0	3103.9	4602.5	2606.1	3934.5	3373.1	-0.04	0.9988
Ist1	4517.9	4634.4	4727.4	4743.0	4435.6	4794.3	0.01	1.0000
Rnf213	4511.5	4811.1	3646.3	5003.0	3825.7	5409.3	0.03	1.0000
Wnk3	4510.4	4812.9	4077.6	5697.3	4760.0	5143.9	0.12	0.7903
Gigyf2	4510.4	4485.9	4441.3	4731.3	4329.8	4816.9	0.04	0.9522
Wasl	4508.3	4188.0	4258.9	4635.8	4572.1	4616.7	0.12	0.7271
Tent5a	4507.2	3901.9	3846.8	4715.4	5168.7	3282.5	0.12	0.8524
Lzts2	4504.0	4324.6	4673.0	4216.4	4490.0	4142.1	-0.02	1.0000
Per2	4503.0	3776.2	3625.2	4458.5	3994.1	4624.9	0.16	0.6618
Kdm7a	4503.0	3889.2	3658.4	4341.7	3909.9	4468.2	0.10	0.8489
mt-Co2	4500.9	4731.0	4924.9	4152.7	4919.2	4742.6	-0.03	0.9986
Rassf3	4497.7	4434.9	4422.1	4191.0	4165.5	3914.8	-0.11	0.7776
Adam9	4496.6	4042.2	3887.1	4435.1	4516.7	4164.7	0.09	0.8759
Pds5b	4496.6	4624.4	4009.1	4789.7	4503.4	5094.1	0.06	0.9417
Wiz	4495.5	4411.2	4287.1	4269.5	4153.2	4630.3	-0.02	0.9893
Snx17	4495.5	4582.5	4485.6	4103.9	4375.0	4022.5	-0.12	0.7648
Vim	4492.3	4953.2	5826.7	6759.9	7142.1	9611.2	0.53	0.0051
Cdipt	4484.9	4370.2	4385.9	4096.5	4507.5	4158.4	-0.04	0.9881
Ccdc88a	4483.8	4562.4	4197.5	4758.9	4521.8	5029.8	0.07	0.9273
Rc3h2	4479.5	3765.3	4024.2	4471.2	4125.5	5009.8	0.21	0.4404
Khdc4	4479.5	4628.0	4117.9	4851.2	4171.7	4750.8	0.00	1.0000
Gm10076	4479.5	5391.4	5063.0	4853.4	5003.4	3826.0	-0.18	0.6257
Slain2	4477.4	4136.1	4264.0	4553.0	4270.3	4285.2	0.06	0.9411
Vasp	4477.4	4185.2	4193.4	4217.5	4182.0	4027.1	-0.02	1.0000
Exoc5	4476.3	4194.4	4286.1	4481.8	4554.7	4442.8	0.08	0.8779
Tia1	4476.3	4821.1	4134.0	4821.5	3982.8	5289.7	-0.01	1.0000
Ralgds	4476.3	4068.6	4646.8	3806.7	3789.8	3595.9	-0.13	0.7227
Midn	4474.2	3954.8	3782.4	4284.4	3789.8	4430.1	0.06	0.9343
Fcho2	4474.2	3826.3	3751.1	3991.4	3775.4	4143.9	0.03	0.9991
Myo9b	4474.2	4636.2	3888.1	4392.7	3660.4	4553.3	-0.10	0.8494
Abhd16a	4474.2	4377.5	4354.6	4066.8	4430.5	3410.2	-0.13	0.8144
Dync1li2	4472.1	4054.1	4355.7	4683.5	4120.4	4951.9	0.14	0.7406
Pim2	4472.1	4034.9	3837.8	3870.4	4042.3	3211.9	-0.11	0.8548
Abi1	4471.0	4119.7	4233.7	4234.5	4017.7	3959.1	-0.03	1.0000
Ddx23	4467.8	4379.3	4308.3	4832.1	4510.5	4640.3	0.08	0.8884
Pcgf3	4466.8	4003.0	4242.8	4131.5	4120.4	4489.0	0.06	0.9117
Gpat4	4465.7	4865.8	4792.9	4609.2	4774.4	4589.6	-0.05	0.9654
Snx5	4464.6	4304.6	4451.4	3782.3	4129.6	3613.1	-0.16	0.6183
Twf1	4462.5	3976.6	4054.4	4106.0	4190.2	3647.5	-0.01	1.0000
Ergic3	4452.9	4895.8	5318.9	4275.9	4898.7	4157.5	-0.12	0.8207
Fbh1	4451.8	4251.8	4250.9	3961.7	4038.2	3746.3	-0.11	0.7943
Dnmt1	4450.8	5210.2	4350.6	6156.9	5238.5	6393.9	0.20	0.4688
Trim37	4450.8	4241.7	4277.1	4216.4	4237.4	4183.8	-0.01	1.0000
Zfp609	4449.7	4464.0	4451.4	5234.5	4331.9	5517.1	0.15	0.7366
Kat2b	4447.6	4301.9	4201.5	4325.8	4000.2	3731.8	-0.09	0.8515
Lgalsl	4447.6	3336.2	4000.0	3353.4	3634.7	2759.0	-0.09	0.9113
Rnf20	4441.2	4235.4	4243.8	4310.9	4322.6	4405.7	0.03	0.9695
Lmtk3	4440.1	4759.2	3970.8	4313.0	3632.7	3994.5	-0.21	0.4552
Rps11	4438.0	4896.8	5179.8	4784.4	4942.8	4823.2	-0.01	1.0000
Map4k2	4438.0	4532.3	4130.0	4063.6	3659.4	3974.5	-0.19	0.4740
Naa15	4436.9	3987.6	4408.0	4854.4	4802.1	5320.5	0.28	0.1227
Prpf38b	4436.9	4164.3	4010.1	4890.5	4429.4	4669.3	0.15	0.6597
Sept11	4431.6	4176.1	4222.7	4733.4	4588.6	5284.3	0.19	0.4611
Pcdh17	4430.5	3962.0	4075.6	5447.8	4941.8	6078.6	0.40	0.0144
C2cd3	4430.5	3907.4	3820.6	4075.3	3903.7	4638.5	0.08	0.8816
Mkln1	4430.5	4646.2	4367.7	4715.4	4698.4	5005.3	0.05	0.9263
Ctsa	4430.5	4149.7	4495.7	3809.9	4162.5	4066.9	-0.06	0.9268
Stat5b	4428.4	4392.1	4095.7	4458.5	4279.5	4025.3	-0.04	0.9900
Acbd5	4427.3	4231.7	4418.1	4224.9	4153.2	3983.6	-0.04	0.9694
Faf2	4427.3	4173.4	4241.8	4010.5	4176.8	3884.0	-0.05	0.9305
Pde5a	4420.9	2427.0	2144.1	3498.8	3958.1	3361.3	0.16	0.8455
Dock11	4420.9	4285.5	4438.3	4698.4	4772.4	4698.2	0.12	0.7304
Nacc1	4419.9	4691.8	4235.8	5014.7	4463.3	5019.8	0.05	0.9629
Gmfb	4416.7	4074.1	3986.9	4230.2	4267.2	4304.2	0.06	0.9500
Capn2	4415.6	4329.2	4251.9	4655.9	4637.9	4773.4	0.11	0.7961
Igfbp3	4414.5	4428.5	4997.5	5533.8	6902.9	5498.0	0.36	0.0586
Virma	4407.1	4143.3	4011.1	4544.5	4507.5	4845.0	0.14	0.6871
Ankrd50	4407.1	4716.4	4940.0	5304.5	5026.0	5399.3	0.14	0.7157
Smurf2	4407.1	4281.8	4062.5	3990.3	4115.2	3961.9	-0.08	0.8948
Gramd3	4405.0	4250.8	3306.8	4546.6	4719.0	4117.6	0.08	0.8899
Abcb9	4403.9	4135.1	3810.6	3561.5	3592.6	3252.6	-0.23	0.3445
Aplnr	4402.8	4348.3	4129.0	4917.1	5181.0	6382.1	0.30	0.1788

Efr3a	4402.8	4314.6	4154.1	4591.2	4652.2	4594.1	0.09	0.8522
Abcg1	4400.7	4147.9	4071.5	4041.3	4722.0	4166.6	0.05	0.9525
Gm14303	4399.6	5310.4	5837.8	5298.2	5839.2	5799.7	0.09	0.9126
Chmp5	4399.6	4540.5	4622.7	4184.6	4587.5	3632.2	-0.12	0.7896
Bgn	4398.6	4656.2	5595.9	7262.0	7083.6	10438.1	0.49	0.0885
Rab21	4398.6	4225.3	4503.8	4290.8	4415.0	3784.3	-0.03	1.0000
Hnrnpa3	4398.6	4992.4	4401.0	4709.0	4469.5	5148.4	-0.04	0.9649
Cyld	4397.5	4388.4	4089.7	4295.0	4379.1	4431.9	0.00	1.0000
Cpn1	4396.4	4833.0	4693.2	4416.0	4482.8	3739.0	-0.16	0.6656
Gm9385	4395.4	4855.8	4930.0	4792.9	5088.6	4703.7	0.01	1.0000
Wbp11	4394.3	4315.5	4243.8	4709.0	4300.1	4300.6	0.04	0.9603
Smarcc1	4392.2	4535.1	4309.3	4760.0	4556.7	4947.3	0.07	0.9267
Dpf2	4391.1	4321.0	4007.0	4394.8	4667.6	4321.4	0.05	0.9739
Nudt4	4389.0	4277.3	4490.7	4582.7	4400.7	4571.4	0.07	0.8937
Sec13	4387.9	4570.6	3919.4	4634.7	4568.0	3916.6	-0.04	0.9877
2510039O18R	4374.1	4336.5	4600.5	4013.7	4247.7	3189.2	-0.17	0.6672
Tsn	4373.0	4589.7	4430.2	4553.0	4436.6	4729.9	0.00	1.0000
Retreg3	4370.9	4504.1	4679.1	4179.3	4536.2	3997.2	-0.08	0.8727
Grb2	4370.9	4290.0	4632.7	4153.8	4117.3	3862.2	-0.09	0.8814
Gna13	4362.3	3767.1	4057.4	4161.2	3911.9	4080.5	0.08	0.9096
Zscan26	4362.3	3993.9	4081.6	4298.2	3925.3	4195.5	0.04	0.9877
Ank3	4361.3	3958.4	3963.7	4807.7	4034.1	4966.4	0.18	0.5813
Lrrc8d	4361.3	4017.6	4557.2	4585.9	4521.8	4628.5	0.16	0.5876
Nek7	4361.3	4004.0	4544.1	4260.0	4382.2	3983.6	0.05	0.9474
Amigo1	4361.3	4390.2	4067.5	3724.9	4102.9	3609.5	-0.18	0.5768
Eml2	4360.2	3717.0	4289.2	3160.2	3476.6	2881.3	-0.24	0.3174
Cdk13	4358.1	4264.5	4491.7	4702.6	4377.1	4680.1	0.09	0.8558
Slmap	4358.1	3920.1	3970.8	3956.4	3703.5	4074.2	-0.01	1.0000
H2-Q2	4357.0	4122.4	4708.3	3929.8	4443.8	3428.4	-0.08	0.8938
Brpf3	4352.7	4602.5	4365.7	4875.7	4290.8	4827.8	0.03	0.9804
Uri1	4351.7	4007.6	4025.2	4085.9	4022.8	3824.2	-0.02	1.0000
Gm5835	4350.6	4169.8	4719.4	4359.8	4812.4	4127.6	0.06	0.9452
Rabep1	4350.6	3962.0	4125.9	4020.1	4247.7	3992.6	0.03	0.9986
Clic1	4347.4	4439.4	4319.4	3924.5	4199.4	3621.3	-0.16	0.6433
Tnk2	4342.1	4547.8	4040.3	4388.4	4376.0	4803.3	0.01	1.0000
Nt5c2	4342.1	4311.0	3920.4	4110.3	4085.5	4070.5	-0.06	0.9348
9930021J03Ri	4338.9	3845.4	4267.0	4417.1	4323.7	4618.5	0.18	0.5210
Ube2h	4337.8	3849.1	5154.6	4568.9	4908.9	4194.6	0.17	0.6208
Ccar2	4330.4	4261.8	4071.5	4511.6	4054.7	4256.2	0.01	1.0000
Mettl9	4330.4	4615.3	4355.7	3924.5	4349.3	4027.1	-0.15	0.6372
Ppp3ca	4329.3	4160.7	5202.0	4526.4	4427.4	4366.7	0.06	0.9629
Selenok	4329.3	4483.2	4453.4	3961.7	4450.0	3458.2	-0.15	0.7185
Pigq	4328.2	4423.9	4424.2	4519.0	4685.1	4488.1	0.04	0.9486
Gm38070	4328.2	4382.0	4623.7	4844.9	4208.7	4453.7	0.03	0.9731
Spata13	4325.0	3999.4	4207.5	4390.5	4441.7	4958.2	0.17	0.5497
Gm5869	4325.0	4251.8	4130.0	4061.5	4627.6	3576.0	-0.05	0.9810
Anapc2	4320.8	4137.0	4405.0	4112.4	4381.2	4192.8	0.02	1.0000
Tssc4	4318.6	4262.7	4111.8	3415.0	3742.5	3405.7	-0.25	0.2101
Tesk1	4317.6	4085.9	3729.0	4007.3	3839.0	3553.3	-0.09	0.8558
Mdm1	4316.5	4052.2	3000.5	3658.1	3492.0	3374.9	-0.15	0.7531
Ints1	4315.4	4659.0	4052.4	4704.8	4051.6	4732.7	-0.02	1.0000
2700081O15R	4313.3	3965.7	4345.6	3891.6	4283.6	4446.4	0.06	0.9155
Tm9sf4	4313.3	4514.1	4339.5	4843.8	4651.2	4553.3	0.06	0.9486
Elf2	4308.0	4258.1	4249.9	4469.1	4507.5	4591.4	0.08	0.8526
Ankrd12	4305.9	4011.2	4296.2	4032.8	3918.1	4381.2	0.02	0.9858
Btrc	4305.9	4552.4	4305.3	4149.6	4150.1	4566.0	-0.07	0.8960
Atm	4304.8	4273.6	3791.4	4309.9	4034.1	4932.8	0.06	0.9660
Tomm70a	4304.8	4252.7	4436.3	4366.1	4319.6	3960.9	-0.02	0.9931
Gmps	4303.7	4227.2	3830.7	4485.0	4484.9	4595.9	0.10	0.8218
Tgfbrap1	4301.6	4100.5	3821.6	4020.1	3611.1	4164.7	-0.05	0.9457
Stx16	4297.3	4534.2	4158.2	4272.7	4094.7	4266.2	-0.09	0.8730
Ggnbp2	4296.3	3804.4	3923.4	4200.5	4136.8	4297.9	0.12	0.7741
Rpl37rt	4295.2	4498.6	4953.1	4493.5	5080.4	4570.5	0.06	0.9660
Lztr1	4295.2	4254.5	4042.3	3994.6	3718.9	3632.2	-0.15	0.6242
Grn	4294.1	3883.7	4306.3	4302.4	4624.5	4868.5	0.20	0.3837
Ilk	4293.1	4429.4	4356.7	4443.6	4392.5	4237.2	-0.02	0.9893
Tcf3	4289.9	4297.3	4372.8	4114.5	4068.0	4022.5	-0.07	0.9096
Jag2	4287.7	4176.1	4151.1	5299.2	4275.4	5891.1	0.26	0.3262
Camta2	4285.6	4133.3	3811.6	4125.1	4160.4	4500.8	0.04	0.9515
Tnks1bp1	4278.2	3960.2	4282.1	4407.5	4777.5	5246.2	0.24	0.2860
Foxp1	4277.1	4372.0	4480.6	4700.5	4612.2	4849.5	0.10	0.8313
Foxk2	4277.1	4183.4	4059.4	4509.4	4423.3	4289.7	0.07	0.8884
Cdv3	4276.0	3993.0	3953.6	4099.7	3637.8	3911.1	-0.04	0.9748
Srsf10	4275.0	4301.9	4364.7	5040.2	4558.8	4671.1	0.13	0.7286
Ykt6	4275.0	3971.2	4104.8	4056.1	4135.8	3952.8	0.02	0.9916
Zbtb7b	4273.9	4132.4	3635.3	3526.4	3468.4	3231.8	-0.24	0.3174

Micu2	4272.8	4355.6	4546.1	4017.9	4352.4	3459.1	-0.13	0.7873
Parp8	4271.8	4056.8	3480.1	4275.9	3999.2	3595.9	-0.02	1.0000
Eif2ak3	4268.6	4152.5	3607.0	3872.5	3651.1	3974.5	-0.09	0.8470
Trir	4268.6	4219.9	4260.9	3603.9	3891.4	3134.9	-0.23	0.4012
Kcnc3	4267.5	4161.6	3863.0	3865.1	3425.3	3927.4	-0.13	0.7539
Rab11b	4266.4	3898.3	4341.5	3992.5	4361.7	4079.6	0.06	0.9059
Spin1	4264.3	4067.7	4801.0	4472.3	4534.2	4597.7	0.12	0.7621
Kdm6a	4264.3	2895.2	4564.2	2961.7	2738.4	2807.9	-0.10	0.8029
Caskin2	4263.2	3939.3	3689.7	4230.2	3960.2	4322.4	0.07	0.9326
Cyfip1	4262.2	4194.4	4109.8	4622.0	4468.4	4725.4	0.12	0.7506
Nup153	4262.2	4436.7	3797.5	4823.6	4425.3	4685.6	0.08	0.9160
Vdac2	4261.1	4203.5	4531.0	4403.3	4475.6	4524.3	0.07	0.8740
Cacnb3	4261.1	4014.9	4094.7	3874.6	3983.8	3545.2	-0.08	0.8719
Usp16	4251.5	4032.2	3653.4	3905.4	3827.7	3696.5	-0.07	0.9057
Hmbox1	4249.4	4060.4	3972.8	4423.4	3749.7	4998.1	0.10	0.8911
Cd63	4249.4	4539.6	4825.2	4260.0	5294.0	3793.4	-0.02	1.0000
Diaph1	4248.3	3817.2	3851.9	4361.9	4145.0	4668.4	0.17	0.5886
Arid4b	4247.3	4260.0	4150.1	4338.5	4525.9	4535.2	0.07	0.9343
Tuba1b	4243.0	4772.9	4715.3	4885.2	4802.1	5638.4	0.10	0.8702
Txndc11	4239.8	4552.4	4013.1	4074.2	3506.4	3921.1	-0.21	0.4583
Cbfb	4236.6	4085.0	4470.5	4143.2	4259.0	4143.0	0.02	0.9861
Atl2	4234.5	4045.0	3837.8	3972.3	4107.0	3738.1	-0.03	0.9679
Coro1b	4232.3	4236.3	4106.8	3802.4	3937.6	3326.0	-0.18	0.5985
Park7	4230.2	4579.7	4906.8	4721.7	4651.2	3903.0	-0.04	0.9812
Sppl2a	4228.1	3754.3	3885.1	3795.0	3937.6	3706.4	0.01	1.0000
Gatad2a	4225.9	4123.3	3885.1	4160.2	4197.4	4363.1	0.04	0.9542
Pfkm	4222.7	3780.8	3982.9	3782.3	3687.1	3566.9	-0.05	0.9395
Gnaz	4222.7	4183.4	4050.4	4131.5	3959.2	3607.7	-0.09	0.8730
Ndel1	4222.7	4065.0	4041.3	3930.9	3807.2	3572.4	-0.10	0.8410
Dhdds	4221.7	4818.4	4038.3	3738.7	3925.3	4272.5	-0.22	0.3174
Rasa1	4218.5	4002.1	4351.6	4251.5	3817.5	4086.8	0.00	1.0000
Cep104	4217.4	3945.6	3486.1	3647.5	3693.2	3720.0	-0.08	0.8773
Pdgfrb	4213.2	4196.2	4647.8	5862.9	5855.6	7578.6	0.52	0.0014
Pnrc2	4213.2	3828.1	4257.9	3894.8	4048.5	3417.5	-0.03	0.9884
Usp24	4212.1	4215.3	3576.8	4577.4	4194.3	5319.6	0.16	0.7332
Igfbp5	4208.9	4477.7	4083.6	7225.9	5271.4	9937.2	0.44	0.1787
Tcf12	4208.9	4382.0	3722.9	4434.1	4128.6	4943.7	0.06	0.9574
Dnaja4	4208.9	4176.1	3940.5	4019.0	4671.7	3897.5	0.01	1.0000
Dlc1	4206.8	3624.1	3812.6	4988.2	4636.8	5828.7	0.42	0.0126
Itsn2	4206.8	3950.2	3778.3	4272.7	3693.2	4165.7	0.03	0.9778
Zfp266	4204.6	3986.6	3909.3	4676.1	4306.2	4778.9	0.18	0.4962
Pggt1b	4204.6	3972.1	3860.9	4541.3	4387.3	4681.0	0.17	0.5607
Thra	4204.6	4320.1	5078.1	4119.8	4391.4	4379.4	-0.02	0.9858
Dyrk1a	4203.6	3817.2	4399.0	4525.4	4525.9	4258.0	0.18	0.5153
Gm4202	4203.6	3854.5	3822.7	4085.9	4168.6	4257.1	0.10	0.8533
Zhx1	4203.6	4083.2	4277.1	3912.8	3845.2	3818.7	-0.08	0.8868
Cherp	4201.4	4501.4	3958.7	4599.7	4208.7	4776.1	0.03	0.9823
Atxn7l3	4195.0	4048.6	4445.3	4089.1	4109.1	4182.9	0.02	0.9912
Arhgap29	4192.9	4022.2	4074.5	4638.9	4666.6	4532.5	0.18	0.4917
Taldo1	4191.8	4741.9	4447.3	4714.3	4602.9	3783.4	-0.08	0.8816
Nop53	4190.8	3784.4	4599.5	3753.6	4339.1	3679.3	0.02	1.0000
Slc37a1	4190.8	4145.2	3987.9	3453.2	3871.9	3210.1	-0.21	0.4233
Wdr7	4184.4	4085.0	3756.2	4344.9	4583.4	4996.2	0.17	0.5723
Atf7ip	4182.3	4358.3	3815.6	4615.6	4323.7	5044.3	0.10	0.8520
Gsk3a	4180.1	4327.4	4233.7	4137.9	4286.7	4286.1	-0.02	0.9823
Zfp598	4178.0	4107.8	3638.3	3855.5	3748.7	3693.7	-0.10	0.8429
Tbc1d15	4178.0	4426.7	4165.2	4074.2	3959.2	3930.2	-0.13	0.7274
Elp2	4173.7	4133.3	3948.6	4044.5	3924.3	3883.1	-0.06	0.9196
Mtch2	4170.5	4241.7	4457.4	4198.4	4384.2	4094.1	-0.01	1.0000
Tecpr1	4170.5	4083.2	4226.7	3723.9	3776.4	3939.2	-0.10	0.8416
Dnpep	4170.5	4288.2	4222.7	3754.7	3731.2	3363.1	-0.22	0.3485
Eea1	4167.3	4018.5	3889.2	4467.0	4044.4	4738.1	0.12	0.7868
Itch	4167.3	3838.1	3792.4	3942.6	3963.3	4098.6	0.05	0.9611
Morf4l1-ps1	4167.3	3835.4	3967.7	3763.2	4160.4	3416.6	-0.03	0.9731
Rps4x	4166.3	4378.4	5100.2	4628.3	4932.5	4502.6	0.08	0.9005
Plxna2	4166.3	4455.8	3737.0	4597.5	4048.5	5086.8	0.06	0.9605
Cul9	4166.3	4283.6	4009.1	4019.0	3733.3	3769.8	-0.14	0.7147
Man1b1	4165.2	4536.9	4263.0	3924.5	4136.8	3894.8	-0.16	0.5589
N4bp1	4162.0	4029.5	3952.6	3855.5	3854.4	4152.1	-0.03	0.9778
Ctso	4160.9	3914.7	3966.7	3422.4	3640.9	3098.7	-0.20	0.5100
Ddx41	4156.7	4055.0	4111.8	3758.9	3968.4	3197.4	-0.14	0.7492
Ptprg	4152.4	4045.0	4000.0	5150.6	4773.4	5705.5	0.33	0.0626
Ctr9	4152.4	4050.4	4171.3	4187.8	4107.0	4244.5	0.04	0.9850
Slc30a7	4152.4	3827.2	3155.7	3818.4	3739.4	4228.1	0.04	0.9881
Rb1cc1	4152.4	4118.7	3999.0	4334.3	3980.7	4175.6	0.02	1.0000
Taok3	4148.2	4339.2	4272.0	4282.3	3974.6	3947.4	-0.08	0.8599

Mpp2	4146.0	3822.7	3829.7	3468.1	3380.1	3764.4	-0.11	0.7888
Ndufv1	4142.8	4413.0	4264.0	4413.9	4647.1	4153.9	0.01	1.0000
Vil1	4139.6	3370.8	3477.1	3567.8	3711.7	3283.4	0.03	0.9981
Man2b2	4139.6	4006.7	4073.5	3735.6	3807.2	3586.0	-0.11	0.7795
Cntrob	4136.4	4032.2	3651.4	3506.3	3817.5	3782.5	-0.10	0.8455
Neurl4	4136.4	4139.7	3833.7	3835.3	3789.8	3644.8	-0.12	0.7623
Ptpn4	4131.1	4096.9	3672.5	4199.5	3751.8	4470.9	0.02	1.0000
Ankrd24	4130.0	4044.0	3753.1	3770.6	3404.7	3749.0	-0.13	0.7506
Seh1l	4129.0	3994.8	3776.3	3899.0	3988.9	3785.2	-0.03	0.9662
Nipal1	4129.0	4008.5	3542.6	3738.7	3956.1	3311.5	-0.10	0.8370
Pbx2	4129.0	3988.5	4151.1	3630.5	3634.7	3359.5	-0.16	0.5573
Lrfn4	4129.0	3959.3	4176.3	3371.5	3687.1	3218.2	-0.20	0.4648
Gabpb2	4127.9	4191.6	3675.6	4352.3	3887.3	4941.9	0.08	0.9379
Tbc1d20	4127.9	3958.4	4070.5	3785.5	3914.0	3803.3	-0.05	0.9679
Gm6767	4127.9	4318.3	4117.9	4066.8	3282.5	3849.5	-0.18	0.6220
Cntfr	4125.8	4157.9	3939.5	3490.3	4344.2	4311.5	-0.03	0.9841
Ube2l3	4124.7	4065.9	4066.5	4020.1	3971.5	4037.0	-0.02	0.9850
Arrdc3	4124.7	4852.1	5169.8	4560.4	4491.0	4415.6	-0.09	0.9007
Nmt1	4123.6	4214.4	4317.4	4340.6	4359.6	4345.0	0.04	0.9507
Unc45a	4119.4	4106.9	3756.2	3876.7	4677.9	4008.0	0.03	0.9695
Zfyve27	4119.4	4011.2	3931.5	3598.6	3559.8	3343.2	-0.18	0.5220
Ppp2r3a	4117.3	3366.2	3032.7	4152.7	3953.0	4046.1	0.21	0.4909
Rragc	4117.3	3914.7	4110.8	3991.4	4128.6	3801.5	0.01	1.0000
Ksr2	4116.2	4260.9	3453.9	4762.1	4160.4	5352.2	0.17	0.7091
Ptpn23	4116.2	4109.6	3904.3	4499.9	4177.9	4466.4	0.09	0.8666
Rpl22	4116.2	4101.4	5009.6	3726.0	4289.8	4019.8	-0.05	0.9670
Zfp687	4115.1	4494.1	4000.0	4164.4	3795.9	4029.8	-0.14	0.6695
Rflnb	4111.9	4167.0	4956.2	4607.1	5199.5	5269.8	0.22	0.4415
Tmem178b	4110.9	3714.2	3549.6	3600.7	3614.2	3606.8	-0.04	0.9516
Rnf217	4110.9	3582.2	3961.7	3702.7	3448.9	3277.1	-0.06	0.9168
Prdx1	4110.9	4576.1	4208.5	4193.1	4262.1	3557.9	-0.15	0.6938
Apbb1	4109.8	3886.4	3979.8	4200.5	4397.6	3938.3	0.09	0.8440
Ptch1	4108.7	4021.3	4276.1	3999.9	3829.8	4236.3	-0.01	1.0000
Zer1	4108.7	4446.7	4249.9	3839.6	3620.3	3898.4	-0.20	0.4016
Osbpl2	4106.6	4083.2	3933.5	4012.6	3815.4	3850.4	-0.06	0.9368
Nsf	4105.5	4207.1	4138.0	4750.4	4579.3	4556.0	0.13	0.6819
Lims1	4100.2	4151.5	4361.7	4711.1	4557.8	5180.1	0.19	0.4653
Man1c1	4100.2	4224.4	4441.3	3649.6	3694.3	3457.3	-0.21	0.3378
Gripap1	4097.0	4059.5	3758.2	3912.8	3534.1	3941.9	-0.08	0.8730
Etv1	4094.9	4514.1	4037.3	5571.0	4911.0	4787.0	0.18	0.5855
Stau1	4094.9	3752.5	4247.8	4138.9	3983.8	4211.8	0.10	0.8113
Gga1	4094.9	4068.6	4205.5	3840.7	3966.4	3767.1	-0.07	0.8772
Zfp523	4090.6	4275.4	4172.3	3801.4	3570.0	3300.6	-0.23	0.2989
Gpbp1	4089.6	4005.8	3977.8	3741.9	3849.3	3556.1	-0.10	0.7985
Lsr	4089.6	4366.5	4134.0	3660.2	3801.0	3623.1	-0.21	0.3225
Acap2	4088.5	4191.6	3968.8	4353.4	4194.3	4420.2	0.05	0.9397
Kdm4b	4087.4	4171.6	4409.1	4077.4	3762.0	4114.9	-0.06	0.9476
Endod1	4086.4	3714.2	3875.0	4149.6	4412.0	4248.1	0.17	0.5459
Mtor	4085.3	3862.7	3516.4	4078.4	3890.4	4356.8	0.08	0.9126
Gm12966	4085.3	5135.4	5075.0	4014.7	4120.4	4785.2	-0.19	0.5985
R3hdm4	4084.2	4222.6	4420.1	3985.0	4254.9	4176.5	-0.03	1.0000
Actr10	4082.1	3854.5	3874.0	3791.8	3967.4	3556.1	-0.03	0.9654
Hnrnpll	4078.9	3940.2	3682.6	4056.1	3877.0	4023.4	0.02	1.0000
Sec24b	4074.6	4147.0	4005.0	4373.6	4279.5	4641.2	0.09	0.8520
Aebp2	4074.6	3671.4	3825.7	3865.1	3776.4	4098.6	0.07	0.9157
Gclm	4071.4	3954.8	4575.3	4016.9	4038.2	3800.6	-0.02	1.0000
Plpbp	4068.2	4178.9	3912.3	3905.4	3761.0	3577.8	-0.14	0.6839
Rprd2	4064.0	3751.6	3611.1	4336.4	4040.3	4460.0	0.17	0.5797
Lmo7	4064.0	3776.2	3922.4	4118.8	4318.5	4035.2	0.12	0.7795
Tmem181a	4060.8	3707.0	3366.2	3187.8	3124.4	3102.3	-0.22	0.4160
Cpq	4059.7	4338.3	3959.7	3837.5	4138.9	3198.3	-0.18	0.6625
Adi1	4058.7	3964.8	4102.8	3866.1	4409.9	3799.7	0.01	1.0000
Kars	4056.5	4213.5	4005.0	4469.1	4393.5	4251.7	0.06	0.9522
Map1lc3b	4056.5	4219.0	4744.6	3950.0	4418.1	3853.2	-0.05	0.9450
Spryd3	4054.4	3932.0	3800.5	3878.9	3856.5	3563.3	-0.06	0.9263
Aatk	4046.9	4202.6	4593.4	4184.6	4537.2	4058.8	0.01	1.0000
Sin3b	4044.8	4081.4	4208.5	4034.9	3877.0	3755.3	-0.07	0.8995
Marf1	4043.7	4076.8	3939.5	4099.7	3782.6	4339.6	0.00	1.0000
Gtpbp2	4043.7	3950.2	3984.9	3719.6	3575.2	3391.2	-0.14	0.7074
Raf1	4042.7	4486.8	4299.2	4486.1	4265.1	4378.5	-0.02	1.0000
Cald1	4040.5	4090.5	4594.4	5827.9	5569.1	7228.1	0.51	0.0019
Rps2	4040.5	4280.0	4757.7	4480.8	4352.4	3893.9	-0.02	1.0000
Dazap1	4040.5	4201.6	3844.8	3945.7	3804.1	3964.6	-0.09	0.8748
Ufc1	4040.5	4564.2	4309.3	4185.7	3997.2	3993.6	-0.14	0.6819
Tpst2	4036.3	4172.5	4056.4	3370.4	3951.0	3381.3	-0.20	0.5141
Chd2	4035.2	4069.5	3982.9	4267.4	4069.0	4802.4	0.10	0.8316

Enho	4033.1	4132.4	3629.2	3602.9	4235.4	2922.9	-0.15	0.7830
Xrn1	4032.0	3757.1	3669.5	4353.4	3920.2	4872.2	0.19	0.5810
Snrpb	4032.0	4286.4	3870.0	4090.1	4348.3	4202.8	-0.01	1.0000
Carhsp1	4032.0	4250.8	4189.4	3668.7	3944.8	3726.4	-0.15	0.6128
Tmed10-ps	4028.8	4540.5	3745.1	4000.9	4170.7	4023.4	-0.12	0.7336
Arhgap32	4027.8	3959.3	3471.0	4147.4	3991.0	5090.4	0.15	0.7648
Zfand5	4026.7	3907.4	4195.5	4230.2	4382.2	4213.7	0.11	0.7932
Snx3	4026.7	4203.5	4145.1	3930.9	4436.6	4007.1	-0.02	0.9884
Rpl28-ps1	4024.6	4361.1	4781.8	4362.9	4831.9	4559.7	0.06	0.9483
Sfxn2	4024.6	4269.1	3854.9	4077.4	3754.8	4044.3	-0.09	0.8498
Ntrk2	4023.5	4230.8	3863.0	4381.0	4705.6	5294.2	0.18	0.5669
Dvl1	4023.5	4078.7	3746.1	3636.8	3743.6	3394.8	-0.16	0.6400
Cant1	4021.4	3800.8	3626.2	4053.0	3756.9	3921.1	0.04	0.9896
Tubb2a	4021.4	3654.1	4481.6	4069.9	3723.0	3714.6	0.03	0.9681
Tspan8	4021.4	3369.0	3405.5	2359.8	2490.9	1973.7	-0.51	0.0016
Mapt	4019.2	3401.8	4070.5	2982.9	2731.2	3564.2	-0.16	0.6773
Phf1	4018.2	3362.6	3492.2	2850.2	3087.5	2509.9	-0.25	0.3390
Rptor	4016.0	4105.1	3570.8	4210.1	3694.3	4156.6	-0.01	1.0000
Tox4	4016.0	3983.0	3735.0	3780.1	3787.7	3976.3	-0.04	0.9841
R3hdm1	4013.9	4172.5	3867.0	4581.6	4177.9	4927.4	0.13	0.7470
Slf2	4010.7	3788.0	3452.9	4078.4	3529.0	4230.9	0.06	0.9680
Rnf167	4008.6	3981.2	4207.5	3746.2	3904.8	3460.1	-0.10	0.8514
Irf6	4007.5	3543.9	3764.2	3510.5	3756.9	3354.1	-0.02	1.0000
Ube2b	4007.5	4158.8	4539.0	3848.1	4477.7	3277.1	-0.10	0.9083
Cdc37l1	4006.4	3868.2	3503.3	3827.9	3406.8	3738.1	-0.07	0.9377
Ube4a	4004.3	3483.8	3576.8	3715.4	3531.0	4073.3	0.09	0.8705
Iars2	4003.2	4365.6	4172.3	4346.0	4174.8	4396.6	-0.01	1.0000
Tkt	4002.2	4706.4	4397.0	5082.7	4720.0	4781.6	0.06	0.9487
Gcc2	4001.1	3796.2	3638.3	3971.2	3706.6	4005.3	0.03	0.9981
Map2k1	3999.0	3796.2	4034.2	3568.9	3928.4	3248.1	-0.09	0.8652
F11r	3995.8	4228.1	4502.8	3889.5	4053.6	3891.2	-0.09	0.8643
Zfp608	3990.5	3905.6	3903.3	4090.1	4061.8	3873.1	0.03	0.9931
Shroom3	3989.4	3787.1	3497.2	3836.4	4129.6	4316.9	0.10	0.8207
Wdr11	3988.3	4014.0	3891.2	4133.6	4075.2	4203.7	0.04	0.9767
Mdm2	3987.3	3727.0	3609.1	3613.5	3446.8	3307.9	-0.10	0.8455
Insr	3985.1	3573.0	4042.3	4201.6	4150.1	4547.0	0.22	0.3039
Ythdc1	3983.0	3917.4	4316.4	4387.4	4023.9	4162.9	0.08	0.8732
Srsf4	3978.7	4009.4	3961.7	4247.2	4092.6	4488.1	0.09	0.8779
Map3k11	3977.7	3963.9	3892.2	3857.6	3794.9	3991.7	-0.03	0.9744
Cops2	3976.6	3814.5	3900.2	3864.0	3816.5	3720.0	-0.01	0.9988
Rbm26	3973.4	4045.9	3651.4	4676.1	3774.4	4669.3	0.11	0.8518
Mogs	3973.4	3995.8	3434.7	3461.7	3386.2	3200.1	-0.21	0.4232
Stam2	3969.1	3876.4	4291.2	4092.2	4179.9	3884.0	0.05	0.9452
Acvr1b	3969.1	3667.8	4029.2	3606.1	3823.6	3677.4	0.00	1.0000
Pa2g4	3968.1	4208.0	4097.7	4196.3	4225.1	3966.4	-0.02	0.9877
Reep3	3967.0	3510.2	3781.3	4277.0	3954.0	4782.5	0.25	0.2836
Srp54c	3967.0	4071.4	3776.3	3715.4	3688.1	3732.7	-0.11	0.7503
Pwwp3a	3964.9	3905.6	4011.1	3831.1	3747.7	4014.4	-0.02	1.0000
Gm21887	3964.9	1864.9	3696.7	397.0	556.5	1126.8	-0.61	0.0108
Lypla2	3962.8	3882.8	3884.1	3640.0	3722.0	3521.6	-0.09	0.8237
Anapc4	3959.6	3902.8	3701.7	3657.0	3970.5	3818.7	-0.03	0.9805
Cux1	3958.5	4020.4	3972.8	4510.5	4370.9	5100.4	0.20	0.4655
Erlec1	3957.4	4183.4	4033.2	3843.8	4164.5	3737.2	-0.08	0.9071
Hspd1	3956.4	4034.9	4160.2	4014.7	4298.0	4229.1	0.05	0.9486
Khdrbs3	3956.4	3942.0	4077.6	3994.6	4384.2	3811.5	0.04	0.9911
Wnk4	3956.4	3592.2	3490.2	3245.1	3272.3	3353.2	-0.12	0.7806
Rbm33	3955.3	4613.4	3688.7	4762.1	3828.8	5149.3	0.04	0.9991
Ice1	3954.2	3814.5	3296.7	4149.6	3678.9	4592.3	0.12	0.8429
Auts2	3954.2	3643.2	3772.3	3895.9	3580.3	4470.0	0.10	0.8665
Adipor2	3954.2	4123.3	3987.9	4186.7	4099.8	3955.5	-0.01	1.0000
Smg5	3948.9	3947.5	3460.9	3960.6	3778.5	3816.9	-0.02	1.0000
Tns2	3946.8	3688.7	3740.0	3974.4	3907.8	4182.9	0.11	0.8175
Uqcrfs1	3945.7	4087.8	4049.4	3837.5	4138.9	3755.3	-0.05	0.9326
Hmgcs1	3944.6	4500.5	4399.0	5068.9	4340.1	4720.0	0.07	0.9314
Prom1	3944.6	4303.7	3834.7	4652.7	3577.2	4401.2	0.00	1.0000
Cavin1	3943.6	3779.8	4312.3	5430.8	5875.1	6560.5	0.57	0.0000
Kpna3	3943.6	3892.8	4040.3	3903.3	3911.9	4055.1	0.02	0.9881
Tgfbr2	3942.5	3870.9	4054.4	4902.2	4825.8	5066.9	0.32	0.0332
Rcor1	3940.4	3743.4	3573.8	3978.7	3759.0	3992.6	0.06	0.9553
Tfg	3939.3	3712.4	3828.7	3824.7	3742.5	3835.9	0.02	0.9818
Hspa4l	3939.3	3200.4	3041.8	3147.5	3176.8	3253.5	-0.02	0.9881
Rpl29	3934.0	4163.4	4468.5	4481.8	4609.1	4280.7	0.09	0.8791
Aqr	3934.0	4180.7	3913.3	4453.2	4375.0	4201.0	0.06	0.9126
Casc3	3930.8	3547.5	3656.4	3840.7	3574.1	3682.0	0.04	0.9796
Pcmtd2	3930.8	3627.7	4434.2	3546.6	3930.4	3294.3	-0.04	0.9788
Soat1	3928.7	3278.8	3636.3	3193.1	3495.1	3267.1	-0.01	1.0000

Mff	3927.6	3908.3	3963.7	3578.5	3925.3	3389.4	-0.10	0.8529
Man2c1	3927.6	4065.9	4033.2	3901.2	3692.2	3581.4	-0.11	0.7997
Sec61b	3925.5	4283.6	3729.0	3645.3	3919.1	3155.7	-0.21	0.4627
Ncapd2	3924.4	4388.4	3253.4	4146.4	3853.4	5188.3	0.06	0.9748
Scaf8	3922.3	3932.0	3875.0	4421.3	4073.1	4112.2	0.09	0.8640
Gm8399	3921.2	3712.4	3939.5	4021.1	4157.3	4331.4	0.14	0.6731
Dock9	3919.1	3935.6	3529.5	4406.5	4084.4	5013.5	0.18	0.6042
Stag1	3918.0	3980.3	3669.5	4285.4	3898.6	4409.3	0.08	0.9064
Mapk6	3916.9	3884.6	3930.5	4013.7	4012.6	3917.5	0.03	0.9884
Vps11	3915.9	3734.3	3488.1	3955.3	3909.9	3692.8	0.04	0.9842
Drap1	3915.9	3964.8	4293.2	3816.2	4439.7	3121.3	-0.06	0.9559
Strn3	3912.7	3430.0	3811.6	3976.5	3660.4	3465.5	0.08	0.8795
Gprin1	3912.7	3728.8	3913.3	3806.7	3731.2	3383.1	-0.04	0.9850
Nedd4l	3911.6	3919.2	3827.7	3957.4	3915.0	4001.7	0.02	0.9932
Pacs2	3909.5	3673.3	3774.3	3600.7	3681.9	4125.8	0.04	0.9896
Rexo1	3909.5	3725.2	3440.8	3894.8	3378.0	3893.0	0.00	1.0000
Ubap2	3907.3	3965.7	3689.7	4492.4	3830.8	4759.8	0.13	0.7585
Necap1	3907.3	3664.1	3617.1	3623.0	3798.0	3152.1	-0.06	0.9372
Ppp1r13b	3905.2	3715.2	3921.4	4409.6	4024.9	4545.2	0.19	0.4917
Syndig1l	3902.0	3717.0	3502.3	3586.9	3863.7	3445.6	-0.03	0.9739
Sart1	3901.0	4022.2	3986.9	4029.6	3902.7	4002.6	-0.01	1.0000
Fnip1	3901.0	3597.6	3417.6	3562.5	3368.8	3715.5	-0.02	1.0000
Acadsb	3898.8	3637.7	3551.6	3654.9	4028.0	3690.1	0.05	0.9471
Cacul1	3898.8	3631.3	3811.6	3836.4	3696.3	3643.9	0.02	1.0000
Limk2	3896.7	3991.2	4239.8	3694.2	3506.4	3637.6	-0.13	0.7321
Pomgnt2	3895.6	3662.3	3591.9	3110.3	3448.9	2743.6	-0.22	0.4537
Pcf11	3893.5	3820.8	3488.1	3839.6	3754.8	3878.5	0.01	1.0000
Rps24-ps3	3893.5	4425.8	4483.6	4082.7	4259.0	3449.2	-0.14	0.7503
Yipf2	3892.4	3668.7	3476.1	2993.5	3559.8	2585.1	-0.23	0.4601
Ypel2	3891.4	3631.3	3884.1	3481.9	3544.4	3359.5	-0.07	0.9126
Gm6170	3889.2	4085.0	4170.3	3809.9	3978.7	3136.7	-0.14	0.7374
Psd	3889.2	3696.9	3860.9	3320.5	3365.7	3226.4	-0.16	0.5768
Tet2	3888.2	3701.5	3263.5	3765.3	3035.1	4477.2	0.03	1.0000
Ltn1	3887.1	3987.6	3506.3	4142.1	3909.9	4464.6	0.07	0.9321
Pcmtd1	3887.1	3440.0	3269.5	3549.8	3669.6	3778.9	0.07	0.9334
Gm13910	3886.0	3343.5	3880.1	3573.1	3647.0	3311.5	0.04	0.9607
B4galt5	3885.0	3440.0	3955.7	3287.6	3254.8	2981.8	-0.13	0.7237
Fbxo38	3882.8	4009.4	3988.9	4064.6	3779.5	4087.8	-0.01	1.0000
Prpf6	3881.8	3693.3	3777.3	3753.6	4158.4	4133.0	0.10	0.8376
Kcnmb2	3881.8	3492.0	3783.4	3423.5	3868.8	2998.1	-0.04	0.9877
Iffo1	3880.7	3660.5	3501.2	3585.9	3361.6	3513.5	-0.07	0.9096
Phf21a	3880.7	4083.2	4065.5	3668.7	3403.7	3759.9	-0.16	0.6042
Slc25a36	3878.6	3825.4	3798.5	4068.9	3842.1	4010.8	0.05	0.9649
Tbcd	3876.4	4208.9	4215.6	3823.7	4056.7	4016.2	-0.07	0.9237
Syngap1	3874.3	4031.3	3735.0	3468.1	3100.8	3669.3	-0.20	0.4740
Tmem63b	3873.2	3958.4	3798.5	3765.3	3631.6	3622.2	-0.09	0.8429
Vps26b	3872.2	3947.5	3919.4	3816.2	4001.3	3699.2	-0.03	0.9850
Gm4617	3872.2	4358.3	4296.2	4045.5	4169.7	4418.4	-0.03	0.9948
Fermt2	3871.1	3668.7	4022.2	3915.0	4064.9	4067.8	0.11	0.8036
Washc2	3871.1	3925.6	4019.1	3821.5	3873.9	4106.8	0.00	1.0000
Extl3	3869.0	3882.8	4194.4	4522.2	4274.4	4692.8	0.19	0.5102
Dnajb14	3869.0	3344.4	3301.8	3816.2	3503.3	4009.9	0.14	0.7470
Mapk1ip1l	3866.9	3535.7	3694.7	3886.3	3661.4	3924.7	0.09	0.8316
Sod1	3865.8	4168.9	4225.7	3680.4	3975.6	3483.6	-0.15	0.7065
Npm1	3863.7	3727.0	3874.0	3756.8	3907.8	3637.6	0.01	1.0000
Lrrc47	3861.5	3798.1	3941.5	3836.4	3744.6	3617.7	-0.03	1.0000
Gpr107	3860.5	3874.6	3688.7	3696.3	3790.8	3809.7	-0.03	0.9662
Sbk1	3860.5	3993.9	3946.6	3286.5	3291.8	3479.1	-0.23	0.2595
Vps13c	3859.4	3761.6	3188.9	4491.4	3866.8	5175.6	0.24	0.4552
Selenom	3859.4	3810.8	3896.2	3553.0	4041.3	3158.4	-0.08	0.9151
Nbeal1	3854.1	3456.4	3094.2	3925.6	3204.5	4458.2	0.13	0.7960
Tmem176a	3853.0	3850.9	3690.7	3583.8	3809.3	3555.2	-0.07	0.9244
Hdac7	3851.9	3646.8	3771.3	3930.9	3828.8	4341.4	0.12	0.7436
Ino80d	3850.9	3288.8	3282.6	3892.7	3239.4	4512.6	0.18	0.6967
Arnt	3850.9	3541.2	3300.7	4049.8	3731.2	4179.2	0.15	0.7157
Emc7	3850.9	3930.2	3760.2	3711.1	4062.9	3340.5	-0.07	0.9114
Colgalt1	3848.7	4222.6	3946.6	3858.7	3920.2	4114.0	-0.07	0.8874
Mbd3	3847.7	4113.3	3963.7	3990.3	4035.2	3698.3	-0.06	0.9515
Ubp1	3846.6	3963.9	3744.1	4343.8	3637.8	4434.7	0.06	0.9515
Atg2b	3846.6	3807.2	3259.4	4072.1	3552.6	4118.6	0.05	0.9778
Vps39	3844.5	3839.1	3913.3	3779.1	3700.4	3659.3	-0.05	0.9467
Slc37a3	3842.3	3726.1	3847.8	3734.5	3794.9	3661.1	0.00	1.0000
Ppme1	3842.3	3563.9	3687.6	3454.3	3649.1	3153.0	-0.06	0.9382
Cpsf1	3840.2	4188.0	4172.3	3942.6	3887.3	3932.9	-0.08	0.8650
Cops7a	3839.1	4125.1	4002.0	3844.9	4085.5	3326.9	-0.11	0.8031
Ddi2	3838.1	3220.5	3329.0	3882.1	3440.7	4377.6	0.21	0.5204

Trpm7	3838.1	3631.3	3475.0	4010.5	3408.8	4115.8	0.07	0.9407
Bap1	3836.0	3943.8	3715.9	3806.7	3764.1	3491.8	-0.08	0.9007
Klhl24	3834.9	4237.2	4168.2	3939.4	4337.0	3825.1	-0.05	0.9348
Fkbp2	3834.9	4178.0	3846.8	3539.2	4112.2	3463.7	-0.14	0.7539
Lonp2	3833.8	3627.7	3821.6	3782.3	3937.6	3529.8	0.03	0.9654
Timm29	3829.6	3413.6	3411.6	3288.7	3485.8	2969.1	-0.08	0.8912
Agpat1	3827.4	3934.7	3722.9	3739.8	3667.6	3342.3	-0.12	0.7896
Tbc1d23	3825.3	3484.7	3484.1	3488.2	3399.6	3440.1	-0.02	1.0000
Spcs1	3825.3	4129.7	3744.1	4078.4	4240.5	3584.1	-0.04	0.9881
Pak4	3825.3	3768.0	3746.1	3432.0	3513.6	3320.6	-0.13	0.7056
Ccdc6	3824.2	3627.7	3568.8	3750.4	3723.0	4133.9	0.08	0.8960
Eml1	3824.2	3841.8	3733.0	3765.3	4216.9	4203.7	0.08	0.9186
Grk5	3824.2	3540.2	3624.2	3824.7	3516.6	3856.8	0.06	0.9208
Rab3a	3824.2	3605.8	3862.0	3323.7	3503.3	3194.7	-0.11	0.8056
Ripor1	3823.2	3425.5	3611.1	4050.8	3846.2	4305.1	0.21	0.3625
Hnrnpa1	3822.1	4130.6	3903.3	4099.7	3731.2	4702.8	0.03	1.0000
Cnot3	3818.9	3816.3	3489.2	3791.8	3535.1	3720.9	-0.04	0.9557
Kbtbd2	3817.8	3428.2	3924.4	3900.1	4083.4	3990.8	0.18	0.4550
Itga1	3816.8	3440.0	3362.2	4833.2	4116.3	5695.5	0.42	0.0286
Cyth2	3815.7	3558.5	3771.3	3490.3	3877.0	3185.6	-0.03	1.0000
Pan3	3813.6	3625.0	3674.5	4253.6	3925.3	4309.7	0.18	0.5097
Zfp395	3813.6	3679.6	3909.3	4082.7	3864.7	4498.1	0.15	0.6681
Naa50	3813.6	3636.8	3634.2	3934.1	4125.5	3941.9	0.12	0.7648
Psma1	3812.5	4012.2	3833.7	3477.6	4031.0	3326.9	-0.13	0.7983
AI480526	3810.4	3876.4	3693.7	1653.9	1728.0	1948.3	-1.00	0.0000
Atp13a2	3809.3	3606.7	3929.5	3489.3	3145.0	3308.8	-0.12	0.7422
Mtmr2	3807.2	3390.8	3550.6	3797.1	3936.6	3499.9	0.11	0.8010
Lcorl	3807.2	3398.1	3179.8	3584.8	3370.8	3576.9	0.04	0.9729
Kansl3	3807.2	3789.9	3491.2	3910.7	3690.2	3738.1	0.00	1.0000
Psmb7	3807.2	4361.1	4175.3	3854.5	4328.8	3885.8	-0.09	0.8515
Mgat2	3805.1	3514.7	3035.8	3072.1	3038.2	2883.1	-0.20	0.5557
Sypl	3802.9	4046.8	3871.0	4238.7	4644.0	4614.9	0.15	0.6220
Phb2	3802.9	3856.4	3983.9	3987.1	4269.3	3584.1	0.03	0.9986
Mat2a	3802.9	3941.1	3784.4	3859.8	3569.0	3905.7	-0.05	0.9679
Foxj3	3801.9	3379.9	3378.3	3925.6	3652.2	4222.7	0.18	0.5417
Kpna6	3801.9	3264.2	3331.0	3688.9	3332.8	3597.7	0.09	0.8868
Tbc1d14	3801.9	3697.9	3724.9	3244.1	3428.3	3351.4	-0.14	0.6967
Hivep2	3799.7	3297.9	2737.5	3383.1	3321.6	3781.6	0.07	0.9269
Snx30	3797.6	3736.1	3419.6	3751.5	3473.5	3741.7	-0.02	0.9848
Lrp8	3796.5	4751.9	3734.0	3672.9	3088.5	3911.1	-0.32	0.1200
Tra2b	3794.4	3692.4	3676.6	3925.6	3599.8	3785.2	0.03	1.0000
Slc9a3r2	3794.4	3665.1	3804.5	3528.6	3859.6	3600.4	-0.01	1.0000
Cdk11b	3793.3	4187.1	3951.6	4425.6	4063.9	4542.5	0.06	0.9160
Bmi1	3793.3	3625.9	3799.5	3557.2	3548.5	3396.6	-0.05	0.9559
Xpr1	3791.2	3200.4	3555.7	3774.8	3442.7	3968.2	0.17	0.5985
Pik3ca	3790.1	3611.3	3635.3	3626.2	3474.5	3668.4	-0.01	0.9956
Psmc1	3789.1	4016.7	3825.7	3876.7	3822.6	3653.9	-0.07	0.9229
Fbxl3	3788.0	3751.6	3907.3	3640.0	3563.9	3091.4	-0.12	0.8031
Nans	3786.9	4128.8	3561.7	3716.5	3990.0	3624.9	-0.10	0.8237
Rubcn	3786.9	3999.4	3692.7	3474.4	3379.1	3665.7	-0.16	0.5614
Kpna4	3785.9	3336.2	3397.5	4177.2	4173.8	4258.0	0.29	0.1035
Ints6l	3785.9	3998.5	3364.2	4170.8	3493.0	4297.0	0.02	0.9877
Ulk1	3783.7	3938.4	3662.5	3723.9	3444.8	3888.5	-0.08	0.8772
Ddx21	3781.6	3265.1	3036.8	3982.9	3485.8	4184.7	0.21	0.5557
Nudt3	3781.6	3949.3	4209.6	3781.2	4245.6	3318.8	-0.06	0.9382
Rgp1	3780.5	3893.7	3703.8	3901.2	3777.4	4299.7	0.04	0.9868
Sbf2	3780.5	4067.7	3727.9	4155.9	3796.9	4367.6	0.03	1.0000
Xpot	3780.5	4010.3	3804.5	3734.5	3430.4	3938.3	-0.09	0.8366
Rims2	3779.5	3689.7	3368.2	3996.7	3633.7	3907.5	0.06	0.9521
Ablim1	3777.3	3439.1	3725.9	3582.7	3619.3	4173.8	0.11	0.8370
Crybg3	3776.3	3716.1	3216.1	4374.6	3778.5	4718.2	0.20	0.5872
Maea	3776.3	3596.7	3918.4	3391.6	3869.8	3565.1	-0.01	1.0000
Tmem163	3776.3	3980.3	3643.3	3520.1	3531.0	3246.3	-0.18	0.4786
Fam160b1	3772.0	3348.9	3346.1	3839.6	3508.4	3851.3	0.13	0.7719
Irf2bp1	3772.0	3525.7	3602.0	3406.5	3757.9	3391.2	-0.01	1.0000
Abcf3	3771.0	3617.7	3266.5	3081.7	3254.8	2879.5	-0.21	0.4783
Pcmt1	3769.9	3781.7	3713.8	3423.5	3953.0	3587.8	-0.04	0.9526
Snx27	3767.8	3424.5	3599.0	3531.7	3299.0	3772.5	0.03	0.9980
Fam168a	3767.8	3989.4	3981.8	3875.7	3885.2	4344.1	0.02	1.0000
Fkbp1b	3765.6	3291.5	4034.2	3153.8	3844.2	2821.5	-0.05	0.9532
Chmp1a	3765.6	3696.9	3898.2	3597.6	3687.1	3105.9	-0.09	0.8548
Aldh3a2	3764.6	3821.7	3929.5	3565.7	3855.5	3556.1	-0.06	0.9521
Psmd13	3763.5	4025.8	3647.3	3571.0	3541.3	3286.1	-0.18	0.4988
Trim33	3762.4	3582.2	3477.1	4427.7	3850.3	4380.3	0.21	0.4466
Xpo5	3762.4	3717.9	3499.2	4071.0	3811.3	4320.5	0.12	0.7809
Prkar2b	3762.4	4045.9	4044.3	3580.6	3804.1	3199.2	-0.17	0.6226

Rgl2	3762.4	3962.0	4014.1	3473.4	3337.0	3132.2	-0.23	0.2820
Tmem250-ps	3758.2	3875.5	3730.0	3612.4	4025.9	3514.4	-0.05	0.9495
Ppp6c	3755.0	3469.2	3705.8	3665.5	3663.5	3669.3	0.06	0.9334
Snx1	3753.9	3492.9	3822.7	3383.1	3683.0	3254.4	-0.03	0.9944
C2cd2l	3753.9	3530.2	3277.6	3011.6	3159.3	3008.1	-0.19	0.5463
Togaram1	3752.8	3253.3	3319.9	3764.2	3437.6	3653.0	0.12	0.7571
Ubxn7	3752.8	3660.5	3644.3	3630.5	3868.8	3889.4	0.05	0.9713
Fam160a2	3750.7	3620.4	3744.1	3444.7	3262.0	3432.0	-0.10	0.8068
Stk38	3749.6	3585.8	3415.6	3716.5	3376.0	3892.1	0.03	1.0000
Sap130	3748.6	3143.0	2958.2	3338.5	3008.4	3794.3	0.07	0.9452
Atp5d	3748.6	3906.5	4077.6	3664.4	3941.7	3630.3	-0.06	0.9559
Map9	3747.5	3405.4	3418.6	3724.9	3719.9	3641.2	0.10	0.8117
Zbtb41	3747.5	3518.4	3357.2	3777.0	3459.1	3664.8	0.04	0.9557
Pmpca	3745.4	4018.5	3854.9	3904.3	3953.0	3595.0	-0.06	0.9253
Tm9sf1	3744.3	3889.2	3399.5	3929.8	3693.2	3645.7	-0.03	0.9925
Ppp5c	3744.3	3621.3	3546.6	3597.6	3753.8	3158.4	-0.04	0.9845
Creb1	3743.3	3686.0	3623.2	3796.1	3723.0	3990.8	0.05	0.9521
Dbnl	3741.1	3587.6	3678.6	3502.0	3449.9	3276.2	-0.07	0.9168
Gnptg	3741.1	4191.6	4054.4	3540.2	3829.8	3065.1	-0.22	0.4525
Slc35b4	3740.1	3748.9	3497.2	3581.6	3781.5	3854.1	0.00	1.0000
Mgat1	3740.1	3773.5	3699.7	3675.1	3895.5	3517.1	-0.03	1.0000
Zcchc2	3737.9	3713.3	3585.9	3900.1	3678.9	3911.1	0.04	0.9804
Ncaph2	3737.9	3849.1	3480.1	3761.0	3895.5	3589.6	-0.02	1.0000
Abhd13	3735.8	3302.5	3282.6	3394.8	3412.9	3153.0	0.00	1.0000
6430548M08R	3733.7	3578.5	3384.4	3412.9	3504.3	3891.2	0.01	1.0000
Pcif1	3732.6	3768.0	3676.6	3780.1	4000.2	3986.3	0.06	0.9521
Scamp5	3729.4	3496.5	3774.3	3475.5	3504.3	3550.6	-0.01	1.0000
Col1a1	3727.3	3983.9	5795.4	9419.1	10716.3	25017.5	0.45	0.1204
Azi2	3726.2	3562.1	3349.1	3374.6	3395.5	3348.6	-0.07	0.9029
Pde8a	3725.1	3456.4	3278.6	3795.0	3861.6	3868.6	0.14	0.7420
Hsph1	3725.1	3275.1	3343.1	3419.2	3277.4	3259.9	0.00	1.0000
Stx6	3725.1	3943.8	4196.5	3924.5	4020.8	3717.3	-0.02	1.0000
Usf2	3725.1	3819.9	3963.7	3439.4	3862.7	3426.5	-0.09	0.8817
Cops3	3725.1	4066.8	3470.0	3697.3	3614.2	3288.9	-0.16	0.6422
Pip5k1b	3721.9	3182.2	3153.6	3298.2	3363.7	2893.0	-0.02	0.9931
Akirin1	3721.9	3717.9	3725.9	3588.0	3578.2	2958.3	-0.12	0.7713
Ahi1	3720.9	3589.4	3484.1	3464.9	3823.6	3576.9	0.01	0.9981
Casz1	3719.8	3682.4	3720.9	3803.5	3741.5	3909.3	0.05	0.9690
Plekhb2	3718.7	3477.4	3487.1	3382.1	3694.3	3506.2	0.01	1.0000
Hprt	3718.7	3697.9	3570.8	3576.3	3753.8	3367.7	-0.05	0.9515
Hipk2	3716.6	3191.3	2635.8	3545.5	2870.8	4326.9	0.10	0.9126
Zfp24	3716.6	3531.1	3657.4	3496.7	3690.2	3343.2	-0.02	1.0000
Cnot10	3715.5	3821.7	3361.2	3633.7	3356.5	3730.0	-0.07	0.9201
Strbp	3712.3	3646.8	3589.9	3744.1	3675.8	3786.1	0.03	0.9942
St6gal1	3712.3	3595.8	4097.7	3385.3	3382.1	3378.5	-0.10	0.8591
Mcfd2	3712.3	3810.8	3200.0	3198.4	3368.8	3178.4	-0.19	0.5012
Stx12	3711.3	3574.9	3987.9	3649.6	3882.2	3464.6	0.02	1.0000
Prkar2a	3711.3	3314.3	3424.7	3375.7	3104.9	3561.5	0.00	1.0000
Cog3	3710.2	3518.4	3256.4	3325.8	3072.1	3209.2	-0.12	0.7713
Mfsd14b	3709.2	3636.8	3580.8	3601.8	3583.4	3167.5	-0.07	0.9382
Gm8355	3708.1	3690.6	3722.9	4204.8	4079.3	4018.9	0.14	0.6881
Ythdf1	3708.1	3533.0	3388.4	3546.6	3373.9	3618.6	-0.01	1.0000
Cfap20	3707.0	3757.1	3642.3	3401.2	3472.5	3216.4	-0.14	0.6827
1500011B03R	3704.9	4071.4	4014.1	3329.0	4002.3	3065.1	-0.19	0.6185
Ccnt1	3703.8	3587.6	3379.3	3963.8	3829.8	4111.3	0.13	0.7406
Pik3r2	3703.8	3649.6	3734.0	3139.0	3433.5	3092.3	-0.17	0.5601
Crebzf	3700.6	3625.0	3502.3	3732.4	3598.8	3191.9	-0.04	0.9804
Sfswap	3698.5	3982.1	3966.7	4045.5	3584.4	4039.7	-0.02	0.9857
Slc35a4	3694.2	3669.6	3295.7	3373.6	3470.4	3379.4	-0.09	0.8816
Sh3glb2	3694.2	3475.6	3394.4	3107.1	3133.7	2744.5	-0.20	0.4811
Myo5a	3693.2	3543.9	3293.7	4082.7	3583.4	4814.2	0.20	0.5745
Rhpn2	3693.2	3045.6	3201.0	3470.2	3727.1	3175.6	0.14	0.7506
Flot2	3693.2	3316.1	3884.1	3522.2	3373.9	3551.5	0.04	0.9515
Dlk1	3693.2	4895.8	4671.0	3874.6	3983.8	4610.4	-0.17	0.6955
Serinc5	3691.0	3815.4	4177.3	3756.8	3658.3	3870.4	-0.02	1.0000
Nemf	3688.9	3321.6	3019.6	3196.3	2984.8	3461.9	-0.04	0.9810
Slc25a1	3688.9	3683.3	3904.3	3390.6	3822.6	3221.8	-0.08	0.9100
Unc5b	3686.8	3560.3	3472.0	3215.4	3516.6	3921.1	0.00	1.0000
Sumf1	3685.7	3584.9	3831.7	3322.6	3878.1	3273.5	-0.04	0.9838
Usp20	3685.7	3225.0	3077.1	2979.7	3162.4	3173.8	-0.06	0.9392
Axin1	3684.6	3936.5	4064.5	3517.9	3881.1	3559.7	-0.09	0.8340
Fnbp4	3683.6	3419.1	3131.5	3541.3	3049.5	3817.8	0.02	0.9896
Cse1l	3682.5	3804.4	3531.5	4011.6	3866.8	3998.1	0.06	0.9126
Tmem39a	3680.4	3305.2	3008.6	3379.9	3182.9	3322.4	-0.01	1.0000
Ppip5k2	3676.1	3552.1	3406.5	3753.6	3467.4	3985.4	0.07	0.9108
Cnpy2	3676.1	3783.5	3852.9	3277.0	3753.8	2859.5	-0.17	0.6695

Atp6v1d	3667.6	3361.7	3510.3	3296.1	3671.7	3055.2	-0.02	0.9884
Adar	3665.5	3443.7	3374.3	3727.1	3381.1	3626.7	0.05	0.9781
Lrrc41	3663.3	3816.3	3786.4	3847.0	4025.9	3525.3	0.00	1.0000
Egln2	3662.3	3589.4	3566.7	3383.1	3778.5	3098.7	-0.06	0.9476
Cmas	3662.3	3574.9	3617.1	3783.3	3461.2	2906.6	-0.07	0.9126
Sfr1	3662.3	3740.7	3878.1	3506.3	3639.8	3424.7	-0.08	0.8990
Senp5	3658.0	3524.8	3344.1	3628.3	3433.5	3592.3	0.01	1.0000
Gcnt2	3656.9	3268.8	3073.0	3399.1	3285.6	3027.1	-0.02	0.9967
Psmc3	3656.9	3993.0	3878.1	4003.1	4055.7	3637.6	-0.02	1.0000
Lama5	3655.9	4311.0	4201.5	5985.0	4805.2	8036.9	0.37	0.3174
Cbx7	3654.8	3242.3	3447.8	2812.0	2987.9	2354.1	-0.24	0.3625
Ybx3	3653.7	3881.0	4052.4	3994.6	3851.4	3806.1	0.00	1.0000
Selenow	3653.7	3387.2	3791.4	3031.8	3838.0	3172.9	-0.04	0.9744
Eps8l2	3653.7	3793.5	3507.3	3279.1	3496.1	3304.3	-0.15	0.6172
Upf2	3652.7	3513.8	3608.0	3841.7	3732.3	3980.0	0.12	0.7830
Agl	3650.5	3656.9	3466.0	4054.0	3900.6	4235.4	0.14	0.6819
Rheb	3650.5	3621.3	3550.6	3664.4	3863.7	3404.8	0.01	1.0000
Fam222a	3649.5	3711.5	3308.8	3353.4	3346.2	3003.5	-0.17	0.6211
Nelfb	3647.4	3246.0	3618.1	3234.5	3429.4	3093.2	-0.02	0.9886
Fam8a1	3647.4	3081.1	3522.4	3202.7	3073.1	3040.7	-0.02	0.9942
Clasp2	3644.2	3346.2	3896.2	4186.7	4024.9	4380.3	0.28	0.1349
Ptpn9	3644.2	3555.7	3647.3	3692.0	3652.2	3616.8	0.03	0.9931
Micos10	3642.0	3625.0	3674.5	3583.8	3731.2	3040.7	-0.06	0.9244
Morc2a	3639.9	3806.3	3450.9	3947.9	3749.7	4206.4	0.07	0.9382
Pxk	3638.8	3296.1	2966.2	3272.7	3189.1	3163.9	-0.03	0.9967
Osbpl5	3638.8	3758.9	4021.1	3522.2	3402.7	3884.9	-0.06	0.9326
Reep6	3632.4	3195.9	3457.9	3194.2	3386.2	3108.6	0.00	1.0000
Nipsnap2	3632.4	3798.1	3837.8	3603.9	3765.1	3627.6	-0.04	0.9744
Btbd1	3631.4	3200.4	3523.4	3160.2	3373.9	2826.9	-0.06	0.9343
Sdhc	3630.3	3750.7	3777.3	3537.1	3860.6	3495.4	-0.04	0.9559
Kpna1	3629.2	3294.3	2937.0	3469.1	3203.5	3508.1	0.04	0.9925
Pan2	3629.2	4018.5	3275.6	4055.1	3051.5	4005.3	-0.06	0.9676
Mest	3628.2	4229.0	4817.1	4597.5	4086.5	5518.9	0.14	0.8259
Zc3h18	3628.2	3461.9	3288.7	3717.5	3481.7	3783.4	0.07	0.8907
Rpl18-ps2	3628.2	3914.7	3980.8	3492.5	4107.0	2855.0	-0.13	0.7896
Gpbp1l1	3627.1	3488.3	3777.3	3519.0	3804.1	3196.5	-0.01	1.0000
Wdr33	3625.0	3774.4	3536.5	4071.0	3916.0	4191.9	0.11	0.7831
Golga5	3623.9	3683.3	3515.4	3802.4	3493.0	3220.0	-0.06	0.9515
Fbxl5	3623.9	3677.8	3493.2	3389.5	3384.2	3122.2	-0.14	0.6819
Gtpbp1	3622.8	3483.8	3229.2	3679.3	3779.5	3715.5	0.09	0.8404
Gmppb	3622.8	3376.3	2631.7	2949.0	2772.2	2661.2	-0.21	0.5557
Ddx19b	3621.8	3100.2	2987.4	3499.9	3467.4	3224.6	0.10	0.8314
Dcaf6	3621.8	3587.6	3721.9	3532.8	3403.7	3254.4	-0.08	0.8742
Phyh	3620.7	3645.0	3682.6	3734.5	3865.7	3471.8	0.02	1.0000
Washc5	3620.7	3572.1	3288.7	3652.8	3480.7	3628.5	0.01	1.0000
Dip2b	3619.6	3347.1	2878.6	4077.4	3811.3	4230.9	0.25	0.3501
Bag1	3617.5	3655.0	4169.3	3818.4	3969.4	3660.2	0.04	0.9742
Coa5	3617.5	3590.3	3479.1	3589.1	3552.6	3443.7	-0.02	0.9841
Pex19	3617.5	3668.7	3917.4	3174.0	3828.8	3171.1	-0.11	0.8314
Cdc23	3615.4	3851.8	3551.6	3743.0	3851.4	3368.6	-0.06	0.9522
Fkbp5	3614.3	3844.5	4120.9	3957.4	4019.7	3884.9	0.04	0.9662
Dynlt3	3614.3	3061.0	3389.4	3089.1	3304.1	2962.8	0.00	1.0000
Nfatc2	3612.2	3025.5	3058.9	3068.9	2758.9	3158.4	-0.03	0.9731
Psmb6	3612.2	4051.3	3975.8	3599.7	3953.0	2943.8	-0.17	0.6812
Fubp3	3609.0	4006.7	3570.8	3485.0	3438.6	3591.4	-0.16	0.5561
Psma2	3607.9	3874.6	3909.3	3763.2	3909.9	3314.2	-0.07	0.9474
Pomgnt1	3607.9	3423.6	3439.8	3199.5	3344.1	2941.0	-0.11	0.7910
Plekha5	3606.9	3562.1	3763.2	3555.1	3472.5	3603.2	-0.01	1.0000
Ap3s2	3606.9	3786.2	3989.9	3662.3	3499.2	3576.9	-0.07	0.8809
Zfc3h1	3606.9	3595.8	3229.2	3341.7	2902.6	3371.3	-0.14	0.7405
Inpp5a	3605.8	3562.1	3282.6	3512.6	3579.3	3470.0	-0.01	1.0000
Mysm1	3604.7	3505.6	3422.7	3854.5	3199.4	3720.0	0.03	1.0000
Ube3b	3604.7	3917.4	3590.9	3352.3	3219.9	3544.3	-0.18	0.4786
Chmp3	3603.7	3327.1	3480.1	3342.8	3478.6	2919.3	-0.04	0.9593
Nrbp1	3601.5	3550.3	3817.6	3519.0	3711.7	3248.1	-0.03	0.9944
Map4k3	3601.5	3914.7	3932.5	3459.6	3327.7	3843.2	-0.12	0.7682
Hsbp1	3600.5	3525.7	3795.5	3404.4	3700.4	3052.5	-0.06	0.9244
Gm10221	3599.4	4188.9	4117.9	3525.4	4021.8	3015.3	-0.20	0.6123
Ap1m1	3598.3	3846.3	4029.2	3327.9	3621.4	3444.7	-0.14	0.7406
Eif4a1	3597.3	3802.6	3800.5	3873.6	3831.9	3838.7	0.02	1.0000
Fto	3596.2	3424.5	3773.3	3869.3	3829.8	4186.5	0.18	0.5014
Afg3l2	3596.2	3258.7	3391.4	3710.1	3586.5	3557.9	0.13	0.7544
Med22	3596.2	3686.0	3605.0	3314.1	3650.1	3308.8	-0.09	0.8375
Atxn1l	3593.0	3498.3	3315.9	3818.4	3662.4	3907.5	0.11	0.8183
Cox8a	3593.0	3753.4	3896.2	3657.0	4265.1	3406.6	0.01	1.0000
Tmem131l	3589.8	3492.0	3519.4	4233.4	3941.7	4133.0	0.21	0.3574

Trappc8	3589.8	3417.3	3261.4	3299.3	3340.0	3323.3	-0.04	0.9604
Rwdd4a	3588.7	3493.8	2908.8	3089.1	3251.7	3009.0	-0.13	0.7608
Zfp236	3584.5	3943.8	3622.2	3448.9	3500.2	3931.1	-0.09	0.8775
Rps19-ps6	3582.4	3761.6	4146.1	4047.7	4098.8	3955.5	0.09	0.8845
Ptpru	3581.3	3291.5	3166.7	3101.8	3111.1	3218.2	-0.06	0.9202
Ppip5k1	3580.2	3850.9	3549.6	3513.7	3342.1	3546.1	-0.13	0.7509
Ndn	3580.2	3924.7	3990.9	3455.3	3499.2	3054.3	-0.20	0.4800
Ttc14	3579.2	3746.1	3182.9	3760.0	2984.8	4083.2	-0.02	0.9946
Tspyl2	3579.2	3376.3	3026.7	3131.5	3070.0	3355.0	-0.07	0.9404
Rab12	3578.1	3352.6	3472.0	3527.5	3807.2	3246.3	0.06	0.9610
1110004F10Ri	3578.1	3351.7	3546.6	3441.5	3461.2	3009.9	-0.03	1.0000
Ildr1	3576.0	3411.8	3465.0	3234.5	3258.9	2918.4	-0.12	0.7797
Ormdl3	3574.9	3623.1	3466.0	3266.4	3761.0	3179.3	-0.08	0.9208
Rnf216	3573.8	3621.3	3531.5	3484.0	3470.4	3480.9	-0.05	0.9616
Vkorc1l1	3571.7	3123.0	3301.8	3202.7	3271.2	3415.7	0.05	0.9343
Psmc5	3570.6	3940.2	3923.4	3802.4	3992.0	3568.7	-0.04	0.9557
Lingo1	3570.6	3958.4	3838.8	3004.2	3040.2	2874.0	-0.37	0.0131
Trim8	3569.6	3326.2	3729.0	3693.1	3599.8	3157.5	0.04	0.9593
Fam32a	3569.6	4141.5	4264.0	3771.7	3998.2	3406.6	-0.12	0.8207
N4bp2	3569.6	3817.2	3260.4	3342.8	2969.4	3720.9	-0.14	0.7271
Vps41	3568.5	3575.8	3417.6	3577.4	3663.5	3614.9	0.02	0.9858
Paics	3567.4	3475.6	3709.8	3931.9	4145.0	3840.5	0.17	0.5623
Ergic2	3567.4	3672.3	3405.5	3272.7	3270.2	3151.2	-0.16	0.5965
Brf1	3565.3	3421.8	3657.4	3533.9	3240.4	3560.6	0.00	1.0000
Uhrf2	3565.3	3708.8	3316.9	3646.4	3285.6	3599.5	-0.06	0.9260
Cbx1	3564.2	3522.0	3911.3	3774.8	3770.2	3969.1	0.10	0.8029
Yipf4	3564.2	3651.4	3864.0	3265.3	3763.1	3027.1	-0.11	0.8462
Inpp5f	3563.2	3373.5	3321.9	3564.7	3601.9	3329.6	0.04	0.9555
Rorc	3562.1	2402.4	3750.1	2988.2	2700.4	2587.8	0.09	0.8809
Mrtfb	3561.0	3631.3	3467.0	4167.6	3694.3	4677.4	0.19	0.5985
Vwa8	3560.0	3751.6	3379.3	4092.2	4156.3	4344.1	0.16	0.5905
Chuk	3560.0	3563.0	3121.4	3666.6	3167.5	3528.9	-0.03	0.9834
Csk	3558.9	3386.3	3620.1	3708.0	3732.3	3541.6	0.09	0.8234
Ptk2	3557.8	4013.1	3824.7	4113.5	4176.8	4764.4	0.12	0.7648
Dach1	3556.8	3448.2	3614.1	3664.4	3947.9	3444.7	0.08	0.8730
Pamr1	3555.7	3304.3	3462.0	2961.7	3469.4	3318.8	-0.03	0.9977
Gpatch8	3554.6	3854.5	3769.3	4009.4	3763.1	4330.5	0.07	0.9297
Atp6v0a2	3554.6	3369.9	3057.9	3386.3	3284.6	3384.9	0.00	1.0000
Klf9	3553.6	3222.3	3172.8	3619.9	3926.3	3622.2	0.18	0.5273
Sft2d2	3553.6	3538.4	3664.5	3478.7	3626.5	3739.9	0.03	1.0000
Eif3g	3553.6	3428.2	3426.7	3473.4	3386.2	3187.4	-0.03	0.9651
Asxl1	3551.5	3438.2	3451.9	3833.2	3408.8	3964.6	0.10	0.8465
Knop1	3551.5	3387.2	3246.3	3247.3	3600.8	3424.7	0.01	0.9980
Dusp18	3551.5	3197.7	3402.5	2883.1	3334.9	3072.4	-0.06	0.9297
Edc4	3549.3	3428.2	3477.1	3705.8	3359.5	3585.1	0.04	0.9545
Usp12	3548.3	3352.6	3613.1	3469.1	3219.9	3219.1	-0.03	0.9896
Rif1	3547.2	3654.1	3040.8	4358.7	3660.4	4466.4	0.19	0.5646
Prr14l	3547.2	3370.8	3033.7	3842.8	3259.9	4083.2	0.13	0.7910
Hmgb1	3546.1	3717.9	3780.3	3217.5	3579.3	3017.1	-0.16	0.6217
Mapkapk2	3545.1	3238.7	3423.7	3347.0	3587.5	3289.8	0.05	0.9377
Hspd1-ps3	3545.1	3475.6	3184.9	3559.3	3702.5	3432.0	0.04	0.9573
Whrn	3542.9	3292.4	3157.7	3437.3	3241.5	3276.2	0.01	1.0000
Dek	3541.9	4300.9	3823.7	4420.3	4279.5	4289.7	0.03	0.9857
Csnk2a1	3539.7	3398.1	3597.0	3571.0	3581.3	3797.0	0.09	0.8455
Cuedc1	3539.7	3428.2	3697.7	3553.0	3479.7	3851.3	0.06	0.9114
Psmc4	3539.7	3532.0	3292.7	3590.1	3671.7	3460.1	0.02	1.0000
Sgsm2	3536.5	3713.3	3534.5	3349.2	3159.3	3243.6	-0.17	0.5571
Smchd1	3534.4	3458.3	2922.9	3730.3	3541.3	4208.2	0.14	0.7332
Patj	3533.3	3442.8	3125.4	3843.8	3598.8	3899.4	0.13	0.7414
Rps26-ps1	3533.3	4105.1	4756.7	3944.7	4117.3	4133.9	-0.01	0.9966
Ell2	3530.1	3740.7	3379.3	3696.3	3757.9	3161.1	-0.06	0.9362
Rreb1	3529.1	3342.6	3458.9	3723.9	3120.3	4055.1	0.09	0.9007
Htatsf1	3529.1	3573.0	3553.6	3649.6	3516.6	3748.1	0.03	1.0000
Impact	3529.1	3045.6	2911.8	2988.2	2894.4	2929.3	-0.06	0.9334
Gm8822	3526.9	3503.8	3473.0	3464.9	3384.2	3154.8	-0.07	0.9132
Tsc1	3524.8	3649.6	3312.8	3945.7	3296.9	4039.7	0.05	0.9486
Sptbn2	3523.7	3584.0	3680.6	3579.5	3226.1	3661.1	-0.04	0.9662
Lrfn3	3523.7	3369.9	3600.0	2831.1	3196.3	2953.7	-0.16	0.6191
Hip1	3521.6	3526.6	3471.0	4129.4	3855.5	4647.5	0.23	0.3339
Ppp1ccb	3521.6	3337.1	3311.8	3403.3	3332.8	2920.2	-0.05	0.9694
Dars	3517.4	3694.2	3603.0	3463.8	3613.2	3220.9	-0.09	0.8797
Nup88	3514.2	3807.2	3629.2	4027.5	3985.9	3731.8	0.05	0.9694
Ap3b1	3513.1	3551.2	3688.7	3997.8	3707.6	4088.7	0.13	0.7075
Tmx3	3512.0	3249.6	3245.3	3251.5	3081.3	3264.4	-0.03	0.9972
Psmd6	3512.0	3778.0	3543.6	3531.7	3774.4	3367.7	-0.07	0.9305
Snw1	3508.8	3435.5	3489.2	3410.7	3744.6	3195.6	0.00	1.0000

C1qtnf6	3508.8	3519.3	3312.8	3175.1	3435.5	3242.7	-0.09	0.8598
Adarb1	3506.7	3532.0	3444.8	3289.7	3151.1	3581.4	-0.07	0.9096
Ppp1r9a	3505.6	3446.4	3420.6	3509.5	3099.8	3922.0	0.02	0.9884
Tmem167	3505.6	3334.4	3074.0	3212.2	3076.2	3326.9	-0.05	0.9662
Eif3e	3505.6	3466.4	3617.1	3192.1	3517.7	2900.3	-0.11	0.8494
Pias2	3503.5	3531.1	3452.9	3512.6	3669.6	3476.4	0.01	0.9986
Gosr1	3503.5	3540.2	3244.3	3471.2	3457.1	3200.1	-0.05	0.9614
Elavl1	3499.2	2883.4	3283.6	3595.4	3600.8	3627.6	0.27	0.1263
Zfp36l2	3497.1	3295.2	3406.5	4623.0	4115.2	5161.1	0.42	0.0094
Fnbp1l	3496.0	3717.0	3961.7	3440.5	3132.6	4204.6	-0.04	0.9900
Rogdi	3496.0	4107.8	3759.2	3651.7	3566.9	3350.5	-0.18	0.4988
Ankrd54	3496.0	3619.5	3131.5	2981.9	3270.2	2742.7	-0.22	0.4186
Dhx36	3495.0	3266.0	3250.4	3514.8	3458.1	3581.4	0.09	0.8316
Fech	3495.0	3074.7	3303.8	3030.7	3065.9	3115.0	-0.02	0.9932
Arel1	3495.0	3484.7	3712.8	3219.7	3392.4	3376.7	-0.07	0.9074
Mfsd14a	3495.0	3367.1	3305.8	3393.7	3351.3	2819.7	-0.07	0.9096
Rcn2	3495.0	3541.2	3694.7	3223.9	3662.4	2966.4	-0.10	0.8470
Fam171a1	3493.9	3761.6	3634.2	3575.3	3678.9	4016.2	0.01	1.0000
Per1	3492.8	2961.7	2627.7	3281.2	3035.1	3357.7	0.10	0.8714
Rab8a	3492.8	3476.5	3362.2	3211.2	3328.7	2888.5	-0.13	0.7648
Ppat	3491.8	2756.8	2370.8	3159.1	3371.9	2878.5	0.14	0.8316
Fam120b	3491.8	3177.7	3300.7	3105.0	3005.3	3153.9	-0.05	0.9604
Pla2g6	3491.8	3728.8	3465.0	3150.7	3329.8	2981.8	-0.21	0.4006
Bahcc1	3490.7	3777.1	3008.6	3743.0	3125.4	3934.7	-0.02	0.9858
Bahd1	3490.7	3102.0	3669.5	3094.4	3084.4	2981.8	-0.05	0.9521
Mzt1	3489.7	3227.8	3086.1	2929.9	3266.1	2923.8	-0.08	0.8809
Cops5	3489.7	3629.5	3469.0	3240.9	3526.9	2950.1	-0.14	0.7134
Cenpf	3485.4	2893.4	1927.4	2787.6	2409.8	4077.8	0.07	0.9649
Cspp1	3484.3	3238.7	2828.2	3214.3	2815.4	3650.3	0.00	1.0000
Ambra1	3484.3	3460.1	3182.9	3369.3	3248.7	3511.7	-0.03	0.9858
Myo15b	3483.3	2841.5	2574.3	2928.8	2442.7	2644.9	-0.09	0.9108
Fbxl17	3479.0	3416.3	3302.8	4085.9	3829.8	3873.1	0.19	0.4598
Cdc27	3479.0	3038.3	3135.5	3738.7	3339.0	3420.2	0.17	0.5972
Arl8a	3477.9	2980.0	3801.5	3347.0	3160.4	3134.9	0.06	0.9232
Dhx32	3473.7	3468.3	3591.9	3649.6	3610.1	2980.0	-0.02	1.0000
Pogz	3472.6	3387.2	3406.5	3729.2	3364.7	4040.7	0.12	0.8237
Exoc4	3472.6	3757.1	3222.2	3768.5	3732.3	3793.4	0.02	1.0000
Ube2e3	3472.6	3417.3	3786.4	3442.6	3599.8	3335.1	0.01	1.0000
Hnrnph2	3470.5	3339.8	3361.2	3429.8	3557.7	3519.8	0.06	0.9487
Rora	3470.5	3113.0	3155.7	3222.8	3197.3	3266.2	0.04	0.9640
Slc4a2	3470.5	3538.4	3517.4	3558.3	3231.2	3400.3	-0.05	0.9377
Ckap4	3468.3	3296.1	3081.1	3620.9	3883.2	3602.3	0.15	0.6726
Ube2k	3468.3	3617.7	3466.0	3581.6	3672.7	3629.4	0.01	1.0000
Triobp	3468.3	3559.4	3665.5	3320.5	3344.1	3237.2	-0.10	0.8063
AU040320	3468.3	3278.8	3323.9	3037.1	3139.8	2991.8	-0.10	0.8170
Abhd15	3468.3	3628.6	3175.8	3498.8	3373.9	3100.5	-0.10	0.8314
Gm16580	3467.3	3448.2	3353.1	3617.7	3809.3	3698.3	0.10	0.8440
Evl	3467.3	3629.5	4070.5	3823.7	3847.3	3765.3	0.06	0.9323
Carm1	3465.1	3464.6	3292.7	3446.8	3431.4	3531.6	0.01	1.0000
Fbxw5	3465.1	3592.2	3684.6	3439.4	3466.3	3381.3	-0.06	0.9487
Srpk1	3464.1	3198.6	3013.6	3407.5	3184.0	3684.7	0.09	0.9007
Sos2	3464.1	3355.3	3302.8	3416.0	3051.5	3538.9	-0.01	1.0000
Rps20	3463.0	4042.2	4342.6	4040.2	4308.3	4234.5	0.05	0.9400
Rbm6	3461.9	3314.3	3385.4	3453.2	3532.0	3352.3	0.05	0.9515
2410002F23Ri	3461.9	3484.7	3430.7	3447.9	3433.5	3529.8	0.00	1.0000
Atpif1	3461.9	4220.8	4114.8	3659.1	3773.3	2894.9	-0.22	0.5110
Nfxl1	3459.8	3533.9	2950.1	3153.8	2844.1	3338.7	-0.14	0.7413
Parp6	3458.8	3143.9	3514.3	3478.7	3318.5	3111.3	0.05	0.9490
Scamp2	3458.8	3213.2	3308.8	3238.8	3181.9	3242.7	-0.01	1.0000
Zkscan3	3458.8	3456.4	3534.5	3497.8	2978.6	3271.7	-0.08	0.9096
Ankrd28	3457.7	3478.3	3322.9	3869.3	3557.7	3871.3	0.11	0.8059
Idh3b	3457.7	3375.3	3363.2	3333.2	3601.9	3052.5	-0.02	1.0000
Bub3	3457.7	3604.0	3326.9	3105.0	3458.1	3033.4	-0.14	0.7185
Lpcat3	3455.6	3387.2	3359.2	3389.5	3523.8	3092.3	-0.02	1.0000
Uhrf1bp1	3455.6	3421.8	3311.8	3116.7	2767.1	3364.9	-0.13	0.7564
Zkscan1	3454.5	3019.1	2996.5	3688.9	3759.0	3675.6	0.26	0.2346
Traf7	3454.5	3749.8	3746.1	3680.4	3423.2	3717.3	-0.04	0.9515
Sirt2	3454.5	2966.3	2956.2	2775.9	2987.9	2804.3	-0.06	0.9215
Chpf2	3453.4	3374.4	2982.4	3226.0	3266.1	3237.2	-0.04	0.9532
Cdip1	3449.2	3227.8	3090.2	3271.7	3369.8	3587.8	0.07	0.9035
Egln1	3449.2	3297.9	3413.6	3417.1	3556.7	3332.3	0.05	0.9645
Gne	3449.2	3148.5	2885.6	3043.4	2915.0	3057.9	-0.06	0.9263
Blmh	3449.2	3658.7	3597.0	3523.3	3566.9	3331.4	-0.06	0.9474
Tmem234	3448.1	3444.6	3531.5	3090.1	3241.5	2957.3	-0.14	0.6695
Lemd2	3448.1	3488.3	3512.3	3291.8	3173.7	2618.6	-0.18	0.6336
Mfge8	3446.0	3548.4	4103.8	4034.9	4662.5	4924.7	0.30	0.1906

Ric8b	3446.0	3186.8	2656.9	2739.8	3192.2	2602.3	-0.13	0.8237
Frs2	3444.9	3430.9	3261.4	3590.1	3349.3	3676.5	0.04	0.9515
Bhlha15	3444.9	3170.4	2618.6	2831.1	2242.4	2888.5	-0.20	0.6295
Jmy	3442.8	3560.3	3107.3	3337.5	3340.0	3483.6	-0.05	0.9593
Ifnar1	3441.7	3399.9	3233.2	3271.7	3113.1	3496.3	-0.04	0.9679
Usp35	3440.6	2811.4	2564.2	2771.7	2729.1	2603.2	-0.07	0.9515
Tmem184a	3439.6	3296.1	3210.1	3149.6	3153.2	2892.1	-0.10	0.8343
Ficd	3439.6	3646.8	2946.1	3218.6	3177.8	3065.1	-0.16	0.6131
Nub1	3438.5	3370.8	3395.5	3101.8	3364.7	3220.9	-0.06	0.9515
Ddx54	3436.4	3232.3	2999.5	3305.6	3347.2	3329.6	0.04	0.9823
Slc25a23	3436.4	3133.9	3417.6	3200.5	3107.0	3457.3	0.03	0.9731
Tor1aip1	3435.3	3324.3	3189.9	3296.1	3355.4	3451.0	0.02	0.9906
Sucla2	3434.2	3327.1	3486.1	3392.7	3611.1	3113.1	0.01	1.0000
Acsl5	3434.2	3277.9	3245.3	3280.2	3259.9	3261.7	-0.01	1.0000
Gm3531	3433.2	3497.4	3851.9	3585.9	3549.5	3427.4	0.00	1.0000
Gm15542	3433.2	3047.4	3572.8	3159.1	3215.8	2845.0	-0.02	1.0000
Comt	3433.2	3287.9	3484.1	3134.7	3391.4	3191.9	-0.03	0.9810
Nynrin	3432.1	3278.8	3408.6	3344.9	3172.7	3643.0	0.03	0.9932
Wrnip1	3432.1	3451.0	3674.5	3206.9	3394.5	3238.1	-0.07	0.9239
Rbms1	3427.8	3354.4	3712.8	3491.4	3634.7	3766.2	0.09	0.8316
Tspan9	3427.8	3855.5	3843.8	3703.7	4224.1	4150.3	0.07	0.9126
Tmem50a	3426.8	3406.3	3590.9	3018.0	3558.7	2997.2	-0.09	0.8960
Ahsa1	3426.8	3482.8	3217.1	3267.4	3395.5	2926.6	-0.10	0.8314
Phactr4	3425.7	3587.6	3390.4	3588.0	3384.2	3895.7	0.02	0.9863
Tex2	3425.7	3289.7	3358.2	3245.1	3073.1	3330.5	-0.03	0.9660
Tmco3	3425.7	3252.4	3141.5	3044.5	3283.6	2858.6	-0.08	0.9152
Fry	3424.7	3291.5	3052.9	4236.6	3615.2	4998.1	0.33	0.1432
Armc10	3424.7	3372.6	3276.6	3128.4	3418.1	3031.6	-0.07	0.9256
Mydgf	3424.7	3801.7	3268.5	3077.4	3299.0	2430.2	-0.29	0.2661
Usp36	3423.6	2918.9	2753.6	3237.7	2948.8	3549.7	0.12	0.8366
Rnf19a	3421.5	3330.7	3271.5	3280.2	3303.1	3110.4	-0.04	0.9592
Bicc1	3420.4	3590.3	3458.9	4189.9	3931.4	5372.1	0.29	0.2424
Iars	3418.3	3252.4	3280.6	3733.4	3391.4	3909.3	0.15	0.6681
Fmnl3	3417.2	3626.8	3731.0	4084.8	3932.5	4203.7	0.16	0.6333
Pdhb	3417.2	3381.7	3417.6	3099.7	3216.8	2991.8	-0.12	0.7896
Sae1	3416.1	3396.3	3346.1	3476.5	3654.2	3776.2	0.09	0.8375
Snap47	3415.1	3380.8	3829.7	3198.4	3527.9	2959.2	-0.07	0.9057
Stxbp5	3414.0	3215.0	2871.5	3487.2	3109.0	3569.7	0.07	0.9155
Ap3m1	3414.0	3265.1	3006.5	3455.3	3401.6	3210.1	0.04	0.9831
Galnt11	3414.0	3367.1	3201.0	3016.9	2860.5	2651.2	-0.22	0.3592
Jun	3412.9	3028.2	4678.1	22535.5	14281.1	21563.7	1.43	0.0000
Snx13	3412.9	3261.5	3399.5	3381.0	3583.4	3147.6	0.03	0.9654
H6pd	3412.9	3565.8	3341.0	3359.8	3744.6	3688.3	0.02	0.9877
Gm9531	3412.9	3640.5	3586.9	3683.5	3673.7	3664.8	0.02	1.0000
Kat7	3409.7	3328.0	2979.3	3586.9	3527.9	3740.8	0.12	0.7811
Tsr1	3409.7	3031.0	2727.4	3157.0	3243.5	3297.0	0.08	0.8899
Gon4l	3409.7	3399.0	3003.5	3408.6	3332.8	3900.3	0.07	0.9152
Clasp1	3407.6	3376.3	2999.5	3611.4	3131.6	4054.2	0.09	0.8726
CT010467.1	3406.5	3984.8	5510.3	1829.0	2273.2	2951.9	-0.44	0.1832
Ncoa5	3404.4	3477.4	3284.6	3087.0	3294.9	3120.4	-0.12	0.7538
Med13l	3403.3	3114.8	2993.4	3579.5	3445.8	4090.5	0.22	0.4679
Sh3bp4	3403.3	3234.1	3325.9	3711.1	3655.3	3382.2	0.13	0.7600
Acadvl	3403.3	3376.3	3593.9	3520.1	3622.4	3067.9	0.00	1.0000
Synj1	3402.3	3196.8	3157.7	3520.1	3054.6	3701.0	0.08	0.8779
Fgfr1op2	3402.3	3512.0	3645.3	3572.1	3582.4	3186.5	-0.03	0.9810
Cers2	3402.3	3635.0	3454.9	3373.6	3398.6	3451.9	-0.08	0.8762
Tmcc3	3402.3	3608.6	3676.6	3361.9	3293.8	3374.0	-0.10	0.8213
Ran	3400.1	3803.5	3475.0	3766.3	3873.9	3666.6	0.00	1.0000
Ppp1r12c	3399.1	3410.9	3430.7	3457.4	3148.0	3538.0	-0.01	1.0000
Chchd2	3399.1	3662.3	3928.4	3096.5	3701.5	2471.9	-0.20	0.6270
Dpysl2	3398.0	2683.9	4148.1	3774.8	4221.0	3912.9	0.42	0.0111
Cc2d2a	3398.0	2747.7	2614.6	2967.0	3156.2	2992.7	0.10	0.8705
Sgta	3398.0	3556.6	3602.0	3092.3	3328.7	3153.9	-0.14	0.6819
Cobll1	3395.9	3574.9	3442.8	3895.9	3939.7	4150.3	0.15	0.6183
Fhdc1	3394.8	2782.3	3021.7	2912.9	3149.1	2973.7	0.08	0.8890
Ext2	3394.8	3601.3	3353.1	3625.2	3654.2	3693.7	0.03	0.9884
Wwp2	3394.8	3492.9	3222.2	3370.4	3514.6	3322.4	-0.03	0.9810
Szt2	3393.8	3798.1	3012.6	3461.7	2919.1	3786.1	-0.10	0.8473
Mdc1	3392.7	3911.0	3405.5	4376.7	3671.7	4792.4	0.15	0.7208
Rictor	3389.5	3276.0	2860.4	3635.8	2986.8	3717.3	0.08	0.9100
Tpp2	3389.5	3309.8	3170.8	3419.2	3288.7	3708.2	0.06	0.9379
Chmp7	3389.5	3265.1	3232.2	2959.6	3291.8	2630.4	-0.13	0.8011
Sos1	3387.4	3147.6	3200.0	3637.9	3409.9	3790.7	0.17	0.5723
Exoc7	3385.2	3522.9	3686.6	3202.7	3419.1	3147.6	-0.10	0.8415
Plk2	3384.2	3178.6	3638.3	5437.2	5021.9	5593.1	0.66	0.0000
Slc29a4	3384.2	3373.5	3279.6	2501.0	2820.5	2662.1	-0.31	0.0785

Mob1a	3383.1	2981.8	3135.5	3322.6	3377.0	3724.5	0.18	0.5574
Cbfa2t2	3383.1	3329.8	3489.2	3585.9	3339.0	3249.0	0.02	0.9896
Nfatc3	3382.0	3136.7	3079.1	3549.8	3353.4	3794.3	0.16	0.6155
Rnf6	3382.0	3353.5	3180.8	3392.7	3526.9	3396.6	0.04	0.9824
Ttc39a	3379.9	3653.2	3586.9	3344.9	3113.1	3173.8	-0.16	0.6060
Rprd1b	3378.8	3362.6	3505.3	3718.6	3965.3	3588.7	0.14	0.7069
Guk1	3378.8	3770.7	3677.6	3384.2	3811.3	3035.2	-0.11	0.8455
Cdc16	3377.8	3338.0	3281.6	3349.2	3369.8	3191.0	-0.01	0.9934
Cc2d1b	3377.8	3535.7	3414.6	3404.4	3219.9	3432.9	-0.06	0.9397
Rnf181	3377.8	3429.1	3325.9	2926.7	3076.2	2848.7	-0.19	0.4316
Foxk1	3376.7	3422.7	3248.4	3548.7	3156.2	3882.1	0.05	0.9737
Celf4	3376.7	3277.0	3696.7	3263.2	3347.2	3171.1	-0.02	0.9884
Klc2	3374.6	3235.0	3338.0	3158.1	2990.9	2838.7	-0.11	0.7888
Cxxc1	3372.4	3406.3	3368.2	3428.8	3391.4	3314.2	-0.01	1.0000
Ctdnep1	3372.4	3523.8	3391.4	3357.7	3667.6	3210.1	-0.03	0.9679
Ncln	3372.4	3518.4	3210.1	3129.4	3294.9	2918.4	-0.15	0.6983
Brox	3371.4	3197.7	3175.8	3161.3	3370.8	2951.0	-0.02	0.9932
Gnpat	3371.4	3285.2	3026.7	2762.1	2906.7	2810.6	-0.19	0.4991
S100pbp	3370.3	2982.7	2989.4	2964.9	2749.7	2980.0	-0.05	0.9515
Qars	3369.2	3324.3	3486.1	2992.5	3164.5	3028.0	-0.11	0.7619
Secisbp2l	3367.1	3014.6	3247.3	3215.4	3033.0	3435.6	0.07	0.8948
Dennd6a	3367.1	3335.3	3369.3	3465.9	3526.9	3350.5	0.04	0.9513
Egfl7	3367.1	3502.0	3766.2	3669.7	3739.4	3018.0	-0.01	0.9999
Shank3	3365.0	3238.7	3299.7	3978.7	3814.4	4846.8	0.33	0.1196
Suclg1	3365.0	3688.7	3878.1	3428.8	3423.2	3140.3	-0.13	0.7535
Ube2g2	3363.9	3100.2	3097.2	2953.2	3111.1	2748.1	-0.08	0.9108
Fh1	3362.9	3763.4	3083.1	3429.8	3861.6	3207.3	-0.06	0.9317
Scp2	3361.8	3410.0	3468.0	3322.6	3509.5	2847.8	-0.07	0.9157
Yipf5	3361.8	3395.4	3050.9	3275.9	3136.7	2874.9	-0.11	0.8316
Bcl2l13	3359.7	3342.6	3179.8	3522.2	3469.4	3722.7	0.09	0.8714
Mcur1	3358.6	3658.7	3589.9	3665.5	3400.6	3160.2	-0.08	0.8719
Gm9625	3357.5	3604.0	3743.1	3659.1	3887.3	3595.0	0.04	0.9850
Gm5905	3357.5	3532.0	3781.3	3384.2	3814.4	3250.8	-0.02	1.0000
Upf3b	3355.4	3508.4	3393.4	3585.9	3724.0	3850.4	0.08	0.8494
Ginm1	3353.3	2985.4	3291.7	3001.0	3182.9	2746.3	-0.02	0.9818
Vcpip1	3351.1	2990.0	2969.3	3386.3	3287.7	3364.9	0.14	0.7405
Zc3h15	3350.1	3114.8	3219.1	3184.6	3290.8	2980.0	0.01	1.0000
Sh3gl1	3347.9	3403.6	3343.1	3209.0	3644.0	3140.3	-0.02	1.0000
Drosha	3345.8	3483.8	3255.4	3206.9	3388.3	3614.0	-0.02	1.0000
Sf3a1	3344.7	3332.5	3116.4	3786.5	3575.2	3818.7	0.15	0.6595
Cgn	3341.5	3672.3	3341.0	3548.7	3220.9	3601.4	-0.06	0.9400
Zfand6	3340.5	3288.8	3487.1	3026.5	3134.7	2935.6	-0.11	0.7896
Psmd8	3338.3	3575.8	3683.6	3435.1	3638.8	3042.5	-0.07	0.9392
Zfp260	3337.3	2990.0	2958.2	3544.5	3800.0	3651.2	0.26	0.2104
Pigk	3335.1	3280.6	3535.5	3460.6	3467.4	3297.9	0.04	0.9522
Gm10093	3335.1	3476.5	3319.9	3280.2	3345.2	3133.1	-0.08	0.8960
Ndufb11	3335.1	3640.5	3729.0	3002.0	3424.2	2983.6	-0.19	0.5409
Igfbp4	3330.9	3784.4	5013.6	5071.0	5457.2	7481.7	0.34	0.3921
Med24	3330.9	3646.8	3465.0	3417.1	3359.5	3303.4	-0.10	0.8054
Aagab	3329.8	3494.7	3891.2	3462.7	3527.9	3164.8	-0.05	0.9695
Map4k5	3328.8	3241.4	3336.0	3268.5	3090.5	3473.6	0.01	1.0000
Arpp19	3328.8	3448.2	3504.3	3332.2	3548.5	3423.8	0.00	1.0000
Syap1	3328.8	3220.5	3160.7	3238.8	3007.4	2773.5	-0.09	0.8545
Susd6	3327.7	3047.4	3383.4	3469.1	3320.5	3221.8	0.10	0.8237
AW549877	3327.7	3357.1	3286.6	3519.0	3431.4	3653.0	0.07	0.9148
Zdhhc17	3326.6	3055.6	2993.4	3097.6	2970.4	2969.1	-0.02	1.0000
Cav1	3325.6	3344.4	3501.2	4804.5	4785.7	5412.0	0.52	0.0001
Mcrip1	3323.4	3511.1	3272.5	3390.6	3278.4	2704.6	-0.13	0.7535
Hexim1	3322.4	2957.2	3152.6	3588.0	3688.1	3632.2	0.26	0.1531
Sobp	3322.4	3307.0	3318.9	3321.6	2936.5	3277.1	-0.05	0.9399
Cul5	3319.2	3102.0	3140.5	3547.7	3400.6	3338.7	0.13	0.7539
Stard13	3319.2	3015.5	2868.5	3186.7	3069.0	3489.0	0.09	0.8591
Pnliprp2	3318.1	6096.6	5798.5	8593.2	4846.3	7836.8	0.20	0.7910
Ubxn10	3318.1	3322.5	3550.6	3040.3	3524.9	2715.5	-0.10	0.8641
Slc48a1	3317.0	2834.2	3310.8	2985.1	3257.9	2978.2	0.08	0.9114
Zfp827	3317.0	3216.8	3133.5	3225.0	3293.8	3576.9	0.06	0.9269
Psen1	3316.0	3138.5	3119.4	3200.5	3084.4	3374.0	0.03	0.9810
Smpd3	3316.0	3358.0	3603.0	3078.5	3117.2	2965.5	-0.13	0.7441
Hacd3	3314.9	3144.9	3457.9	3295.0	3345.2	3181.1	0.04	0.9830
Tra2a	3312.8	3134.8	3166.7	3215.4	2638.8	3378.5	-0.03	0.9839
Zfp282	3311.7	3138.5	3295.7	3639.0	3530.0	3141.2	0.11	0.8028
Tex261	3311.7	3511.1	3161.7	3091.2	3594.7	3603.2	-0.01	1.0000
Elp1	3310.6	2990.9	2949.1	3180.4	3188.1	3437.4	0.11	0.8316
Pex5l	3310.6	3431.8	2826.2	3507.3	3049.5	2930.2	-0.08	0.9005
Scn9a	3309.6	3369.9	3247.3	3865.1	3633.7	3723.6	0.14	0.6465
March4	3307.4	3318.9	3039.8	3004.2	2993.0	2809.7	-0.15	0.6256

Naa60	3304.2	3023.7	3022.7	3140.0	3434.5	3412.0	0.12	0.7682
Cnst	3304.2	3235.0	2896.7	3197.4	3157.3	3017.1	-0.04	0.9681
Pnrc1	3303.2	3143.0	4006.0	4135.8	4240.5	3876.7	0.30	0.1410
Rhbdd2	3303.2	3376.3	2964.2	3170.8	3097.7	3245.4	-0.07	0.9279
Dnajc1	3302.1	2379.6	2232.7	2440.5	2338.9	2557.0	-0.01	1.0000
Vmac	3302.1	3287.9	3340.0	2792.9	2918.0	2730.9	-0.21	0.3794
Ffar2	3301.1	3307.0	3167.7	2001.0	1829.7	1984.5	-0.69	0.0000
H19	3300.0	5089.9	6509.8	5224.9	6212.9	8408.3	0.18	0.8375
Tmem109	3300.0	3614.0	3156.7	3643.2	3618.3	3780.7	0.04	0.9704
Ndfip2	3298.9	3199.5	3214.1	3027.5	3272.3	2627.6	-0.10	0.8479
Frmd4a	3297.9	3360.8	3177.8	3495.7	3428.3	4078.7	0.12	0.8022
Abhd8	3297.9	3152.1	3040.8	3001.0	3267.1	2768.0	-0.06	0.9515
Ccnl1	3296.8	3064.7	2918.9	3264.2	3098.7	2990.9	0.02	1.0000
Supt20	3294.7	3379.9	3202.0	3315.2	3036.1	3430.2	-0.04	0.9594
Tmem87b	3294.7	3134.8	2965.2	3005.2	2923.2	2907.5	-0.08	0.8677
Tfdp1	3291.5	3690.6	3531.5	3491.4	3603.9	3205.5	-0.08	0.8762
Exoc6b	3290.4	3438.2	3084.1	3745.1	3726.1	3798.8	0.13	0.7429
Eef1d	3287.2	3443.7	3436.8	3440.5	3555.7	3241.8	-0.01	1.0000
Pex6	3287.2	3182.2	3756.2	3243.0	3274.3	2769.9	-0.06	0.9559
Ptprm	3286.1	3382.6	3342.1	4329.0	4036.2	4386.7	0.31	0.0681
Amigo2	3285.1	3425.5	3805.5	2990.4	3163.4	2953.7	-0.16	0.6116
Igsf8	3285.1	2988.2	3231.2	2531.8	2878.0	2506.3	-0.18	0.5899
Pcbp3	3284.0	2915.3	3419.6	2673.0	2661.4	3065.1	-0.08	0.9096
Msl2	3282.9	3243.2	3301.8	3844.9	3826.7	3592.3	0.19	0.4658
Arfgap2	3282.9	3041.9	3276.6	2942.6	3205.5	3057.9	0.00	1.0000
Map3k10	3279.7	2954.5	3166.7	3036.0	3043.3	2772.6	-0.02	1.0000
Appl1	3278.7	3246.0	3056.9	3319.4	3294.9	3412.0	0.04	0.9521
Zfp451	3276.5	2907.1	2957.2	3234.5	2827.7	3379.4	0.09	0.9100
Tob1	3274.4	3119.3	2707.3	3876.7	3606.0	3276.2	0.19	0.6131
Dad1	3273.3	3733.4	3372.3	3732.4	3847.3	3482.7	0.00	1.0000
Ap1s1	3270.1	3354.4	3441.8	2994.6	3314.4	2549.8	-0.16	0.6872
Spty2d1	3268.0	3056.5	2686.1	3349.2	3242.5	3493.6	0.13	0.7623
Atp6v1g1	3268.0	3396.3	3416.6	3083.8	3723.0	3051.6	-0.04	0.9925
Arpc3	3268.0	3649.6	3477.1	3405.4	3704.5	2913.9	-0.09	0.8868
Ppp6r2	3265.9	3515.6	3389.4	3546.6	3102.9	3341.4	-0.06	0.9215
Rbck1	3265.9	3714.2	3324.9	3435.1	3367.8	3257.2	-0.12	0.7506
Atxn2	3264.8	3121.2	3068.0	3372.5	3210.7	3654.8	0.11	0.8059
Acaa1a	3264.8	3171.3	4181.3	2880.0	3266.1	2712.8	-0.13	0.7896
Urgcp	3263.8	3064.7	3037.8	3186.7	3303.1	3124.9	0.05	0.9323
Ufd1	3263.8	3272.4	2888.7	2774.9	3018.7	2986.3	-0.13	0.7271
Birc2	3263.8	3158.5	2952.1	2937.3	2850.3	2801.6	-0.13	0.7470
Gm15501	3262.7	3337.1	3504.3	3321.6	3685.0	3471.8	0.06	0.9343
Psmd12	3262.7	3195.9	3451.9	3112.4	3243.5	3038.9	-0.04	0.9654
Kat6b	3260.6	3274.2	3452.9	3961.7	3407.8	4037.0	0.19	0.5561
Bccip	3259.5	3018.2	2921.9	3073.2	3230.2	2754.5	0.00	1.0000
B4galt3	3259.5	3167.6	3060.9	2925.6	3043.3	2772.6	-0.11	0.7896
Mapk9	3255.2	3040.1	3011.6	3215.4	3216.8	3392.1	0.09	0.8375
Nup50	3254.2	3127.5	2978.3	3283.3	3246.6	3384.0	0.07	0.9232
Rbpms	3254.2	3512.0	3795.5	3247.3	3498.2	3579.6	-0.03	0.9858
Mllt10	3252.0	3225.0	3055.9	3369.3	3255.8	3303.4	0.04	0.9649
Slc25a46	3249.9	3111.2	3217.1	3089.1	3094.6	2871.3	-0.05	0.9660
Nadk	3248.8	3277.9	2991.4	2928.8	3085.4	3115.9	-0.09	0.8543
Psma6	3247.8	3532.0	3577.8	3043.4	3481.7	2821.5	-0.15	0.6900
Nup205	3245.6	3614.0	2804.0	3843.8	3253.8	3998.1	0.07	0.9400
Dctn4	3245.6	3098.4	2988.4	3263.2	3296.9	3039.8	0.04	0.9848
Spg7	3245.6	3297.9	3228.2	3294.0	3195.3	3110.4	-0.04	0.9660
Rtl6	3245.6	3017.3	2936.0	2728.2	2971.4	2915.7	-0.07	0.9312
Hint1	3245.6	3536.6	3468.0	3334.3	3400.6	3140.3	-0.09	0.8613
Mbd1	3244.6	3263.3	2986.4	3374.6	3396.5	3022.6	0.01	1.0000
Myh14	3243.5	3201.3	2924.9	3605.0	3343.1	3715.5	0.14	0.6869
Gopc	3243.5	3019.1	3132.5	3066.8	3083.3	3063.3	0.01	1.0000
Zfp146	3242.4	3198.6	3040.8	3435.1	3403.7	3298.8	0.08	0.8762
Prex1	3242.4	3630.4	3166.7	3844.9	3361.6	3732.7	0.03	0.9742
Zfp646	3242.4	3190.4	3192.9	3198.4	3157.3	3470.0	0.03	0.9945
Tcta	3242.4	3582.2	3195.0	2765.3	3114.2	2478.2	-0.30	0.1580
Tmf1	3241.4	2916.2	2790.9	3253.6	3186.0	3186.5	0.12	0.8059
Sox12	3241.4	2918.0	3551.6	2959.6	2791.7	2764.4	-0.06	0.9117
Arhgap23	3240.3	3175.8	3272.5	3894.8	3503.3	3951.9	0.22	0.3174
Mier1	3240.3	2959.0	3148.6	3406.5	3465.3	3514.4	0.20	0.4251
Ranbp9	3237.1	2918.0	3323.9	3199.5	3128.5	2955.5	0.06	0.9260
Sppl3	3237.1	2952.6	3342.1	2968.1	3123.4	3138.5	0.04	0.9679
Sept8	3235.0	3439.1	3345.1	3572.1	3246.6	4402.1	0.12	0.8207
Selenon	3235.0	3266.9	3131.5	3100.8	3031.0	3221.8	-0.06	0.9232
Sh2b1	3235.0	3070.2	3140.5	2856.6	2864.6	2829.6	-0.10	0.7927
Elf1	3232.9	3299.7	3059.9	3353.4	3171.6	3232.7	-0.01	1.0000
Tmem165	3231.8	3196.8	3108.3	3113.5	2982.7	2659.4	-0.12	0.8059

Per3	3230.7	2826.9	3032.7	3439.4	3124.4	4227.2	0.27	0.3140
Tomm20	3230.7	3143.0	3459.9	3002.0	3046.4	2811.5	-0.09	0.8714
Sec11a	3230.7	3360.8	3054.9	3084.8	3229.1	2884.0	-0.11	0.8316
Ypel3	3229.7	3442.8	4406.0	3236.6	3449.9	2941.0	-0.11	0.8410
Rnf38	3228.6	2895.2	3270.5	3354.5	2968.4	3583.2	0.15	0.7310
Eif4a3	3227.5	3296.1	3045.8	3220.7	3424.2	2966.4	-0.03	0.9988
Fmr1	3225.4	3035.5	2847.3	3454.3	3285.6	3221.8	0.12	0.7821
Golt1b	3225.4	3137.6	2726.4	3343.9	3235.3	3014.4	0.04	0.9679
Brk1	3224.3	3066.5	3445.8	3002.0	3313.3	2901.2	-0.01	1.0000
Trim39	3223.3	3005.5	2575.3	3212.2	2655.2	2941.0	-0.02	1.0000
Gpr158	3222.2	3079.3	2895.7	3819.4	3629.6	4156.6	0.30	0.1197
Ace	3222.2	3316.1	3113.3	3958.5	3734.3	4746.3	0.29	0.1798
Zmym3	3221.1	3225.9	3160.7	3091.2	3032.0	3374.0	-0.02	0.9858
Myl6	3220.1	3402.7	3761.2	3432.0	3607.0	3336.0	0.02	1.0000
Eci2	3220.1	3143.9	3218.1	3329.0	3437.6	2784.3	0.01	1.0000
N4bp2l2	3217.9	3117.5	2969.3	3098.6	3022.8	2978.2	-0.03	0.9649
Drg1	3216.9	3106.6	3104.3	2997.8	3004.3	3041.6	-0.04	0.9713
Nras	3215.8	2899.8	3266.5	3186.7	3138.8	3506.2	0.14	0.6881
Slc9a8	3214.7	3184.9	2871.5	3257.9	3053.6	3121.3	-0.01	1.0000
Isca1	3214.7	3236.0	3183.9	3119.9	3143.9	2667.5	-0.11	0.8262
Zfp592	3213.7	3132.1	2932.0	3465.9	3126.5	3557.0	0.10	0.8445
Trerf1	3210.5	3046.5	2957.2	3252.6	2852.3	3152.1	0.01	1.0000
Ipmk	3209.4	2988.2	2927.9	2912.9	3011.5	3010.8	-0.01	1.0000
Cramp1l	3207.3	2941.7	2843.3	3186.7	2745.5	3505.3	0.08	0.9114
Golga1	3207.3	3199.5	2754.6	2752.6	2816.4	2939.2	-0.14	0.7056
Cpt2	3206.2	3054.7	3340.0	2971.3	3404.7	2932.9	0.01	1.0000
Clip2	3206.2	2697.5	2872.5	2946.8	2635.7	2742.7	0.01	1.0000
Ubxn6	3206.2	3286.1	3358.2	2693.1	3111.1	2417.5	-0.23	0.4140
Pi4kb	3205.2	2904.3	3053.9	3109.3	3174.7	3065.1	0.08	0.8995
Rfx3	3205.2	3123.9	2999.5	3066.8	2711.7	2857.7	-0.11	0.8075
Cdk8	3204.1	3138.5	2975.3	3016.9	2745.5	3116.8	-0.07	0.8940
Bsdc1	3204.1	2950.8	3217.1	2659.2	2838.0	2382.2	-0.17	0.6112
Gm27000	3204.1	3347.1	3029.7	2942.6	2303.0	2888.5	-0.25	0.3702
Impa1	3203.0	3012.8	3195.0	3231.3	3291.8	2943.8	0.05	0.9467
Rhoq	3202.0	2610.1	3043.8	2887.4	3228.1	3057.0	0.17	0.5813
Sh3d19	3202.0	2801.4	2412.1	3148.5	3126.5	3237.2	0.15	0.7182
Chmp2a	3200.9	3372.6	3451.9	3238.8	3362.6	2834.2	-0.09	0.9057
Slc12a2	3199.8	3113.0	3173.8	3622.0	3537.2	4026.2	0.23	0.2983
Ipo8	3199.8	3154.0	3076.1	3249.4	3227.1	3322.4	0.05	0.9642
Psmd4	3199.8	3480.1	3344.1	3564.7	3448.9	3245.4	-0.01	1.0000
Phldb1	3198.8	2985.4	3237.3	3350.2	3257.9	3885.8	0.19	0.5557
Jarid2	3195.6	3147.6	2953.1	3530.7	3452.0	3586.0	0.15	0.6561
Rpl14	3195.6	3359.9	4580.3	3580.6	3727.1	3413.0	0.03	0.9748
Junb	3194.5	3262.4	4215.6	14928.5	11945.3	13416.3	1.70	0.0000
Nop58	3194.5	3369.0	3170.8	3723.9	3425.3	3767.1	0.11	0.7835
C2cd5	3194.5	3393.6	2926.9	3123.1	2680.9	3522.6	-0.08	0.8973
Gnl1	3193.4	3247.8	3292.7	3128.4	3337.0	3094.1	-0.02	1.0000
Afg3l1	3191.3	3047.4	3045.8	3066.8	3280.5	2952.8	0.02	0.9884
Trak2	3190.2	3082.9	3069.0	3445.8	3216.8	3671.1	0.14	0.6871
Tmem123	3190.2	3081.1	3259.4	2893.8	3270.2	2968.2	-0.02	0.9877
Bdp1	3189.2	2969.9	2642.8	3210.1	2783.5	3392.1	0.07	0.9456
Slc39a6	3186.0	3252.4	3138.5	3201.6	3201.4	3293.4	0.00	1.0000
Ubxn1	3186.0	3107.5	3283.6	2990.4	3303.1	2604.1	-0.07	0.9500
Fastk	3186.0	3099.3	2826.2	2996.7	3093.6	2573.3	-0.08	0.8859
Ensa	3184.9	3215.9	3135.5	3183.6	3243.5	2946.5	-0.03	0.9649
Gprasp2	3183.8	2803.2	3240.3	2519.0	2911.9	2545.2	-0.10	0.8762
Aga	3181.7	3195.9	2919.9	2893.8	3558.7	2627.6	-0.06	0.9744
Mta3	3181.7	3266.9	3399.5	3153.8	3077.2	2766.2	-0.11	0.8240
Rbm47	3180.6	2670.2	2728.5	3370.4	3192.2	3164.8	0.23	0.3309
Shprh	3178.5	2800.5	2795.0	2908.6	2760.9	3320.6	0.07	0.9361
Wbp1l	3178.5	3246.0	3341.0	3247.3	3223.0	3257.2	0.00	1.0000
Smarca5	3176.4	3222.3	2999.5	3693.1	3454.0	3278.0	0.11	0.8051
Eif3i	3176.4	3503.8	3571.8	3481.9	3478.6	2913.9	-0.07	0.9108
Med15	3173.2	3189.5	3246.3	3241.9	3352.4	3432.9	0.06	0.9215
Prdx5	3172.1	3576.7	3567.7	2990.4	3405.7	2640.3	-0.20	0.5343
Klf7	3171.1	3081.1	3410.6	3920.3	3617.3	4092.3	0.29	0.1235
Creg1	3171.1	3416.3	3402.5	3667.6	3573.1	2833.3	-0.01	0.9988
Cops4	3170.0	3129.4	3126.4	2853.4	3002.2	2898.5	-0.09	0.8599
Mib2	3167.9	2807.8	2899.7	2501.0	2704.5	2440.2	-0.14	0.7056
Psme3	3166.8	2898.0	3023.7	3049.8	3243.5	3184.7	0.10	0.8059
Prpf18	3166.8	3074.7	3045.8	3141.1	3170.6	2940.1	0.00	1.0000
Trappc9	3165.7	3339.8	2911.8	3723.9	3746.6	3672.9	0.16	0.6042
Csnk1e	3165.7	3017.3	3588.9	3172.9	3188.1	3026.2	0.03	0.9858
Eif4e	3164.7	3098.4	2900.7	3103.9	3228.1	2586.0	-0.05	0.9521
Yipf3	3163.6	3072.9	3079.1	2974.4	3390.3	2878.5	0.00	1.0000
Pitpnc1	3162.5	2845.1	2962.2	3327.9	3145.0	3333.2	0.17	0.5478

Gnai1	3162.5	3562.1	3648.3	3206.9	3045.4	2912.1	-0.19	0.5016
Uhrf1bp1l	3161.5	2950.8	2946.1	3044.5	2697.3	3095.0	-0.01	1.0000
Smu1	3160.4	3397.2	3266.5	2868.3	3182.9	2874.9	-0.16	0.5985
Rgs16	3159.3	2183.7	2792.9	2974.4	2693.2	2391.2	0.18	0.6162
Abl2	3159.3	3058.3	2924.9	3253.6	2957.1	3646.6	0.09	0.8613
Smg6	3159.3	2989.1	3123.4	3111.4	3095.7	3392.1	0.08	0.8714
Ogfr	3158.3	2982.7	2685.1	2719.7	3008.4	2786.2	-0.06	0.9314
Unc119b	3157.2	3001.8	2885.6	2684.6	3000.2	2647.6	-0.10	0.8234
Angel2	3156.1	3205.0	3174.8	3025.4	3101.8	2720.0	-0.11	0.8010
Mob4	3152.9	2862.4	3100.2	2823.7	3054.6	2602.3	-0.03	0.9748
Tjp2	3151.9	3321.6	3302.8	3509.5	3305.1	3692.8	0.07	0.9126
Rnf115	3151.9	2892.5	3354.1	3140.0	3276.4	2871.3	0.07	0.9379
Slc37a4	3151.9	3183.1	2961.2	2863.0	3017.6	2366.8	-0.18	0.6656
Pfdn5	3150.8	3164.0	3290.7	2805.7	3147.0	2682.9	-0.13	0.7600
Fbxo42	3149.7	2929.9	2731.5	3105.0	2910.9	3206.4	0.06	0.9497
Cd2bp2	3149.7	3180.4	3212.1	3170.8	3101.8	3155.7	-0.02	0.9931
Pdk4	3148.7	3503.8	4061.4	3496.7	3650.1	2585.1	-0.10	0.9126
Btbd2	3146.5	3083.8	3340.0	3039.2	3142.9	2893.9	-0.03	0.9654
Zfp318	3146.5	3107.5	2770.8	2825.8	2687.0	3009.0	-0.11	0.8494
Luzp1	3145.5	3028.2	2864.5	3339.6	3282.5	4110.4	0.21	0.5267
Gabbr2	3144.4	2534.5	3200.0	2702.7	2856.4	3488.1	0.16	0.7168
Eif3k	3144.4	3055.6	3058.9	3026.5	3249.7	2626.7	-0.04	0.9884
Eps15l1	3144.4	3262.4	3195.0	3229.2	2856.4	3309.7	-0.05	0.9534
Pogk	3142.3	3098.4	2867.5	3103.9	2928.3	3393.9	0.02	0.9831
Galnt18	3142.3	3046.5	3552.6	2654.9	3071.0	2577.8	-0.14	0.7604
Blcap	3140.2	2819.6	2885.6	2738.8	2863.6	2668.4	-0.04	0.9765
Clk3	3139.1	3219.6	3007.5	3116.7	3083.3	2913.0	-0.07	0.9244
Mob1b	3138.0	2475.3	2924.9	3070.0	2828.7	3127.6	0.22	0.3583
Msantd4	3138.0	2875.2	2904.8	3070.0	3036.1	2744.5	0.02	0.9857
Jmjd8	3138.0	3277.9	3162.7	3107.1	3312.3	2734.5	-0.08	0.8745
Sri	3138.0	3099.3	3206.0	2822.6	3134.7	2791.6	-0.08	0.8910
Entr1	3137.0	3148.5	2904.8	2999.9	2863.6	2741.8	-0.11	0.7795
Esm1	3135.9	2812.3	3855.9	3420.3	4700.5	3402.1	0.30	0.2820
Plekhh2	3135.9	3257.8	2698.2	2823.7	2786.6	2820.6	-0.17	0.6107
Arap3	3134.8	2793.2	2815.1	3425.6	3099.8	3453.7	0.21	0.3948
Klhdc3	3133.8	3299.7	3361.2	3030.7	3334.9	3044.3	-0.06	0.9168
Gm28437	3131.6	3088.4	3282.6	3097.6	3175.8	2704.6	-0.05	0.9681
Wdr48	3125.2	3018.2	2765.7	2949.0	2718.8	2834.2	-0.08	0.8926
Psmd7	3124.2	3425.5	3331.0	3029.6	3248.7	2938.3	-0.13	0.7192
Rbm27	3123.1	2947.2	2865.5	3383.1	3089.5	3282.5	0.13	0.7470
Chid1	3123.1	3453.7	3262.5	2757.9	3360.6	2895.8	-0.16	0.6796
Zfx	3122.0	2824.2	2913.8	3167.6	3076.2	3258.1	0.14	0.6819
Kmt5c	3117.8	3002.7	2960.2	2859.8	2770.2	2780.7	-0.09	0.8314
Cdon	3115.6	3147.6	3427.7	3100.8	3150.1	3490.9	0.03	0.9896
Bnip3l	3114.6	3039.2	3612.1	2961.7	3196.3	2692.0	-0.06	0.9318
Ube2d2a	3112.4	2993.6	3059.9	2779.1	2975.5	3022.6	-0.04	0.9694
Msln	3112.4	2701.2	2448.4	2421.4	1983.7	2489.1	-0.21	0.5633
Robo4	3110.3	3145.8	3195.0	3884.2	4185.1	4189.2	0.34	0.0252
Klf3	3110.3	3033.7	3045.8	3430.9	3278.4	3850.4	0.19	0.5053
Kctd10	3110.3	3271.5	3685.6	3495.7	3402.7	3919.3	0.12	0.8037
Slc25a12	3110.3	3009.1	2997.5	3228.1	3322.6	2977.3	0.07	0.9313
Tbc1d10b	3109.3	2861.5	2868.5	2951.1	2983.8	2980.9	0.04	0.9810
Atp1b2	3109.3	2770.4	3085.1	2521.2	2835.9	3333.2	0.03	1.0000
Neu1	3105.0	3057.4	3022.7	2858.7	3244.5	2494.5	-0.08	0.8899
Flcn	3105.0	3103.9	3040.8	2660.2	3097.7	2352.3	-0.17	0.6881
Xrcc6	3103.9	3062.9	3099.2	3244.1	3250.7	3112.2	0.06	0.9486
Mospd2	3103.9	2617.4	2601.5	2762.1	2706.5	2538.0	0.01	1.0000
Shfl	3103.9	2973.6	2887.6	2817.3	2546.4	2733.6	-0.13	0.7648
Elmo2	3102.9	2923.5	3023.7	3093.3	2946.8	2926.6	0.02	0.9850
Ciz1	3102.9	3004.6	2947.1	3016.9	2736.3	3028.9	-0.04	0.9929
Usp10	3101.8	3054.7	2985.4	3283.3	3039.2	3183.8	0.05	0.9624
Ccdc186	3100.7	3043.7	2933.0	3247.3	3057.7	3387.6	0.08	0.8717
Aopep	3099.7	3097.5	3445.8	3354.5	3346.2	3348.6	0.09	0.8598
Dok4	3099.7	2848.8	2986.4	2837.5	2943.7	2517.1	-0.05	0.9436
Itga3	3097.5	3062.0	3375.3	2866.2	3062.8	3183.8	-0.02	0.9925
Phc3	3096.5	2681.1	2648.9	2976.6	2694.2	3522.6	0.15	0.7786
Tmem132b	3096.5	3069.2	2826.2	2430.9	2591.5	2489.1	-0.26	0.2167
Tpt1	3095.4	3696.9	3570.8	3215.4	3419.1	3081.4	-0.15	0.6983
Clpx	3095.4	3403.6	3100.2	3073.2	3103.9	2729.1	-0.16	0.6549
Fitm2	3093.3	2928.0	2896.7	2625.2	2895.5	2457.4	-0.13	0.7797
Zc3h13	3091.1	2965.4	3133.5	3584.8	3304.1	4073.3	0.26	0.2832
Amz2	3091.1	3304.3	3174.8	3261.1	3568.0	3159.3	0.02	1.0000
Tspan18	3090.1	3046.5	3309.8	3200.5	2922.1	3762.6	0.09	0.9157
Plaa	3090.1	3070.2	2788.9	3004.2	2943.7	2857.7	-0.05	0.9676
Chd1	3084.7	2737.6	2760.7	3377.8	2781.5	3335.1	0.17	0.6469
Cnot2	3083.7	2986.3	3279.6	3355.5	3216.8	3394.8	0.13	0.7147

Sart3	3083.7	3067.4	2912.8	3011.6	2898.5	3269.8	0.00	1.0000
Deaf1	3083.7	3407.2	3655.4	3257.9	3440.7	2894.9	-0.08	0.8948
Usp31	3082.6	2836.9	2663.0	3037.1	2942.7	3349.5	0.12	0.7910
Arl2bp	3081.5	3052.8	3511.3	3236.6	3324.6	3249.0	0.08	0.8938
Atraid	3081.5	3019.1	3209.1	2678.3	3259.9	2736.3	-0.06	0.9273
Cdk12	3080.5	2737.6	2449.4	3320.5	2889.3	3470.0	0.20	0.5830
Agtrap	3080.5	2783.2	2889.7	2437.3	2587.4	2367.7	-0.17	0.5618
Ift20	3079.4	3370.8	2979.3	2679.3	2944.7	2346.9	-0.28	0.2109
Ptgr2	3077.3	2728.5	3020.6	2573.2	2928.3	2534.4	-0.05	0.9521
Dcaf10	3075.2	2695.7	2602.5	2861.9	2855.4	3108.6	0.10	0.8366
Kctd3	3074.1	3398.1	3438.8	3232.4	3222.0	3116.8	-0.08	0.9207
Tle1	3074.1	3403.6	3385.4	3007.3	2899.6	2783.4	-0.20	0.4250
Heca	3073.0	2927.1	3199.0	3253.6	3235.3	3009.0	0.09	0.8461
Calcoco1	3072.0	2943.5	3781.3	3068.9	3447.8	3076.0	0.07	0.9498
Ehd4	3070.9	2902.5	3103.3	3772.7	3953.0	4238.1	0.41	0.0054
Bcam	3070.9	3098.4	3417.6	3728.1	3850.3	4403.0	0.32	0.1113
Arvcf	3070.9	2980.9	3200.0	2856.6	2879.0	2871.3	-0.06	0.9500
Syn2	3068.8	2286.7	3701.7	2839.6	2830.8	2997.2	0.21	0.4381
Smap1	3068.8	2983.6	3212.1	3269.5	3211.7	3138.5	0.09	0.8448
Asph	3065.6	2642.9	2461.5	2645.4	2611.0	3004.4	0.04	0.9649
BC004004	3064.5	3291.5	3184.9	3156.0	3013.5	2697.4	-0.13	0.7698
Tbc1d2b	3062.4	2834.2	2998.5	2841.7	3053.6	3384.9	0.10	0.8370
Trpm5	3062.4	3062.0	3087.1	3147.5	3198.3	2870.4	0.00	1.0000
Selenoi	3061.3	2578.2	2773.8	3400.1	3003.3	3076.0	0.24	0.3299
Gdap1	3060.2	2713.0	2893.7	2940.5	2976.6	2797.0	0.07	0.8884
Smad2	3059.2	3012.8	2940.0	3384.2	3085.4	3323.3	0.11	0.7955
Ash2l	3059.2	2931.7	2945.1	3137.9	3002.2	2884.0	0.03	0.9932
Phf8	3058.1	2734.9	2510.8	3077.4	3198.3	3393.9	0.21	0.4852
Ilf2	3058.1	3078.4	3049.9	2994.6	2927.3	3074.2	-0.03	0.9931
Cplane1	3056.0	3109.3	2497.7	3196.3	2747.6	3478.2	0.04	0.9863
Usp29	3054.9	2969.9	3026.7	3559.3	3542.3	3278.0	0.20	0.4177
Gp2	3054.9	5120.9	5578.8	6167.6	4550.6	8337.6	0.20	0.7636
Rps4l	3054.9	3083.8	3228.2	2939.4	3258.9	2536.2	-0.08	0.9297
Dda1	3053.8	2896.1	2959.2	2705.9	2593.6	2346.9	-0.17	0.5785
Ppp2r5e	3052.8	3162.2	2860.4	3307.8	3194.2	3297.9	0.05	0.9334
Abhd17a	3052.8	3129.4	3089.2	2817.3	3239.4	2400.3	-0.13	0.7943
Rpl6	3051.7	3112.1	3319.9	3165.5	3045.4	2783.4	-0.05	0.9411
Prpf39	3050.6	2993.6	2768.8	3320.5	2798.9	3470.9	0.09	0.8710
Gm13436	3050.6	3420.9	3439.8	3223.9	3410.9	2963.7	-0.08	0.8948
Zbtb44	3047.5	2724.9	2745.6	2909.7	2950.9	3189.2	0.12	0.7978
Shc2	3044.3	3126.6	3197.0	2805.7	3000.2	2451.0	-0.16	0.6422
Cpeb1	3043.2	3006.4	2578.3	3270.6	3219.9	3451.9	0.14	0.7147
Cox6b1	3043.2	3069.2	3421.6	3120.9	3150.1	2796.1	-0.03	1.0000
Patz1	3042.1	3214.1	3137.5	2950.0	3027.9	2891.2	-0.10	0.8207
Ccdc127	3041.1	2629.2	2431.2	2947.9	2718.8	2964.6	0.10	0.8719
Cfap36	3041.1	3195.0	3035.8	2915.0	3302.0	2810.6	-0.07	0.9362
Abcc4	3038.9	3103.9	3305.8	3244.1	3152.1	3132.2	0.03	0.9778
Dipk2a	3036.8	3259.6	2859.4	3232.4	2896.5	3115.0	-0.06	0.9343
Rcor3	3036.8	3144.9	3010.6	2900.1	2883.1	3089.6	-0.07	0.9110
Eva1b	3035.7	2903.4	2932.0	2477.6	3004.3	2339.6	-0.14	0.7524
Hist1h2bc	3035.7	3268.8	3298.7	2652.8	2954.0	2064.3	-0.29	0.2719
Prep	3034.7	3082.9	3052.9	3213.3	3100.8	3124.0	0.03	0.9742
Mnt	3034.7	3009.1	2856.4	2807.8	2753.8	2811.5	-0.10	0.8477
Gatad1	3033.6	2664.7	3018.6	2939.4	2955.0	2585.1	0.05	0.9471
Rgs3	3032.5	2896.1	2808.0	3298.2	3024.8	3599.5	0.17	0.6343
Ssh3	3032.5	3093.8	2996.5	2761.1	2606.9	2624.9	-0.19	0.4356
Slc23a2	3031.5	2805.0	2766.7	3233.5	3149.1	3468.2	0.20	0.4507
Xrn2	3031.5	2873.4	2784.9	3169.8	3196.3	3117.7	0.12	0.7470
Nufip2	3029.3	2568.2	2581.4	3208.0	2924.2	3043.4	0.21	0.4578
Loxl2	3029.3	2915.3	2998.5	2976.6	3269.2	3430.2	0.13	0.7571
Sipa1l1	3029.3	3012.8	2742.6	3219.7	2949.9	3327.8	0.07	0.9244
Prxl2c	3029.3	2870.6	3326.9	2938.3	3171.6	2448.3	-0.03	1.0000
Cox6a1	3029.3	3505.6	3292.7	3204.8	3447.8	3280.7	-0.06	0.9557
Pls3	3028.3	3008.2	3557.7	3354.5	3516.6	3923.8	0.21	0.4723
Gabbr1	3028.3	2948.1	3096.2	3032.8	2529.9	2980.0	-0.05	0.9452
Zfp335	3027.2	3183.1	2767.7	2865.1	2613.1	2877.6	-0.16	0.6752
Naa30	3026.1	2288.5	2594.4	2735.6	2770.2	2564.2	0.16	0.6571
Klhl42	3025.1	2439.7	2574.3	2620.9	2795.9	2641.2	0.09	0.8548
Ulk2	3025.1	2936.2	3042.8	3085.9	2932.4	3171.1	0.05	0.9377
Armc8	3022.9	2813.2	2651.9	2876.8	2976.6	2851.4	0.04	0.9884
Jade2	3022.9	2691.2	2985.4	2595.5	2783.5	2880.4	0.01	1.0000
Eri3	3022.9	3075.6	3233.2	2787.6	2963.2	2698.3	-0.12	0.7702
Gria2	3020.8	3297.0	2779.8	3628.3	2702.4	3657.5	0.05	0.9662
Gm9816	3020.8	2995.5	3129.5	3097.6	3283.6	2892.1	0.04	0.9649
Glt8d1	3020.8	3145.8	3015.6	2498.9	2810.2	2657.5	-0.21	0.3900
Tmem33	3019.7	2927.1	3137.5	3040.3	3103.9	3102.3	0.06	0.9148

Fyco1	3018.7	2722.1	2751.6	2791.9	2703.4	3181.1	0.07	0.9457
Paip1	3015.5	2990.0	3266.5	3036.0	3072.1	2905.7	0.00	1.0000
Hs2st1	3014.4	3021.0	3149.6	2977.6	3027.9	3307.0	0.03	0.9764
Rnf220	3014.4	3130.3	2989.4	3194.2	3023.8	2629.5	-0.07	0.9232
Gpx4-ps2	3014.4	3342.6	3423.7	2985.1	3381.1	2369.5	-0.16	0.7618
Ppp2r3d	3014.4	3264.2	2816.1	2691.0	2839.0	2627.6	-0.22	0.3287
Tsen34	3012.3	2918.0	3113.3	2652.8	2794.8	2280.7	-0.17	0.6622
Tmem56	3010.2	2689.3	2454.4	2831.1	2466.3	2453.7	-0.05	0.9731
Dele1	3010.2	3405.4	2690.2	3036.0	2856.4	3047.0	-0.14	0.6889
Cisd2	3007.0	3018.2	2494.7	2958.5	3079.2	3165.7	0.04	0.9676
Sec11c	3007.0	3362.6	3015.6	3263.2	3089.5	2906.6	-0.09	0.8410
Rgl1	3004.8	2904.3	2798.0	3176.1	3035.1	3432.0	0.13	0.7535
Wdtc1	3004.8	3244.2	3257.4	3084.8	2984.8	3031.6	-0.08	0.8874
Ankib1	3003.8	2781.4	2674.0	3040.3	3005.3	3081.4	0.11	0.8124
Rpl9-ps6	3003.8	2864.3	3330.0	2775.9	2949.9	2049.8	-0.15	0.7572
Fam117b	3002.7	2701.2	2912.8	3093.3	3202.5	3268.9	0.20	0.3696
Rbmx	3000.6	2995.5	2596.5	3282.3	2967.3	3004.4	0.05	0.9507
Camsap1	3000.6	2845.1	2742.6	3061.5	2653.1	3110.4	0.04	0.9827
Shisa5	3000.6	3220.5	2985.4	2775.9	3136.7	2994.5	-0.09	0.8748
Sfmbt1	2997.4	2881.6	2609.6	3078.5	3042.3	3100.5	0.09	0.8545
Mark4	2997.4	2785.9	3389.4	2885.3	3027.9	3030.7	0.06	0.9267
B3galt5	2996.3	3283.3	3198.0	2592.3	2890.3	2950.1	-0.19	0.4954
Dhx40	2995.2	3064.7	3013.6	2899.1	3196.3	2990.0	-0.01	0.9967
Cyb561	2994.2	3308.8	3143.6	3428.8	3707.6	3665.7	0.12	0.7378
Ncoa7	2994.2	2663.8	2868.5	3063.6	3003.3	2811.5	0.12	0.7546
Trim3	2994.2	2645.6	2869.5	2751.5	2745.5	2750.8	0.03	0.9729
Hdac11	2993.1	2658.4	3056.9	2559.4	2746.6	2307.9	-0.09	0.8710
Myo1d	2991.0	2774.1	2673.0	3027.5	3096.7	3414.8	0.17	0.5873
Nectin1	2991.0	2323.1	2898.7	2463.8	2421.1	2363.2	0.01	1.0000
Trafd1	2991.0	3056.5	2728.5	2841.7	2995.0	2612.3	-0.09	0.8762
Kif1bp	2989.9	2744.9	2771.8	2703.7	2835.9	2468.2	-0.05	0.9522
Slc4a8	2989.9	3027.3	2507.8	2755.8	2537.1	3073.3	-0.08	0.9320
Btbd3	2988.8	2754.0	3040.8	3448.9	3321.6	3550.6	0.28	0.1252
Slc31a1	2988.8	3033.7	2822.2	2949.0	2960.1	3092.3	-0.01	1.0000
Icmt	2987.8	2831.5	2907.8	2783.4	2848.2	2932.0	0.00	1.0000
Ero1l	2987.8	3010.0	2848.4	2816.3	2988.9	2924.7	-0.04	0.9805
Racgap1	2984.6	2689.3	1966.7	2480.8	2604.9	3110.4	0.04	0.9681
Kcmf1	2983.5	2795.9	2980.3	3232.4	3225.0	3011.7	0.15	0.6372
Bicd2	2983.5	2734.0	2610.6	2989.3	2870.8	3051.6	0.10	0.8202
Agtpbp1	2983.5	2987.3	2850.4	3374.6	2749.7	3266.2	0.06	0.9521
Exosc10	2983.5	3072.9	2977.3	3166.6	3087.5	3099.6	0.02	0.9826
Atp5j	2983.5	3044.6	3285.6	2997.8	3211.7	2654.8	-0.04	0.9542
Vps35l	2978.2	2739.5	2909.8	2867.2	2856.4	2810.6	0.04	0.9651
Lrp12	2978.2	3172.2	3305.8	3217.5	3202.5	3024.4	-0.01	1.0000
Acp2	2977.1	2750.4	2918.9	2842.8	2816.4	2804.3	0.02	0.9834
Adh5	2977.1	2945.3	3027.7	2964.9	3072.1	2583.3	-0.04	0.9881
Rnf123	2977.1	3010.0	2987.4	2708.0	2832.8	2759.0	-0.11	0.8000
Gm10039	2977.1	3534.8	3184.9	2663.4	2469.3	2337.8	-0.43	0.0036
Serf2	2975.0	2842.4	2534.0	2626.3	2563.8	2277.1	-0.16	0.6875
Ehmt1	2973.9	2961.7	3054.9	3050.9	2895.5	3368.6	0.06	0.9303
Cul4b	2972.9	2858.8	3089.2	3260.0	3435.5	3043.4	0.15	0.6287
Tars	2970.7	2877.9	2721.4	2999.9	2990.9	2910.2	0.04	0.9588
Zdhhc1	2970.7	3017.3	2958.2	2609.3	2514.5	2607.7	-0.21	0.3583
Mtfr1l	2969.7	2757.7	2647.8	2673.0	2896.5	2579.6	-0.02	0.9863
Bach1	2968.6	2797.8	2911.8	3049.8	3042.3	3249.0	0.13	0.7326
Ndufa4	2965.4	3031.0	3142.6	2477.6	2717.8	2555.2	-0.21	0.3818
Phf21b	2964.3	2952.6	2895.7	2814.1	2604.9	2635.8	-0.12	0.7414
Ube2i	2964.3	3202.3	2808.0	2459.6	2408.8	2679.3	-0.30	0.1098
Ncbp2	2962.2	2470.7	2690.2	2646.4	2792.8	2410.3	0.05	0.9552
Apbb2	2961.1	2591.0	2819.1	3548.7	3325.7	3656.6	0.38	0.0112
Mcm3ap	2960.1	3141.2	2820.1	3147.5	3114.2	3324.2	0.04	0.9593
Gm16286	2960.1	3225.0	3303.8	3089.1	3212.7	2687.4	-0.09	0.8772
Dmwd	2956.9	2963.6	3058.9	2981.9	2929.3	2764.4	-0.03	0.9918
Gm2223	2956.9	1939.6	2609.6	2005.3	1858.4	1854.1	-0.10	0.8340
Smarcd2	2956.9	3143.9	3418.6	3008.4	2888.3	2829.6	-0.10	0.8518
Lgi2	2956.9	3383.5	2723.4	2876.8	2769.2	3194.7	-0.14	0.7367
Ift81	2954.7	2803.2	2772.8	2714.4	2841.0	2658.4	-0.03	0.9864
Gm6180	2953.7	3103.0	3341.0	3509.5	3354.4	3163.9	0.09	0.8717
Ift122	2953.7	2887.0	2886.6	3001.0	3237.4	2790.7	0.05	0.9495
Men1	2952.6	2934.4	2900.7	2774.9	2861.6	2760.8	-0.06	0.9369
Tmem183a	2951.6	2947.2	2692.2	3082.7	3246.6	2842.3	0.06	0.9343
Fkbp15	2951.6	2777.7	2733.5	2852.4	2900.6	3028.9	0.06	0.9436
Pip5k1a	2950.5	2651.1	2773.8	2861.9	2881.1	2705.5	0.07	0.9155
Mfsd1	2949.4	2728.5	2971.3	2705.9	2868.8	2627.6	-0.01	1.0000
Zmym5	2948.4	3006.4	3015.6	3188.9	3017.6	3172.0	0.05	0.9391
Rab33b	2947.3	2673.9	2435.3	2481.9	2813.3	2451.0	-0.05	0.9573

Rabgap1l	2945.2	2986.3	2757.7	3255.7	3054.6	3254.4	0.09	0.8314
Hbs1l	2945.2	3131.2	2907.8	2896.9	3167.5	3036.2	-0.03	0.9721
Zfhx3	2944.1	2637.4	2688.2	2943.7	2758.9	3774.4	0.20	0.5772
Rnf114	2943.0	2825.1	2853.4	2714.4	2950.9	2283.5	-0.09	0.8792
Relch	2942.0	2897.1	2842.3	2972.3	2608.0	3032.5	-0.01	1.0000
Aggf1	2940.9	2592.8	2731.5	2779.1	2629.5	2504.5	0.00	1.0000
Senp3	2940.9	2764.0	2942.1	2706.9	2762.0	2530.7	-0.06	0.9522
Snx14	2940.9	2870.6	3195.0	2762.1	2848.2	2496.3	-0.09	0.8772
Akt2	2939.8	2995.5	3180.8	2785.5	3037.1	3054.3	-0.02	0.9877
Fam234b	2938.8	2766.8	3109.3	2974.4	2880.1	2926.6	0.06	0.9515
Nes	2937.7	2640.2	2763.7	3473.4	3179.9	4047.0	0.36	0.0613
Fzr1	2936.6	2849.7	2814.1	2748.3	2688.0	2628.6	-0.08	0.9093
Fam114a1	2935.6	3331.6	2730.5	3223.9	3055.6	2852.3	-0.09	0.8666
Oxsr1	2935.6	2988.2	2712.3	2680.4	2690.1	2625.8	-0.14	0.7161
Sema4b	2934.5	2911.6	3001.5	2910.7	2978.6	3004.4	0.02	1.0000
Btaf1	2933.4	2655.6	2355.7	3132.6	2854.4	4140.3	0.28	0.3320
Atp1b3	2933.4	2813.2	2819.1	3030.7	3235.3	3168.4	0.14	0.6679
Tpp1	2933.4	2871.6	2649.9	2543.5	2755.8	2698.3	-0.09	0.8816
Chst11	2931.3	2289.4	2399.0	2543.5	2711.7	2926.6	0.18	0.6131
Lmnb1	2931.3	3076.5	2762.7	3160.2	2886.2	3639.4	0.08	0.8938
Lrch1	2931.3	3203.2	3177.8	3135.8	3140.8	3389.4	0.02	0.9981
Psmc6	2931.3	2989.1	2927.9	2725.0	2736.3	2511.7	-0.15	0.6326
Plcl2	2930.2	2729.4	2590.4	3045.6	3128.5	2657.5	0.09	0.8551
Ptges3	2930.2	2885.2	2961.2	2818.4	2978.6	2953.7	0.01	1.0000
Pacs1	2929.2	2976.3	2916.9	3029.6	3145.0	3413.0	0.10	0.8642
Cyc1	2929.2	3060.1	3284.6	3032.8	3147.0	2555.2	-0.07	0.9232
Got2	2928.1	3021.9	3224.2	3099.7	3269.2	3237.2	0.07	0.8874
Ptdss1	2928.1	2867.9	3128.5	2831.1	3133.7	2840.5	0.02	0.9893
Ankle2	2928.1	2927.1	2789.9	2952.1	2939.6	2960.1	0.01	0.9956
Ctsz	2928.1	2961.7	3029.7	2656.0	2822.6	2855.0	-0.09	0.8558
Rpl34-ps1	2927.0	2861.5	3071.0	2662.3	3008.4	2425.7	-0.09	0.8862
Ube2w	2926.0	2657.5	2869.5	2670.8	3099.8	2470.9	0.02	0.9884
Fbxl20	2926.0	3354.4	3324.9	3254.7	2875.9	3046.1	-0.10	0.8170
Osbpl8	2924.9	2558.2	2498.7	2781.2	2972.5	2755.4	0.12	0.7805
Zfp869	2923.8	2648.3	2464.5	2537.1	2688.0	2630.4	-0.02	0.9931
Zhx3	2922.8	2839.7	2557.2	3265.3	3128.5	3527.1	0.20	0.4916
Capn15	2921.7	2672.0	2387.9	2885.3	2548.4	2788.9	0.03	0.9931
Pdcd11	2921.7	3105.7	2586.4	3046.6	2758.9	3317.8	0.00	1.0000
Ptprj	2920.7	2472.5	2693.2	3060.4	2964.2	3189.2	0.26	0.1890
Gm12191	2920.7	3040.1	3411.6	2827.9	3283.6	3117.7	0.01	1.0000
Slc7a1	2918.5	3115.7	2753.6	3368.3	2867.7	3433.8	0.06	0.9237
Ddx19a	2918.5	2979.1	2842.3	2874.7	3015.6	3012.6	0.00	1.0000
Atp5j2	2918.5	3174.0	2900.7	2736.7	2873.9	2550.7	-0.19	0.5349
Sf3b6	2917.5	2986.3	3004.5	2496.7	2904.7	2106.8	-0.22	0.5402
Zfp92	2916.4	2662.9	2596.5	2518.0	2356.4	2476.4	-0.11	0.8200
C2cd4b	2914.3	2867.9	3032.7	7254.6	4836.0	4249.9	0.52	0.0693
Hgs	2914.3	2797.8	2749.6	2796.1	2827.7	2931.1	0.02	1.0000
Sox13	2914.3	2875.2	3134.5	2853.4	2714.7	2861.3	-0.04	0.9649
Rnf111	2913.2	2569.1	2841.3	3092.3	2949.9	3015.3	0.19	0.4260
Dcaf1	2912.1	3059.2	3016.6	3263.2	3097.7	3220.0	0.06	0.9193
Rab11fip3	2910.0	3055.6	3090.2	3140.0	2843.1	3111.3	-0.01	1.0000
Dnaja1	2908.9	2740.4	2897.7	2846.0	2903.7	2628.6	0.01	1.0000
Map3k4	2907.9	2893.4	2545.1	3060.4	2895.5	3089.6	0.07	0.9395
Mgat5	2907.9	2752.2	2729.5	2924.5	2634.7	2844.1	0.02	1.0000
Casd1	2906.8	2857.9	2772.8	2785.5	2815.4	2845.9	-0.02	0.9895
Unc79	2905.7	2714.9	2250.9	3266.4	2766.1	3023.5	0.14	0.7470
Mtmr9	2904.7	2627.4	2423.2	2613.5	2558.7	2778.9	0.01	1.0000
Tmod3	2904.7	2883.4	2782.9	2839.6	2778.4	2990.0	0.00	1.0000
Gm42047	2904.7	1773.8	2606.5	1394.9	2979.6	999.1	-0.12	0.9229
Arl6ip5	2903.6	2550.0	2813.1	2601.8	2939.6	2423.8	0.03	1.0000
Rchy1	2903.6	2657.5	2743.6	2695.3	2806.1	2480.0	-0.01	1.0000
Cbx4	2903.6	2945.3	2586.4	2709.1	2546.4	2661.2	-0.13	0.7442
1810013L24Ri	2901.5	2600.1	2670.0	3146.4	3210.7	3212.8	0.26	0.1834
Nol6	2901.5	3113.9	2698.2	3175.1	2804.1	3507.2	0.04	0.9649
Gm11361	2901.5	3193.1	3634.2	2992.5	3384.2	2556.1	-0.09	0.8907
Sike1	2900.4	2617.4	2749.6	2700.6	2591.5	2587.8	-0.01	1.0000
Nkiras2	2900.4	3390.8	3518.4	2728.2	3112.1	2455.6	-0.24	0.4117
Atrnl1	2898.3	2708.5	2754.6	2942.6	2699.3	3203.7	0.10	0.8316
Gm10334	2898.3	6030.1	5211.1	6032.7	3667.6	6149.3	0.05	0.9741
Setdb1	2898.3	2785.0	2674.0	2912.9	2610.0	2823.3	0.00	1.0000
Ncbp1	2897.2	3410.9	2796.0	3681.4	3520.7	3364.9	0.07	0.8826
Brip1os	2897.2	3086.6	2569.3	2896.9	2956.0	3274.4	0.01	1.0000
Pi4k2a	2897.2	2667.5	2724.4	2617.8	2800.0	2528.0	-0.02	0.9881
Rapgef2	2896.1	2865.2	2848.4	3369.3	3230.2	3522.6	0.21	0.3318
Med16	2896.1	2721.2	2879.6	2799.3	2979.6	2725.5	0.04	0.9516
Noc2l	2896.1	2787.7	2631.7	2843.9	2921.1	2781.6	0.03	0.9748

Ankrd33b	2895.1	2408.8	2608.6	2504.2	2468.3	2153.9	-0.05	0.9534
Cdk9	2895.1	2867.0	3142.6	2964.9	2781.5	2484.5	-0.07	0.9134
Fbf1	2895.1	2751.3	2700.2	2530.7	2507.3	2672.0	-0.09	0.8748
Rragb	2894.0	2774.1	3197.0	2779.1	2998.1	2628.6	-0.01	1.0000
Pskh1	2892.9	2963.6	2933.0	2956.4	3137.8	2849.6	0.01	1.0000
Otub1	2892.9	3118.4	3165.7	3137.9	3194.2	2855.0	-0.02	0.9923
Unc5c	2891.9	2328.6	2718.4	2661.3	2395.4	2790.7	0.11	0.8059
Epc1	2888.7	3063.8	3048.9	3183.6	2944.7	3092.3	0.01	1.0000
Agps	2888.7	2991.8	2737.5	2785.5	2887.2	2929.3	-0.05	0.9681
Hmg20a	2886.6	2855.2	2942.1	2899.1	2686.0	3085.1	0.01	1.0000
Slc44a3	2886.6	2942.6	2666.0	2863.0	2793.8	2768.9	-0.05	0.9581
Smpd1	2885.5	3043.7	2739.5	2927.7	2943.7	2780.7	-0.06	0.9244
Syt11	2884.4	2566.4	3809.6	2917.1	3066.9	3066.0	0.13	0.8001
Bicral	2884.4	2890.7	2767.7	3145.3	3057.7	3071.5	0.09	0.8284
Cdc5l	2884.4	2822.4	2939.0	2901.2	2907.8	2702.8	0.00	1.0000
Pex5	2882.3	2664.7	2549.1	2687.8	2586.4	2466.4	-0.05	0.9729
Maf1	2881.2	3078.4	3322.9	2904.4	3123.4	2627.6	-0.08	0.8779
Tshz3	2880.2	2636.5	2560.2	3071.0	2842.1	2978.2	0.15	0.7074
Csnk2a2	2880.2	2668.4	2736.5	2939.4	2850.3	2889.4	0.10	0.8225
Gusb	2880.2	2833.3	2902.8	2582.7	2860.5	2699.2	-0.06	0.9215
Vps54	2880.2	2784.1	2630.7	2635.8	2657.2	2692.9	-0.06	0.9312
Vwa1	2879.1	2625.6	2662.0	2590.2	2681.9	2543.4	-0.02	1.0000
Robo1	2878.0	2992.7	2960.2	3766.3	3621.4	3686.5	0.28	0.1031
Smarcad1	2878.0	2893.4	2748.6	3150.7	3098.7	3046.1	0.10	0.8237
Map3k3	2877.0	2719.4	2626.7	2946.8	2793.8	2980.0	0.09	0.8948
Stk24	2877.0	2841.5	2822.2	2964.9	2992.0	2826.9	0.04	0.9857
Rpl6l	2875.9	2889.8	2823.2	3111.4	3086.4	3076.0	0.09	0.8551
Scaf4	2875.9	2747.7	2420.1	3096.5	2637.7	3086.9	0.09	0.8983
Exoc3	2873.8	3010.0	2852.4	2882.1	2958.1	2881.3	-0.04	0.9810
Mknk1	2872.7	2688.4	2498.7	2598.7	2228.1	2692.0	-0.09	0.8775
Tceal8	2871.6	2857.9	2695.2	2733.5	2869.8	2586.9	-0.05	0.9355
Map3k1	2870.6	2591.9	2964.2	3398.0	2878.0	3804.2	0.30	0.2005
Nolc1	2870.6	2768.6	2195.5	2801.4	2833.8	3245.4	0.11	0.8455
Tcerg1	2869.5	2819.6	2757.7	3254.7	3249.7	3691.0	0.24	0.2967
Bsn	2869.5	2514.4	2546.1	2663.4	2135.7	3037.1	0.03	1.0000
Samm50	2869.5	3012.8	3033.7	3058.3	3148.0	2873.1	0.01	1.0000
Spp1	2868.4	3998.5	5345.1	5560.4	4982.8	8080.4	0.27	0.6287
Hnrnph3	2868.4	2764.0	3105.3	2715.4	2849.2	2927.5	0.02	1.0000
Arhgef9	2867.4	2658.4	2613.6	2667.7	2644.9	2366.8	-0.06	0.9559
Gm6421	2867.4	3246.9	3244.3	2926.7	3111.1	2750.8	-0.12	0.7809
Zc3hav1	2865.2	2786.8	2845.3	3202.7	2792.8	3024.4	0.09	0.8462
Atf7	2863.1	2570.9	2575.3	2822.6	2610.0	3431.1	0.16	0.7056
Abcc9	2859.9	2240.2	2511.8	3065.7	2976.6	3567.8	0.41	0.0154
Fam193a	2858.9	2737.6	2712.3	3282.3	3013.5	3436.5	0.22	0.3625
Zbtb40	2857.8	3074.7	3024.7	2982.9	2890.3	3287.1	0.00	1.0000
Zfp36l1	2856.7	2806.9	3672.5	4696.3	4793.9	6120.3	0.70	0.0000
Eif4enif1	2856.7	2776.8	2942.1	3057.2	2917.0	2959.2	0.08	0.8506
Aldh6a1	2856.7	2597.3	3056.9	2385.3	2681.9	2398.5	-0.08	0.8830
Rab3b	2856.7	2826.9	3133.5	2220.7	2353.3	1983.6	-0.34	0.0699
Sipa1l2	2855.7	2756.8	2742.6	2553.0	2745.5	2982.7	0.00	1.0000
Sephs1	2854.6	2903.4	2916.9	2907.6	2952.9	2979.1	0.02	1.0000
Klk1	2852.5	3938.4	5180.8	5766.3	4009.5	6055.1	0.20	0.7896
Dip2c	2850.3	2762.2	2595.5	3193.1	2916.0	3196.5	0.15	0.6460
Vps52	2850.3	2753.1	2849.4	2900.1	3028.9	2875.8	0.08	0.9078
Eml3	2850.3	2914.4	2742.6	2408.6	2423.1	2534.4	-0.22	0.3444
Csrp1	2848.2	3021.0	3381.3	3191.0	3267.1	3481.8	0.11	0.8053
Nol4l	2848.2	2467.1	2851.4	2416.1	2317.4	2702.8	-0.02	1.0000
Tlk2	2847.1	2959.9	2878.6	3023.3	3030.0	2959.2	0.03	0.9813
Bin1	2847.1	2513.5	2465.5	2498.9	2267.1	2530.7	-0.05	0.9629
Prcc	2847.1	2781.4	3102.3	2562.6	2538.1	2284.4	-0.17	0.5853
Ccser2	2846.1	2366.8	2649.9	2876.8	2738.4	3268.0	0.25	0.2775
Sec24a	2846.1	2249.3	2176.3	2848.1	2255.8	2718.2	0.15	0.7548
Kank1	2845.0	2711.2	2882.6	2718.6	2680.9	2932.9	0.02	1.0000
Asah2	2843.9	2420.6	2387.9	2542.4	2511.4	2630.4	0.06	0.9649
Fam53c	2842.9	2542.7	2778.8	2644.3	2574.1	2658.4	0.03	1.0000
Mical3	2841.8	2393.3	2327.4	2696.3	2641.8	3124.9	0.19	0.6003
D430042O09R	2840.7	2753.1	2297.2	2449.0	2268.1	2538.0	-0.15	0.7177
Zfp398	2839.7	2418.8	2175.3	2843.9	2347.2	3064.2	0.15	0.7648
Rundc1	2839.7	2458.9	2377.8	2486.1	2725.0	2214.6	-0.01	1.0000
Med14	2838.6	2845.1	2424.2	3152.8	2822.6	3437.4	0.14	0.7731
Nlk	2838.6	2485.3	2554.1	2565.7	2620.3	2370.4	0.00	1.0000
Fam76a	2836.5	2613.7	2670.0	2722.9	2794.8	2681.1	0.05	0.9400
Pcnp	2836.5	2798.7	2653.9	2761.1	2683.9	2418.4	-0.08	0.8762
Lhfpl4	2835.4	2894.3	2723.4	2850.2	2897.5	2741.8	-0.02	1.0000
Slc30a4	2835.4	2771.3	2788.9	2699.5	2880.1	2570.6	-0.03	0.9820
Ncoa3	2834.3	2680.2	2738.5	2921.4	2557.6	3394.8	0.11	0.8256

Nt5dc3	2833.3	2417.9	2601.5	2526.5	2536.1	2762.6	0.08	0.9152
Hagh	2833.3	2834.2	3005.5	2786.5	2901.6	2010.8	-0.13	0.8394
Hook2	2832.2	2795.9	2789.9	2616.7	2382.1	2346.9	-0.17	0.5557
Tmem50b	2831.1	2802.3	2829.2	2633.7	3040.2	2783.4	0.01	1.0000
Anks6	2831.1	2959.0	2937.0	2411.8	2312.3	2597.8	-0.25	0.2695
Mtus1	2830.1	2766.8	3084.1	3196.3	3364.7	3601.4	0.25	0.2665
Gm6548	2830.1	2586.4	2717.4	2702.7	3461.2	2461.0	0.11	0.8455
Tm7sf3	2829.0	2919.8	3019.6	2705.9	2994.0	2555.2	-0.08	0.8983
Gdap1l1	2829.0	2741.3	2635.8	2389.5	2615.1	2247.2	-0.16	0.6287
Evi5	2827.9	2756.8	2667.0	2587.0	2747.6	2932.9	0.00	1.0000
Zfp362	2825.8	2960.8	3186.9	2848.1	2885.2	3165.7	0.00	1.0000
Lrch3	2824.8	2704.8	2643.8	3008.4	2608.0	3307.0	0.12	0.8002
Pi4k2b	2824.8	2690.3	2809.1	2668.7	2821.5	2165.7	-0.08	0.9318
Nagk	2824.8	2654.7	3126.4	2441.5	2797.9	2248.1	-0.10	0.8445
Aco1	2823.7	2723.1	2722.4	2874.7	3109.0	3134.9	0.14	0.6769
Fzd3	2823.7	2598.2	2519.9	2694.2	2725.0	2845.0	0.07	0.9340
Dlat	2823.7	2732.2	2901.8	2909.7	2866.7	2411.2	-0.01	1.0000
Chchd3	2823.7	3147.6	2912.8	2798.2	3225.0	2517.1	-0.11	0.8343
Fgf12	2823.7	3083.8	2491.7	2567.9	2564.8	2631.3	-0.20	0.4750
Bckdk	2822.6	2859.7	2887.6	2868.3	2863.6	2899.4	0.01	1.0000
Aida	2822.6	2755.9	2683.1	2645.4	2692.2	2672.9	-0.04	0.9551
Slc35a3	2821.6	2658.4	2489.7	2818.4	2533.0	2554.3	-0.01	1.0000
Coa3	2821.6	2899.8	2510.8	2427.7	2906.7	2376.7	-0.13	0.7648
Klf10	2820.5	2509.9	2653.9	2853.4	2456.0	2109.5	-0.04	0.9653
Rbm3-ps	2820.5	3260.6	3225.2	3187.8	3009.4	3112.2	-0.05	0.9649
Rbsn	2819.4	2677.5	2585.4	2678.3	2637.7	2672.0	-0.01	1.0000
Ppil2	2818.4	3152.1	2841.3	2739.8	2727.1	2703.7	-0.18	0.4951
Psmd11	2817.3	2826.0	2675.1	2883.1	2691.1	2751.7	-0.02	0.9881
Dynlrb1	2817.3	2847.0	3161.7	2719.7	3109.0	2679.3	-0.02	0.9966
Stk16	2817.3	2759.5	2521.9	2524.3	2643.9	2110.5	-0.15	0.7074
Ift80	2817.3	2917.1	2751.6	2503.1	2625.4	2552.5	-0.16	0.5657
Fnta	2816.2	2448.8	2795.0	2627.3	2649.0	2479.1	0.05	0.9731
Ilkap	2814.1	2824.2	2732.5	2617.8	2714.7	2693.8	-0.07	0.9326
Usp32	2813.0	2604.6	2879.6	2645.4	2727.1	2899.4	0.06	0.9490
Pxn	2812.0	2815.1	2920.9	2855.5	2631.6	3183.8	0.03	0.9966
Rps6kb1	2812.0	2875.2	2724.4	2920.3	2674.7	2968.2	0.00	1.0000
Letm1	2809.8	2512.6	2240.8	2836.4	2734.3	2865.0	0.14	0.7682
Slc27a4	2807.7	2575.5	2797.0	2554.1	2397.5	2283.5	-0.10	0.8198
Hnf4a	2807.7	2399.6	2402.0	2114.6	2207.5	2163.9	-0.15	0.6860
Gsr	2806.6	2877.9	3018.6	2951.1	2921.1	2693.8	-0.01	0.9995
Ak1	2806.6	2822.4	3015.6	2561.5	2899.6	2027.1	-0.16	0.7648
Bhlhb9	2805.6	2729.4	2884.6	2721.8	2878.0	2697.4	0.01	1.0000
Rhot1	2805.6	2688.4	2693.2	2683.6	2758.9	2661.2	0.00	1.0000
Nin	2804.5	2482.5	2366.7	2697.4	2480.6	3170.2	0.13	0.8198
Pik3r4	2804.5	2996.4	2563.2	3206.9	3026.9	2967.3	0.05	0.9681
Ufsp2	2804.5	2757.7	2662.0	2769.6	2931.4	2547.9	0.00	1.0000
Rasd1	2802.4	1997.9	2722.4	3925.6	3680.9	2705.5	0.55	0.0016
Rnf103	2802.4	3042.8	3054.9	3090.1	3297.9	2884.9	0.03	0.9783
Postn	2801.3	2683.0	3724.9	4445.7	4367.8	6106.7	0.66	0.0002
Pikfyve	2801.3	2438.8	2453.4	2868.3	2667.5	3231.8	0.21	0.5060
Col13a1	2801.3	2421.5	2321.4	2346.0	2893.4	2752.6	0.10	0.8802
Strip1	2799.2	2580.0	2461.5	2696.3	2575.1	2519.9	0.00	1.0000
Sfxn1	2798.1	2617.4	2483.6	2975.5	2677.8	2620.4	0.07	0.9452
Pabpc4	2798.1	2907.1	2644.8	2933.0	2870.8	2989.1	0.02	0.9884
Git2	2797.0	2675.7	2817.1	3043.4	2791.7	2948.3	0.11	0.7864
Ryk	2797.0	2804.1	2838.3	2734.5	2880.1	2630.4	-0.03	0.9804
Ric8a	2797.0	2583.7	2676.1	2432.0	2603.9	2196.5	-0.10	0.8575
Cd151	2797.0	3050.1	2943.1	2685.7	2968.4	2653.9	-0.11	0.7830
Creb3	2797.0	2779.5	2807.0	2576.4	2654.2	2329.6	-0.13	0.7648
Gabpa	2793.9	2924.4	2789.9	2658.1	2688.0	2821.5	-0.09	0.8473
Spg11	2792.8	2663.8	2504.8	2982.9	2573.1	3151.2	0.11	0.8462
Plcb4	2792.8	2713.9	2770.8	2786.5	2602.8	2799.7	0.00	1.0000
Cask	2791.7	3228.7	3088.1	3453.2	3078.2	3596.8	0.08	0.9186
Smyd2	2791.7	3067.4	2719.4	2979.7	2848.2	2568.8	-0.10	0.8316
Eml4	2790.7	2813.2	2489.7	2868.3	2859.5	3423.8	0.12	0.7977
Nsun2	2790.7	2844.2	2961.2	2896.9	2917.0	3097.7	0.06	0.9375
Micall1	2790.7	2575.5	2922.9	2616.7	2519.7	2876.7	0.03	0.9850
Ddrgk1	2790.7	2861.5	2890.7	2869.3	3043.3	2555.2	-0.02	1.0000
Svip	2790.7	2700.3	2710.3	2611.4	2859.5	2354.1	-0.05	0.9516
Tmbim1	2789.6	2540.8	2626.7	2373.6	2584.3	2239.1	-0.09	0.8719
Clip3	2788.5	2905.3	3437.8	2764.3	2870.8	2942.9	-0.04	0.9713
Dnaja3	2787.5	2525.4	2490.7	2637.9	2927.3	2428.4	0.06	0.9559
Rgmb	2786.4	1782.9	3042.8	2418.2	2510.4	2329.6	0.30	0.1150
Nup214	2785.3	3055.6	2651.9	3335.4	3225.0	3615.8	0.16	0.6561
Atp5e	2785.3	3141.2	3106.3	2920.3	3176.8	2613.2	-0.09	0.8772
Nudc	2784.3	2984.5	2987.4	2956.4	3096.7	2976.4	0.02	0.9969

Ak3	2783.2	2898.9	2686.1	2778.1	2980.7	2935.6	0.01	1.0000
Pcnt	2782.1	2734.0	2400.0	3165.5	2535.1	3285.2	0.13	0.8010
Kcnq1ot1	2782.1	2829.6	2297.2	2841.7	1909.8	4130.3	0.07	0.9792
Slc25a17	2782.1	2732.2	3084.1	2822.6	3004.3	2535.3	0.01	1.0000
Ppp4c	2782.1	2919.8	3205.0	2687.8	2970.4	2400.3	-0.11	0.8314
Etfa	2780.0	2629.2	2939.0	2808.8	2660.3	2393.1	-0.02	0.9884
Tle2	2780.0	2714.9	2624.7	2334.3	2322.5	2295.2	-0.21	0.3767
Cux2	2778.9	2484.4	2688.2	2637.9	2426.2	2950.1	0.07	0.9334
Ppp2cb	2778.9	2878.8	3149.6	2722.9	2805.1	2639.4	-0.08	0.8826
Gm2000	2776.8	3411.8	3583.9	3352.3	3382.1	3296.1	-0.01	1.0000
Elof1	2776.8	2980.0	2979.3	2501.0	2834.9	2082.4	-0.22	0.4843
Ints6	2775.7	2773.2	2641.8	3218.6	2816.4	2784.3	0.08	0.9110
Metap1	2775.7	2745.8	2765.7	2758.9	2625.4	2620.4	-0.04	0.9576
Map2k7	2774.7	2771.3	2483.6	2551.9	2618.2	2502.7	-0.09	0.8632
Myt1l	2774.7	2703.9	2231.7	2593.3	2486.8	2339.6	-0.09	0.8868
Eps8	2772.5	2697.5	2724.4	2834.3	2771.2	2884.9	0.06	0.9495
Rer1	2772.5	2897.1	2812.1	2805.7	3003.3	2585.1	-0.04	0.9846
Zfp9	2770.4	2563.6	2060.4	2643.2	2479.6	2560.6	0.01	1.0000
Ubr7	2769.3	2728.5	2688.2	2648.5	2830.8	2836.9	0.02	1.0000
Wdr45b	2769.3	2809.6	2813.1	2924.5	2935.5	2509.0	-0.01	1.0000
Dop1a	2768.3	2618.3	2320.4	2968.1	2534.0	3268.9	0.14	0.7535
Cpne3	2766.1	2435.2	2826.2	2934.1	2891.3	2881.3	0.21	0.3479
Arf2	2766.1	2790.5	2829.2	2719.7	2779.4	2715.5	-0.03	0.9810
March5	2766.1	2675.7	2584.4	2560.4	2658.3	2499.9	-0.05	0.9587
Mvp	2766.1	2658.4	2433.2	2404.4	2571.0	2511.7	-0.08	0.9232
Spg20	2765.1	2879.7	2684.1	2868.3	2888.3	2842.3	0.00	1.0000
Gm6733	2765.1	3056.5	2504.8	3043.4	3086.4	2832.4	0.00	1.0000
Slc39a3	2763.0	2941.7	2920.9	2580.6	2662.4	2344.1	-0.19	0.5377
Nsmaf	2761.9	2805.0	2844.3	2967.0	2629.5	2875.8	0.01	1.0000
Paxbp1	2761.9	2570.0	2508.8	2694.2	2161.3	2807.0	-0.01	1.0000
Arpc5l	2761.9	2749.5	2623.7	2689.9	2820.5	2512.6	-0.03	0.9980
Bcl9l	2759.8	2814.2	3018.6	3430.9	3145.0	4050.6	0.28	0.2368
Th	2759.8	2310.4	2539.0	2457.5	2420.1	2203.7	0.00	1.0000
Mthfr	2759.8	2724.0	2412.1	2337.5	2325.6	2466.4	-0.17	0.6104
Atg4b	2758.7	2847.0	2866.5	2616.7	2685.0	2596.9	-0.10	0.8426
Vps37c	2755.5	2638.3	2492.7	2492.5	2480.6	2490.9	-0.08	0.8911
Gadd45g	2754.4	2138.2	2260.9	3882.1	3661.4	2558.8	0.47	0.0216
Esam	2754.4	2684.8	2952.1	3304.6	3354.4	3316.0	0.27	0.1413
Krcc1	2753.4	2734.9	2718.4	2567.9	2958.1	2357.7	-0.05	0.9515
Ccng1	2752.3	3641.4	3017.6	3087.0	3003.3	2759.9	-0.23	0.3136
Slc25a11	2751.2	2999.1	2994.4	2751.5	3011.5	2528.0	-0.10	0.8543
Abraxas2	2750.2	2093.5	2133.0	2222.9	2360.5	2151.2	0.05	0.9838
Rhobtb3	2749.1	2984.5	3001.5	3368.3	3198.3	3518.0	0.16	0.6072
Tubgcp3	2748.0	3092.9	2538.0	2938.3	2600.8	2627.6	-0.13	0.7539
4833420G17R	2747.0	2430.6	2480.6	2557.3	2507.3	2570.6	0.05	0.9513
Vps33a	2745.9	2890.7	2911.8	2992.5	3015.6	2973.7	0.05	0.9471
Reps1	2744.8	2842.4	2686.1	2973.4	2644.9	2975.5	0.02	0.9898
Myrf	2743.8	2580.0	2824.2	2502.1	2456.0	3086.0	0.03	0.9986
Rbx1	2743.8	2895.2	2815.1	2590.2	2800.0	2540.7	-0.11	0.7829
Cebpg	2742.7	2196.5	2285.1	2606.1	2752.7	2817.0	0.25	0.3097
Lyst	2742.7	2530.8	2198.5	2785.5	2376.9	3094.1	0.11	0.8520
Dbn1	2742.7	2926.2	3218.1	2506.3	2846.2	2669.3	-0.12	0.7873
Pitrm1	2741.6	2908.0	2691.2	3258.9	3159.3	3140.3	0.13	0.7346
Itgb5	2739.5	2563.6	2795.0	2373.6	2839.0	2616.8	0.01	1.0000
Stk4	2739.5	2550.9	2668.0	2580.6	2596.7	2544.3	0.00	1.0000
Pts	2739.5	2677.5	2577.3	2475.5	2376.9	2138.5	-0.18	0.5657
Nfib	2738.4	2664.7	3460.9	4737.7	4270.3	6487.1	0.72	0.0000
Cntn1	2738.4	2275.7	2638.8	2654.9	3153.2	2871.3	0.27	0.2005
Dcp2	2735.2	2658.4	2683.1	2995.7	2663.4	3345.0	0.15	0.7009
Fgd6	2734.2	2672.0	2443.3	2997.8	2669.6	3036.2	0.11	0.8043
Rbm10	2734.2	3062.0	2600.5	2408.6	2323.5	2245.4	-0.34	0.0370
Rfx7	2731.0	2677.5	2563.2	2908.6	2609.0	3019.8	0.08	0.8863
Ubqln4	2731.0	2530.8	2354.7	2369.4	2384.1	2477.3	-0.06	0.9515
Mapkbp1	2731.0	2700.3	2460.4	2417.1	2226.0	2614.1	-0.13	0.7535
Dync1li1	2729.9	2710.3	2736.5	2932.0	2848.2	2707.4	0.05	0.9581
Isyna1	2729.9	3040.1	2778.8	2850.2	2816.4	2500.8	-0.12	0.7628
Mfap3	2728.9	2577.3	2565.2	2939.4	2910.9	3159.3	0.20	0.4836
Vsig10	2728.9	2595.5	2989.4	2676.1	2699.3	2465.5	-0.01	1.0000
Slc4a3	2728.9	2863.4	2562.2	2700.6	2559.7	2499.9	-0.12	0.7619
Atp6v0e	2727.8	2724.9	2910.8	2651.7	2544.3	2028.9	-0.16	0.7185
Ezh1	2726.7	2761.3	2549.1	2630.5	2555.6	2657.5	-0.06	0.9134
Znrf1	2723.5	2769.5	2784.9	2985.1	2740.4	2925.6	0.05	0.9334
Zadh2	2723.5	2699.4	2713.3	2582.7	2662.4	2639.4	-0.04	0.9646
Runx1t1	2722.5	2893.4	3068.0	3239.8	3010.4	3256.3	0.12	0.7811
Cdc123	2722.5	2959.0	3050.9	2808.8	2976.6	2736.3	-0.05	0.9731
Hectd3	2722.5	2579.1	2681.1	2447.9	2476.5	2358.6	-0.09	0.8473

Kdm4c	2720.3	2816.9	2637.8	2744.1	2788.7	2756.3	-0.02	1.0000
Gm13835	2720.3	2877.9	2728.5	2766.4	2871.8	2644.9	-0.05	0.9515
Snx19	2718.2	2663.8	2730.5	2824.8	3007.4	3113.1	0.14	0.7061
Bicd1	2717.1	2656.5	2364.7	2924.5	2631.6	2903.9	0.09	0.8970
Lmbrd1	2716.1	2636.5	2573.3	2800.3	2781.5	2649.4	0.05	0.9365
Smc5	2713.9	2766.8	2282.1	2986.1	2445.7	3095.0	0.06	0.9487
Suox	2713.9	2387.8	2523.9	2257.9	2776.3	2067.0	-0.04	0.9933
Dennd4a	2712.9	2388.7	2517.9	2835.4	2834.9	3137.6	0.25	0.2518
Polg	2712.9	2707.6	2487.6	2837.5	2590.5	2710.1	0.01	1.0000
Hdgfl2	2712.9	2867.9	2587.4	2791.9	2764.0	2872.2	-0.01	1.0000
H2afx	2712.9	2765.9	2300.2	2250.5	2372.8	2684.7	-0.14	0.7806
Epg5	2711.8	2510.8	2145.1	2755.8	2660.3	2973.7	0.14	0.7420
Ccna2	2711.8	2532.6	1579.8	2200.6	2413.9	3000.8	0.07	0.9557
Emsy	2710.7	2488.0	2568.3	2620.9	2424.2	2868.6	0.06	0.9521
Asb1	2709.7	2683.9	2425.2	2434.1	2484.7	2578.7	-0.08	0.8706
Brpf1	2708.6	2483.5	2412.1	2963.8	2595.6	2692.9	0.12	0.7648
Zfp275	2708.6	2550.0	2450.4	2871.5	2511.4	2858.6	0.09	0.8510
B2m	2708.6	2920.7	2571.3	2546.6	3635.7	2363.2	0.00	1.0000
Prpf40b	2708.6	2688.4	2640.8	2685.7	2553.5	2509.0	-0.05	0.9559
Ccdc8	2708.6	2414.2	2295.2	2220.7	2259.9	2316.1	-0.09	0.9003
Plekhn1	2708.6	2605.5	2381.9	2331.1	2166.5	2388.5	-0.16	0.6871
Arhgef3	2707.5	2439.7	2770.8	2673.0	2988.9	2686.5	0.15	0.6626
Irs2	2707.5	2529.0	2934.0	2884.2	2652.1	2750.8	0.09	0.8353
Cpsf3	2707.5	2813.2	2614.6	2867.2	2995.0	3013.5	0.08	0.8830
Ift57	2707.5	2603.7	2292.2	2490.4	2867.7	2290.7	-0.02	1.0000
Lrrfip2	2705.4	2548.1	2122.9	2723.9	2883.1	2528.0	0.09	0.9117
Golga7	2705.4	2617.4	2607.5	2480.8	2701.4	2387.6	-0.05	0.9471
Pcbd1	2704.3	2768.6	2813.1	2823.7	2753.8	2518.0	-0.03	0.9748
Banp	2704.3	2631.0	2725.4	2252.6	2423.1	2060.6	-0.21	0.4350
Txn1	2702.2	3000.0	3003.5	2822.6	3066.9	2845.0	-0.03	0.9818
Insig1	2701.1	3465.5	2885.6	3677.2	3660.4	3503.5	0.09	0.8366
Yeats2	2701.1	3069.2	2742.6	3074.2	2743.5	3203.7	0.00	1.0000
Ap2m1	2701.1	2533.6	2513.8	2374.7	2591.5	2274.4	-0.07	0.9096
Enc1	2700.1	2587.3	2804.0	3219.7	2924.2	3603.2	0.28	0.2054
Zfp950	2700.1	2589.1	2060.4	2635.8	2230.1	2845.0	0.01	1.0000
Fam216a	2700.1	2536.3	2602.5	2179.3	2453.9	1912.1	-0.20	0.5713
B830017H08R	2698.0	2816.9	3044.8	2855.5	3070.0	2801.6	0.04	0.9850
Lrrfip1	2696.9	2588.2	2876.6	2841.7	2881.1	3142.1	0.16	0.6240
Eftud2	2695.8	3082.0	2875.6	3458.5	3359.5	3630.3	0.18	0.5490
Zfp644	2695.8	2592.8	2642.8	2945.8	2672.6	3124.0	0.15	0.6871
Rad54l2	2695.8	2566.4	2451.4	2607.1	2612.1	3050.6	0.09	0.8714
Trp53i13	2695.8	2783.2	3049.9	2352.4	2577.2	2205.6	-0.21	0.4654
Ppp2r1b	2694.8	2652.9	2293.2	2975.5	2988.9	2984.5	0.16	0.6563
Khnyn	2694.8	2761.3	2793.9	2599.7	2671.6	2706.5	-0.05	0.9417
Nup188	2693.7	2734.9	2399.0	2581.7	2425.2	2777.1	-0.05	0.9500
Sugp1	2693.7	2827.8	2553.1	2592.3	2541.2	2903.9	-0.05	0.9559
Pabpn1	2693.7	2577.3	2672.0	2454.3	2341.0	2346.9	-0.11	0.7776
Pdgfb	2691.6	2297.6	2567.2	2825.8	2812.3	2899.4	0.26	0.1798
Atp7a	2691.6	2899.8	2693.2	2894.8	2471.4	2925.6	-0.05	0.9812
Agbl5	2689.4	2754.9	2515.9	2468.1	2320.5	2430.2	-0.17	0.5813
Mef2c	2688.4	2664.7	2573.3	3782.3	3364.7	4139.4	0.44	0.0052
Rnf41	2688.4	2379.6	2561.2	2536.0	2711.7	2355.9	0.06	0.9237
Csf2ra	2688.4	2375.0	2363.7	2304.6	2156.2	2167.5	-0.11	0.8221
Sil1	2687.3	2824.2	2896.7	2643.2	2938.6	2567.0	-0.05	0.9748
Srp14	2687.3	3037.4	2909.8	2468.1	3025.8	2272.6	-0.18	0.6729
C130074G19R	2686.2	2604.6	3247.3	3181.4	3554.6	4281.6	0.38	0.0681
Sema7a	2686.2	2228.4	2679.1	2774.9	3199.4	2969.1	0.34	0.0513
Trim27	2686.2	2711.2	2387.9	2463.8	2582.3	2476.4	-0.09	0.8884
Klhl21	2682.0	2201.9	2378.8	2439.4	2372.8	2347.8	0.08	0.8934
Trp53bp2	2679.8	2548.1	2828.2	2720.7	2801.0	2915.7	0.12	0.7764
Asap2	2678.8	1892.2	2575.3	1860.9	1689.0	1874.0	-0.13	0.7896
Yars	2677.7	2711.2	2504.8	2643.2	2671.6	2759.0	0.00	1.0000
Mknk2	2675.6	2734.0	2808.0	2819.5	3026.9	3031.6	0.10	0.8099
Usf3	2675.6	2466.1	2133.0	2703.7	2252.7	3040.7	0.10	0.8710
Bbs2	2674.5	2567.3	2612.6	2348.1	2475.5	2287.1	-0.11	0.7852
Zcrb1	2674.5	2808.7	2816.1	2589.1	2616.2	2285.3	-0.15	0.6836
Uqcc2	2674.5	3020.0	2793.9	2709.1	2823.6	2316.1	-0.16	0.6618
Kcnn3	2673.4	2076.2	1663.5	2267.5	2480.6	2213.7	0.11	0.8516
Steap2	2673.4	2406.0	2171.3	2524.3	2536.1	2428.4	0.05	0.9844
Phf20	2672.4	2875.2	2665.0	2732.4	2945.8	2974.6	0.02	1.0000
Gpr108	2672.4	3235.0	2825.2	2494.6	2753.8	2390.3	-0.28	0.1843
Srp9	2671.3	2955.4	2446.3	2443.7	2475.5	2303.4	-0.24	0.2920
Naa35	2670.2	2369.6	2542.1	2543.5	2516.6	2421.1	0.05	0.9616
Camkk2	2669.2	2578.2	2518.9	2117.8	2234.2	2304.3	-0.20	0.4507
Dnajc8	2668.1	2619.2	2615.6	2634.7	2692.2	2595.0	0.01	1.0000
Zbtb14	2667.1	2712.1	2985.4	2835.4	3295.9	2876.7	0.12	0.7830

Aldh2	2666.0	2452.5	2964.2	2841.7	3088.5	3298.8	0.26	0.2355
Wtap	2666.0	2396.0	2633.7	2737.7	2590.5	2580.5	0.11	0.8175
Rbl2	2664.9	2692.1	2665.0	2617.8	2727.1	2733.6	0.00	1.0000
Nr2f6	2663.9	2697.5	2735.5	2245.2	2512.5	2073.3	-0.22	0.4324
Copg2	2662.8	2763.1	2708.3	2902.3	2987.9	2961.9	0.09	0.8669
Med1	2661.7	2434.3	2501.8	2822.6	2669.6	3096.8	0.20	0.5060
Ndufv2	2659.6	2842.4	2834.2	2782.3	2859.5	2355.0	-0.07	0.9114
Gpx1	2658.5	2930.8	2806.0	2592.3	2705.5	2362.3	-0.17	0.6128
Cers6	2657.5	2359.6	2045.3	2618.8	2344.1	2728.2	0.10	0.8868
Ss18	2657.5	2483.5	2572.3	2557.3	2609.0	2596.9	0.05	0.9679
Epc2	2657.5	2805.0	2759.7	2645.4	2810.2	2816.0	-0.02	1.0000
Nhlrc2	2657.5	2695.7	2457.4	2695.3	2338.9	2641.2	-0.06	0.9649
Eif2a	2657.5	2803.2	2658.9	2680.4	2769.2	2493.6	-0.07	0.9126
Clk2	2657.5	2870.6	2558.2	2820.5	2591.5	2599.6	-0.08	0.9154
Thbs3	2657.5	2755.9	2636.8	2253.7	2392.3	2326.9	-0.22	0.3353
Ehd2	2656.4	2756.8	2954.1	3839.6	3730.2	4896.6	0.50	0.0028
Ptar1	2656.4	2434.3	2670.0	2998.9	2609.0	3365.9	0.24	0.3699
Rxra	2656.4	2741.3	3010.6	2801.4	2902.6	3360.4	0.12	0.7992
Skap1	2655.3	2656.5	2600.5	2743.0	2949.9	2314.3	0.01	1.0000
Glrx5	2655.3	2460.7	2837.3	2377.9	2798.9	2158.5	-0.04	1.0000
Sgsm3	2655.3	2637.4	2535.0	2469.1	2437.5	2253.6	-0.13	0.7405
Crebrf	2654.3	2631.0	2407.0	2371.5	2564.8	2064.3	-0.14	0.7406
Ndufv3	2652.1	2718.5	2787.9	2460.7	2675.7	2729.1	-0.05	0.9487
Apba1	2651.1	2134.5	2948.1	2688.9	2654.2	2922.9	0.27	0.1798
Tfip11	2651.1	2440.6	2569.3	2607.1	2555.6	2370.4	0.02	0.9877
Scn3a	2651.1	2542.7	2202.5	2745.1	2314.3	2448.3	-0.01	1.0000
Dock10	2650.0	2513.5	2590.4	2624.1	2619.3	2666.6	0.06	0.9300
Chrnb2	2650.0	2547.2	2611.6	2372.5	2453.9	2408.5	-0.08	0.9108
Adcy9	2648.9	2460.7	2191.4	2607.1	2473.5	2825.1	0.09	0.8717
Armcx3	2648.9	2572.7	2397.0	2501.0	2127.4	2119.5	-0.17	0.6491
Sh2b3	2647.9	2630.1	2777.8	2938.3	2965.3	3038.0	0.16	0.6204
Paf1	2647.9	2508.1	2668.0	2645.4	2759.9	2659.4	0.08	0.8990
Tdrd7	2647.9	2280.3	2408.1	2130.5	2276.3	2170.2	-0.07	0.9096
Cpox	2647.9	2368.7	2202.5	2169.8	2034.0	2201.9	-0.14	0.7470
Snx6	2646.8	2529.9	2837.3	2643.2	2680.9	2535.3	0.03	0.9731
Ift172	2646.8	2623.8	2391.9	2459.6	2404.7	2833.3	-0.02	0.9923
Mmadhc	2646.8	2609.2	2752.6	2444.7	2793.8	2229.1	-0.07	0.9457
Taz	2645.7	2662.0	2525.9	2437.3	2463.2	2210.1	-0.15	0.6819
Arf6	2644.7	2525.4	2636.8	3087.0	2588.5	2073.3	0.01	1.0000
Flywch1	2644.7	2552.7	2754.6	2334.3	2584.3	2579.6	-0.04	0.9679
Fam114a2	2644.7	2666.6	2833.2	2732.4	2516.6	2342.3	-0.07	0.9343
Laptm4b	2641.5	2432.4	2792.9	2471.3	2615.1	2625.8	0.05	0.9649
Ehd1	2640.4	2498.9	2514.9	2301.4	2680.9	2575.1	0.00	1.0000
Vamp4	2640.4	2576.4	2651.9	2357.7	2609.0	2106.8	-0.12	0.7920
Atp6v1f	2640.4	2779.5	2919.9	2413.9	3002.2	1920.2	-0.15	0.7780
Rrm1	2639.3	3343.5	2649.9	3503.1	3379.1	4090.5	0.16	0.6258
Rnf2	2639.3	2503.5	2681.1	2748.3	2730.1	2796.1	0.12	0.7608
Zfp142	2639.3	2190.1	2002.0	2335.4	2123.3	2618.6	0.07	0.9232
Prelid1	2639.3	2723.1	2827.2	2830.1	2943.7	2778.0	0.06	0.9216
Dusp5	2638.3	2524.5	2615.6	2456.4	2127.4	1978.2	-0.19	0.5798
Arhgap31	2637.2	2253.0	2310.3	3470.2	3082.3	3881.2	0.52	0.0009
Smarcb1	2637.2	2721.2	2666.0	2917.1	2557.6	2715.5	0.01	1.0000
Lrp3	2637.2	2421.5	2514.9	2043.5	2094.6	1902.1	-0.25	0.2359
Bcl7a	2636.2	2629.2	2579.3	2609.3	2419.0	2637.6	-0.04	0.9916
Trim24	2635.1	2304.9	2450.4	2645.4	2614.1	2806.1	0.18	0.5495
Mtrex	2634.0	2591.9	2473.5	2918.2	2780.5	2657.5	0.10	0.8558
Herc3	2634.0	2520.8	2686.1	2712.2	2529.9	2610.4	0.04	0.9557
Cstf2	2633.0	2662.9	2474.5	2686.8	2494.0	2632.2	-0.02	0.9877
Mrpl24	2633.0	2589.1	2578.3	2399.1	2506.3	2441.1	-0.07	0.8890
Zfp280d	2630.8	2560.0	2490.7	2825.8	2692.2	2885.8	0.12	0.7910
Dnajb12	2630.8	2910.7	2697.2	2750.5	2836.9	2727.3	-0.05	0.9417
Cdk5r2	2630.8	2283.9	2483.6	2312.0	2332.8	2044.3	-0.05	0.9586
Rars	2629.8	2713.0	2471.5	2624.1	2532.0	2505.4	-0.07	0.9343
Pcsk4	2629.8	2495.3	2279.1	1739.9	1982.7	1782.6	-0.39	0.0254
Ncapd3	2628.7	2801.4	2295.2	2946.8	2513.5	2976.4	0.03	0.9797
Gnl2	2628.7	2657.5	2530.0	2794.0	2610.0	2638.5	0.02	0.9896
Gpr45	2628.7	2551.8	2552.1	2359.8	2610.0	1975.5	-0.13	0.8000
Dgke	2627.6	2056.2	2305.3	2467.0	2458.1	2519.9	0.21	0.4537
Dnajc21	2627.6	2693.9	2603.5	2696.3	2657.2	2528.9	-0.03	0.9789
Zfp384	2626.6	2756.8	2469.5	2896.9	2486.8	2881.3	0.02	0.9966
Rcan2	2625.5	2431.5	2711.3	2919.2	2839.0	2992.7	0.22	0.2980
Akt1s1	2625.5	2779.5	2666.0	2428.8	2639.8	2343.2	-0.14	0.6836
Snx18	2624.4	2559.1	2614.6	3130.5	2951.9	2956.4	0.21	0.3485
Ttc37	2624.4	2552.7	2357.7	2787.6	2584.3	2847.8	0.10	0.8370
Rtf1	2623.4	2465.2	2638.8	2725.0	2682.9	2688.3	0.11	0.7873
Btbd9	2621.2	2767.7	2813.1	2712.2	2584.3	2938.3	-0.01	1.0000

Nsmf	2621.2	2708.5	2918.9	2590.2	2689.1	2360.4	-0.08	0.8759
Tsc22d4	2621.2	2683.0	2754.6	2467.0	2447.8	2368.6	-0.13	0.7414
Tcn2	2620.2	2357.7	2651.9	2639.0	2896.5	2697.4	0.18	0.5261
Apeh	2620.2	2654.7	2783.9	2320.5	2737.3	2222.8	-0.12	0.8281
Slc12a6	2619.1	2549.0	2632.7	2556.2	2582.3	2726.4	0.03	0.9972
Vcl	2618.0	2394.2	2573.3	3299.3	3247.6	4181.0	0.48	0.0046
Idh3a	2618.0	2908.0	2689.2	3082.7	2785.6	2756.3	0.00	1.0000
Fam98a	2617.0	2624.7	2337.5	2723.9	2619.3	2832.4	0.06	0.9278
Ppp4r1	2617.0	2627.4	2729.5	2798.2	2640.8	2602.3	0.02	0.9884
Ap2s1	2617.0	2638.3	2574.3	2679.3	2817.4	2342.3	-0.01	1.0000
Bhlhe41	2617.0	2480.7	2494.7	2467.0	2439.6	2322.4	-0.04	0.9515
Kdm3a	2615.9	2865.2	2376.8	2551.9	2332.8	2473.7	-0.18	0.5571
Med12l	2614.8	2596.4	2478.6	2941.5	2458.1	2860.4	0.08	0.8911
Pip4k2b	2612.7	2273.0	2399.0	2405.5	2455.0	2522.6	0.09	0.8569
Pou2f1	2611.6	2117.2	2253.9	2515.9	2342.0	3017.1	0.23	0.4507
Fis1	2611.6	2616.5	2765.7	2643.2	2926.3	2476.4	0.03	0.9815
Tlnrd1	2611.6	2593.7	2866.5	2671.9	2801.0	2452.8	0.01	0.9979
Pmpcb	2611.6	2570.0	2409.1	2312.0	2483.7	2122.2	-0.13	0.7406
Nr1h2	2610.6	2616.5	2379.8	2305.7	2405.7	2230.0	-0.15	0.6592
Srsf9	2608.4	2605.5	2846.3	2665.5	2751.7	2385.8	-0.01	1.0000
Pcyt1a	2608.4	2752.2	2505.8	2647.5	2565.9	2739.1	-0.04	0.9895
Sec23a	2607.4	2519.0	2580.3	2745.1	2616.2	2692.0	0.08	0.8728
Vma21	2607.4	2638.3	2478.6	2592.3	2613.1	2587.8	-0.01	1.0000
Serpine2	2606.3	2862.4	3355.2	3287.6	3202.5	3332.3	0.16	0.7161
Topbp1	2605.3	3302.5	2545.1	3642.1	3176.8	3838.7	0.15	0.7075
Slc27a1	2605.3	2370.5	2506.8	2315.2	2323.5	2165.7	-0.07	0.9304
Tpd52l2	2605.3	2701.2	2681.1	2234.5	2553.5	2244.5	-0.18	0.5588
Adprh	2604.2	2604.6	2630.7	2553.0	2915.0	2475.5	0.02	0.9944
Gtf2a1	2603.1	2568.2	2771.8	2786.5	2990.9	2878.5	0.14	0.7048
Ypel5	2602.1	2566.4	2781.9	2559.4	2870.8	2337.8	0.00	1.0000
Heatr5a	2601.0	2559.1	2208.6	2732.4	2455.0	3091.4	0.11	0.8591
Crkl	2601.0	2802.3	2519.9	2965.9	2867.7	3036.2	0.09	0.8762
Ube2g1	2601.0	2421.5	2652.9	2481.9	2599.7	2294.3	0.00	1.0000
Dus3l	2601.0	2822.4	2767.7	2388.5	2403.6	2214.6	-0.24	0.2989
Scyl2	2599.9	2495.3	2431.2	2668.7	2650.1	2628.6	0.08	0.9126
Tbc1d9	2599.9	2546.3	2290.2	2728.2	2680.9	2590.5	0.07	0.9158
Mbtd1	2599.9	2299.4	2338.5	2356.6	2337.9	2481.8	0.04	0.9627
A330076H08R	2599.9	1921.4	1752.1	1824.8	1570.9	2139.4	-0.09	0.9162
Cacybp	2599.9	2592.8	2546.1	2328.0	2386.2	2095.1	-0.17	0.6120
Plxna1	2598.9	2163.7	2238.8	2666.6	2519.7	3107.7	0.28	0.2688
Emcn	2598.9	2869.7	2728.5	3107.1	3228.1	3244.5	0.15	0.6081
Fbxl14	2598.9	2494.4	2428.2	2540.3	2657.2	2275.3	0.00	1.0000
Fam76b	2597.8	2769.5	2500.7	2998.9	2703.4	2905.7	0.06	0.9178
Dlg3	2597.8	2556.3	2304.3	2432.0	2610.0	2297.0	-0.05	0.9789
Ikbkg	2597.8	2376.0	2278.1	2190.0	2332.8	2203.7	-0.08	0.8779
Sntb2	2596.7	2271.2	2419.1	2858.7	2572.0	3139.4	0.27	0.2545
Foxj2	2595.7	2386.0	2371.8	2526.5	2344.1	2489.1	0.03	0.9748
Rala	2594.6	2512.6	2587.4	2352.4	2436.5	2291.6	-0.09	0.8558
Atad2b	2593.5	2395.1	2498.7	2728.2	2537.1	3096.8	0.18	0.5890
Flot1	2593.5	2295.8	2620.6	2106.1	2467.3	1954.7	-0.10	0.8558
Tapt1	2592.5	2632.0	2594.4	2874.7	2866.7	2701.0	0.09	0.8748
Ndufb8	2592.5	2928.9	2787.9	2606.1	2933.4	2149.4	-0.14	0.7830
Plod3	2591.4	2509.9	2421.1	2684.6	2846.2	2517.1	0.09	0.8665
Lrrc10b	2591.4	2611.9	2453.4	2714.4	3083.3	2221.0	0.04	0.9703
Def8	2590.3	2681.1	2566.2	2775.9	2744.5	2585.1	0.02	0.9896
Zfyve1	2590.3	2763.1	3016.6	2689.9	2838.0	2376.7	-0.06	0.9269
Ric1	2589.3	2387.8	2397.0	2738.8	2613.1	2944.7	0.18	0.5618
Tmod1	2589.3	1945.9	2053.4	2316.3	2587.4	1786.2	0.10	0.8745
Plcxd2	2587.1	2400.6	2173.3	2921.4	2626.4	2478.2	0.14	0.7749
Dap3	2587.1	2721.2	2904.8	2614.6	2581.3	2540.7	-0.07	0.9318
Ddx47	2586.1	2656.5	2595.5	2595.5	2677.8	2501.7	-0.03	1.0000
Gorasp1	2586.1	2415.1	2232.7	2193.1	2189.0	2101.4	-0.14	0.7043
Vps13a	2585.0	2419.7	2044.3	3091.2	2544.3	3354.1	0.27	0.3532
Trappc6b	2585.0	2450.7	2818.1	2373.6	2537.1	2028.0	-0.10	0.8558
Ago3	2583.9	2243.9	1787.4	2405.5	1865.6	2829.6	0.06	0.9672
March9	2583.9	2370.5	2569.3	2482.9	2286.6	2039.8	-0.07	0.9040
Lss	2581.8	2789.6	2373.8	2716.5	2259.9	2755.4	-0.07	0.9117
Slu7	2581.8	2717.6	2603.5	2434.1	2629.5	2373.1	-0.11	0.7873
Unc50	2581.8	2576.4	2502.8	2268.5	2474.5	2231.8	-0.13	0.7295
Tbc1d30	2580.7	2148.2	2219.6	2418.2	2585.4	2414.8	0.16	0.6655
Klhdc8b	2580.7	2523.5	2687.1	2099.7	2302.0	1821.5	-0.26	0.2977
Spast	2579.7	2359.6	2486.6	2308.9	2486.8	2337.8	0.00	1.0000
Hdac3	2579.7	2827.8	2738.5	2566.8	2619.3	2224.6	-0.16	0.6421
Cstf2t	2578.6	2383.2	2300.2	2562.6	2566.9	2454.6	0.07	0.9073
Dr1	2578.6	2422.4	2381.9	2337.5	2540.2	2158.5	-0.05	0.9534
Cyth1	2577.5	2601.0	2470.5	2813.1	2689.1	2499.9	0.04	0.9649

Faah	2577.5	2505.3	2468.5	2496.7	2469.3	2284.4	-0.05	0.9729
Podxl2	2577.5	2582.8	2876.6	2359.8	2630.5	2338.7	-0.08	0.9126
Try10	2576.5	4679.9	5604.0	5222.8	3387.3	6221.8	0.07	0.9522
Vbp1	2576.5	2493.5	2475.6	2229.2	2331.8	2028.9	-0.17	0.6131
Cog4	2575.4	2454.3	2523.9	2455.3	2615.1	2656.6	0.06	0.9559
Mettl26	2575.4	2687.5	2859.4	2416.1	2556.6	1979.1	-0.19	0.6068
Col5a2	2574.4	2507.1	3442.8	4357.6	3942.7	5991.7	0.48	0.0982
Cep120	2574.4	2399.6	2247.9	2699.5	2691.1	2802.5	0.17	0.6074
Dlgap4	2573.3	2393.3	2582.4	2113.5	2357.4	2615.0	-0.03	0.9806
Fgd2	2573.3	2517.2	2407.0	2082.7	2419.0	1813.4	-0.22	0.4893
Pdgfa	2572.2	2419.7	2780.8	2554.1	2640.8	2414.8	0.04	0.9615
Rps21	2572.2	2850.6	3166.7	2730.3	3102.9	2345.0	-0.06	0.9679
Timp2	2571.2	2307.6	3324.9	3748.3	3247.6	4904.8	0.55	0.0039
Strn	2570.1	2017.9	2048.4	2428.8	2439.6	2910.2	0.27	0.2911
Wdr37	2569.0	2354.1	2490.7	2692.1	2753.8	2635.8	0.16	0.5979
Sap18b	2569.0	2462.5	2606.5	2447.9	2571.0	2143.1	-0.05	0.9679
Pgp	2569.0	2575.5	2266.0	2434.1	2421.1	2207.4	-0.10	0.8347
Rpsa	2569.0	2826.0	5174.8	2696.3	2643.9	2211.0	-0.21	0.7764
Fyn	2568.0	2414.2	2615.6	2479.8	2466.3	2589.6	0.04	0.9798
Snph	2566.9	2504.4	2595.5	2523.3	2417.0	2632.2	0.00	1.0000
Pex7	2566.9	2493.5	2559.2	2464.9	2381.0	2101.4	-0.10	0.8669
Slc25a22	2566.9	2591.0	2385.9	2476.6	2214.7	2418.4	-0.11	0.8445
Ercc6l2	2565.8	2230.2	2191.4	2330.1	2228.1	2574.2	0.07	0.9299
Parg	2564.8	2544.5	2430.2	2588.0	2554.6	2385.8	-0.01	1.0000
Lpcat1	2564.8	2636.5	2531.0	2297.2	2348.2	2343.2	-0.16	0.6071
Leprotl1	2562.6	2427.9	2736.5	2544.5	2734.3	2502.7	0.07	0.9352
Vgf	2561.6	2794.1	1998.0	4527.5	4040.3	3903.0	0.55	0.0005
Arhgef28	2561.6	2281.2	2755.7	2819.5	2546.4	2542.5	0.16	0.6695
Ankrd46	2561.6	2582.8	2782.9	2402.3	2573.1	2314.3	-0.09	0.8619
Asna1	2561.6	2887.0	2628.7	2760.0	2831.8	2294.3	-0.10	0.8820
Fam183b	2559.4	2662.0	2443.3	2186.8	2763.0	1986.4	-0.16	0.7607
Zeb1	2557.3	2294.9	2507.8	3020.1	2938.6	3572.4	0.39	0.0235
Edf1	2557.3	2668.4	2744.6	2463.8	2608.0	2313.3	-0.10	0.8206
Dnajb2	2556.2	2511.7	2322.4	2322.7	2444.7	2223.7	-0.09	0.8560
Ppp1r16a	2556.2	2791.4	2615.6	2399.1	2427.3	2301.6	-0.20	0.4140
Acvr2a	2555.2	2099.9	2464.5	2618.8	2660.3	2761.7	0.29	0.1074
Clec14a	2555.2	2597.3	2462.5	2582.7	2818.4	2919.3	0.09	0.8445
Ecd	2555.2	2406.0	2376.8	2361.9	2393.4	2386.7	-0.02	0.9902
Elavl4	2555.2	2322.2	2761.7	2249.4	2335.9	2288.9	-0.04	0.9522
Acadl	2555.2	2537.2	2713.3	2376.8	2642.9	2387.6	-0.04	0.9773
Tpm3-rs7	2554.1	2643.8	2589.4	2556.2	2517.6	2743.6	-0.01	1.0000
Chfr	2550.9	2546.3	2382.9	2449.0	2131.5	2096.9	-0.17	0.6618
Crip2	2549.8	2314.9	2748.6	2808.8	2882.1	3309.7	0.30	0.1378
Gas5	2549.8	2598.2	2662.0	2754.7	2803.0	2955.5	0.12	0.7656
Srf	2549.8	2502.6	2467.5	2644.3	2328.7	2504.5	-0.01	1.0000
Golph3l	2549.8	2678.4	2180.3	2089.1	2158.2	1986.4	-0.30	0.1333
Evpl	2548.8	2468.0	2318.4	2479.8	2478.6	2464.6	0.01	1.0000
Rxrb	2547.7	2724.9	2817.1	2528.6	2562.8	2208.3	-0.14	0.7477
Fscn1	2546.6	2274.8	3241.3	2723.9	2796.9	3410.2	0.26	0.3479
Rps3a1	2546.6	2491.7	2997.5	2625.2	2952.9	2427.5	0.06	0.9439
Eif6	2546.6	2796.8	2665.0	2570.0	2668.5	2280.7	-0.13	0.7830
Iws1	2545.6	2501.7	2334.5	2683.6	2489.9	3067.9	0.12	0.8053
Erc1	2545.6	2594.6	2242.8	2783.4	2497.1	3054.3	0.11	0.8375
Lsm14b	2545.6	2365.9	2621.7	2420.3	2713.7	2519.9	0.08	0.9015
Uvrag	2545.6	2536.3	2457.4	2720.7	2391.3	2438.3	-0.01	1.0000
Csnk1g3	2544.5	2399.6	2727.4	2708.0	2659.3	2401.2	0.08	0.9105
Napg	2544.5	2433.3	2316.4	2275.9	2362.6	2315.2	-0.06	0.9186
Ninj1	2544.5	2386.0	2346.6	2201.6	2392.3	2001.8	-0.11	0.8099
Dsg2	2543.4	2549.0	2591.4	2984.0	2931.4	3571.5	0.27	0.2470
Otud7b	2543.4	2147.3	2151.1	2536.0	2422.1	2776.2	0.21	0.4669
Atxn7	2543.4	2417.9	2416.1	2731.3	2303.0	2802.5	0.09	0.8710
Gxylt1	2543.4	2411.5	2324.4	2609.3	2325.6	2670.2	0.06	0.9495
Anxa2	2541.3	2587.3	3001.5	2860.9	3157.3	3485.4	0.24	0.4100
Slc25a53	2541.3	2519.0	2572.3	2485.1	2603.9	2305.2	-0.03	1.0000
Nsfl1c	2540.3	2847.9	2908.8	2560.4	2823.6	2459.2	-0.10	0.8651
Map2k4	2539.2	2419.7	2510.8	2479.8	2524.8	2559.7	0.05	0.9515
Kremen1	2539.2	2377.8	2535.0	2246.2	2388.2	2372.2	-0.04	0.9923
Gpatch2	2538.1	2435.2	2382.9	2469.1	2291.7	2520.8	-0.01	1.0000
Sphk2	2537.1	2280.3	2485.6	2205.9	2478.6	2048.0	-0.04	0.9880
Uqcrh	2537.1	2568.2	2875.6	2487.2	2762.0	2065.2	-0.08	0.9343
Pygo2	2537.1	2535.4	2386.9	2290.8	2297.9	2219.1	-0.14	0.6822
Cnnm2	2536.0	2515.3	2192.4	2368.3	2331.8	2487.3	-0.05	0.9731
Slc39a1	2536.0	2477.1	2473.5	2198.5	2524.8	2384.9	-0.06	0.9343
Lcor	2534.9	2302.2	1901.3	2401.2	1900.5	2681.1	0.02	0.9896
Clasrp	2533.9	2279.4	2168.3	2373.6	2112.0	2419.3	0.01	1.0000
Pias1	2531.7	2266.6	2585.4	2493.6	2634.7	2516.2	0.13	0.7134

Uchl1	2531.7	2178.3	3792.4	2380.0	2334.8	2260.8	-0.04	0.9956
Gm10080	2531.7	2837.8	2799.0	2618.8	3053.6	2216.4	-0.08	0.9376
Rap1gds1	2530.7	2483.5	2814.1	2740.9	2731.2	2857.7	0.13	0.7524
Ezr	2530.7	2681.1	3128.5	2715.4	2823.6	3453.7	0.12	0.8113
Ap1ar	2530.7	2642.0	2669.0	2528.6	2783.5	2525.3	-0.01	1.0000
Ide	2529.6	2522.6	2222.7	2615.6	2504.3	4495.4	0.19	0.7939
Arhgef18	2529.6	2435.2	2358.7	2567.9	2416.0	2682.9	0.06	0.9280
Arpc4	2529.6	2446.1	2493.7	2489.3	2531.0	2567.0	0.04	0.9576
Pgd	2529.6	2626.5	2658.9	2756.8	2676.8	2644.9	0.03	0.9662
Src	2529.6	2243.9	2371.8	2116.7	2229.1	2043.4	-0.08	0.9011
Tmem64	2528.5	2163.7	2112.8	2595.5	2511.4	2572.4	0.20	0.4878
Gm37494	2527.5	2408.8	2090.7	2686.8	2297.9	2841.4	0.11	0.8599
P2ry1	2527.5	2138.2	2392.9	2300.4	2624.4	1841.4	0.02	0.9858
Arih2	2527.5	2358.6	2143.1	2458.5	2148.0	2298.9	-0.03	0.9778
Piezo2	2525.3	2631.0	2592.4	2871.5	2517.6	3834.1	0.19	0.6819
Slc36a4	2525.3	2420.6	2269.0	2514.8	2671.6	2589.6	0.09	0.8465
Phc1	2525.3	2530.8	2314.3	2434.1	2414.9	2636.7	-0.01	1.0000
Rybp	2525.3	2415.1	2320.4	2428.8	2356.4	2337.8	-0.02	0.9884
Mgat4b	2525.3	2782.3	3118.4	2519.0	2663.4	2545.2	-0.10	0.8465
Cd276	2524.3	2579.1	2555.2	2360.9	2394.4	2559.7	-0.07	0.9244
Tmem106c	2524.3	2665.7	2805.0	2427.7	2631.6	2104.1	-0.14	0.7524
Arg1	2523.2	2656.5	2582.4	2233.5	2282.5	1625.9	-0.30	0.2419
Glce	2522.1	2292.1	2487.6	2300.4	2217.8	2387.6	-0.01	1.0000
Herpud2	2522.1	2649.3	2285.1	2343.9	2680.9	2394.9	-0.07	0.9467
Xpo4	2521.1	2508.1	2149.1	2740.9	2403.6	3041.6	0.12	0.8042
Vldlr	2520.0	2247.5	2303.3	2137.9	2213.7	2233.6	-0.04	0.9515
Fbxo9	2520.0	2467.1	2560.2	2284.4	2337.9	1981.8	-0.15	0.7168
St3gal5	2517.9	2734.0	3006.5	2666.6	2797.9	2638.5	-0.02	1.0000
Xab2	2516.8	2389.6	2285.1	2667.7	2708.6	2509.0	0.12	0.7571
Zgpat	2516.8	2562.7	2503.8	2247.3	1953.9	1855.0	-0.30	0.1494
Pdlim5	2514.7	2115.4	2056.4	2642.2	2643.9	2908.4	0.31	0.1474
Zfp865	2514.7	2412.4	2374.8	2839.6	2549.4	3038.9	0.19	0.5557
Zfp507	2514.7	2301.2	2182.4	2604.0	2397.5	2748.1	0.14	0.7192
Ikbkb	2513.6	2677.5	2490.7	2453.2	2564.8	2718.2	-0.04	0.9850
Mcmbp	2512.5	2357.7	2452.4	2807.8	2636.7	2824.2	0.20	0.4418
Stk35	2512.5	2394.2	2211.6	2702.7	2531.0	2806.1	0.15	0.7148
Ndufa9	2512.5	2731.3	2753.6	2792.9	2900.6	2626.7	0.03	0.9851
Synj2bp	2512.5	2363.2	2465.5	2450.0	2552.5	2288.9	0.03	1.0000
Rab6b	2511.5	2091.7	3183.9	2068.9	2316.4	2692.0	0.06	0.9679
Cnpy3	2511.5	2715.8	2551.1	2381.0	2585.4	2223.7	-0.15	0.7108
Fras1	2508.3	2196.5	1957.7	2829.0	2383.1	3522.6	0.32	0.2260
Iqsec1	2508.3	2682.1	2612.6	2889.5	2707.6	3422.9	0.16	0.6952
Cep250	2507.2	2826.9	2283.1	2969.1	2553.5	3474.5	0.12	0.8264
Fktn	2507.2	2536.3	2145.1	2408.6	2417.0	2557.9	-0.02	0.9877
Clcn5	2506.2	2162.8	2094.7	2376.8	2372.8	2784.3	0.17	0.6397
Axl	2504.0	2768.6	2887.6	3119.9	3143.9	4187.4	0.29	0.2768
Smad1	2504.0	2457.9	3038.8	2631.6	2759.9	2597.8	0.08	0.9263
Snx9	2504.0	2455.2	2673.0	2648.5	2660.3	2526.2	0.07	0.8958
Frmd4b	2503.0	2378.7	2420.1	2450.0	2566.9	2651.2	0.09	0.8520
Epn2	2503.0	2504.4	2494.7	2588.0	2697.3	2574.2	0.06	0.9513
Dhx57	2501.9	2391.4	2302.3	2463.8	2230.1	2513.5	0.01	1.0000
Slc44a2	2500.8	2488.0	2689.2	2599.7	2873.9	3009.0	0.16	0.6469
Arhgef5	2498.7	2507.1	2277.1	2556.2	2543.3	2874.9	0.09	0.8965
Smcr8	2498.7	2382.3	2246.8	2354.5	2319.4	2442.9	0.00	1.0000
Zmat3	2496.6	2148.2	2509.8	2806.7	2560.7	2894.9	0.29	0.1174
Glod4	2495.5	2341.3	2414.1	2174.0	2213.7	1942.9	-0.14	0.6949
Rcn1	2494.4	2570.9	2697.2	2750.5	2594.6	2750.8	0.06	0.9503
Ano8	2494.4	2437.9	2596.5	2492.5	2260.9	2138.5	-0.09	0.9078
Efhd2	2493.4	2516.3	3024.7	2338.6	2506.3	2889.4	0.01	1.0000
Ptges3-ps	2493.4	2539.9	2396.0	2404.4	2689.1	2334.2	-0.03	0.9877
Cox7a2	2493.4	2761.3	2578.3	2497.8	2682.9	2273.5	-0.12	0.7830
Ubxn2a	2492.3	2434.3	2385.9	2469.1	2544.3	2511.7	0.04	0.9557
Rsbn1l	2491.2	2410.6	2512.8	2589.1	2617.2	2418.4	0.06	0.9410
Tspan31	2491.2	2432.4	2269.0	2277.0	2470.4	2152.1	-0.07	0.9155
Denr	2491.2	2621.0	2471.5	2430.9	2590.5	2291.6	-0.08	0.8710
Dhx16	2490.2	2540.8	2405.0	2364.1	2210.6	2381.3	-0.11	0.7830
Rrm2b	2490.2	2304.0	2387.9	2233.5	2281.5	1911.2	-0.11	0.8236
Slc35b2	2490.2	2602.8	2631.7	2257.9	2604.9	2223.7	-0.12	0.8143
Sptlc2	2489.1	2335.0	2466.5	2587.0	2622.3	2779.8	0.16	0.5911
Spred2	2488.0	2017.9	2253.9	2542.4	2683.9	2624.0	0.31	0.0853
Vps51	2488.0	2857.9	2687.1	2518.0	2558.7	2499.0	-0.14	0.6597
Usp38	2487.0	2302.2	2322.4	2394.8	2405.7	2388.5	0.04	0.9516
Hspa12a	2487.0	2568.2	2314.3	2623.1	2416.0	2766.2	0.03	0.9731
Plod1	2485.9	2689.3	2752.6	3166.6	3244.5	3407.5	0.26	0.2095
Dmbt1	2484.8	4534.2	3908.3	7913.8	4902.8	8352.1	0.46	0.1531
Dmpk	2484.8	2344.1	2408.1	2134.8	2449.8	2641.2	0.03	0.9885

Carmil1	2484.8	2683.0	2538.0	2560.4	2707.6	2608.6	-0.02	1.0000
Trmt6	2484.8	2625.6	2525.9	2567.9	2501.2	2537.1	-0.04	0.9810
Sipa1	2483.8	2323.1	2360.7	2393.8	2258.9	2459.2	0.02	0.9884
Zkscan8	2482.7	2381.4	2204.5	2349.2	2227.0	2735.4	0.03	0.9731
Efnb2	2481.6	2486.2	2633.7	2967.0	2926.3	3026.2	0.23	0.2777
Wdfy1	2481.6	3425.5	2205.5	3337.5	3399.6	3401.2	0.05	0.9624
Pdcl	2481.6	2647.4	2293.2	2395.9	2590.5	2390.3	-0.08	0.8869
Ephb4	2480.6	2679.3	2920.9	2924.5	2908.8	3464.6	0.18	0.6042
Rtf2	2480.6	2499.9	2654.9	2317.3	2675.7	2083.3	-0.08	0.9342
Pdia5	2479.5	2661.1	2385.9	2274.9	2539.2	2114.1	-0.16	0.6511
Mmp15	2478.5	2529.0	2692.2	2911.8	2956.0	3516.2	0.27	0.2572
Ier5	2477.4	1971.5	2102.8	2239.9	2525.8	2287.1	0.19	0.5941
Mcm6	2476.3	3427.3	2808.0	4267.4	3515.6	4685.6	0.31	0.1876
Tmem185a	2476.3	2329.5	2583.4	2433.1	2677.8	2067.9	0.01	1.0000
Rp2	2476.3	2358.6	2228.7	2184.7	2390.3	2040.7	-0.09	0.8772
Abi2	2475.3	2102.6	2179.3	2498.9	2521.7	2613.2	0.23	0.3390
Trmt2a	2475.3	2539.9	2192.4	2384.2	2409.8	2308.8	-0.07	0.9269
Notch4	2474.2	2657.5	2383.9	2733.5	2934.5	3310.6	0.17	0.6131
Mepce	2473.1	2513.5	2341.6	2562.6	2662.4	2519.9	0.04	0.9542
Tmem258	2473.1	2917.1	2728.5	2443.7	2503.2	2253.6	-0.23	0.3225
Socs5	2468.9	2346.8	2144.1	2620.9	2664.4	2557.0	0.14	0.6983
Dcaf12	2468.9	2545.4	2428.2	2515.9	2708.6	2354.1	0.00	1.0000
Zfp612	2467.8	2155.5	2083.6	2318.4	2367.7	2283.5	0.08	0.8772
Ccdc97	2467.8	2417.9	2346.6	2284.4	2186.0	2400.3	-0.07	0.9067
Sdk1	2466.7	2292.1	2023.2	2384.2	2227.0	2356.8	0.02	0.9893
Hsd17b12	2466.7	2805.0	2278.1	2529.7	2526.8	2499.0	-0.11	0.8037
Pde3a	2464.6	2334.0	2390.9	3164.5	2592.6	3691.0	0.35	0.1173
Fzd4	2462.5	2270.3	2193.4	3082.7	2997.1	3624.0	0.44	0.0072
Ror2	2462.5	2654.7	2226.7	2386.3	2242.4	2204.7	-0.17	0.5634
Zfp710	2461.4	2632.0	2628.7	2547.7	2535.1	2367.7	-0.07	0.9096
Psmb2	2460.3	2632.0	2615.6	2581.7	2497.1	2306.1	-0.08	0.8791
Tbc1d8	2460.3	2392.4	2389.9	2066.8	2190.1	2107.7	-0.16	0.6025
Lbr	2459.3	2340.4	2289.2	2542.4	2435.5	2787.1	0.13	0.7623
Pmepa1	2458.2	2130.0	2540.0	2377.9	2417.0	2688.3	0.17	0.5855
Ak2	2458.2	2794.1	2770.8	2401.2	2440.6	2355.0	-0.19	0.5477
Gm1840	2456.1	2233.8	2450.4	2501.0	2630.5	2605.0	0.17	0.5417
Acap3	2456.1	2574.6	2360.7	2260.0	2083.3	2374.0	-0.17	0.6350
Rps25-ps1	2455.0	2605.5	2850.4	2398.0	2716.8	2518.0	-0.03	0.9902
Prdx3	2455.0	2364.1	2278.1	2283.4	2420.1	1967.3	-0.08	0.8946
Atic	2452.9	2326.8	2208.6	2549.8	2578.2	2865.0	0.17	0.5813
Gm2423	2452.9	2440.6	2258.9	2176.2	2285.6	2133.1	-0.13	0.7455
Gm21399	2452.9	2831.5	2775.8	2453.2	2520.7	2184.7	-0.20	0.4812
Gucy1a1	2450.7	2294.9	2653.9	3034.9	3063.8	3444.7	0.40	0.0121
Adam15	2449.7	2336.8	2409.1	2504.2	2791.7	2644.9	0.16	0.6716
Prc1	2449.7	2427.0	1539.5	2108.2	2160.3	2643.0	0.00	1.0000
Atp6v0c-ps2	2449.7	2664.7	2624.7	2349.2	2772.2	2504.5	-0.05	0.9737
Vps28	2449.7	2663.8	2715.4	2342.8	2664.4	1984.5	-0.16	0.7468
Atp9b	2448.6	2737.6	2559.2	2846.0	2707.6	2874.9	0.05	0.9679
Gm12481	2448.6	2844.2	2810.1	2935.2	3113.1	2384.9	0.01	1.0000
Cited2	2447.6	1912.2	2558.2	3348.1	2739.4	2380.4	0.40	0.0502
Crtc3	2447.6	2240.2	2321.4	2662.3	2644.9	2824.2	0.24	0.2646
Syngr1	2447.6	2437.0	2697.2	1991.5	2229.1	2132.2	-0.19	0.5429
Asap1	2446.5	2317.6	2383.9	2935.2	2763.0	3100.5	0.30	0.1108
Rbm17	2446.5	2333.1	2578.3	2660.2	2660.3	2442.0	0.12	0.7916
Gpcpd1	2446.5	2673.9	2757.7	2740.9	2737.3	2947.4	0.07	0.9126
Rsl1d1	2446.5	2481.6	2465.5	2402.3	2251.7	2313.3	-0.09	0.8587
Atg2a	2446.5	2435.2	2251.9	2226.1	2233.2	2153.9	-0.12	0.7847
Gpd1l	2446.5	2529.0	2332.5	2125.2	2103.8	2218.2	-0.20	0.4157
Slc35g1	2446.5	2121.8	2179.3	1787.6	1708.5	1481.8	-0.33	0.1174
Trappc13	2445.4	2131.8	2308.3	2272.8	2420.1	2042.5	0.04	0.9559
Dixdc1	2445.4	2230.2	2044.3	2156.0	2172.6	2319.7	-0.01	1.0000
Mid2	2444.4	2071.7	1961.7	2370.4	2444.7	2453.7	0.18	0.5896
Vps29	2443.3	2542.7	2580.3	2553.0	2621.3	2104.1	-0.06	0.9493
Epb41l2	2442.2	2107.2	2540.0	2859.8	2882.1	3370.4	0.43	0.0062
Golm1	2442.2	2345.0	2084.6	2385.3	2473.5	2209.2	0.01	1.0000
Ddhd2	2441.2	2225.6	2349.6	2501.0	2192.1	2450.1	0.07	0.9269
Fam214a	2441.2	2629.2	2550.1	2481.9	2478.6	2567.0	-0.05	0.9436
Hdhd5	2441.2	2429.7	2221.7	2143.3	2264.0	1968.2	-0.16	0.6258
Slc35c1	2440.1	2379.6	1880.1	2093.4	2079.2	1986.4	-0.16	0.6983
Tmem134	2440.1	2749.5	2426.2	2388.5	2434.4	2022.6	-0.21	0.4553
Stk40	2439.0	2153.7	2168.3	2381.0	2288.6	2413.0	0.11	0.8119
Nasp	2439.0	2758.6	2289.2	2885.3	2664.4	2890.3	0.05	0.9369
Krit1	2439.0	2472.5	2484.6	2561.5	2353.3	2504.5	0.00	1.0000
Commd7	2439.0	2376.0	2361.7	2361.9	2478.6	2269.9	-0.01	1.0000
Mmp14	2438.0	2543.6	2908.8	3051.9	3036.1	4345.9	0.35	0.1656
Rrp7a	2438.0	2370.5	2451.4	2361.9	2356.4	1950.1	-0.09	0.8714

Rpl17-ps10	2438.0	2644.7	2633.7	2413.9	2467.3	1893.1	-0.19	0.6196
Mnx1	2438.0	2974.5	2592.4	2336.5	2142.8	2078.7	-0.37	0.0231
Maml1	2435.8	2095.4	2265.0	2636.9	2398.5	2682.9	0.24	0.3025
Parn	2435.8	2460.7	2479.6	2627.3	2718.8	2451.9	0.07	0.8946
Fnbp1	2434.8	2382.3	2583.4	2382.1	2204.4	2822.4	0.03	0.9820
Tbc1d17	2434.8	2333.1	2528.0	2240.9	2536.1	2030.7	-0.05	0.9717
Marveld3	2434.8	2090.8	2288.2	1922.5	2015.5	1866.8	-0.12	0.7873
Mcm2	2433.7	3344.4	2427.2	4220.7	3433.5	4083.2	0.27	0.2336
Cc2d1a	2433.7	2274.8	2130.0	2266.4	2209.6	2310.6	-0.01	1.0000
Syn1	2432.6	2404.2	2579.3	2830.1	3010.4	2929.3	0.25	0.2417
Esco1	2432.6	2208.3	2216.6	2218.6	2286.6	2019.0	-0.03	0.9729
Txndc12	2432.6	2703.0	2656.9	2475.5	2572.0	2252.7	-0.12	0.7741
Rab35	2431.6	2370.5	2440.3	2378.9	2671.6	2437.4	0.06	0.9297
Rint1	2431.6	2341.3	1962.7	2337.5	2220.9	2438.3	0.01	1.0000
St6galnac6	2431.6	2384.2	2605.5	2185.7	2287.6	2096.0	-0.12	0.7648
Glis2	2429.4	2309.4	2379.8	2496.7	2310.2	2778.0	0.11	0.8207
Rragd	2429.4	1941.4	2167.2	2195.3	2371.8	1976.4	0.11	0.8314
Cldnd1	2429.4	2255.7	2476.6	2289.7	2481.7	2044.3	-0.01	1.0000
Anks3	2428.4	2237.5	2228.7	2444.7	2242.4	2121.3	0.01	1.0000
Sdhb	2427.3	2620.1	2580.3	2291.9	2766.1	2191.1	-0.09	0.9007
Trap1	2426.2	2560.0	2345.6	2417.1	2441.6	2169.3	-0.10	0.8214
Fam174a	2426.2	2443.4	2271.0	2089.1	2476.5	1928.4	-0.14	0.7477
Mmrn2	2425.2	2469.8	2664.0	3229.2	3309.2	3735.4	0.41	0.0084
Scmh1	2425.2	2211.1	2480.6	2436.2	2417.0	2484.5	0.11	0.8002
Cops6	2424.1	2557.2	2653.9	2429.9	2594.6	2486.3	-0.03	1.0000
Clk4	2423.0	2165.5	2061.5	2473.4	2191.1	2439.2	0.11	0.8304
Stim2	2423.0	2399.6	2588.4	2670.8	2525.8	2517.1	0.08	0.8666
Obsl1	2423.0	2472.5	2324.4	2304.6	2113.1	2865.0	-0.01	1.0000
Tiprl	2423.0	2295.8	2311.3	2177.2	2282.5	2055.2	-0.08	0.8802
Nfrkb	2422.0	2406.9	2310.3	2505.2	2464.2	2441.1	0.04	0.9649
Hmgxb3	2422.0	2307.6	2305.3	2356.6	2393.4	2314.3	0.02	1.0000
Rasal2	2420.9	1952.3	1798.5	2842.8	2447.8	2993.6	0.40	0.0526
Vps4a	2417.7	2249.3	2412.1	2366.2	2444.7	2338.7	0.06	0.9462
Slc6a17	2414.5	3167.6	2352.6	2957.5	2972.5	3303.4	0.02	0.9929
Kif5c	2413.5	2085.3	2379.8	2359.8	2254.8	2324.2	0.11	0.8188
Fam155a	2413.5	2887.0	2629.7	2669.8	2575.1	2323.3	-0.15	0.7185
Foxred2	2411.3	2491.7	1957.7	2179.3	2340.0	2238.2	-0.10	0.8560
Sephs2	2411.3	2306.7	2310.3	2226.1	2112.0	2004.5	-0.12	0.7817
Erf	2410.3	2164.6	2496.7	2445.8	2436.5	2334.2	0.12	0.7910
Ptpn21	2409.2	2407.8	2366.7	2524.3	2679.8	2692.9	0.12	0.7574
Cebpz	2409.2	2350.4	2319.4	2373.6	2308.1	2472.8	0.02	0.9915
Mboat7	2409.2	2494.4	2413.1	2111.4	2217.8	2169.3	-0.18	0.5060
Tm4sf1	2408.1	2563.6	2905.8	2936.2	3288.7	2959.2	0.22	0.4745
Klf11	2408.1	2301.2	2701.2	2598.7	2783.5	2339.6	0.12	0.7896
Ndufc2	2408.1	2610.1	2514.9	2613.5	2887.2	2248.1	0.00	1.0000
Ctnnbip1	2408.1	2509.9	2637.8	2614.6	2531.0	2249.0	-0.02	1.0000
Csnk2a3	2407.1	2550.0	2224.7	2492.5	2296.9	2429.3	-0.06	0.9365
Hsd17b4	2406.0	2621.9	2903.8	2686.8	2694.2	2658.4	0.03	0.9826
Plcb3	2404.9	2194.7	2315.4	1837.5	1818.4	2013.5	-0.21	0.4635
Fads1	2402.8	2585.5	2548.1	2957.5	2723.0	3072.4	0.17	0.6055
Dstyk	2402.8	2337.7	2331.5	2548.8	2361.5	2710.1	0.11	0.8164
Leprot	2402.8	2412.4	2747.6	2544.5	2592.6	2230.9	0.01	1.0000
A230057D06R	2402.8	2246.6	1740.0	2005.3	1627.4	2447.4	-0.09	0.9126
Faf1	2401.7	2560.9	2609.6	3026.5	2733.2	2849.6	0.15	0.6469
Galnt4	2401.7	2021.6	1868.0	1671.9	1962.1	1664.8	-0.18	0.6582
Gm4332	2400.7	2911.6	3186.9	2872.5	3142.9	3063.3	0.06	0.9681
Ccz1	2400.7	2346.8	2212.6	2270.6	2407.7	2098.7	-0.05	0.9542
Max	2399.6	2314.9	2475.6	2467.0	2401.6	2201.9	0.01	0.9966
Ace2	2399.6	1995.1	2010.1	1958.5	2074.0	2075.1	0.00	1.0000
Tuba4a	2399.6	2865.2	2523.9	2819.5	2463.2	2604.1	-0.08	0.9108
Eif2ak1	2398.5	2412.4	2535.0	2305.7	2458.1	2300.7	-0.04	0.9662
Dgkh	2396.4	2048.0	1659.4	2513.7	2230.1	2979.1	0.27	0.3907
Zbtb38	2396.4	2132.7	2163.2	2581.7	2418.0	2736.3	0.23	0.3445
Nrp2	2395.3	2173.7	2291.2	2991.4	2652.1	3508.1	0.40	0.0327
Rbm18	2395.3	2331.3	2311.3	2232.4	2448.8	2278.0	-0.01	1.0000
Fads6	2394.3	2445.2	2326.4	2004.2	1988.8	1957.4	-0.27	0.1548
Bcap31	2393.2	2331.3	2500.7	2440.5	2564.8	2543.4	0.09	0.8395
Pnpla6	2393.2	2318.6	2142.1	2242.0	2033.0	2232.7	-0.08	0.8793
Bet1	2392.1	2366.8	2337.5	2116.7	2331.8	2165.7	-0.09	0.8429
Pcgf2	2392.1	2345.0	2579.3	2186.8	2079.2	2341.4	-0.09	0.8455
Abhd14b	2392.1	2100.8	2206.5	1887.4	2134.6	1844.2	-0.11	0.8051
Dip2a	2391.1	2289.4	2165.2	2309.9	2121.3	2462.8	0.01	1.0000
Ncdn	2391.1	2178.3	2335.5	2197.4	2107.9	2295.2	0.00	1.0000
Jak2	2390.0	2400.6	2431.2	2443.7	2243.5	2487.3	-0.01	1.0000
Fbxo28	2387.9	2021.6	2008.1	2554.1	2424.2	2487.3	0.25	0.3202
Mex3c	2387.9	2232.0	2242.8	2684.6	2609.0	2763.5	0.24	0.2904

Mgll	2387.9	2355.0	2857.4	2704.8	2922.1	3021.7	0.23	0.3943
Arpc1b	2387.9	2475.3	2627.7	2418.2	2575.1	2749.9	0.05	0.9503
Gid8	2387.9	2356.8	2424.2	2383.2	2411.8	2191.1	-0.02	0.9932
Ggps1	2386.8	2110.8	2177.3	2252.6	2206.5	2039.8	0.02	1.0000
Rab28	2386.8	2406.9	2308.3	2181.5	2385.2	2074.2	-0.10	0.8314
Atxn1	2385.7	1874.0	2136.0	2342.8	2270.2	2573.3	0.27	0.2278
Ranbp10	2385.7	2509.9	2370.8	2530.7	2504.3	2355.0	-0.02	0.9927
Eml6	2385.7	2335.9	2299.2	2259.0	2256.8	2363.2	-0.02	1.0000
Tent4a	2384.7	1808.4	2101.8	2184.7	2027.8	2096.0	0.15	0.6832
Fam49b	2384.7	2222.0	2491.7	2351.3	2338.9	1946.5	-0.03	0.9810
Rad23a	2383.6	2690.3	2350.6	2528.6	2552.5	2333.3	-0.09	0.8795
Fkrp	2383.6	2520.8	2344.6	2368.3	2421.1	1915.7	-0.14	0.7636
Dnajc6	2382.6	2314.9	2530.0	2558.3	2579.2	2252.7	0.07	0.9471
Fbxw2	2382.6	2331.3	2355.7	2343.9	2423.1	2184.7	-0.01	1.0000
Tomm34	2382.6	2249.3	2465.5	2114.6	2162.3	2121.3	-0.08	0.8710
Bambi	2381.5	2036.1	2359.7	2295.1	2465.2	1827.9	0.05	0.9532
Oat	2379.4	2901.6	2669.0	3359.8	3340.0	3387.6	0.22	0.3390
Ppp1r12b	2378.3	2621.0	2202.5	2668.7	2155.2	3071.5	0.04	0.9654
Galk2	2378.3	2369.6	2346.6	2050.9	2301.0	1980.0	-0.15	0.7271
Psme1	2377.2	2424.2	2701.2	2253.7	2503.2	2122.2	-0.08	0.9244
Car8	2377.2	2210.1	2055.4	2016.9	2008.3	2097.8	-0.10	0.8353
Grb7	2377.2	2621.0	2474.5	2145.4	2469.3	2021.7	-0.20	0.5234
Zmat2	2376.2	2112.7	2388.9	2123.1	2405.7	1932.0	0.00	1.0000
Stk11ip	2376.2	2457.0	2381.9	2126.3	2176.7	2188.3	-0.16	0.5855
Zfp651	2375.1	2673.9	2656.9	2764.3	2901.6	2880.4	0.09	0.8455
Miga1	2375.1	2170.1	2475.6	2355.6	1925.2	2275.3	-0.02	0.9956
Polr3k	2375.1	2474.3	2310.3	2412.9	2366.7	2422.9	-0.03	0.9737
Elmo3	2375.1	2238.4	2311.3	1887.4	2013.5	1786.2	-0.22	0.3470
Capn5	2374.0	2345.9	2405.0	2132.6	2029.9	2215.5	-0.13	0.7607
Usp14	2373.0	2447.9	2492.7	2297.2	2267.1	2505.4	-0.05	0.9500
Cldn7	2373.0	2240.2	2338.5	2014.8	2222.9	2115.9	-0.08	0.9078
Pomp	2373.0	2660.2	2634.8	2472.3	2473.5	2144.0	-0.14	0.7414
Bcl2l1	2371.9	2183.7	2452.4	2346.0	2491.9	2331.5	0.10	0.8285
Lima1	2370.8	2160.0	2609.6	2702.7	2561.8	2881.3	0.27	0.2237
Mdn1	2370.8	2458.9	1699.7	2650.7	1917.0	3157.5	0.11	0.9343
Eef1g	2369.8	2480.7	2565.2	2653.9	2717.8	2577.8	0.09	0.8556
Xpnpep1	2369.8	2530.8	2192.4	2507.4	2552.5	2413.9	0.00	1.0000
Ech1	2369.8	2289.4	2283.1	2174.0	2295.8	1961.0	-0.09	0.8647
Gid4	2367.6	2340.4	2167.2	2422.4	2349.2	2473.7	0.05	0.9681
Smurf1	2366.6	2355.9	2640.8	2544.5	2649.0	2671.1	0.13	0.7427
Trappc12	2366.6	2408.8	2294.2	2421.4	2136.7	2315.2	-0.06	0.9269
Plekhm2	2365.5	2243.9	2374.8	2359.8	2090.5	2273.5	-0.01	0.9956
Vamp8	2365.5	2180.1	2633.7	2088.1	2320.5	1781.7	-0.10	0.8560
Rpl7l1	2364.4	2341.3	2083.6	2300.4	2248.6	2045.2	-0.07	0.9100
Syngr2	2364.4	2373.2	2317.4	2176.2	2284.5	2174.8	-0.09	0.8516
Samd8	2363.4	2038.0	2115.9	2389.5	2327.7	2408.5	0.18	0.5343
E2f4	2363.4	2375.0	2002.0	2133.7	1987.8	2111.4	-0.15	0.6819
Bag4	2362.3	2027.0	2208.6	2151.7	2333.8	2197.4	0.10	0.8314
Brd7	2362.3	2215.6	2431.2	2352.4	2447.8	2234.5	0.06	0.9280
Oaz1	2362.3	2512.6	2686.1	2493.6	2620.3	2635.8	0.04	0.9881
Atg16l1	2362.3	2216.5	2265.0	2228.2	2172.6	2157.6	-0.03	0.9798
Trib1	2361.2	2162.8	1928.5	2356.6	2110.0	2059.7	0.01	1.0000
Gprc5c	2360.2	2349.5	2612.6	1726.1	1826.6	2198.3	-0.27	0.2944
Mat2b	2358.0	2087.2	2166.2	2200.6	2239.4	2146.7	0.05	0.9694
Hdac4	2357.0	2122.7	2020.1	2497.8	2232.2	2626.7	0.18	0.6128
Gad1	2355.9	2507.1	2482.6	3206.9	3132.6	3204.6	0.32	0.0454
Sar1b	2355.9	2386.0	2359.7	2088.1	2273.2	1869.5	-0.17	0.6042
Emc1	2354.8	2185.5	2216.6	2756.8	2533.0	2663.9	0.24	0.2557
Klhl22	2353.8	2241.1	2484.6	2423.5	2489.9	2184.7	0.05	0.9471
Fbn1	2352.7	2543.6	2861.5	4851.2	4286.7	8351.2	0.53	0.0696
Ap1m2	2352.7	2498.0	2504.8	2087.0	2433.4	1920.2	-0.18	0.6045
Col6a2	2351.7	2996.4	3679.6	4937.2	5304.2	8489.8	0.49	0.1264
Dhx38	2351.7	2578.2	2610.6	2571.1	2680.9	2759.0	0.05	0.9576
Ripply3	2351.7	2652.0	2436.3	2216.5	2542.2	1984.5	-0.18	0.6221
Pithd1	2350.6	2473.4	2270.0	2405.5	2700.4	2111.4	-0.02	0.9980
Ralb	2350.6	2498.9	2515.9	2444.7	2463.2	2109.5	-0.08	0.9256
Dpysl3	2349.5	1776.5	3919.4	2143.3	1983.7	3095.9	-0.02	1.0000
Arhgap44	2349.5	2314.9	2114.9	2039.2	2135.7	1945.6	-0.16	0.6618
Nod1	2348.5	2421.5	2247.9	2085.9	2176.7	2247.2	-0.13	0.7255
Gpr27	2346.3	2059.8	2405.0	1892.7	2254.8	1946.5	-0.05	0.9515
Unk	2345.3	2416.9	2180.3	2620.9	2309.2	2595.9	0.06	0.9541
Bcas2	2345.3	2228.4	2339.5	2266.4	2389.3	2293.4	0.04	0.9525
Nvl	2345.3	2618.3	2360.7	2792.9	2264.0	2827.8	0.03	0.9850
Slc35c2	2345.3	2304.9	2220.6	2100.8	2233.2	2211.9	-0.07	0.9096
Gcc1	2344.2	2148.2	1896.2	2346.0	2226.0	2241.8	0.07	0.9073
Crtc2	2343.1	2236.6	2447.3	2390.6	2245.5	2232.7	0.02	0.9884

Cdcp1	2343.1	2045.3	1858.9	1991.5	2020.7	1991.8	-0.03	0.9810
Scand1	2342.1	2468.0	2206.5	2169.8	2387.2	1839.6	-0.16	0.6819
Zfp622	2341.0	2226.5	2352.6	2236.7	2265.0	1791.6	-0.09	0.8827
Traf6	2338.9	2262.1	2442.3	2338.6	2439.6	2538.0	0.09	0.8787
Wnk2	2337.8	2292.1	2282.1	2550.9	2347.2	2697.4	0.13	0.7844
Psmf1	2337.8	2225.6	2309.3	2247.3	2439.6	2274.4	0.05	0.9486
Akr1c14	2337.8	1608.9	1454.9	1846.0	2326.6	1354.1	0.03	0.9893
Cr1l	2337.8	2344.1	2270.0	2274.9	2315.3	2189.3	-0.04	0.9500
Heatr5b	2335.7	2323.1	2193.4	2488.3	2414.9	2708.3	0.12	0.7952
Rsbn1	2335.7	2098.1	2155.2	2204.8	2178.8	2190.2	0.04	0.9559
Ino80	2334.6	2188.3	2023.2	2344.9	1860.5	2343.2	0.00	1.0000
Ssu72	2334.6	2430.6	2595.5	2270.6	2479.6	1866.8	-0.12	0.8103
Col6a1	2333.5	2413.3	3467.0	4317.3	4721.0	7495.3	0.49	0.1182
Gm6206	2333.5	2349.5	2324.4	2222.9	2401.6	1991.8	-0.08	0.8980
Hcfc2	2332.5	2034.3	1859.9	2344.9	2036.1	2140.3	0.08	0.9108
Serpinb6a	2332.5	2274.8	2290.2	2344.9	2523.8	2365.0	0.07	0.9171
Ptpn13	2332.5	2222.9	1748.1	2112.5	1872.8	2428.4	-0.02	0.9884
Spred1	2331.4	2207.4	2460.4	2608.2	2713.7	2911.2	0.27	0.1859
Lgals8	2331.4	2289.4	2433.2	2204.8	2230.1	2331.5	-0.03	0.9785
Unc5a	2330.3	2881.6	2215.6	3307.8	3335.9	2981.8	0.18	0.5097
Cog5	2330.3	2545.4	2450.4	2708.0	2817.4	2599.6	0.09	0.8666
Zbtb33	2330.3	2181.9	2119.9	2367.2	2356.4	2253.6	0.08	0.8866
Pex26	2330.3	2074.4	2164.2	2089.1	2095.6	1954.7	-0.03	0.9715
Naa40	2330.3	2312.2	2111.8	2197.4	2128.5	2325.1	-0.05	0.9521
Pofut1	2329.3	2161.9	2114.9	2321.6	2136.7	2480.9	0.08	0.8816
Sox18	2329.3	2714.9	2573.3	2234.5	2691.1	2407.5	-0.11	0.8112
Bicdl1	2329.3	2051.6	2071.5	1930.9	1900.5	1734.6	-0.14	0.7192
Gm29216	2328.2	2644.7	2646.8	2315.2	2828.7	2409.4	-0.05	0.9593
Gpd1	2328.2	2225.6	2150.1	1934.1	2216.8	1894.0	-0.13	0.7682
Pias3	2327.1	2399.6	2305.3	2425.6	2413.9	2256.3	-0.01	1.0000
Rpl36a-ps2	2327.1	2520.8	2863.5	2408.6	2547.4	2335.1	-0.05	0.9493
Mfsd11	2327.1	2565.4	2451.4	2380.0	2425.2	2317.9	-0.09	0.8814
H2-Q4	2326.1	2553.6	2117.9	2191.0	2471.4	1899.4	-0.16	0.7033
Fuca1	2325.0	2534.5	2540.0	2469.1	2615.1	2152.1	-0.05	0.9711
Krba1	2325.0	2185.5	2149.1	1988.3	1750.6	2089.6	-0.15	0.6912
Ppp4r2	2322.9	2104.5	2191.4	2289.7	2303.0	2220.1	0.09	0.8899
Dock8	2321.8	2209.2	2074.6	2820.5	2577.2	2693.8	0.26	0.2505
Gabrb3	2320.8	2182.8	2099.7	2164.5	2030.9	2234.5	-0.03	0.9835
Pigs	2320.8	2354.1	2368.8	2249.4	2275.3	2120.4	-0.08	0.8772
Tnfrsf1a	2319.7	2234.7	2306.3	2281.3	2222.9	2603.2	0.07	0.9114
Mbd5	2319.7	2245.7	2182.4	2352.4	2275.3	2461.9	0.07	0.9439
Pip4p1	2319.7	2532.6	2443.3	2536.0	2698.3	2134.9	-0.02	1.0000
Atp2b2	2318.6	2268.5	1723.9	2498.9	2391.3	2887.6	0.19	0.6074
Wdr5	2318.6	2351.4	2243.8	2147.5	2053.5	2097.8	-0.14	0.6819
Gm10250	2317.6	2752.2	2549.1	2583.8	2797.9	2414.8	-0.05	0.9498
Rassf4	2316.5	2449.7	2233.7	2462.8	2400.6	2409.4	0.00	1.0000
Slc25a25	2315.4	2086.2	1915.4	2521.2	2221.9	2258.1	0.14	0.7845
Trim26	2315.4	2404.2	2364.7	2627.3	2478.6	2488.2	0.07	0.8879
Sptssa	2315.4	2346.8	2618.6	2460.7	2631.6	2143.1	0.02	0.9931
Trim23	2314.4	2182.8	2204.5	2179.3	2340.0	1932.0	-0.03	0.9810
Anapc16	2314.4	2199.2	2309.3	2065.8	2101.8	1969.2	-0.10	0.8440
Vipas39	2314.4	2355.9	2442.3	2213.3	2206.5	2107.7	-0.11	0.7977
Gga3	2313.3	2243.9	1878.1	2228.2	2055.6	2401.2	0.01	1.0000
Aip	2311.2	2289.4	2530.0	2337.5	2631.6	1983.6	0.00	1.0000
Cep70	2311.2	2387.8	2215.6	2274.9	2142.8	2228.2	-0.09	0.8465
Ralgapa2	2309.0	2222.0	1915.4	2413.9	2181.9	2442.0	0.08	0.8946
Hmox2	2309.0	2447.0	2672.0	2326.9	2521.7	2278.0	-0.04	0.9812
Gtf2b	2309.0	2313.1	2231.7	1994.6	2052.5	1562.5	-0.26	0.3422
Itpr2	2308.0	1929.6	2424.2	2591.2	2618.2	3137.6	0.42	0.0149
Gm9892	2308.0	2210.1	2321.4	2316.3	2379.0	2238.2	0.05	0.9400
Dpy19l4	2306.9	2162.8	2186.4	2344.9	2328.7	2414.8	0.11	0.7873
Ube2m	2306.9	2304.0	2316.4	2375.7	2176.7	2250.8	-0.02	1.0000
Lratd2	2305.8	2065.3	1827.7	2342.8	2329.7	2465.5	0.18	0.6116
Slc9a1	2305.8	2154.6	2052.4	2476.6	2320.5	2590.5	0.17	0.6025
Stau2	2305.8	2240.2	2352.6	2272.8	2549.4	2191.1	0.05	0.9515
Asns	2304.8	2515.3	2501.8	2812.0	2666.5	2845.0	0.14	0.7056
Wbp4	2304.8	2195.6	2382.9	2244.1	2401.6	1875.9	-0.03	0.9810
Ccdc92	2302.6	1984.2	2005.0	2071.1	2064.8	2018.1	0.03	0.9839
Mast1	2302.6	2584.6	2546.1	2149.6	2074.0	2464.6	-0.17	0.6416
Eif2b5	2300.5	2493.5	2291.2	2442.6	2488.9	2239.1	-0.04	0.9813
Relt	2300.5	2391.4	2519.9	2066.8	2020.7	1701.9	-0.27	0.2468
Aspscr1	2299.4	2314.0	2294.2	2127.3	2192.1	2021.7	-0.12	0.7682
Plcb1	2298.4	2149.1	2131.0	2977.6	2650.1	2790.7	0.34	0.0543
Meaf6	2298.4	2389.6	2509.8	2315.2	2374.9	2474.6	0.00	1.0000
Pdk1	2298.4	2386.9	2328.5	2360.9	2464.2	2180.2	-0.02	0.9849
Galns	2298.4	2376.0	2248.9	2329.0	2208.6	2114.1	-0.08	0.9090

Bckdha	2298.4	2224.7	2536.0	1888.5	2134.6	1745.4	-0.20	0.5113
Aqp4	2298.4	2270.3	2174.3	1912.9	1862.5	1871.3	-0.24	0.2588
Zfp12	2297.3	2124.5	2013.1	2187.8	2402.6	2028.0	0.04	0.9858
Chordc1	2297.3	2228.4	2231.7	2113.5	2182.9	2146.7	-0.05	0.9472
Ssh1	2296.2	1918.6	2206.5	2236.7	2246.5	2472.8	0.21	0.3907
Desi2	2296.2	2172.8	2277.1	2408.6	2310.2	2274.4	0.08	0.8599
Gm3724	2296.2	2064.4	2429.2	1900.2	2142.8	1598.7	-0.15	0.7352
Rnpep	2295.2	2258.4	2421.1	2178.3	2473.5	2336.9	0.03	0.9805
Krtcap2	2295.2	2695.7	2263.0	2360.9	2382.1	2093.2	-0.18	0.5400
Coq2	2294.1	2137.3	2407.0	2250.5	2265.0	2008.1	0.00	1.0000
Pdia2	2293.0	4462.2	4263.0	4927.7	3117.2	4977.2	0.09	0.9667
Nol7	2293.0	2194.7	2192.4	2128.4	2271.2	1917.5	-0.06	0.9635
Srm	2293.0	2307.6	1865.0	2220.7	1956.0	2221.0	-0.07	0.9137
Sesn1	2292.0	2437.9	2599.5	2488.3	2741.4	2270.8	0.03	0.9972
Armc5	2292.0	2359.6	2161.2	2265.3	2553.5	2225.5	0.01	1.0000
Phf10	2292.0	2438.8	2331.5	2171.9	2134.6	2181.1	-0.15	0.6422
Wwtr1	2290.9	2125.4	2233.7	2611.4	2433.4	3075.1	0.29	0.2013
Pcgf5	2290.9	2047.1	2008.1	2250.5	2296.9	2192.9	0.11	0.8000
Zfp746	2290.9	2200.1	2107.8	2406.5	2424.2	2128.6	0.07	0.9126
Sugt1	2290.9	2325.8	2388.9	2042.4	2148.0	2120.4	-0.13	0.7535
Brsk2	2289.9	2395.1	2193.4	2271.7	2194.2	2143.1	-0.10	0.8217
Fhod3	2289.9	1982.4	2166.2	1835.4	1708.5	1718.3	-0.18	0.5345
Phf14	2288.8	2415.1	2534.0	2675.1	2718.8	2767.1	0.16	0.6674
Med23	2287.7	2416.9	2146.1	2521.2	2402.6	2652.1	0.07	0.9286
Mms19	2287.7	2357.7	1881.1	2391.7	2299.9	2460.1	0.04	0.9597
Rab7-ps1	2287.7	2273.0	2253.9	2177.2	2346.1	2093.2	-0.04	0.9672
Pgm3	2287.7	2294.0	1988.9	2056.2	2037.1	1976.4	-0.15	0.6881
Tenm4	2286.7	2390.5	2628.7	2863.0	2518.6	3673.8	0.27	0.3622
Socs2	2286.7	1967.8	2537.0	2393.8	2305.1	2118.6	0.14	0.7168
Etfdh	2286.7	2187.4	2289.2	2162.4	2186.0	1940.2	-0.06	0.9500
Cers5	2285.6	2355.9	2205.5	2360.9	2324.6	2069.7	-0.05	0.9487
Calcrl	2284.5	2192.8	2308.3	2974.4	2433.4	2742.7	0.26	0.2591
Gm49336	2284.5	1925.9	2089.7	2206.9	2110.0	2235.4	0.14	0.7161
Lig3	2284.5	2207.4	2229.7	2415.0	2330.7	2576.9	0.13	0.7682
Ddx49	2284.5	2299.4	2179.3	2184.7	1949.8	1884.0	-0.17	0.6074
Senp1	2282.4	2075.3	1957.7	2717.5	2291.7	2715.5	0.27	0.2768
Eif1a	2282.4	2437.9	2339.5	2350.3	2391.3	2089.6	-0.08	0.8833
Tada2b	2281.3	2009.7	2063.5	2243.0	2065.8	2259.0	0.09	0.8470
Cfap97	2281.3	2171.0	2115.9	2312.0	2175.7	2064.3	0.00	1.0000
Foxn3	2280.3	1938.7	2269.0	2408.6	2514.5	2795.2	0.33	0.0702
Sprtn	2280.3	2077.1	2059.4	2548.8	2445.7	2385.8	0.21	0.4117
Tmem135	2280.3	2154.6	2336.5	2418.2	2331.8	2384.0	0.12	0.7619
Flrt1	2280.3	1848.5	1905.3	1514.8	1553.5	1910.3	-0.17	0.6959
Eif4e2	2278.1	2118.1	2167.2	2402.3	2080.2	2283.5	0.07	0.9053
Pgm1	2277.1	2330.4	2279.1	2433.1	2399.5	2237.3	0.02	1.0000
Plppr2	2277.1	2420.6	2237.8	2056.2	1985.7	2034.4	-0.22	0.3485
Ptpn6	2277.1	2211.1	2017.1	1953.2	1959.1	1588.7	-0.23	0.4490
Fem1c	2276.0	2079.9	2034.2	2462.8	2372.8	2479.1	0.20	0.4552
Exoc2	2276.0	2420.6	2213.6	2476.6	2468.3	2266.2	0.00	1.0000
Smim1	2276.0	2326.8	2380.8	1915.0	2230.1	1847.8	-0.19	0.5343
Ccdc91	2274.9	2355.9	2338.5	2330.1	2484.7	1927.5	-0.05	0.9521
Ttbk2	2273.9	1858.5	2064.5	2136.9	2123.3	2309.7	0.18	0.5601
Iqce	2273.9	2180.1	1988.9	1908.7	1930.3	2061.5	-0.13	0.7638
Tnfrsf21	2272.8	2191.9	2169.3	2581.7	2478.6	2585.1	0.20	0.4758
Matn2	2272.8	2498.0	2617.6	2555.1	2575.1	3098.7	0.12	0.7939
Top3b	2272.8	2328.6	2277.1	2333.3	2116.1	2292.5	-0.04	0.9846
Morf4l1	2272.8	2011.5	2229.7	2161.3	1608.9	1893.1	-0.11	0.8410
Tmub2	2272.8	2199.2	2291.2	1983.0	2244.5	1786.2	-0.13	0.7845
Prkag2	2266.4	2407.8	2271.0	2205.9	2489.9	2484.5	0.00	1.0000
Uba3	2265.3	2105.4	2402.0	2239.9	2314.3	2119.5	0.05	0.9400
Ascc2	2265.3	2302.2	2196.5	2401.2	2142.8	2365.0	0.01	1.0000
AW551984	2265.3	2194.7	2663.0	2015.9	1989.9	1794.3	-0.19	0.5604
Dgcr8	2264.3	2189.2	2034.2	2389.5	2276.3	2295.2	0.08	0.8791
Tubb3	2264.3	1803.8	3388.4	2107.2	1837.9	2309.7	0.02	1.0000
Glmp	2264.3	2120.0	2327.4	2021.2	2278.4	1953.8	-0.04	0.9896
Ppt1	2264.3	2200.1	2293.2	2133.7	2252.7	1997.2	-0.05	0.9480
Ttc7b	2263.2	2319.5	2187.4	2489.3	2287.6	2326.9	0.03	0.9681
Thumpd1	2263.2	2273.9	2461.5	2307.8	2289.7	2067.9	-0.04	0.9610
Alg11	2263.2	2091.7	2004.0	1876.8	1911.8	1879.5	-0.14	0.7238
Fam126b	2262.1	2191.0	2214.6	2236.7	2151.1	2379.5	0.03	0.9694
Cspg4	2261.1	2024.3	2128.0	2850.2	2546.4	3394.8	0.44	0.0165
Smarcal1	2261.1	2357.7	2334.5	2325.8	2232.2	2251.8	-0.05	0.9487
Cyth3	2258.9	2109.0	2653.9	2415.0	2493.0	2728.2	0.21	0.5103
Tet1	2257.9	1963.3	2065.5	2292.9	2155.2	2606.8	0.21	0.5293
H1fx	2257.9	2089.9	2776.8	2152.8	1957.0	1830.6	-0.11	0.8558
Brap	2256.8	2086.2	2231.7	2338.6	2310.2	2083.3	0.08	0.8877

Arhgap11a	2256.8	2232.0	1616.1	2006.3	1902.6	2747.2	0.04	0.9806
Auh	2256.8	2540.8	2589.4	1785.5	1971.4	1541.6	-0.44	0.0131
Piezo1	2255.8	2365.0	2257.9	2696.3	2687.0	3903.0	0.33	0.1916
Chrm3	2255.8	2052.5	2113.8	2697.4	2370.8	2343.2	0.22	0.3767
Thbs1	2254.7	1801.1	1781.4	4540.2	3505.3	4313.3	0.97	0.0000
Eps15	2253.6	1989.7	2207.5	1994.6	2120.3	2290.7	0.07	0.9114
Ints7	2253.6	2392.4	2197.5	2364.1	2395.4	2668.4	0.06	0.9318
Rnps1	2253.6	2151.8	2134.0	2291.9	2196.2	2196.5	0.04	0.9834
Gm9762	2252.6	2149.1	2212.6	2111.4	2064.8	1995.4	-0.06	0.9148
Wbp1	2252.6	2281.2	2316.4	2057.3	2328.7	2001.8	-0.09	0.8717
Rtn2	2252.6	2023.4	2182.4	1745.2	1826.6	1470.1	-0.26	0.3407
Tln2	2251.5	1818.4	2607.5	2013.7	1740.4	2694.7	0.10	0.8816
Vps53	2251.5	2231.1	2222.7	2273.8	2151.1	2394.0	0.02	0.9815
Trappc3	2251.5	2242.9	2360.7	1954.3	2238.3	1972.8	-0.12	0.8207
Rlf	2250.4	2005.2	2098.7	2326.9	2154.1	2469.1	0.17	0.6074
Gart	2250.4	2395.1	2140.0	2563.6	2349.2	2752.6	0.10	0.8666
Tmem87a	2250.4	2182.8	2039.3	2160.2	2033.0	2254.5	-0.02	0.9925
Phlpp2	2248.3	1927.7	2178.3	2425.6	2106.9	2433.8	0.21	0.4470
Phf23	2248.3	2020.7	1899.2	2021.2	2124.4	1725.5	-0.05	0.9557
Zfp330	2248.3	2162.8	1971.8	2125.2	2005.3	1987.3	-0.07	0.9057
Lztfl1	2248.3	2200.1	2020.1	2005.3	1831.7	1883.1	-0.18	0.6055
Cd44	2247.2	1672.6	1958.7	2015.9	1974.5	2005.4	0.18	0.5576
Kank2	2246.2	2049.8	2460.4	2173.0	2271.2	2575.1	0.14	0.7430
Kmt5a	2246.2	2094.4	1944.6	2210.1	1969.3	2032.6	-0.01	0.9980
Uspl1	2245.1	2182.8	2108.8	2391.7	2024.8	2355.9	0.04	0.9557
2610002M06I	2245.1	2307.6	2222.7	1997.8	2193.2	2175.7	-0.10	0.8514
Fam43a	2244.0	2163.7	2550.1	2556.2	2718.8	2283.5	0.17	0.6287
Cox6c	2244.0	2438.8	2311.3	2181.5	2611.0	2086.9	-0.06	0.9395
Rasgrp3	2243.0	1919.5	1951.6	2323.7	2470.4	2557.9	0.29	0.1419
Map6	2243.0	2138.2	2569.3	2014.8	1841.0	2230.9	-0.09	0.8688
Coq9	2243.0	2273.9	2274.0	2141.1	2316.4	1902.1	-0.09	0.8714
Gpam	2240.8	2141.8	2229.7	2412.9	2367.7	2672.0	0.18	0.5634
Slc35g2	2240.8	1903.1	1819.6	1693.2	2036.1	1414.8	-0.14	0.7865
Fdft1	2239.8	2364.1	2131.0	2369.4	2193.2	2124.0	-0.06	0.9314
Scp2-ps2	2239.8	2484.4	2427.2	2247.3	2355.4	2006.3	-0.14	0.7201
Samd14	2238.7	2263.9	2575.3	2277.0	2455.0	2215.5	0.02	0.9921
Vps26c	2238.7	2268.5	2327.4	2061.5	2375.9	1807.9	-0.11	0.8445
Ttc8	2238.7	2168.2	2208.6	1957.5	1900.5	1697.4	-0.21	0.4466
Limch1	2237.6	1991.5	2233.7	2652.8	2285.6	2661.2	0.28	0.1931
Fam120c	2236.6	2223.8	1874.0	2325.8	2228.1	2717.3	0.13	0.7896
Tmem260	2236.6	2460.7	2302.3	2377.9	2467.3	2321.5	-0.02	0.9877
Insig2	2235.5	2059.8	2108.8	2118.8	2327.7	1804.3	0.00	1.0000
Numbl	2235.5	1963.3	2176.3	1957.5	1956.0	2044.3	-0.01	1.0000
Gabarapl2	2235.5	2203.8	2574.3	2076.4	2338.9	1734.6	-0.11	0.8410
Adk	2233.4	2480.7	2675.1	2454.3	2494.0	2366.8	-0.02	0.9948
Lamb2	2232.3	2132.7	2363.7	3375.7	3160.4	4418.4	0.63	0.0001
Trip4	2232.3	1908.6	2232.7	2022.2	2063.8	2119.5	0.08	0.8884
Ssx2ip	2232.3	2143.6	2255.9	2089.1	2284.5	2299.8	0.04	0.9616
Actr8	2232.3	1902.2	2062.5	1929.9	1960.1	1777.1	-0.03	0.9748
Jcad	2231.2	2049.8	2192.4	2871.5	2707.6	3299.7	0.45	0.0044
Prkab1	2231.2	2133.6	2181.4	2277.0	2392.3	2243.6	0.09	0.8374
Gm9774	2231.2	2395.1	2465.5	2450.0	2591.5	2256.3	0.02	0.9860
Apmap	2231.2	2386.0	2582.4	2316.3	2562.8	2331.5	0.01	1.0000
Car10	2229.1	2411.5	2490.7	2389.5	2665.5	2535.3	0.07	0.9483
Nup93	2228.0	2296.7	2094.7	2938.3	2622.3	2664.8	0.24	0.2817
Thap12	2228.0	2421.5	2264.0	2696.3	2533.0	2577.8	0.11	0.8184
Atg16l2	2228.0	2244.8	2162.2	2114.6	2102.8	1831.5	-0.13	0.7440
Irf1	2227.0	1855.8	2198.5	2678.3	2510.4	2385.8	0.36	0.0268
Poldip2	2225.9	2214.7	2209.6	2127.3	2180.8	1984.5	-0.07	0.8948
Dnal1	2225.9	2064.4	2073.5	1885.3	1813.3	1903.0	-0.14	0.7075
Tfe3	2223.8	2336.8	2171.3	2389.5	2347.2	2135.8	-0.01	0.9980
Hes6	2223.8	2201.9	2399.0	2041.3	2164.4	1815.2	-0.13	0.7655
Ankrd16	2222.7	2144.6	2122.9	2365.1	1768.1	2347.8	0.01	1.0000
Usp30	2222.7	1905.9	2083.6	2010.6	1976.5	1831.5	0.00	1.0000
Zfp62	2221.7	2183.7	2140.0	2522.2	2387.2	2567.0	0.17	0.5535
Smarce1	2221.7	2055.3	2328.5	2153.9	2221.9	1913.9	0.00	1.0000
Rufy2	2221.7	2171.9	2240.8	2203.8	1926.2	2137.6	-0.06	0.9382
Osbpl9	2220.6	2172.8	2300.2	2506.3	2348.2	2485.4	0.15	0.6539
Usp1	2219.5	2437.0	1804.5	2352.4	2145.9	2370.4	-0.03	0.9715
Ints13	2219.5	2354.1	2014.1	2372.5	2095.6	1969.2	-0.09	0.8591
Emc8	2218.5	2442.5	2119.9	2154.9	2215.7	2123.1	-0.14	0.6955
Usp45	2217.4	2144.6	1894.2	2109.3	2047.4	2016.3	-0.04	0.9842
Rnf146	2216.3	1941.4	2118.9	2409.7	2535.1	2340.5	0.27	0.1664
Socs4	2216.3	2061.6	1908.3	2447.9	2127.4	2379.5	0.15	0.7470
Ppard	2216.3	2104.5	2079.6	2067.9	2158.2	2093.2	0.00	1.0000
Tomm40	2215.3	2273.9	2284.1	2289.7	2094.6	1886.7	-0.11	0.8316

Elmsan1	2214.2	2179.2	2070.5	2386.3	2257.8	2634.0	0.14	0.7182
Zfp532	2214.2	2180.1	2099.7	2267.5	2171.6	2461.9	0.07	0.9011
Dpp6	2214.2	2156.4	1946.6	1432.0	1348.1	1539.8	-0.50	0.0010
Col5a1	2213.1	2281.2	2886.6	4409.6	4318.5	8065.0	0.52	0.0773
Scai	2213.1	2091.7	2094.7	2341.8	1962.1	2457.4	0.09	0.8791
Pgls	2213.1	2404.2	2142.1	2212.3	2281.5	2012.6	-0.11	0.7896
Efnb1	2211.0	2156.4	2547.1	1905.5	2045.3	2038.0	-0.12	0.7841
Sdf2	2211.0	2178.3	2514.9	1945.8	2170.6	1659.4	-0.17	0.6983
Galnt12	2211.0	2204.7	2055.4	1824.8	1901.6	1709.2	-0.25	0.3025
Filip1	2208.9	1866.7	1754.1	2077.4	1960.1	2173.0	0.11	0.8264
Hars	2208.9	1978.7	1998.0	1962.8	1952.9	2104.1	0.01	1.0000
Tmem184c	2208.9	2151.8	2033.2	1976.6	1953.9	1827.9	-0.15	0.6959
Thada	2207.8	2144.6	1775.3	2439.4	2155.2	2577.8	0.15	0.7168
Nsmce4a	2207.8	2238.4	2425.2	2417.1	2290.7	1949.2	-0.02	0.9895
Eya3	2206.7	2047.1	1940.5	2245.2	2278.4	2450.1	0.16	0.6472
Sh3bp5l	2206.7	2242.9	2303.3	2067.9	2220.9	1881.3	-0.11	0.7990
Nelfcd	2206.7	2476.2	2434.2	2298.2	2181.9	2176.6	-0.13	0.7692
Slc45a4	2206.7	2040.7	2166.2	1750.5	1827.6	2012.6	-0.13	0.7830
Krt8-ps	2206.7	2465.2	2262.0	1958.5	2197.3	1910.3	-0.24	0.3502
Taf15	2205.7	2478.9	2343.6	2742.0	2574.1	3119.5	0.18	0.5813
Kif13a	2205.7	2152.8	2095.7	2209.1	2341.0	2756.3	0.16	0.6893
Ncbp3	2205.7	2202.9	2181.4	2286.6	2455.0	2443.8	0.11	0.7920
Cops8	2203.5	2281.2	2257.9	2243.0	2237.3	2067.0	-0.05	0.9343
Meis3	2203.5	1941.4	1871.0	1597.6	1879.0	1639.4	-0.17	0.6317
Rnf168	2202.5	2184.6	2011.1	2413.9	2365.6	2470.0	0.14	0.7041
Taf2	2202.5	1928.6	1900.2	2182.5	2123.3	2214.6	0.14	0.7255
Atmin	2202.5	2181.0	2074.6	2136.9	2190.1	2256.3	0.01	1.0000
Aldh7a1	2201.4	2150.0	2298.2	2323.7	2368.7	2324.2	0.10	0.8037
Exph5	2201.4	2244.8	1676.6	2449.0	2130.5	2321.5	0.07	0.9311
Sdc4	2201.4	2301.2	2538.0	2020.1	2319.4	2386.7	-0.04	0.9925
Gm10282	2201.4	2416.9	2307.3	2252.6	2365.6	2125.8	-0.08	0.8772
Ubl4a	2199.3	2288.5	2253.9	2228.2	2205.5	2035.3	-0.07	0.9314
Adgra2	2198.2	2080.8	2592.4	2456.4	2378.0	3532.5	0.29	0.3439
Vps8	2197.1	2656.5	2487.6	2664.5	2584.3	2449.2	-0.02	0.9895
5830417I10Ri	2196.1	2098.1	1890.2	2268.5	1947.8	2346.9	0.06	0.9377
Sf3a2	2195.0	2130.0	2092.7	2147.5	2165.4	2663.9	0.11	0.8314
Gm12258	2195.0	1960.5	1801.5	1530.7	1701.3	1519.9	-0.27	0.2683
Dynll1	2194.0	2265.7	2333.5	2088.1	2253.7	2461.0	0.00	1.0000
Echs1	2194.0	2137.3	2360.7	2123.1	2283.5	1983.6	-0.02	0.9980
Grk6	2192.9	2132.7	2183.4	2168.7	2104.8	2182.0	0.01	1.0000
Marcksl1	2191.8	2434.3	3006.5	2475.5	2458.1	2854.1	0.06	0.9449
Esd	2191.8	2267.5	2179.3	2256.8	2362.6	2008.1	-0.03	1.0000
Lrrc8b	2190.8	2032.5	2220.6	2101.9	1964.2	2386.7	0.05	0.9474
Rbm7	2188.6	2119.0	2034.2	2265.3	2279.4	1793.4	0.00	1.0000
Pknox1	2188.6	2354.1	2114.9	2063.6	2425.2	2172.0	-0.06	0.9453
Tmcc2	2188.6	2409.7	2369.8	2134.8	2297.9	2047.0	-0.13	0.7524
Hcn4	2187.6	1877.6	1846.8	1726.1	1780.4	1697.4	-0.12	0.8031
Terf2	2186.5	1896.8	1998.0	2112.5	2232.2	2141.2	0.15	0.7023
Rps15-ps2	2186.5	2206.5	2594.4	2240.9	2527.9	2289.8	0.06	0.9343
Zkscan17	2186.5	2072.6	1983.9	2132.6	2043.2	2284.4	0.05	0.9500
Sash1	2184.4	2166.4	2424.2	2725.0	2555.6	3290.7	0.33	0.1252
Gm13461	2184.4	2540.8	2204.5	2615.6	2613.1	2305.2	0.01	1.0000
Kif19a	2184.4	2048.9	1950.6	2089.1	2100.7	1903.9	-0.01	1.0000
Inha	2184.4	2200.1	2030.2	1763.2	1785.5	1915.7	-0.24	0.3080
Spns1	2184.4	2171.9	2009.1	1782.3	1791.7	1566.1	-0.30	0.1567
Ripk1	2183.3	2048.9	2121.9	2268.5	2141.8	2134.0	0.07	0.8911
Tatdn2	2183.3	2203.8	2100.7	2380.0	2186.0	2326.9	0.06	0.9269
Igf2bp2	2183.3	2206.5	2163.2	1954.3	1745.5	2025.3	-0.18	0.5911
Cxadr	2182.2	1919.5	2564.2	2440.5	2284.5	2972.7	0.30	0.2336
Bend7	2182.2	2506.2	2366.7	2314.2	2543.3	1990.9	-0.09	0.8710
Trpc1	2181.2	1990.6	1861.0	1998.9	1984.7	1819.7	-0.04	0.9633
Gm9761	2181.2	2623.8	2370.8	2468.1	2549.4	2206.5	-0.08	0.8884
Zfp219	2180.1	2242.9	2071.5	2358.7	2177.7	2211.9	0.01	0.9931
Eloc	2180.1	2160.0	2293.2	2202.7	2190.1	1966.4	-0.03	0.9695
Rpl38-ps2	2180.1	2832.4	2809.1	2585.9	2735.3	2655.7	-0.05	0.9810
Snx12	2179.0	2051.6	1991.9	2229.2	2351.3	2259.9	0.13	0.7192
Rpl7-ps7	2178.0	2245.7	2276.1	2409.7	2369.8	2150.3	0.04	0.9603
Nfe2l2	2176.9	2017.9	2215.6	2378.9	2336.9	2382.2	0.19	0.4469
Use1	2176.9	1925.9	2286.1	1947.9	2168.5	1751.8	-0.02	0.9980
Galr1	2175.8	2266.6	2130.0	2200.6	2284.5	2171.1	-0.02	0.9944
Rnf214	2174.8	2011.5	1875.1	2121.0	1950.8	2221.9	0.05	0.9731
Sugp2	2174.8	2132.7	1740.0	2298.2	2003.2	2108.6	0.02	0.9863
Cep131	2172.6	2243.9	2078.6	1980.8	2022.7	2004.5	-0.14	0.6871
Wdr81	2171.6	2104.5	2060.4	1937.3	1764.0	2008.1	-0.13	0.7470
Rbm8a	2170.5	2056.2	2426.2	2182.5	2221.9	2072.4	0.04	0.9579
Cep192	2170.5	2340.4	1857.9	2256.8	2028.9	2645.8	0.02	0.9931

Cyp51	2169.4	2496.2	1927.4	2781.2	2574.1	2537.1	0.11	0.8113
Fam167a	2169.4	1529.6	1341.1	1500.0	1672.6	1712.8	0.03	0.9877
Fbxo45	2168.4	1884.9	1899.2	2059.4	2061.7	1926.6	0.07	0.9126
Gm5526	2167.3	2094.4	2120.9	2261.1	2386.2	1975.5	0.06	0.9334
Fam3a	2167.3	2236.6	2231.7	2182.5	2218.8	1913.0	-0.07	0.9083
Eif4e3	2167.3	2222.0	2128.0	1906.5	2098.7	1788.0	-0.17	0.6013
Pag1	2166.2	1999.7	2034.2	2021.2	1857.4	2354.1	0.04	0.9981
Csnk1g1	2165.2	1904.0	1898.2	2125.2	1997.0	2261.7	0.13	0.7682
Utp6	2165.2	2180.1	2112.8	2203.8	2190.1	2122.2	0.00	1.0000
Zcchc7	2165.2	2152.8	2093.7	2270.6	1815.3	2362.3	0.00	1.0000
Pacsin2	2164.1	2506.2	2536.0	2372.5	2364.6	2639.4	-0.01	1.0000
Eny2	2164.1	2324.0	2174.3	2349.2	2261.9	2168.4	-0.02	0.9810
Myo9a	2163.1	1973.3	1879.1	2434.1	1780.4	2567.0	0.15	0.7535
Mmut	2163.1	2060.7	1969.8	2178.3	2241.4	2054.3	0.06	0.9559
Mfsd6	2163.1	2046.2	2000.0	2021.2	2172.6	2180.2	0.05	0.9559
Gm8430	2163.1	2339.5	2519.9	2076.4	2340.0	1927.5	-0.13	0.7821
Pisd-ps1	2162.0	1577.9	1856.9	6774.8	5507.5	3023.5	0.68	0.0060
Mylk	2162.0	2286.7	2327.4	3128.4	3227.1	4703.7	0.56	0.0022
Irx2	2162.0	2794.1	2384.9	2895.9	2698.3	2411.2	-0.01	1.0000
Hexb	2160.9	1800.2	2057.4	1853.5	2029.9	1777.1	0.03	0.9866
Stard4	2159.9	3030.1	2395.0	3348.1	3017.6	3006.3	0.10	0.8762
Dnajc11	2159.9	2188.3	2118.9	2174.0	2120.3	2325.1	0.02	1.0000
Ptk2b	2159.9	2440.6	2558.2	2134.8	2187.0	1987.3	-0.18	0.6120
Smarcd1	2157.7	2028.9	2113.8	2273.8	2191.1	2164.8	0.10	0.8059
Tacc2	2156.7	2220.2	2587.4	2820.5	2707.6	3185.6	0.32	0.1177
Wdr43	2156.7	1946.9	2064.5	2461.7	2026.8	2242.7	0.16	0.6601
Gm47163	2156.7	2091.7	2157.2	2046.7	2096.6	1605.9	-0.12	0.8237
Mpp7	2156.7	2317.6	2234.8	1825.9	1808.1	1768.1	-0.32	0.0586
Ndufa13	2155.6	2257.5	2385.9	2114.6	2227.0	1949.2	-0.10	0.8445
Gtf2h1	2155.6	2273.9	2189.4	2137.9	2050.4	2009.9	-0.12	0.7702
Mphosph8	2154.5	2086.2	2224.7	2215.4	2204.4	2235.4	0.07	0.9005
Pdcd6	2154.5	2130.0	2100.7	2066.8	2195.2	1888.5	-0.05	0.9513
Cfdp1	2154.5	2247.5	2123.9	2137.9	2119.2	2065.2	-0.08	0.8836
Mapk10	2153.5	1880.4	1995.0	2081.7	2154.1	2122.2	0.14	0.7107
Zfp46	2152.4	1765.6	1740.0	2502.1	2531.0	2632.2	0.44	0.0064
Polr1a	2152.4	2204.7	2052.4	2363.0	2101.8	2817.0	0.13	0.7865
Shtn1	2152.4	2107.2	2086.6	2278.1	2163.4	2240.0	0.07	0.9051
Cntrl	2152.4	1973.3	1818.6	2094.4	1597.6	2221.9	0.00	1.0000
Nfix	2151.3	2253.0	2660.9	3193.1	3229.1	4906.6	0.42	0.2029
Parm1	2151.3	1825.7	2498.7	2359.8	1961.1	2873.1	0.25	0.4441
Prpsap1	2150.3	1989.7	2176.3	2132.6	2050.4	2077.8	0.05	0.9478
Dmtf1	2150.3	2387.8	2371.8	2340.7	1947.8	2257.2	-0.10	0.8391
Rap1a	2148.1	2332.2	2426.2	2213.3	2290.7	1875.0	-0.11	0.8559
Slc9a3r1	2147.1	2067.1	2239.8	1888.5	1954.9	1949.2	-0.10	0.8217
Lrrc45	2147.1	2102.6	2167.2	1991.5	1905.7	1924.8	-0.11	0.7873
Babam1	2147.1	2417.9	2233.7	2049.8	2181.9	2006.3	-0.18	0.5429
G2e3	2146.0	2070.8	1569.8	2257.9	2192.1	2420.2	0.15	0.7357
Fam149b	2146.0	2135.4	2061.5	2237.7	2105.9	2115.9	0.01	1.0000
Pdk2	2146.0	1980.6	2292.2	2021.2	2036.1	1964.6	0.00	1.0000
Josd1	2146.0	2038.9	2273.0	2081.7	2099.7	1754.5	-0.06	0.9376
Dhx8	2144.9	1982.4	1895.2	2242.0	2238.3	2246.3	0.16	0.6416
Atg3	2144.9	2111.8	2320.4	2110.3	2362.6	1969.2	0.01	1.0000
Ubap1	2144.9	2040.7	2060.4	2019.1	2340.0	1853.2	0.01	1.0000
Rrn3	2143.9	2109.0	2062.5	2145.4	2214.7	2204.7	0.05	0.9507
Camk2g	2142.8	2008.8	2100.7	2222.9	1944.7	2117.7	0.04	0.9559
Nck1	2142.8	1963.3	1759.2	2118.8	2089.4	1688.4	0.00	1.0000
Ccdc88c	2142.8	2283.9	2084.6	1936.3	1833.8	2381.3	-0.11	0.8237
Sema4c	2141.7	2061.6	1906.3	2039.2	2287.6	2375.8	0.11	0.8599
Nck2	2141.7	2402.4	2589.4	2186.8	2160.3	2014.4	-0.16	0.6869
8030462N17R	2140.7	1778.3	2119.9	2386.3	2307.1	2148.5	0.29	0.1366
Pnpla2	2140.7	2041.6	2215.6	2038.2	2273.2	2163.0	0.06	0.9256
Ppp2r2d	2140.7	2077.1	2287.1	2191.0	2338.9	2033.5	0.05	0.9681
Gatd1	2140.7	2001.5	2197.5	1674.1	1988.8	1519.9	-0.20	0.5853
Kalrn	2139.6	1828.4	2355.7	2119.9	1918.0	2379.5	0.15	0.7295
Usp46	2139.6	1753.7	1789.4	1952.2	1929.3	1942.9	0.10	0.8404
Stxbp5l	2139.6	2061.6	1822.7	2127.3	1929.3	2205.6	0.03	0.9858
Tuft1	2139.6	2018.8	2350.6	2097.6	2114.1	2024.4	0.02	0.9931
Gm20300	2138.5	1828.4	2118.9	1796.1	1897.4	1792.5	-0.03	0.9731
Gm43952	2138.5	9453.7	2867.5	2786.5	1869.7	3398.5	-0.59	0.0272
Serpina10	2136.4	1876.7	1831.7	1910.8	1924.1	1615.0	-0.05	0.9457
Kat2a	2136.4	2379.6	2207.5	2305.7	2059.7	2201.0	-0.09	0.8560
Get4	2136.4	2281.2	2177.3	2045.6	2128.5	1880.4	-0.15	0.7192
Zfp251	2135.3	1897.7	2058.4	1853.5	1929.3	1797.1	-0.05	0.9521
Kat5	2135.3	2089.9	2126.9	1974.5	2073.0	1775.3	-0.10	0.8421
Arhgap39	2135.3	2293.0	1996.0	1926.7	1676.7	1986.4	-0.24	0.3311
Adgrl2	2134.3	1929.6	2167.2	2559.4	2361.5	3003.5	0.37	0.0526

Eif2s2	2134.3	2030.7	2112.8	2137.9	2004.2	2074.2	0.02	0.9884
Tbc1d13	2134.3	2077.1	2056.4	2028.6	2058.6	1980.0	-0.04	0.9690
Dusp1	2133.2	1888.6	2522.9	7978.5	5714.9	6748.0	1.49	0.0000
Cntln	2133.2	2142.7	1997.0	2202.7	2132.6	2313.3	0.05	0.9457
Prkci	2133.2	2098.1	2153.1	2099.7	2021.7	2204.7	0.00	1.0000
Lemd3	2132.2	1920.4	2122.9	2325.8	2009.4	2175.7	0.14	0.7302
Fam104a	2132.2	2150.0	2282.1	2288.7	2250.6	1951.0	0.00	1.0000
Tmco1	2132.2	2212.9	2225.7	2018.0	2374.9	1995.4	-0.05	0.9840
Exoc3l2	2131.1	2004.3	2050.4	2352.4	2373.9	2307.9	0.20	0.4299
Mtf1	2131.1	2073.5	1904.3	2106.1	2320.5	2120.4	0.07	0.9126
Ube2e1	2131.1	2041.6	2165.2	2022.2	2044.3	1809.7	-0.06	0.9186
Uap1	2130.0	2121.8	2012.1	2130.5	2086.4	1962.8	-0.03	0.9695
Rpap1	2130.0	2220.2	2002.0	2119.9	1896.4	2150.3	-0.08	0.8762
Gm11223	2129.0	2130.0	2021.2	1890.6	2004.2	2339.6	-0.02	0.9896
Cep164	2129.0	2157.3	2075.6	1947.9	1682.9	2156.6	-0.13	0.7786
Opa3	2127.9	1956.0	1904.3	2066.8	1971.4	1850.5	0.00	1.0000
Ifrd1	2127.9	2286.7	2212.6	2245.2	2162.3	2229.1	-0.03	0.9681
Lyrm9	2127.9	2039.8	2052.4	1869.4	1718.8	1824.2	-0.16	0.6131
Txndc16	2126.8	2143.6	2119.9	2038.2	2285.6	2026.2	-0.01	0.9931
BC031181	2126.8	2049.8	1856.9	1934.1	2075.1	1687.5	-0.09	0.8809
Ralgps1	2126.8	2110.8	2016.1	1977.7	1730.1	2080.6	-0.11	0.8213
Tmprss4	2126.8	1884.9	2379.8	1752.6	1835.8	1603.2	-0.15	0.7420
Map3k12	2125.8	1897.7	2203.5	2015.9	1908.7	1948.3	0.02	1.0000
Lysmd3	2124.7	1763.7	1561.7	1885.3	1697.2	1772.6	0.00	1.0000
Slc35a5	2124.7	1963.3	2002.0	2030.7	2031.9	1847.8	-0.01	1.0000
Cd300lg	2123.6	2055.3	2112.8	2354.5	2473.5	2492.7	0.22	0.3321
Adgre5	2123.6	1880.4	2024.2	2088.1	2027.8	2249.0	0.14	0.7544
Ndufa6	2123.6	2201.0	2320.4	2010.6	2336.9	1903.0	-0.07	0.9114
Cog2	2123.6	2018.8	1877.1	1921.4	1908.7	1859.6	-0.08	0.8830
Ppil4	2122.6	2161.9	1942.6	2060.5	2100.7	2281.6	0.00	1.0000
Rnf19b	2122.6	1881.3	2175.3	1826.9	1837.9	1555.2	-0.13	0.7978
Ssh2	2121.5	1844.8	1844.8	2213.3	1821.5	2509.9	0.18	0.6550
Usp6nl	2120.4	2090.8	2335.5	2158.1	2061.7	2445.6	0.06	0.9557
B3gnt3	2120.4	1913.2	1953.6	1794.0	2011.4	2017.2	0.01	1.0000
Prkag1	2120.4	1968.7	2144.1	2035.0	2165.4	1795.2	0.00	1.0000
Srxn1	2120.4	2052.5	2002.0	2080.6	2069.9	1940.2	-0.02	1.0000
Ehbp1	2120.4	2089.9	2018.1	2036.0	2056.6	2071.5	-0.02	1.0000
Topors	2119.4	2021.6	2031.2	2330.1	2366.7	2374.0	0.20	0.4362
Mars	2119.4	2275.7	1990.9	2397.0	2371.8	2436.5	0.09	0.8816
Fbxl18	2119.4	2030.7	2045.3	1847.1	1938.5	1989.1	-0.07	0.8890
Habp4	2119.4	2124.5	2251.9	1963.9	2039.1	1872.2	-0.11	0.8314
Atf1	2117.2	1882.2	2100.7	2141.1	2217.8	2211.0	0.18	0.5354
Cdk5rap2	2117.2	2134.5	1629.2	2272.8	2045.3	2831.4	0.17	0.7134
Gm5422	2117.2	2272.1	1875.1	2279.1	2272.2	2226.4	0.02	0.9970
Hgfac	2117.2	1810.2	1857.9	1767.5	1769.1	1453.8	-0.13	0.7830
Dalrd3	2117.2	2370.5	2176.3	1886.4	1829.7	1659.4	-0.34	0.0522
Gc	2116.2	2028.9	1578.8	2537.1	2133.6	2410.3	0.21	0.5532
Idh1	2116.2	2076.2	2477.6	2160.2	2229.1	1962.8	0.00	1.0000
Ctnnbl1	2116.2	2175.5	2244.8	2181.5	2212.7	2046.1	-0.02	1.0000
Llgl1	2116.2	2196.5	2053.4	2112.5	2061.7	2187.4	-0.04	0.9903
Irf2	2115.1	2129.1	2161.2	2364.1	2320.5	2225.5	0.10	0.8056
Enpp4	2113.0	1940.5	1868.0	1940.5	2104.8	1972.8	0.04	0.9679
Rab36	2113.0	2060.7	2006.0	1728.2	1837.9	1829.7	-0.18	0.5521
Orc3	2110.8	2139.1	2036.3	2094.4	2197.3	2311.5	0.04	0.9517
Alyref	2110.8	2160.0	2066.5	2229.2	1965.2	2048.0	-0.04	0.9649
Ints8	2109.8	1950.5	1940.5	2060.5	2046.3	1776.2	0.00	1.0000
Ctnnal1	2109.8	2206.5	2219.6	2162.4	1769.1	2209.2	-0.09	0.8759
Cry1	2108.7	1836.6	2143.1	2100.8	1946.7	1914.8	0.07	0.8919
Trp53	2108.7	2294.9	2265.0	2318.4	2226.0	2336.0	0.01	1.0000
Rab24	2108.7	1948.7	2245.8	1794.0	1862.5	1531.7	-0.18	0.6196
Dcun1d4	2106.6	1782.0	1900.2	1636.9	1776.3	1644.9	-0.10	0.8455
Dchs1	2105.5	1998.8	2020.1	2387.4	2315.3	3203.7	0.33	0.1843
